# Assessment of Chinese suitable habitats of *Amomum tsao-ko* in different climatic conditions

**DOI:** 10.3389/fpls.2025.1561026

**Published:** 2025-05-08

**Authors:** Tian-yu Guo, Qing Yang, Da-ju Chen, Xiao-yu Wang, Qi-Qing Cheng, Shi Wang, Ming-li Hu

**Affiliations:** ^1^ School of Pharmacy, Hubei University of Science and Technology, Xianning, China; ^2^ Institute of Medicinal Biological Technique, Wenshan Academy of Agricultural Sciences, Wenshan, China

**Keywords:** environmental factor, species distribution, potential habitat, volatile oil, *Amomum tsao-ko*

## Abstract

**Introduction:**

*Amomum tsao-ko* Crevost et Lemaire is not only a traditional Chinese medicine but also a significant cash crop in the border regions of southwest China. However, challenges pertaining to its growing environment, yield, and overall quality have considerably impeded its development. This paper investigates the responses of *A. tsao-ko* to climatic challenges, aiming to contribute to the long-term stability and sustainability of the industry.

**Methods:**

The MaxEnt model, combined with ArcGIS software, was utilized to analyze key environmental factors and predict potential suitable habitats for *A. tsao-ko* under various climatic conditions. Furthermore, the volatile oils in *A. tsao-ko* samples from high-suitable habitats were analyzed using gas chromatography-mass spectrometry (GC-MS).

**Results:**

The results indicated that Bio04 (Temperature seasonality (standard deviation * 100)), Bio17 (Driest quarterly precipitation), and Bio12 (Precipitation of the wettest month) were the primary environmental factors influencing the distribution of *A. tsao-ko*. Under future climatic scenarios, it is expected to gradually adapt to new environmental conditions, with suitable habitats progressively shifting northward. The volatile oil extraction and GC-MS analysis revealed that the sample from Xishuangbanna (S8) exhibited not only the highest extraction rate (32.6 μL/g) but also the highest relative content of terpenes, particularly eucalyptol (29.26%).

**Discussion:**

S8 is regarded as a source of high-quality production that fulfills the criteria outlined in the Chinese Pharmacopoeia. The results show that Xishuangbanna can be used as a high-quality production area for *A. tsao-ko* planting, and large-scale artificial planting can be carried out to realize the sustainable development of *A. tsao-ko* industry and ecology.

## 1 Introduction

*Amomum tsao-ko* Crevost et Lemaire refers to the dried ripe fruit of *Amomum* Roxb., which belongs to the Zingiberaceae family. This species prefers warm and humid environments at elevations between 1100 and 1800 meters (Wu, 1987). China is the largest global producer of *A. tsao-ko*, accounting for over 80% of total production, with the primary growing regions being the provinces of Yunnan, Guangxi, Guizhou, Chongqing, and Sichuan. Significantly, Yunnan is responsible for 95% of the nation’s yield (Liu et al., 2021a). *A. tsao-ko* is recognized not only as a precious spice but also as a crucial herbal remedy (Jia et al., 2021). It is frequently used to treat conditions such as cold and dampness, abdominal distension, vomiting, and fevers linked to malaria and plague (Wen et al., 2024). Consequently, this medicinal and culinary plant is predominantly employed in China for enhancing flavor, as a food additive, and in the formulation of traditional Chinese medicines, among various other uses (Yang et al., 2014; Liang et al., 2024).

As market demand for *A. tsao-ko* increases, it has emerged as a significant cash crop in the southwestern border region, highlighting the importance of expanding its cultivation area. Global climate change is impacting the habitats of medicinal plants and altering the accumulation of chemical constituents (Wang et al., 2019a; Rana et al., 2020). Research indicates that environmental factors such as light, temperature, and humidity are critical determinants of fruiting rates and the accumulation of secondary metabolites (Zhao et al., 2016; Koch et al., 2017; Zhang et al., 2018). Furthermore, variations in yield, morphological characteristics, and volatile oil composition of *A. tsao-ko* have been observed across different latitudes, altitudes, and soil conditions (Yang et al., 2014; Kazemi et al., 2017; Li et al., 2021). Despite its long history of cultivation in China, *A. tsao-ko* ‘s growth and quality are highly dependent on environmental conditions. The cultivation areas remain semi-wild, typically characterized by abundant flowers but limited fruit production, resulting in poor and unstable yields and significant damage to natural resources (Yang et al., 2020). Therefore, it is crucial to establish effective analytical methods and scientifically select appropriate planting habitats to optimize *A. tsao-ko* yields and prevent resource wastage.

MaxEnt (maximum entropy) model has high accuracy in predicting the potential distribution of species (Elith et al., 2010; Tarroso et al., 2012). In recent years, it has been extensively utilized for predicting suitable habitats for Chinese herbs and cash crops. For instance, Yang et al. (2023a) assessed and predicted the distribution of *Zanthoxylum nitidum* in China under various climate scenarios using the MaxEnt model, as well as identifying and evaluating the species through high-performance liquid chromatography (HPLC) techniques, hierarchical cluster analysis, and principal component analysis. Similarly, Zhan et al. (2022) employed the MaxEnt model in conjunction with HPLC to assess suitable cultivation habitats for *Panax notoginseng* under different climatic conditions in China. Furthermore, Wan et al. (2021) investigated the factors influencing the quality of *Codonopsis pilosula* by integrating chromatographic fingerprinting with MaxEnt modeling. The MaxEnt model is characterized by its high prediction accuracy, effective application, and user-friendly procedures, thereby providing a scientific foundation for biodiversity conservation and management (Li et al., 2019; Liu et al., 2021b). It can also provide information about the area of distribution of species, the suitable range of environmental variables, etc (Adhikari et al., 2012; Xu et al., 2021).

This research employed MaxEnt software along with ArcGIS software to investigate *A. tsao-ko* and identify key environmental factors that affect its geographical distribution. The study also aims to forecast how climate change over time will impact the growth environments of *A. tsao-ko* and alter the distribution of its potential habitats. This paper proposes strategies and management measures to adapt to climate change. Meanwhile, gas chromatography-mass spectrometry (GC-MS) was used to determine the volatile oils of *A. tsao-ko* from different regions. Cluster analysis was used to analyze the volatile oil constituents and to compare the quality of *A. tsao-ko* from different regions. This study is the first to integrate the MaxEnt model, ArcGIS technology, and GC-MS in *A. tsao-ko*. By leveraging big data analytics, it accurately predicts the distribution patterns of suitable habitats and volatile oil composition variations under different climate change scenarios. The findings provide a scientific basis for the adaptive management and future cultivation planning of the *A. tsao-ko* industry, demonstrating significant innovative and practical value for promoting sustainable development and enhancing farmers’ economic benefits.

## 2 Materials and methods

### 2.1 Prediction of potentially suitable habitat

#### 2.1.1 Distribution data collection and processing

The distribution data of *A. tsao-ko* in China were sourced from online databases, including the Chinese Virtual Herbarium (http://www.cvh.ac.cn/), the National Specimen Information Infrastructure (http://www.nsii.org.cn/), and the Global Biodiversity Information Facility (GBIF). To minimize sampling deviations, the data points were analyzed in the field using ArcGIS 10.4.1 software. In order to reduce the overfitting of the model caused by sampling bias, ArcGIS was used to select neighborhood analysis, set a buffer with a radius of 5km, randomly retain a distribution point within 10km, and screen out other distribution points. The final distribution sample points (**Supplementary Figure S1**) were then obtained for further data analysis.

#### 2.1.2 Selection and dealing with environmental factors

Climate factors such as temperature, precipitation, and humidity were sourced from the WorldClim (version 2.1) website (http://www.worldclim.org) for the period from 1970 to 2000. Paleo-climate data (CURRENT) served as a reference standard. Past climate scenarios for the Last Glacial Maximum (LGM) and the Middle Holocene (MH) were selected, along with future climate scenarios for the periods 2041–2060 and 2081-2100 (Yang et al., 2023a). In addition, 11 soil factors, including vegetation type and soil cover, as well as three topographic factors–elevation, slope, and aspect–were collected from the Harmonized Word Soil Database (version 1.2) (Harmonized World Soil Database v1.2 | FAO SOILS PORTAL | Food and Agriculture Organization of the United Nations) and the WorldClim website. This resulted in a total of 33 independent environmental factors selected for the study (**Supplementary Table S1**).

In order to mitigate multicollinearity among environmental factors and prevent overfitting of the model, Spearman correlation and variance inflation factor (VIF) of all environmental variables were analyzed. The high contribution factors were retained, especially the factors with correlation coefficients of |r| > 0.8 and VIF <10 (Karakaya and Yücel, 2021; Wu et al., 2024). Through analyses conducted with ArcGIS, MaxEnt, and SPSS, we ultimately identified 14 valid environmental factors to construct a prediction model for *A. tsao-ko* growth, ensuring the accuracy and stability of the model predictions.

#### 2.1.3 MaxEnt modeling and suitable habitat delineation

To quantitatively assess the contribution of each environmental factor to the construction of the prediction model, this study employed the MaxEnt model to analyze the contributions of 14 selected environmental factors. The modeling parameters included the Bootstrap method for sampling, with the output format set to Logistic. A random selection of 75% of the distribution points was utilized for training, while the remaining 25% was reserved for testing. After 10^6^ iterations, the model was operated 10 times (Zhao et al., 2021; Hosni et al., 2022). To evaluate model uncertainty and prediction accuracy, multiple random resampling of the dataset was conducted using Bootstrap resampling. The accuracy of the model’s predictions was assessed using the area under the receiver operating characteristic (ROC) curve (AUC) (Zhan et al., 2022; Wu et al., 2024). The Jackknife method was used to determine the degree of contribution of each environmental factor. In addition, the Maximum Test Sensitivity Plus Specificity Logistic threshold (MTSPS) was used to delineate the suitable potential habitats for *A. tsao-ko* (Ramos et al., 2019; Yang et al., 2023a). The habitats were categorized into four levels: 0-MTSPS indicating unsuitable habitats, MTSPS-0.3 indicating low-suitable habitats, 0.3-0.5 indicating medium-suitable habitats, and 0.5–1 indicating high-suitable habitats. Subsequently, the various categories of suitable habitats were quantified.

### 2.2 Quality evaluation of the volatile oil of *A. tsao-ko* from different regions

#### 2.2.1 Sample collection

Samples were collected from the Honghe Hani and Yi Autonomous Prefecture, Lincang, Nujiang Lisu Autonomous Prefecture, Baoshan, Wenshan Zhuang and Miao Autonomous Prefecture, and Xishuangbanna Dai Autonomous Prefecture in Yunnan Province (see **Supplementary Table S2**). The samples were collected in November 2022. The samples were identified as *A. tsao-ko* by Yang Qing, a Deputy Senior Agronomist at the Academy of Agricultural Sciences in Wenshan Zhuang and Miao Autonomous Prefecture, Yunnan Province.

#### 2.2.2 Extraction of volatile oil

The extraction procedure was carried out following Method A, as described in the four general rules 2204 of the 2020 version of the Chinese Pharmacopoeia for measuring volatile oils (China Pharmacopoeia Commission, 2020). The reflux was heated until the oil volume in the extractor reached a stable state; thereafter, the upper layer of volatile oil was collected and quantified. This procedure was repeated three times. Samples of volatile oil from *A. tsao-ko*, sourced from various regions, were transferred in 5 μL aliquots and then diluted to a final volume of 4 mL with an acetone solution. The mixture was thoroughly mixed and subsequently filtered through a 0.4 μm microporous membrane, resulting in a filtrate designated as the sample solution. This solution was then subjected to analysis using gas chromatography-mass spectrometry (GC7890B-MS5977A, Agilent, USA).

GC conditions: Column, VF-1701 MS flexible quartz capillary column (30 m×0.25 mm×0.25 μm). Column temperature: 40°C, retained for 1 min; warmed to 100°C at 30°C/min, retained for 1 min; then warmed to 220°C at 3°C/min, and then warmed to 280°C at 5°C/min, retained for 14 min. Carrier gas: He, 1.0 mL/min; non-split injection; injection volume of 1.0 μL. Mass spectrometry conditions: EI ion source, ionization voltage of 70 eV; scanning range m/z: 40–650 amu; four-stage rod temperature 150°C; ion source temperature 230°C; electron multiplier voltage 2300V; GC/MS interface temperature 280°C (Wang et al., 2021; Qin et al., 2022). Identification of compounds was performed by comparing with the NIST 20.L standard mass spectral library and was further validated through references from previously published literature.

#### 2.2.3 Systematic cluster analysis

Hierarchical Clustering is a hierarchical clustering analysis method that gradually merges or segments sample sets by calculating similarity measures between samples (such as Euclidean distance, Manhattan distance, or correlation coefficient, etc.) to form a hierarchical clustering structure. In this clustering method, samples belonging to the same cluster are highly similar, while samples of different clusters show significant differences (Buchgraber et al., 2004). This research investigated the volatile oil content of *A. tsao-ko* from various sources using SPSS 26.0 for statistical analysis. For systematic cluster analysis, the Euclidean squared distance was employed as the measurement, applying the homogeneous correlation method among groups (Yang et al., 2023b).

## 3 Results and analysis

### 3.1 Evaluation of model prediction accuracy

**Figure 1:**
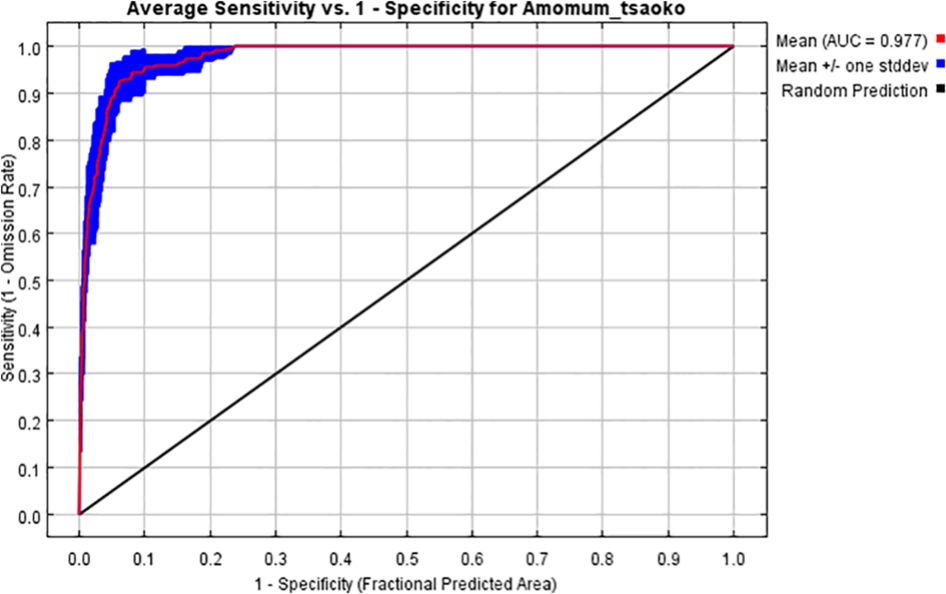
ROC curve of MaxEnt model.

The distribution prediction of habitats for *A. tsao-ko* was conducted using MaxEnt. After 10 cycles, an average ROC curve was generated. AUC values typically range from 0 to 1, with a value of 0 indicating no predictive power (similar to random chance) and a value of 1 representing perfect prediction (Porfirio et al., 2014). AUC values nearing 1 demonstrate an enhanced model performance. In this study, the average training AUC value obtained from the ROC curve was 0.977 and the standard deviation is 0.007. (**Figure 1**), indicating that the model was built with a remarkably high accuracy, enabling an effective assessment of the favorable habitats for the growth of *A. tsao-ko*.

### 3.2 Environmental factors affecting the growth of *A. tsao-ko* and the extent of their influence

**Figure 2:**
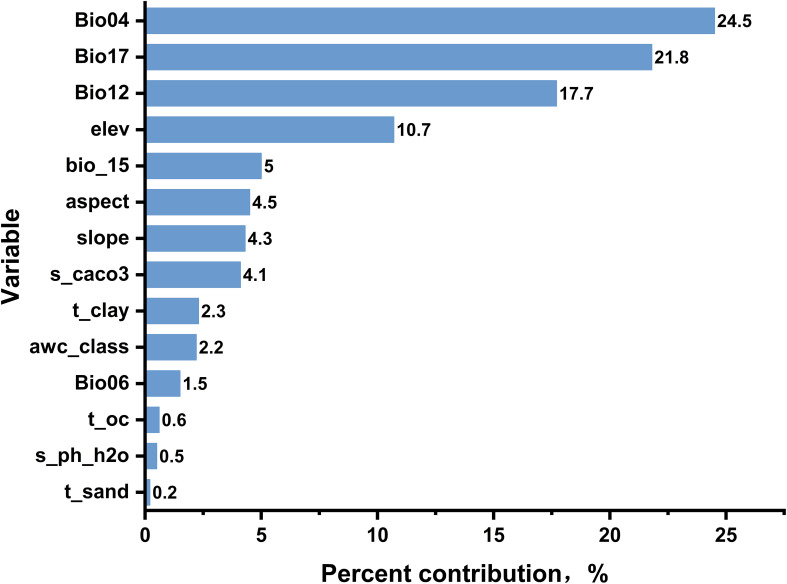
Estimates of relative contributions of the environmental variables.

In the MaxEnt model, Percent Contribution is a key metric used to measure the relative contribution of each environment variable (feature) to the model’s predictions (Shi et al., 2023). In order to characterize the impact of various environmental factors on the results of prediction model construction, MaxEnt model was used to analyze the contribution rates of 14 environmental factors. In the univariate analysis illustrated in **Figure 2**, Bio04, Bio17, and Bio12 each contributed over 15%, with Bio04 being the most prominent at 24.5%. This reveals the magnitude and frequency of temperature fluctuations, indicating that these changes significantly affect *A. tsao-ko*. Consequently, temperature emerges as the primary environmental factor influencing the identification of suitable habitats for *A. tsao-ko*. Together, the cumulative effects of climatic variables—such as Bio04, Bio06, Bio12, Bio15, and Bio17—reach a total of 70.5%, underscoring the crucial influence of climate on the distribution of *A. tsao-ko.* In contrast, topographical elements—such as aspect, elevation, and slope—contribute a combined 19.5%. These topographical factors affect both direct and indirect variables related to water distribution, light accessibility, and soil erosion. Additionally, soil parameters, including t_clay, awc_class, t_oc, t_sand, s_caco3, and s_ph_h2o, account for a total of 9.9%. Although the individual contributions of each soil factor might appear minor, their overall influence is vital for the growth of *A. tsao-ko*.

As indicated in **Table 1**, the range of suitable habitats (both high and medium suitable habitat) for the distribution of *A. tsao-ko* concerning Bio04 spans from 140.256 to 1600.848. Specifically, the high-suitability habitat ranges from 140.256 to 977.688, with the optimal condition identified at 460.451. Furthermore, the most suitable conditions for the environmental factors Bio17 and Bio12 are recorded at 59.283 mm and 1666.240 mm, respectively. These three ecological factors provide critical insights that significantly influence *A. tsao-ko* and are essential for research and forecasting suitable habitats for *A. tsao-ko* in designated regions. Seasonal temperature variation, which refers to the fluctuation range of temperature throughout the year, has a significant impact on the growth and development of plants. Extreme temperature changes can affect the photosynthesis, respiration, and transpiration of plants, thereby influencing their growth rate and morphology (Cheng et al., 2024b; Yin et al., 2024). For a plant like *A. tsao-ko*, seasonal temperature variation may affect its seed germination rate, flowering, and fruiting times. The amount of precipitation directly affects the water supply for plants, thereby influencing their growth and distribution (Zhang et al., 2025). For some drought-tolerant plants, the less precipitation in the driest quarter, the better these plants can grow and reproduce (Bao et al., 2024; Krasensky and Jonak, 2012). However, *A. tsao-ko* has a high demand for water, and too little precipitation in the driest quarter may limit its growth and even cause its death. Therefore, the distribution of *A. tsao-ko* may be significantly influenced by the precipitation in the driest quarter, and it is usually distributed in areas with relatively sufficient precipitation. Months with high precipitation can provide sufficient water for plants, promoting their growth and reproduction. For *A. tsao-ko*, the adequate precipitation in the wettest month is helpful for its growth and development, especially during the growth period and flowering period. If the precipitation in the wettest month is insufficient, it may affect *A. tsao-ko* ‘s flowering and fruiting, thereby affecting its distribution and population size.

**Table 1:** Range of suitable habitat for key environmental variables affecting the potential distribution of *Amomum tsao-ko*.

Environmental factors	Total suitable habitat range	High-suitable habitat range	Optimal value	Unit
Bio04	140.256-1600.848	140.256-977.688	460.451	–
Bio17	-62.800-274.059	-62.800-158.758	59.283	mm
Bio12	379.984-4654.00	790.168-4654.00	1666.240	mm

### 3.3 Distribution of suitable habitat for *A. tsao-ko* under different climatic conditions

#### 3.3.1 Distribution of suitable habitats under current climatic conditions

**Figure 3:**
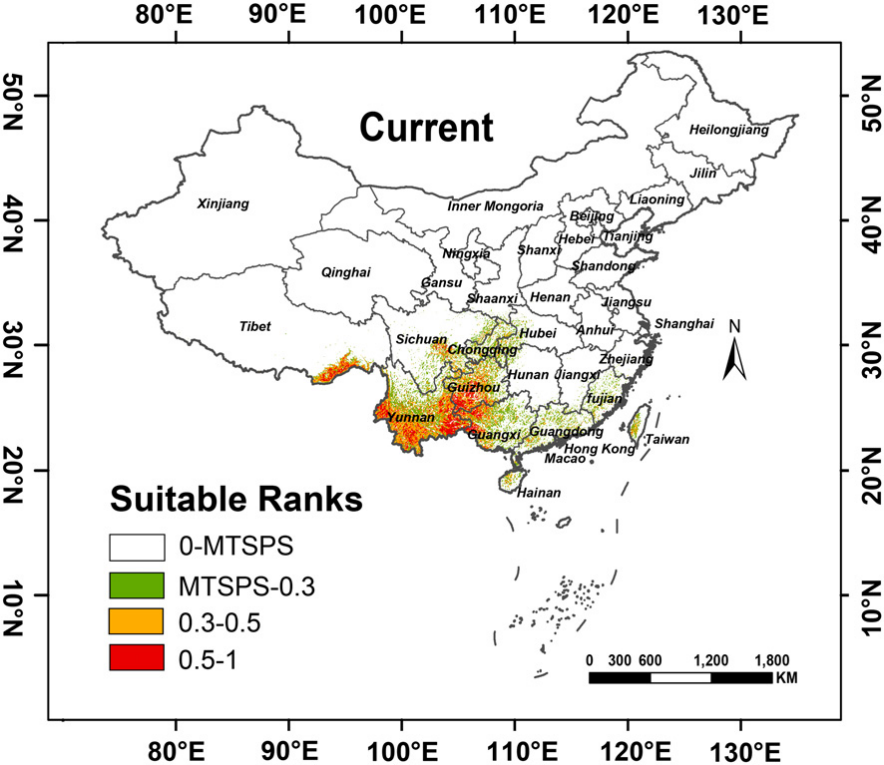
Distribution of suitable habitats for *Amomum tsao-ko* under the current climate conditions. [Maximum Test Sensitivity Plus Specificity Logistic threshold (MTSPS)].

Based on **Figure 3**, the distribution of *A. tsao-ko* is primarily found in certain regions of China, including Yunnan, Guangxi, and Guizhou, aligning with existing documentation in FRPS. Its major distribution zones occur within the latitudinal range of 20° to 30° N and the longitudinal range of 90° to 110° E. The total area identified as suitable for its habitat is a mere 422,933.30 km², representing only 4.41% of China’s entire land mass. High-suitability habitats encompass an area of 137,814.34 km², predominantly situated in Yunnan, southern Guizhou, and western Guangxi. The medium-suitable habitat covers 285,118.96 km², accounting for 2.97% of the country’ s total land area, primarily located in Yunnan, Guizhou, Guangxi, and other regions. The area of low-suitable habitat accounts for 3.40% of the total land area of China, which is 326,702.33 km^2^. In addition to the above areas, there are a small number of distributions in Sichuan, Guangdong, and Chongqing.

#### 3.3.2 Distribution of suitable habitats under past climatic conditions

**Figure 4:**
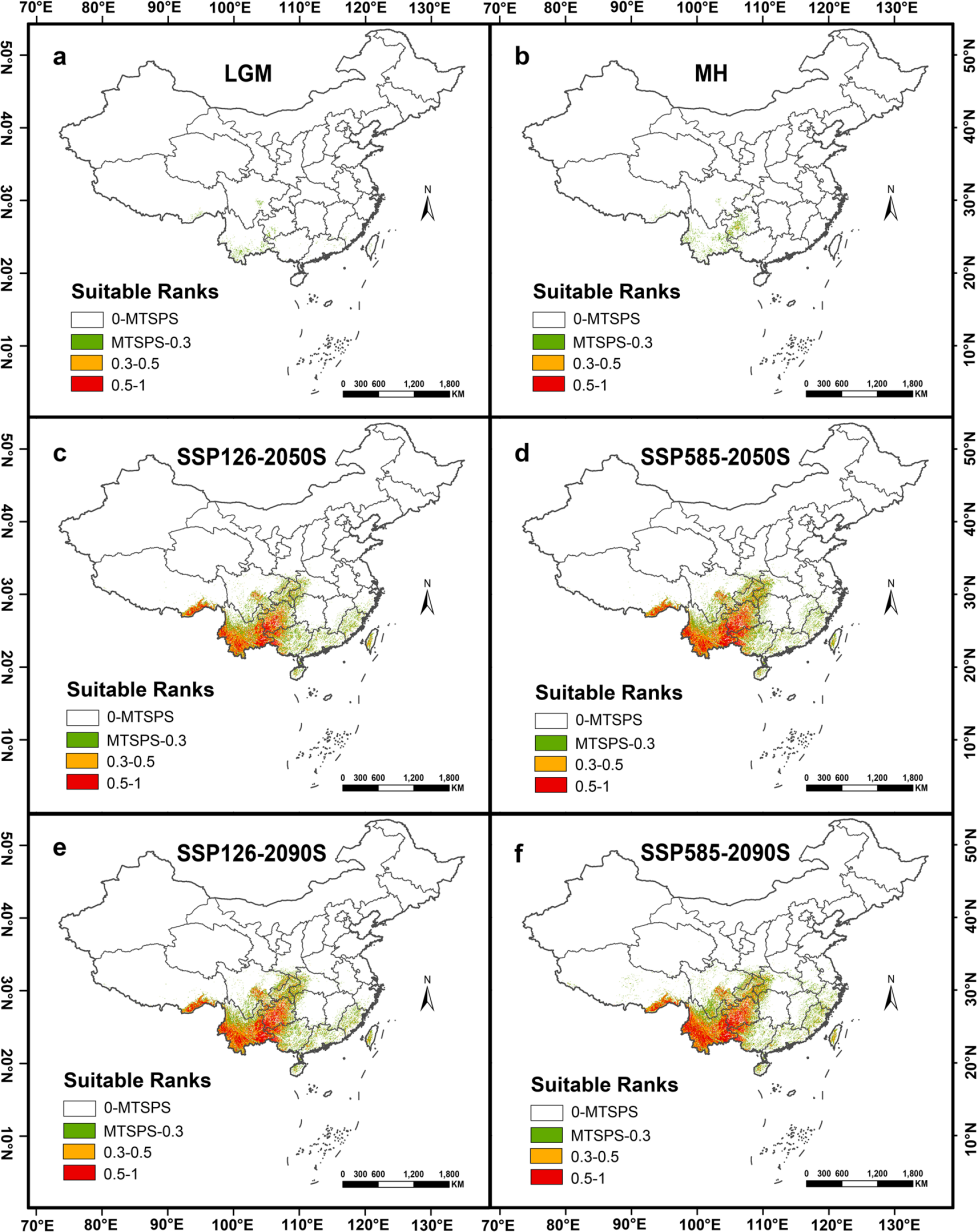
Distribution of suitable habitats for *Amomum tsao-ko* under different climatic conditions. **(a)** Last glacial maximum (LGM). **(b)** Middle holocene (MH). **(c)** Average for 2041–2060 (2050S), SSP126. **(d)** Average for 2041–2060 (2050S), SSP585. **(e)** Average for 2081–2100 (2090S), SSP126. **(f)** Average for 2081–2100 (2090S), SSP585.

**Figures 4a, b** illustrates the distribution of suitable habitat for *A. tsao-ko* under past climate conditions. The range of suitable habitat for *A. tsao-ko* during the Last Glacial Maximum (LGM) is relatively limited compared to current climate conditions (**Figure 4a**), primarily concentrated in a few regions, including Yunnan, Guangxi, and Guizhou. Notably, this habitat is categorized as low-suitability, with a total area of only 748.60 km², which constitutes merely 0.01% of China’s land area. This phenomenon is likely associated with the extreme cold climate prevalent during the LGM, which may have adversely impacted the growth of *A. tsao-ko*, leading to a restricted range of suitable habitat (Huang and Zhang, 2000; Wu et al., 2024).

In contrast, suitable habitat during the Middle Holocene (MH) period (**Figure 4b**) expanded significantly into surrounding areas compared to the LGM period. The total suitable habitat area increased to 3767.13 km², likely due to the notable warming of temperatures during the MH period (Zhan et al., 2022; Yang et al., 2023a). The expansion of suitable habitats for *A. tsao-ko* correlates with rising temperatures, thereby providing enhanced opportunities and space for its growth.

#### 3.3.3 Distribution of suitable habitats under future climate conditions

**Figure 5:**
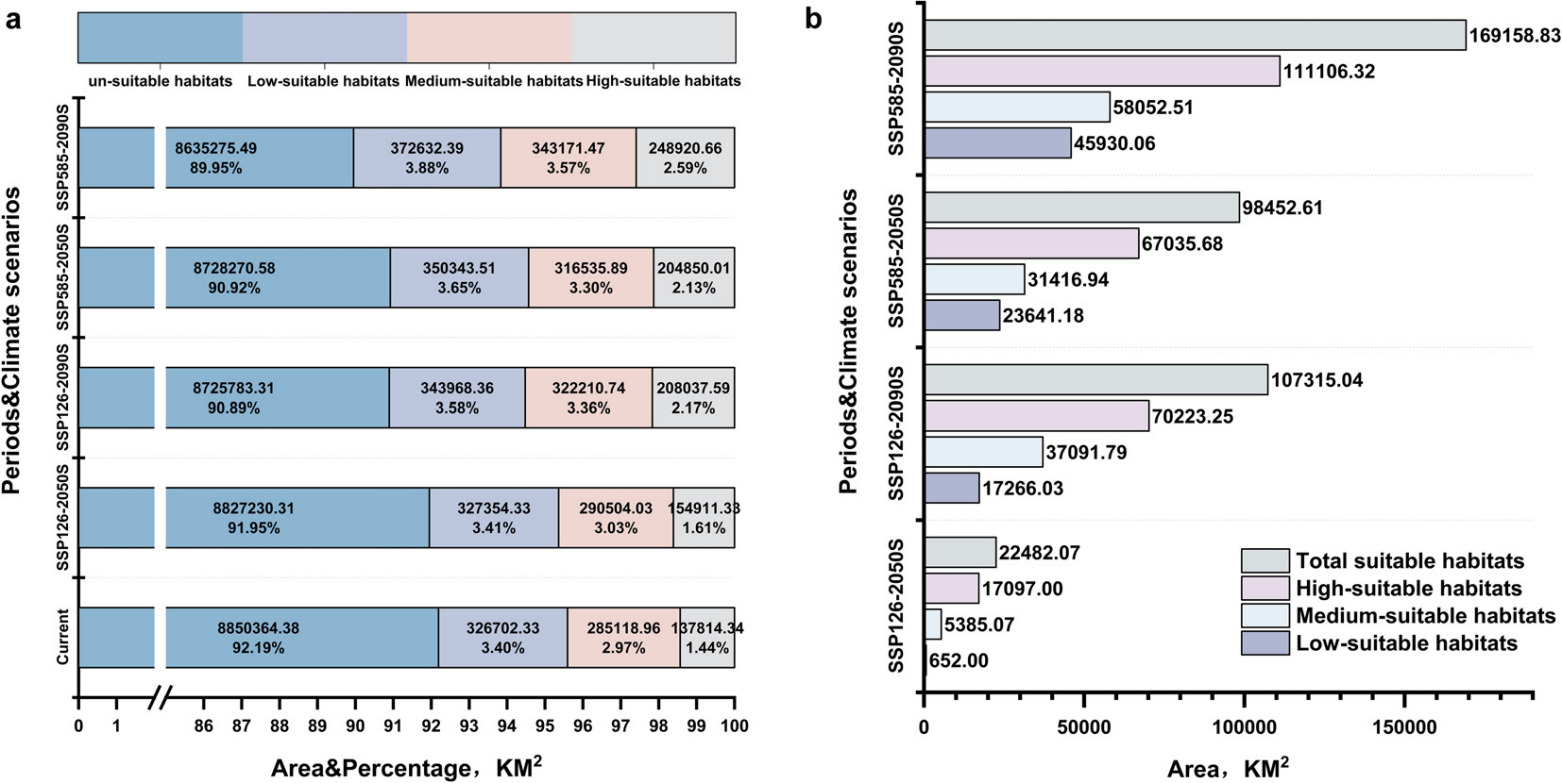
Total suitable habitats: Medium-suitable habitats + High-suitable habitats. **(a)** Area of suitable habitat for *Amomum tsao-ko* under different climatic conditions. **(b)** Future reduction and expansion of the area of suitable habitat for *Amomum tsao-ko* under different climatic conditions.

**Figures 4c–f** clearly demonstrate that the future suitable habitat for *A. tsao-ko* is expanding beyond its original distribution. This indicates that as climate change progresses, *A. tsao-ko* possesses the ability to adapt to new environmental conditions and extend its range for survival. **Figures 5a, b** depict the projected expansion of suitable habitat area for *A. tsao-ko* in the future. According to the SSP126 model scenario, the overall suitable habitat area increases by 22,482.07 km² in SSP126–2050 and by 107,315.04 km² in SSP126-2090. In SSP126-2050, the area of highly suitable habitat rises by 17,097.00 km², while in 2090 under the SSP126 scenario, the highly suitable habitat area increases by 70,223.25 km². In the SSP585 scenario, the medium suitability area expands significantly, with increases of 998,452.61 km² and 169,158.83 km² projected for SSP585–2050 and SSP585-2090, respectively. For both SSP126 and SSP585 future climate conditions, the area of suitable habitat in 2090 surpasses that of 2050. This trend further substantiates the expectation that the habitat area for *A. tsao-ko* will continue to increase in the future.

**Figure 6:**
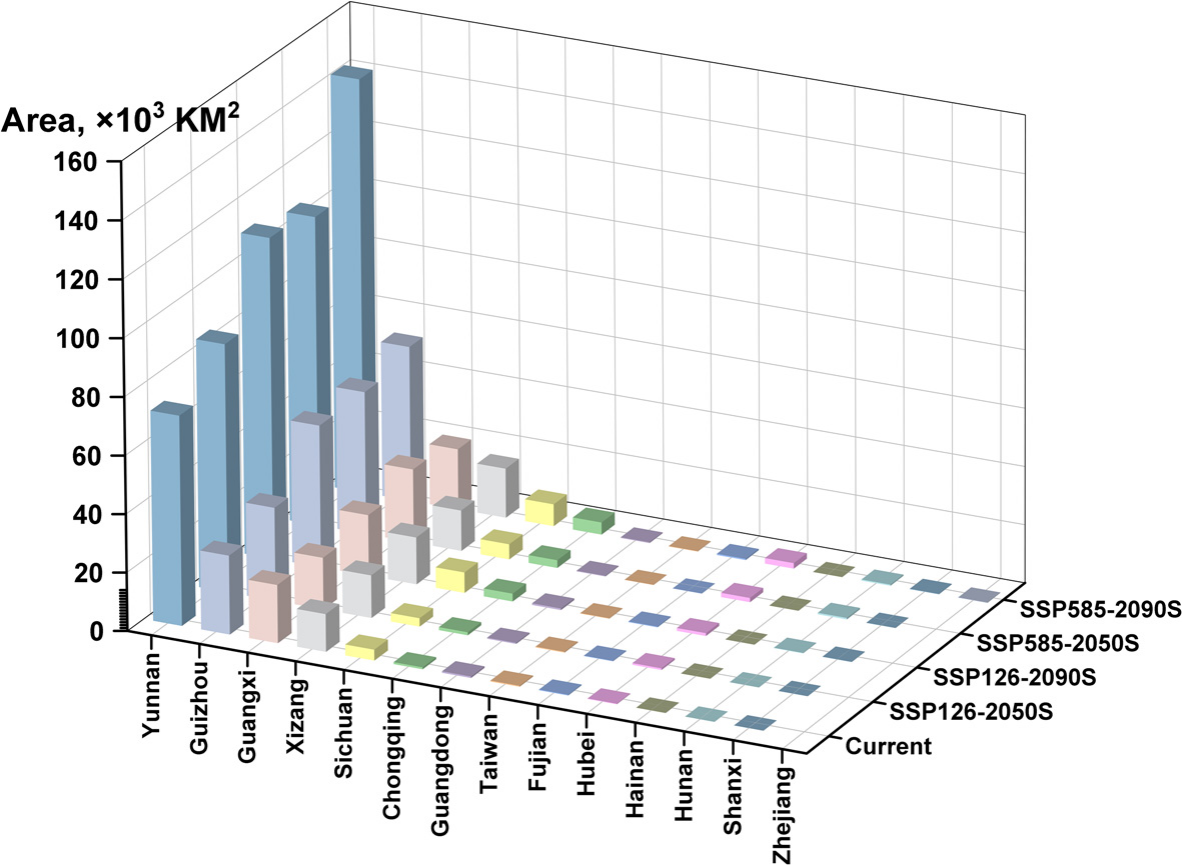
Area of *Amomum tsao-ko* suitable habitat in different provinces in different periods.

**Figure 6** clearly illustrates the spatial distribution of *A. tsao-ko*’s highly suitable habitats across different provinces during various periods. The data for Yunnan, Guizhou, Guangxi, and Xizang provinces were significantly higher compared to other regions, with Yunnan Province consistently exhibiting the highest values across all periods (including the present period). This underscores Yunnan’s absolute dominance as the primary production area for *A. tsao-ko*. Additionally, the suitable areas in each province have shown a continuous increasing trend, suggesting that there is potential for further exploration of its cultivation capabilities in the future.

### 3.4 Volatile oil content of *A. tsao-ko* from different regions

**Figure 7:**
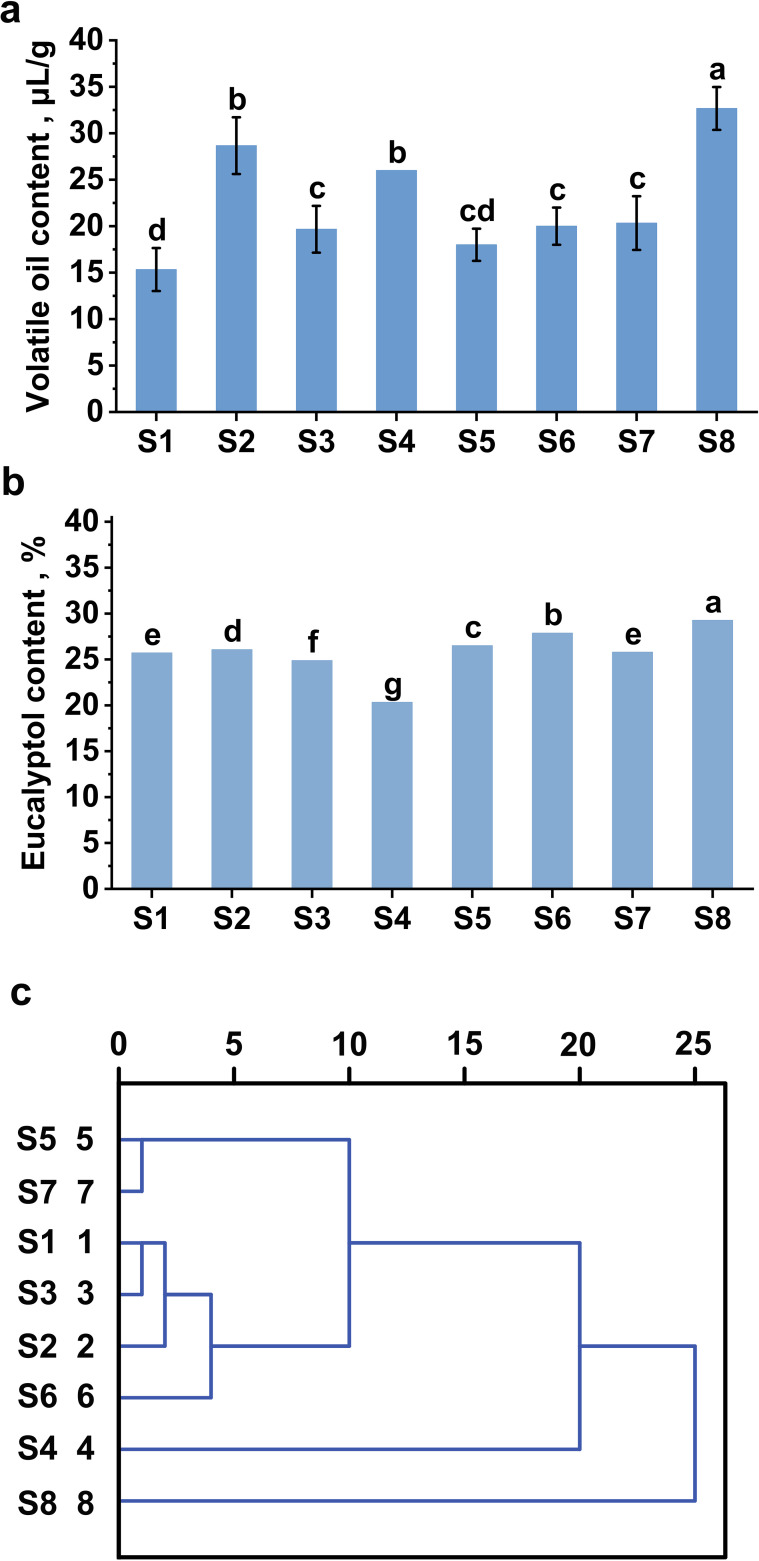
**(a)** Total volatile oil content of *Amomum tsao-ko* from different regions. **(b)** Eucalyptol content of samples from different regions. **(c)** Results of volatile oil cluster analysis of *Amomum tsao-ko* from different regions. Different letters indicate significant differences (p<0.05).

The samples were gathered from Yunnan, a region that is ideal for *A. tsao-ko*, where both geographic and climatic factors establish an optimal ecological environment for its development. As illustrated in **Figure 7a**, the sample obtained from Xishuangbanna (S8) exhibited the highest content of volatile oil at 32.6 μL/g, showing a notable difference in volatile oil levels when compared to samples from other locations. The volatile oil concentrations for samples sourced from Lincang, Tengchong, Wenshan, Maguan, Nujiang, Malipo, and Honghe were recorded as 28.6 μL/g, 26.0 μL/g, 20.4 μL/g, 20.0 μL/g, 19.6 μL/g, 18.0 μL/g, and 15.4 μL/g, respectively. All these measurements surpass the criteria established in the 2020 edition of the Chinese Pharmacopoeia. This observation suggests that the volatile oil content of *A. tsao-ko* typically adheres to high-quality benchmarks within Yunnan’s favorable habitat, thus underscoring the importance of this optimal environment in preserving the quality of *A. tsao-ko*.

### 3.5 Analysis of volatile oil composition of *A. tsao-ko* from different regions

Gas chromatography-mass spectrometry (GC-MS) methods are extensively utilized in various fields including traditional herbal medicine, analyzing pesticide residues, and assessing food flavors (Hailemariam et al., 2018; Sharma et al., 2021; Liang et al., 2024). The assessment of *A. tsao-ko’s* quality can be conducted by examining the variations in its odor-active compounds. In this study, a total of 53 compounds were identified in samples from eight regions (**Table 2**). Specifically, 20, 22, 23, 19, 21, 18, 20, and 23 compounds were identified in *A. tsao-ko* samples from the S1-S8 regions, respectively. Eucalyptol, 2(E)-Decenal, β-Citral, α-Phellandrene, D-Limonene, Terpinen-4-ol, and Nerolidol were present in each sample, though the contents of these components varied significantly. Eucalyptol is its main component. Eucalyptol has rich pharmacological activities, used for antibacterial, anti-inflammatory, antioxidant, anti-tumor, nervous system and promoting osmolar effects (Nikhil et al., 2021; Zhang et al., 2020). Notably, Eucalyptol exhibited the highest terpenoid content across all samples, with a significant difference observed in the Xishuangbanna (S8), which had a content of 29.26%, compared to the other samples (**Figure 7b**). In contrast, the Honghe (S1) and Wenshan (S7) showed no significant difference, with contents of 25.71% and 25.79%, respectively. The Maguan (S6) yielded a content of 27.86%, while the Malipo (S5) had 26.5%. The Lincang (S2) presented a content of 26.07%, and the Nujiang (S3) had 24.88%. The Tengchong (S4) recorded the lowest content at 20.33%. The content of 2(E)-Decenal ranged from 14.18% to 20.02%, with the highest concentration of 20.02% found in the Lincang (S2). No significant difference was noted between the Lincang (S2) and the Tengchong (S4), nor between the Honghe (S1) and Nujiang (S3). Additionally, no significant difference was observed in the D-Limonene content between the S1 and S8.

**Table 2:** GC-MS results of volatile oil composition of *Amomum tsao-ko* from different regions.

No.	Compound	RT (min)	Relative content (%)								Identification
			S1	S2	S3	S4	S5	S6	S7	S8	
1	β-trans-Ocimene	5.019	0.22 ± 0.04	–	–	–	–	–	–	–	GC-MS, RT
2	1S-α-Pinene	5.026	–	0.27 ± 0.03c	0.37 ± 0.03c	0.51 ± 0.02b	0.84 ± 0.03a	–	tr	–	GC-MS, RT
3	1R-α-Pinene	5.026	–	–	–	–	–	0.24 ± 0.02b	–	0.70 ± 0.04a	GC-MS, RT
4	β-Pinene	5.863	0.59 ± 0.01b	0.43 ± 0.01c	–	0.84 ± 0.04a	–	–	–	0.94 ± 0.02a	GC-MS, RT
5	(+)-Sabinene	5.863	–	–	–	–	–	–	0.32 ± 0.02	–	GC-MS, RT
6	N-Methyl-2-pyrydylmethylamine	5.864	–	–	0.50 ± 0.03	–	–	–	–	–	GC-MS, RT
7	Bicyclo[2.1.1]hexane, 1-ethenyl-5,5-dimethyl-	5.870	–	–	–	–	–	0.42 ± 0.01	–	–	GC-MS, RT
8	2-Methylpiperazine	6.345	–	–	–	–	0.33 ± 0.02a	0.46 ± 0.07a	–	–	GC-MS, RT
9	2-(Pentylamino)ethanol	6.339	–	–	0.50 ± 0.04	–	–	–	–	–	GC-MS, RT
10	Octanal	6.339	–	–	–	–	–	–	0.51 ± 0.03	–	GC-MS, RT
11	α-Phellandrene	6.411	3.25 ± 0.02b	1.19 ± 0.03e	0.90 ± 0.11e	4.63 ± 0.19a	0.80 ± 0.01e	2.19 ± 0.02d	tr	2.69 ± 0.02c	GC-MS, RT
12	p-Cymene	6.840	1.56 ± 0.02a	–	–	1.06 ± 0.02b	0.51 ± 0.01c	–	–	–	GC-MS, RT
13	o-Cymene	6.840	–	0.36 ± 0.01c	0.83 ± 0.04b	–	–	1.19 ± 0.03a	0.13 ± 0.02d	–	GC-MS, RT
14	β-Cymene	6.840	–	–	–	–	–	–	–	0.62 ± 0.02	GC-MS, RT
15	D-Limonene	6.932	1.27 ± 0.16a	0.95 ± 0.03a	1.07 ± 0.01a	1.12 ± 0.21a	0.87 ± 0.02a	1.25 ± 0.37a	0.77 ± 0.04a	1.17 ± 0.07a	GC-MS, RT
16	Eucalyptol	7.005	25.71 ± 0.02e	26.07 ± 0.03d	24.88 ± 0.02f	20.33 ± 0.02g	26.50 ± 0.03c	27.86 ± 0.03b	25.79 ± 0.02e	29.26 ± 0.01a	GC-MS, RT, CO
17	2-Octenal, (E)-	7.546	2.12 ± 0.06b	1.68 ± 0.02c	1.61 ± 0.02c	2.11 ± 0.03b	–	1.59 ± 0.08c	2.12 ± 0.03b	2.42 ± 0.01a	GC-MS, RT
18	2-Methylene cyclopentanol	7.546	–	–	–	–	1.19 ± 0.17	–	–	–	GC-MS, RT
19	Terpinen-4-ol	10.336	0.43 ± 0.02b	0.41 ± 0.03bc	0.41 ± 0.02bc	0.74 ± 0.02a	0.31 ± 0.03c	0.38 ± 0.02bc	0.40 ± 0.02bc	0.33 ± 0.02bc	GC-MS, RT
20	α-Terpineol	10.633	1.75 ± 0.03b	2.03 ± 0.04a	–	–	–	1.72 ± 0.02b	–	–	GC-MS, RT
21	Cyclofenchene	10.633	–	–	–	–	1.40 ± 0.04	–	–	–	GC-MS, RT
22	Terpineol	10.633	–	–	1.86 ± 0.06b	1.86 ± 0.01b	–	–	2.01 ± 0.03a	1.53 ± 0.03c	GC-MS, RT
23	β-Citral	11.754	5.41 ± 0.03d	4.05 ± 0.03f	6.99 ± 0.07b	5.02 ± 0.05e	7.13 ± 0.05b	6.73 ± 0.03c	8.59 ± 0.02a	1.29 ± 0.04g	GC-MS, RT
24	2,7-Dimethylocta-2,6-dienol	12.025	0.44 ± 0.02b	–	–	–	–	–	–	5.26 ± 0.07a	GC-MS, RT
25	Farnesol	12.031	–	1.82 ± 0.03	–	–	–	–	–	–	GC-MS, RT
26	Bicyclooctyl	12.032	–	–	0.82 ± 0.01	–	–	–	–	–	GC-MS, RT
27	2(E)-Decenal	12.196	18.04 ± 0.05b	20.02 ± 0.08a	17.70 ± 0.11b	19.75 ± 0.06a	14.97 ± 0.01c	14.18 ± 0.10d	15.07 ± 0.02c	17.82 ± 0.10b	GC-MS, RT
28	Cyclodecanol	12.348	–	–	–	–	–	–	–	0.56 ± 0.01	GC-MS, RT
29	α-Citral	12.408	14.24 ± 0.10c	13.27 ± 0.23d	15.05 ± 0.28bc	–	21.57 ± 0.13a	15.25 ± 0.25b	21.28 ± 0.11a	3.80 ± 0.08e	GC-MS, RT
30	Citral	12.408	–	–	–	17.55 ± 0.14	–	–	–	–	GC-MS, RT
31	3-Phenylpentane	12.863	9.60 ± 0.15a	9.73 ± 0.03a	2.47 ± 0.20c	1.33 ± 0.09d	8.99 ± 0.30a	2.31 ± 0.08c	–	7.84 ± 0.24a	GC-MS, RT
32	1,2-Dimethyl-3-phenyldiaziridine	12.863	–	–	–	–	–	10.48 ± 0.12	–	–	GC-MS, RT
33	2-phenylbutyraldehyde	12.863	–	–	10.11 ± 0.07a	8.69 ± 0.03b	–	–	–	–	GC-MS, RT
34	2-Furfurylfuran	12.863	–	–	–	–	–	–	9.25 ± 0.03	–	GC-MS, RT
35	Hexane, 3,4-diphenyl-	13.041	–	–	–	–	0.46 ± 0.02	–	–	–	GC-MS, RT
36	2,3-Dimethylamphetamine	13.047	–	0.39 ± 0.02a	–	–	–	–	–	0.29 ± 0.03b	GC-MS, RT
37	1-(2,6-Dimethylphenyl)-2-propanamine	13.054	0.45 ± 0.01a	0.17 ± 0.03b	0.46 ± 0.03a	–	0.21 ± 0.02b	–	0.19 ± 0.03b	tr	GC-MS, RT
38	2-Methyl-2-phenylpropanal	13.265	–	1.59 ± 0.11	–	–	–	–	–	–	GC-MS, RT
39	7-[(1E)-1-Propenyl]bicyclo[4.2.0]oct-1-ene	13.265	–	–	–	–	1.10 ± 0.02	–	–	–	GC-MS, RT
40	4-Propylbenzaldehyde	13.272	1.26 ± 0.05	–	–	–	–	–	–	–	GC-MS, RT
41	2,4-Dimethylamphetamine	13.865	–	–	0.14 ± 0.02b	0.24 ± 0.03a	–	–	–	–	GC-MS, RT
42	Indane-4-carboxaldehyde	14.057	0.69 ± 0.03b	1.31 ± 0.12a	1.48 ± 0.04a	–	0.75 ± 0.03b	1.21 ± 0.03a	0.71 ± 0.05b	–	GC-MS, RT
43	trans-2-Phenyl-1-cyclopropanecarbonyl chloride	14.057	–	0.55 ± 0.06	–	–	–	–	–	–	GC-MS, RT
44	2-Ethylindane	14.057	–	–	–	0.37 ± 0.01	–	–	–	–	GC-MS, RT
45	Phthalan	14.347	–	–	–	–	1.54 ± 0.03	–	–	–	GC-MS, RT
46	α-Tolualdehyde	14.347	–	–	1.47 ± 0.02a	–	–	–	1.68 ± 0.10a	1.15 ± 0.02b	GC-MS, RT
47	α-Methylcinnamaldehyde	14.743	0.9 ± 0.04b	–	–	–	0.84 ± 0.04b	–	1.20 ± 0.04a	0.80 ± 0.03b	GC-MS, RT
48	2-Butenal, 3-phenyl-	14.743	–	–	1.90 ± 0.03a	0.98 ± 0.06b	–	–	–	–	GC-MS, RT
49	β-Myrcene	14.868	–	1.31 ± 0.03b	–	–	–	–	–	9.51 ± 0.12a	GC-MS, RT
50	5-decenyl acetate	15.396	–	–	–	–	–	–	–	2.41 ± 0.07	GC-MS, RT
51	(E)-2-Tridecenal	16.616	6.14 ± 0.06a	6.27 ± 0.03a	5.18 ± 0.01b	–	6.25 ± 0.03a	–	–	3.82 ± 0.07c	GC-MS, RT
52	2-Dodecenal	16.616	–	–	–	4.87 ± 0.08a	–	4.48 ± 0.14a	4.60 ± 0.12a	–	GC-MS, RT
53	Nerolidol	18.589	1.33 ± 0.05b	1.62 ± 0.06a	0.57 ± 0.04e	1.27 ± 0.02b	1.00 ± 0.03cd	1.12 ± 0.05bc	1.04 ± 0.06cd	0.87 ± 0.02d	GC-MS, RT
	Terpenoid		61.90	60.05	58.11	59.80	65.78	62.41	65.07	58.94	
	Aliphatics		30.20	31.43	23.10	23.56	28.42	29.44	17.70	33.90	
	Aromatics		3.30	4.01	16.06	9.91	3.36	1.21	13.03	2.33	
	Total		95.40	95.49	97.27	93.27	97.56	93.06	95.80	95.17	

Data are expressed as the mean ± SD. Bars sharing the same small letter within a line did not share significant differences at P<0.05.

GC–MS, gas chromatography–mass spectrometry.; CO, co-injection with authentic compounds; –, not detected; tr (trace), relative content < 0.1%.

### 3.6 System cluster analysis

The eight samples mentioned above were clustered and analyzed (**Figure 7c**). At a Euclidean distance of 5, the cluster analysis categorized the eight samples into four major groups. The Malipo (S5) and the Wenshan (S7) were grouped together. The Honghe (S1), Lincang (S2), Nujiang (S3), and Maguan (S6) formed another group. The Tengchong (S4) was classified in a separate group, as was the Xishuangbanna (S8). When the Euclidean distance was increased to 25, samples S1 through S7 were clustered into one group, while the Xishuangbanna (S8) remained in a distinct group. Notably, S8 exhibited the highest volatile oil content among all samples, along with the highest relative content of eucalyptol, indicating superior quality. These results suggest that there are notable differences in the volatile oil composition of *A. tsao-ko* across different regions, which may be related to geographical location, soil conditions, and the ecological environment of the samples.

## 4 Discussion

### 4.1 Impacts of climate change on *A. tsao-ko*’s habitats

Precipitation and temperature are essential elements that impact the growth and development of plants. They directly influence metabolic processes, such as signaling, self-defense mechanisms, and physiological regulation within plant systems (Pant et al., 2021). In this research, Bio04, Bio17, and Bio12 were identified as the primary environmental factors that affect the potential distribution of *A. tsao-ko*. The findings highlight the crucial influence of temperature and rainfall in defining the suitable habitats for *A. tsao-ko*, particularly noting the significant impact of temperature. These results align with the analysis of suitable habitats for medicinal *Amomum* Roxb. species, where annual temperature fluctuations and the lowest temperature during the coldest month surfaced as vital bioclimatic factors determining their distribution (Zhao, 2022).

Environmental changes can lead to variations in the living space and distribution range of medicinal plants. For instance, under future climate scenarios, the total area of suitable habitat for *Angelica dahurica* is projected to increase, primarily expanding to the middle and high latitudes (Zhang et al., 2024). Similarly, future warming is expected to alter the area of highly suitable habitats for *Gentiana rigescens*, with its suitable habitat shifting to higher altitudes (Shen et al., 2021). Future climate scenarios also indicate a significant trend of expansion in the habitable zone of *Lonicera japonica Flos* in certain regions (Cheng et al., 2024a). Currently, the cultivation of *A. tsao-ko* is predominantly found in the southern Yunnan areas of Honghe, Wenshan, Nujiang, and Xishuangbanna (Wu and Zhu, 1987). These regions experience an average annual temperature exceeding 15°C, along with abundant rainfall and sunlight, creating an ideal ecological environment for the growth of *A. tsao-ko*. Moderate rainfall enhances fruit set and quality (Wang et al., 2024). However, both excessive and insufficient rainfall can negatively impact yield; excessive rainfall may lead to flower rot, whereas insufficient rainfall can desiccate the flowers. The SSP126 and SSP585 scenarios predict increasing trends in future temperatures and precipitation. As *A. tsao-ko* thrives in warm and humid climates, it is likely that ongoing global warming will further increase the area of suitable habitat for this species, facilitating its expansion and spread into middle and high latitudes. This outcome reflects the adaptive adjustments of plants to climate change, which is significant for the future planting strategies and industrial development of *A. tsao-ko*. The change of the suitable area of *A. tsao-ko* was consistent with the expansion direction of *Amomum villosum* Lour and other ginger plants, which expanded to the high latitude area (Ban et al., 2022).

### 4.2 Comparison of volatile oil content and composition of *A. tsao-ko* in different regions

Geographic diversity influences the secondary metabolic pathways in medicinal plants. Environmental factors significantly affect the content of secondary metabolites produced by plants and their biological activity (Wang et al., 2019b; Li et al., 2020). The habitat of *Litsea cubeba* is influenced by factors such as altitude and soil composition. Notably, higher fruit yields correlate with increased volatile oil content (Fan et al., 2023). The volatile oil content and composition of the bark of *Cinnamomum cinnamomi* vary significantly based on species, regions, and growing conditions (Li et al., 2013). In the study of *Zanthoxylum nitidum*, the content of active ingredients in samples from suitable habitat areas met established criteria. Specifically, the concentration of active ingredients was greater in high-suitability habitats compared to low-suitability habitats (Yang et al., 2023a). For instance, among *Z. nitidum* cultivated in Guangdong and Guangxi—regions recognized as highly suitable for growth—the root medicine from Guangdong exhibited a higher concentration of effective medicinal components (Yang et al., 2023b). Moreover, *Coptis* herbs sourced from highly suitable habitats demonstrated the highest total alkaloid content, while medium and averagely suitable habitats yielded lower alkaloid levels. This suggests a potential relationship between alkaloid content and environmental factors (Li et al., 2019). The volatile oil contents of the eight samples analyzed in this study exceeded the standards set forth in the 2020 edition of the Chinese Pharmacopoeia, indicating that the volatile oil content of *A. tsao-ko* from highly suitable habitats generally meets high-quality standards. This finding underscores a positive correlation between volatile oil content and the growing environment.

Environmental changes can alter the chemical composition of medicinal plants and their relative contents (Li et al., 2019; Fan et al., 2023). Both excessive and insufficient water can place the plant in a state of adversity. Moisture levels can impair the secondary metabolic processes within the plant, subsequently affecting the accumulation of bioactive compounds (Yang et al., 2019; Cisse et al., 2024). Temperature influences enzyme activity, thereby affecting the plant’s metabolic rate and developmental growth period (Moore et al., 2021). Additionally, temperature impacts the rate of photosynthesis and secondary metabolic processes; within an appropriate range, higher temperatures can enhance photosynthesis, whereas excessively high temperatures can inhibit it (Chang et al., 2021). Experimental results from GC-MS analysis by Liu et al. indicated that the volatile oil composition of *A. tsao-ko* from Yunnan contained the highest total content of monoterpenes, with eucalyptol being the most abundant component (Liu et al., 2022). Although the volatile oils from Yunnan and Guangxi shared similar major constituents, their contents differed, with Yunnan exhibiting a higher eucalyptol content compared to Guangxi (Sim et al., 2019). The volatile oil species and their relative contents of *A. tsao-ko* varied significantly across different regions, influenced by genetic and geographical factors, with higher altitudes promoting the accumulation of *A. tsao-ko* biomass (Li et al., 2021).

In this study, the volatile oil composition of eight regions samples exhibited variation. All samples demonstrated a high diversity and content of terpenoids, with eucalyptol identified as the most abundant constituent, comprising over 20% of the total. This finding suggests that regions with optimal environmental conditions are more conducive to the synthesis of secondary metabolites in A. tsao-ko, ultimately enhancing the concentration of active ingredients in its volatile oil. Cluster analysis showed that the volatile oil of Xishuangbanna sample S8 was significantly different from that of other regions. This may be due to the complex and diverse local ecosystems, the unique tropical climate (high temperature and high humidity) and the special evolutionary process of plant populations, forming a specific genetic background, affecting their volatile oil synthesis mechanism and producing a unique profile. Xishuangbanna, located in the southern part of Yunnan, China, is characterized by abundant sunshine, a warm climate, minimal temperature fluctuations throughout the year, and significant rainfall. These conditions are favorable for the growth and secondary metabolism of *A. tsao-ko*, thereby influencing the synthesis and accumulation of specific chemical components in its volatile oil. Consequently, this region may serve as a high-quality production area for *A. tsao-ko*, yielding superior medicinal herbs.

### 4.3 Limitations of the study and future prospect

In this study, the distribution characteristics and changing trends of *A. tsao-ko* were preliminarily analyzed. However, due to limitations in sample size and geographic coverage, the results may not fully capture the actual distribution of *A. tsao-ko* or the geographical variations in essential oil components. In future research, it is essential to investigate the impact of climate change on the physiological traits of *A. tsao-ko* by integrating long-term meteorological data with physiological and ecological experiments. This will facilitate an evaluation of how temperature, precipitation, and other environmental factors influence its growth and metabolic processes. Additionally, the interaction mechanisms between environmental factors and essential oil components should be explored. Multi-site sampling combined with metabolomics analysis can be employed to elucidate the relationships between key environmental factors and the accumulation of specific compounds, thereby informing the optimization of cultivation techniques. Furthermore, given the decline in wild resources, efforts should focus on strengthening the collection and evaluation of germplasm resources. This includes incorporating molecular marker-assisted breeding and genetic diversity conservation strategies to advance variety improvement and genetic enhancement.

In the context of resource protection, it is crucial to first accurately delineate the primary distribution areas of production zones (e.g., Yunnan and Guangxi), implement dynamic monitoring systems for changes in plant habitats, enforce strict protection measures, prohibit illegal extraction, and prioritize the preservation of indigenous communities and ecosystems. Secondly, establish relocation sites and appropriate conservation areas for research purposes, collect germplasm materials, develop techniques for artificial breeding and cultivation, and simultaneously plan the construction of demonstration parks for *A. tsao-ko*. These parks should integrate cultural and ecological functions, such as oil extraction and product development, while formulating sustainable harvesting standards, including rotational practices. Furthermore, efforts should focus on scientifically exploring the medicinal properties and economic value of the resources. Additionally, mixed forest management models that combine traditional knowledge with modern technologies are being explored to create synergies between resource conservation and community development.

## 5 Conclusion

In this study, we systematically evaluated the variation in suitable habitats of *A. tsao-ko* under different climatic scenarios using the MaxEnt model and gas chromatography-mass spectrometry (GC-MS) coupling technology, as well as the quality of *A. tsao-ko* across various regions. The following conclusions were drawn: (1) The primary environmental factors influencing the distribution of *A. tsao-ko* are Bio04, Bio12, and Bio17. (2) Currently, the suitable habitats for *A. tsao-ko* are predominantly located in Yunnan, southern Guizhou, and western Guangxi in southwest China. Under future climate scenarios, there is a tendency for the distribution center to shift to higher latitudes. (3) The combined volatile oil content, the relative content of the indicator constituent eucalyptol, and cluster analysis indicate that sample S8 (from the Xishuangbanna production area) can be recognized as a high-quality production source in accordance with the Chinese Pharmacopoeia standard. In conclusion, the integration of the MaxEnt model and gas chromatography-mass spectrometry (GC-MS) provides a scientific foundation for evaluating the suitable habitats and the quality of the volatile oils of *A. tsao-ko*. This approach is of great significance for resource utilization, the planning of cultivation areas, and the quality control of *A. tsao-ko*.

## Data Availability

The datasets presented in this study can be found in online repositories. The names of the repository/repositories and accession number(s) can be found in the article/**Supplementary Material**.
