# Supplementary material for: Assessment of Chinese suitable habitats of Amomum tsao-ko in different climatic conditions
Source: Front Plant Sci. 2025 May 8;16:1561026. doi: 10.3389/fpls.2025.1561026 (PMC12095335; doi:10.3389/fpls.2025.1561026)
Supplement: Supplementary file 1 [file SupplementaryFile1.zip › MaxEnt version 3.4.4.DOCX]

MaxEnt version 3.4.4

Checking header of E:\Amomum_tsaoko\environment\Climate5 Soil6 Topographic3\Current\aspect.asc

Checking header of E:\Amomum_tsaoko\environment\Climate5 Soil6 Topographic3\Current\awc_class.asc

Checking header of E:\Amomum_tsaoko\environment\Climate5 Soil6 Topographic3\Current\bio_12.asc

Checking header of E:\Amomum_tsaoko\environment\Climate5 Soil6 Topographic3\Current\bio_15.asc

Checking header of E:\Amomum_tsaoko\environment\Climate5 Soil6 Topographic3\Current\bio_17.asc

Checking header of E:\Amomum_tsaoko\environment\Climate5 Soil6 Topographic3\Current\bio_4.asc

Checking header of E:\Amomum_tsaoko\environment\Climate5 Soil6 Topographic3\Current\bio_6.asc

Checking header of E:\Amomum_tsaoko\environment\Climate5 Soil6 Topographic3\Current\elev.asc

Checking header of E:\Amomum_tsaoko\environment\Climate5 Soil6 Topographic3\Current\s_caco3.asc

Checking header of E:\Amomum_tsaoko\environment\Climate5 Soil6 Topographic3\Current\s_ph_h2o.asc

Checking header of E:\Amomum_tsaoko\environment\Climate5 Soil6 Topographic3\Current\slope.asc

Checking header of E:\Amomum_tsaoko\environment\Climate5 Soil6 Topographic3\Current\t_clay.asc

Checking header of E:\Amomum_tsaoko\environment\Climate5 Soil6 Topographic3\Current\t_oc.asc

Checking header of E:\Amomum_tsaoko\environment\Climate5 Soil6 Topographic3\Current\t_sand.asc

Reading samples from Sampling_Site.csv

Read samples: max memory 259522560, total allocated 16252928, free 7272536, used 8980392, increment 8980392

Extractor: max memory 259522560, total allocated 16252928, free 6395976, used 9856952, increment 876560

Extracting random background and sample data

429265 points with values for all grids

Adding samples to background in feature space

Command line used:

Command line to repeat: java density.MaxEnt nowarnings noprefixes responsecurves jackknife outputformat=logistic outputdirectory=E:\Amomum_tsaoko\results2 "projectionlayers=E:\Amomum_tsaoko\environment\Climate6 Soil6 Topographic3\LGM, E:\Amomum_tsaoko\environment\Climate6 Soil6 Topographic3\MH, E:\Amomum_tsaoko\environment\Climate6 Soil6 Topographic3\SSP126-2050S, E:\Amomum_tsaoko\environment\Climate6 Soil6 Topographic3\SSP126-2090S, E:\Amomum_tsaoko\environment\Climate6 Soil6 Topographic3\SSP585-2050S, E:\Amomum_tsaoko\environment\Climate6 Soil6 Topographic3\SSP585-2090S" "samplesfile=E:\Amomum_tsaoko\Sampling Site\Sampling_Site.csv" "environmentallayers=E:\Amomum_tsaoko\environment\Climate5 Soil6 Topographic3\Current" randomseed randomtestpoints=25 replicates=10 replicatetype=bootstrap writeplotdata -t awc_class

Species: Amomum_tsaoko_0 Amomum_tsaoko_1 Amomum_tsaoko_2 Amomum_tsaoko_3 Amomum_tsaoko_4 Amomum_tsaoko_5 Amomum_tsaoko_6 Amomum_tsaoko_7 Amomum_tsaoko_8 Amomum_tsaoko_9

Layers: aspect awc_class bio_12 bio_15 bio_17 bio_4 bio_6 elev s_caco3 s_ph_h2o slope t_clay t_oc t_sand

Layertypes: Continuous Categorical Continuous Continuous Continuous Continuous Continuous Continuous Continuous Continuous Continuous Continuous Continuous Continuous

responsecurves: true

jackknife: true

outputformat: logistic

outputdirectory: E:\Amomum_tsaoko\results2

projectionlayers: E:\Amomum_tsaoko\environment\Climate6 Soil6 Topographic3\LGM, E:\Amomum_tsaoko\environment\Climate6 Soil6 Topographic3\MH, E:\Amomum_tsaoko\environment\Climate6 Soil6 Topographic3\SSP126-2050S, E:\Amomum_tsaoko\environment\Climate6 Soil6 Topographic3\SSP126-2090S, E:\Amomum_tsaoko\environment\Climate6 Soil6 Topographic3\SSP585-2050S, E:\Amomum_tsaoko\environment\Climate6 Soil6 Topographic3\SSP585-2090S

samplesfile: E:\Amomum_tsaoko\Sampling Site\Sampling_Site.csv

environmentallayers: E:\Amomum_tsaoko\environment\Climate5 Soil6 Topographic3\Current

randomseed: true

randomtestpoints: 25

replicates: 10

replicatetype: bootstrap

writeplotdata: true

getSamples: max memory 259522560, total allocated 16252928, free 2616824, used 13636104, increment 3779152

Making features

makeFeatures: max memory 259522560, total allocated 23281664, free 6614744, used 16666920, increment 3030816

Amomum_tsaoko_0:

Regularization values: linear/quadratic/product: 0.385, categorical: 0.250, threshold: 1.770, hinge: 0.500

23 samples

Density: max memory 259522560, total allocated 46051328, free 17899776, used 28151552, increment 11484632

linearPredictor: max memory 259522560, total allocated 46051328, free 17819576, used 28231752, increment 80200

Deactivating (awc_class=2.0)

Deactivating (awc_class=3.0)

Deactivating (awc_class=4.0)

Deactivating (awc_class=6.0)

FeaturedSpace: max memory 259522560, total allocated 46051328, free 17819576, used 28231752, increment 0

Sequential: max memory 259522560, total allocated 46051328, free 17804200, used 28247128, increment 15376

Initial loss: 9.212637731024866

Initial test loss: 9.212637731024866

Time since start: 7.199

500: time = 6.755000 loss = 6.674502 testLoss 6.180582

Resulting gain: 2.5381357430964178

Projecting...

Writing file E:\Amomum_tsaoko\results2\Amomum_tsaoko_0.asc

Time since start: 8.777

Writing E:\Amomum_tsaoko\results2\plots\Amomum_tsaoko_0.png

Projecting...

Writing file E:\Amomum_tsaoko\results2\Amomum_tsaoko_0_LGM.asc

Writing file E:\Amomum_tsaoko\results2\Amomum_tsaoko_0_LGM_clamping.asc

Time since start: 11.067

Writing E:\Amomum_tsaoko\results2\plots\Amomum_tsaoko_0_LGM.png

Writing E:\Amomum_tsaoko\results2\plots\Amomum_tsaoko_0_LGM_clamping.png

Time since start: 12.253

Writing file E:\Amomum_tsaoko\results2\Amomum_tsaoko_0_LGM_novel.asc

Writing file E:\Amomum_tsaoko\results2\Amomum_tsaoko_0_LGM_novel_limiting.asc

Time since start: 14.24

Projecting...

Writing file E:\Amomum_tsaoko\results2\Amomum_tsaoko_0_MH.asc

Writing file E:\Amomum_tsaoko\results2\Amomum_tsaoko_0_MH_clamping.asc

Time since start: 16.706

Writing E:\Amomum_tsaoko\results2\plots\Amomum_tsaoko_0_MH.png

Writing E:\Amomum_tsaoko\results2\plots\Amomum_tsaoko_0_MH_clamping.png

Time since start: 17.843

Writing file E:\Amomum_tsaoko\results2\Amomum_tsaoko_0_MH_novel.asc

Writing file E:\Amomum_tsaoko\results2\Amomum_tsaoko_0_MH_novel_limiting.asc

Time since start: 19.686

Projecting...

Writing file E:\Amomum_tsaoko\results2\Amomum_tsaoko_0_SSP126-2050S.asc

Writing file E:\Amomum_tsaoko\results2\Amomum_tsaoko_0_SSP126-2050S_clamping.asc

Time since start: 21.078

Writing E:\Amomum_tsaoko\results2\plots\Amomum_tsaoko_0_SSP126-2050S.png

Writing E:\Amomum_tsaoko\results2\plots\Amomum_tsaoko_0_SSP126-2050S_clamping.png

Time since start: 22.131

Writing file E:\Amomum_tsaoko\results2\Amomum_tsaoko_0_SSP126-2050S_novel.asc

Writing file E:\Amomum_tsaoko\results2\Amomum_tsaoko_0_SSP126-2050S_novel_limiting.asc

Time since start: 23.996

Projecting...

Writing file E:\Amomum_tsaoko\results2\Amomum_tsaoko_0_SSP126-2090S.asc

Writing file E:\Amomum_tsaoko\results2\Amomum_tsaoko_0_SSP126-2090S_clamping.asc

Time since start: 25.394

Writing E:\Amomum_tsaoko\results2\plots\Amomum_tsaoko_0_SSP126-2090S.png

Writing E:\Amomum_tsaoko\results2\plots\Amomum_tsaoko_0_SSP126-2090S_clamping.png

Time since start: 26.541

Writing file E:\Amomum_tsaoko\results2\Amomum_tsaoko_0_SSP126-2090S_novel.asc

Writing file E:\Amomum_tsaoko\results2\Amomum_tsaoko_0_SSP126-2090S_novel_limiting.asc

Time since start: 31.176

Projecting...

Writing file E:\Amomum_tsaoko\results2\Amomum_tsaoko_0_SSP585-2050S.asc

Writing file E:\Amomum_tsaoko\results2\Amomum_tsaoko_0_SSP585-2050S_clamping.asc

Time since start: 34.858

Writing E:\Amomum_tsaoko\results2\plots\Amomum_tsaoko_0_SSP585-2050S.png

Time since start: 36.218

Writing E:\Amomum_tsaoko\results2\plots\Amomum_tsaoko_0_SSP585-2050S_clamping.png

Time since start: 37.457

Writing file E:\Amomum_tsaoko\results2\Amomum_tsaoko_0_SSP585-2050S_novel.asc

Writing file E:\Amomum_tsaoko\results2\Amomum_tsaoko_0_SSP585-2050S_novel_limiting.asc

Time since start: 42.198

Projecting...

Writing file E:\Amomum_tsaoko\results2\Amomum_tsaoko_0_SSP585-2090S.asc

Writing file E:\Amomum_tsaoko\results2\Amomum_tsaoko_0_SSP585-2090S_clamping.asc

Time since start: 45.912

Writing E:\Amomum_tsaoko\results2\plots\Amomum_tsaoko_0_SSP585-2090S.png

Time since start: 47.287

Writing E:\Amomum_tsaoko\results2\plots\Amomum_tsaoko_0_SSP585-2090S_clamping.png

Time since start: 48.732

Writing file E:\Amomum_tsaoko\results2\Amomum_tsaoko_0_SSP585-2090S_novel.asc

Writing file E:\Amomum_tsaoko\results2\Amomum_tsaoko_0_SSP585-2090S_novel_limiting.asc

Time since start: 53.223

Amomum_tsaoko_0 response curves

Response curve: only aspect

Making features

makeFeatures: max memory 259522560, total allocated 139407360, free 65044608, used 74362752, increment 46115624

Amomum_tsaoko_0 aspect:

Regularization values: linear/quadratic/product: 0.385, categorical: 0.250, threshold: 1.770, hinge: 0.500

23 samples

Time since start: 54.225

Density: max memory 259522560, total allocated 139407360, free 61909424, used 77497936, increment 3135184

linearPredictor: max memory 259522560, total allocated 139407360, free 61909424, used 77497936, increment 0

FeaturedSpace: max memory 259522560, total allocated 139407360, free 61909424, used 77497936, increment 0

Sequential: max memory 259522560, total allocated 139407360, free 61909424, used 77497936, increment 0

Initial loss: 9.212637731024866

60: time = 0.870000 loss = 9.104881

Resulting gain: 0.1077569500609723

Amomum_tsaoko_0 response curves

Response curve: only awc_class

Making features

makeFeatures: max memory 259522560, total allocated 139407360, free 67768608, used 71638752, increment -5859184

Amomum_tsaoko_0 awc_class:

Regularization values: linear/quadratic/product: 0.385, categorical: 0.250, threshold: 1.770, hinge: 0.500

23 samples

Density: max memory 259522560, total allocated 139407360, free 67758008, used 71649352, increment 10600

linearPredictor: max memory 259522560, total allocated 139407360, free 67758008, used 71649352, increment 0

Deactivating (awc_class=2.0)

Deactivating (awc_class=3.0)

Deactivating (awc_class=4.0)

Deactivating (awc_class=6.0)

FeaturedSpace: max memory 259522560, total allocated 139407360, free 67758008, used 71649352, increment 0

Sequential: max memory 259522560, total allocated 139407360, free 67758008, used 71649352, increment 0

Initial loss: 9.212637731024866

Time since start: 55.398

100: time = 0.187000 loss = 9.168644

Resulting gain: 0.04399404918563121

Amomum_tsaoko_0 response curves

Response curve: only bio_12

Making features

makeFeatures: max memory 259522560, total allocated 139407360, free 63089728, used 76317632, increment 4668280

Amomum_tsaoko_0 bio_12:

Regularization values: linear/quadratic/product: 0.385, categorical: 0.250, threshold: 1.770, hinge: 0.500

23 samples

Density: max memory 259522560, total allocated 139407360, free 61537792, used 77869568, increment 1551936

linearPredictor: max memory 259522560, total allocated 139407360, free 61537792, used 77869568, increment 0

FeaturedSpace: max memory 259522560, total allocated 139407360, free 61537792, used 77869568, increment 0

Sequential: max memory 259522560, total allocated 139407360, free 61537792, used 77869568, increment 0

Initial loss: 9.212637731024866

120: time = 0.692000 loss = 7.951014

Resulting gain: 1.2616234322166946

Amomum_tsaoko_0 response curves

Response curve: only bio_15

Making features

makeFeatures: max memory 259522560, total allocated 139407360, free 49989488, used 89417872, increment 11548304

Amomum_tsaoko_0 bio_15:

Regularization values: linear/quadratic/product: 0.385, categorical: 0.250, threshold: 1.770, hinge: 0.500

23 samples

Density: max memory 259522560, total allocated 139407360, free 47192072, used 92215288, increment 2797416

linearPredictor: max memory 259522560, total allocated 139407360, free 47192072, used 92215288, increment 0

FeaturedSpace: max memory 259522560, total allocated 139407360, free 47192072, used 92215288, increment 0

Sequential: max memory 259522560, total allocated 139407360, free 47192072, used 92215288, increment 0

Initial loss: 9.212637731024866

Time since start: 57.159

80: time = 0.885000 loss = 8.853958

Resulting gain: 0.35867982507931373

Amomum_tsaoko_0 response curves

Response curve: only bio_17

Making features

makeFeatures: max memory 259522560, total allocated 139407360, free 40190760, used 99216600, increment 7001312

Amomum_tsaoko_0 bio_17:

Regularization values: linear/quadratic/product: 0.385, categorical: 0.250, threshold: 1.770, hinge: 0.500

23 samples

Density: max memory 259522560, total allocated 139407360, free 39288608, used 100118752, increment 902152

linearPredictor: max memory 259522560, total allocated 139407360, free 39288608, used 100118752, increment 0

FeaturedSpace: max memory 259522560, total allocated 139407360, free 39288608, used 100118752, increment 0

Sequential: max memory 259522560, total allocated 139407360, free 39288608, used 100118752, increment 0

Initial loss: 9.212637731024866

Time since start: 58.333

300: time = 1.089000 loss = 7.800135

Resulting gain: 1.4125031884950774

Amomum_tsaoko_0 response curves

Response curve: only bio_4

Making features

makeFeatures: max memory 259522560, total allocated 139407360, free 57977368, used 81429992, increment -18688760

Amomum_tsaoko_0 bio_4:

Regularization values: linear/quadratic/product: 0.385, categorical: 0.250, threshold: 1.770, hinge: 0.500

23 samples

Density: max memory 259522560, total allocated 139407360, free 54773128, used 84634232, increment 3204240

linearPredictor: max memory 259522560, total allocated 139407360, free 54692928, used 84714432, increment 80200

FeaturedSpace: max memory 259522560, total allocated 139407360, free 54692928, used 84714432, increment 0

Sequential: max memory 259522560, total allocated 139407360, free 54692928, used 84714432, increment 0

Initial loss: 9.212637731024866

Time since start: 59.849

120: time = 1.432000 loss = 8.024836

Resulting gain: 1.187801383648246

Amomum_tsaoko_0 response curves

Response curve: only bio_6

Making features

makeFeatures: max memory 259522560, total allocated 139407360, free 36963104, used 102444256, increment 17729824

Amomum_tsaoko_0 bio_6:

Regularization values: linear/quadratic/product: 0.385, categorical: 0.250, threshold: 1.770, hinge: 0.500

23 samples

Density: max memory 259522560, total allocated 139407360, free 34534184, used 104873176, increment 2428920

linearPredictor: max memory 259522560, total allocated 139407360, free 34534184, used 104873176, increment 0

FeaturedSpace: max memory 259522560, total allocated 139407360, free 34534184, used 104873176, increment 0

Sequential: max memory 259522560, total allocated 139407360, free 34534184, used 104873176, increment 0

Initial loss: 9.212637731024866

Time since start: 61.288

180: time = 1.327000 loss = 7.816595

Resulting gain: 1.3960428392438722

Amomum_tsaoko_0 response curves

Response curve: only elev

Making features

makeFeatures: max memory 259522560, total allocated 139407360, free 38095136, used 101312224, increment -3560952

Amomum_tsaoko_0 elev:

Regularization values: linear/quadratic/product: 0.385, categorical: 0.250, threshold: 1.770, hinge: 0.500

23 samples

Density: max memory 259522560, total allocated 139407360, free 36545776, used 102861584, increment 1549360

linearPredictor: max memory 259522560, total allocated 139407360, free 36545776, used 102861584, increment 0

FeaturedSpace: max memory 259522560, total allocated 139407360, free 36545776, used 102861584, increment 0

Sequential: max memory 259522560, total allocated 139407360, free 36545776, used 102861584, increment 0

Initial loss: 9.212637731024866

Time since start: 62.343

160: time = 0.967000 loss = 8.855730

Resulting gain: 0.35690723992554396

Amomum_tsaoko_0 response curves

Response curve: only s_caco3

Making features

makeFeatures: max memory 259522560, total allocated 139407360, free 48904496, used 90502864, increment -12358720

Amomum_tsaoko_0 s_caco3:

Regularization values: linear/quadratic/product: 0.385, categorical: 0.250, threshold: 1.770, hinge: 0.500

23 samples

Density: max memory 259522560, total allocated 139407360, free 48126480, used 91280880, increment 778016

linearPredictor: max memory 259522560, total allocated 139407360, free 48126480, used 91280880, increment 0

FeaturedSpace: max memory 259522560, total allocated 139407360, free 48126480, used 91280880, increment 0

Sequential: max memory 259522560, total allocated 139407360, free 48126480, used 91280880, increment 0

Initial loss: 9.212637731024866

140: time = 0.747000 loss = 8.672303

Resulting gain: 0.5403351267727263

Amomum_tsaoko_0 response curves

Response curve: only s_ph_h2o

Making features

makeFeatures: max memory 259522560, total allocated 139407360, free 41434000, used 97973360, increment 6692480

Amomum_tsaoko_0 s_ph_h2o:

Regularization values: linear/quadratic/product: 0.385, categorical: 0.250, threshold: 1.770, hinge: 0.500

23 samples

Density: max memory 259522560, total allocated 139407360, free 39724752, used 99682608, increment 1709248

linearPredictor: max memory 259522560, total allocated 139407360, free 39724752, used 99682608, increment 0

FeaturedSpace: max memory 259522560, total allocated 139407360, free 39724752, used 99682608, increment 0

Sequential: max memory 259522560, total allocated 139407360, free 39724752, used 99682608, increment 0

Initial loss: 9.212637731024866

Time since start: 64.397

340: time = 1.102000 loss = 8.345314

Resulting gain: 0.8673242286911176

Amomum_tsaoko_0 response curves

Response curve: only slope

Making features

makeFeatures: max memory 259522560, total allocated 139407360, free 67959440, used 71447920, increment -28234688

Amomum_tsaoko_0 slope:

Regularization values: linear/quadratic/product: 0.385, categorical: 0.250, threshold: 1.770, hinge: 0.500

23 samples

Density: max memory 259522560, total allocated 139407360, free 65662336, used 73745024, increment 2297104

linearPredictor: max memory 259522560, total allocated 139407360, free 65662336, used 73745024, increment 0

FeaturedSpace: max memory 259522560, total allocated 139407360, free 65662336, used 73745024, increment 0

Sequential: max memory 259522560, total allocated 139407360, free 65662336, used 73745024, increment 0

Initial loss: 9.212637731024866

Time since start: 66.723

240: time = 2.229000 loss = 9.055976

Resulting gain: 0.15666198872488657

Amomum_tsaoko_0 response curves

Response curve: only t_clay

Making features

makeFeatures: max memory 259522560, total allocated 139407360, free 39667312, used 99740048, increment 25995024

Amomum_tsaoko_0 t_clay:

Regularization values: linear/quadratic/product: 0.385, categorical: 0.250, threshold: 1.770, hinge: 0.500

23 samples

Density: max memory 259522560, total allocated 139407360, free 37959160, used 101448200, increment 1708152

linearPredictor: max memory 259522560, total allocated 139407360, free 37959160, used 101448200, increment 0

FeaturedSpace: max memory 259522560, total allocated 139407360, free 37959160, used 101448200, increment 0

Sequential: max memory 259522560, total allocated 139407360, free 37959160, used 101448200, increment 0

Initial loss: 9.212637731024866

120: time = 0.335000 loss = 8.761769

Resulting gain: 0.45086865370910445

Amomum_tsaoko_0 response curves

Response curve: only t_oc

Making features

makeFeatures: max memory 259522560, total allocated 139407360, free 71011240, used 68396120, increment -33052080

Amomum_tsaoko_0 t_oc:

Regularization values: linear/quadratic/product: 0.385, categorical: 0.250, threshold: 1.770, hinge: 0.500

23 samples

Density: max memory 259522560, total allocated 139407360, free 70224024, used 69183336, increment 787216

linearPredictor: max memory 259522560, total allocated 139407360, free 70224024, used 69183336, increment 0

FeaturedSpace: max memory 259522560, total allocated 139407360, free 70224024, used 69183336, increment 0

Sequential: max memory 259522560, total allocated 139407360, free 70224024, used 69183336, increment 0

Initial loss: 9.212637731024866

Time since start: 67.734

120: time = 0.484000 loss = 8.640603

Resulting gain: 0.5720350885666239

Amomum_tsaoko_0 response curves

Response curve: only t_sand

Making features

makeFeatures: max memory 259522560, total allocated 139407360, free 62305680, used 77101680, increment 7918344

Amomum_tsaoko_0 t_sand:

Regularization values: linear/quadratic/product: 0.385, categorical: 0.250, threshold: 1.770, hinge: 0.500

23 samples

Density: max memory 259522560, total allocated 139407360, free 61526880, used 77880480, increment 778800

linearPredictor: max memory 259522560, total allocated 139407360, free 61526880, used 77880480, increment 0

FeaturedSpace: max memory 259522560, total allocated 139407360, free 61526880, used 77880480, increment 0

Sequential: max memory 259522560, total allocated 139407360, free 61526880, used 77880480, increment 0

Initial loss: 9.212637731024866

160: time = 0.696000 loss = 9.094963

Resulting gain: 0.11767437834753558

Amomum_tsaoko_0 response curves

Time since start: 69.535

Jackknife: leave aspect out

Making features

makeFeatures: max memory 259522560, total allocated 139407360, free 44385360, used 95022000, increment 17141520

Amomum_tsaoko_0 aspect:

Regularization values: linear/quadratic/product: 0.385, categorical: 0.250, threshold: 1.770, hinge: 0.500

23 samples

Density: max memory 259522560, total allocated 139407360, free 51928368, used 87478992, increment -7543008

linearPredictor: max memory 259522560, total allocated 139407360, free 51928368, used 87478992, increment 0

Deactivating (awc_class=2.0)

Deactivating (awc_class=3.0)

Deactivating (awc_class=4.0)

Deactivating (awc_class=6.0)

FeaturedSpace: max memory 259522560, total allocated 139407360, free 51928368, used 87478992, increment 0

Sequential: max memory 259522560, total allocated 139407360, free 51928368, used 87478992, increment 0

Initial loss: 9.212637731024866

Initial test loss: 9.212637731024866

Time since start: 86.922

500: time = 17.112000 loss = 6.760212 testLoss 6.303319

Jackknife: leave awc_class out

Making features

makeFeatures: max memory 259522560, total allocated 139407360, free 49822456, used 89584904, increment 2105912

Amomum_tsaoko_0 awc_class:

Regularization values: linear/quadratic/product: 0.385, categorical: 0.250, threshold: 1.770, hinge: 0.500

23 samples

Density: max memory 259522560, total allocated 139407360, free 28707904, used 110699456, increment 21114552

linearPredictor: max memory 259522560, total allocated 139407360, free 28627704, used 110779656, increment 80200

FeaturedSpace: max memory 259522560, total allocated 139407360, free 28627704, used 110779656, increment 0

Sequential: max memory 259522560, total allocated 139407360, free 28627704, used 110779656, increment 0

Initial loss: 9.212637731024866

Initial test loss: 9.212637731024866

Time since start: 106.723

500: time = 19.445000 loss = 6.674391 testLoss 6.181770

Jackknife: leave bio_12 out

Making features

makeFeatures: max memory 259522560, total allocated 139407360, free 78763400, used 60643960, increment -50135696

Amomum_tsaoko_0 bio_12:

Regularization values: linear/quadratic/product: 0.385, categorical: 0.250, threshold: 1.770, hinge: 0.500

23 samples

Density: max memory 259522560, total allocated 139407360, free 58645728, used 80761632, increment 20117672

linearPredictor: max memory 259522560, total allocated 139407360, free 58645728, used 80761632, increment 0

Deactivating (awc_class=2.0)

Deactivating (awc_class=3.0)

Deactivating (awc_class=4.0)

Deactivating (awc_class=6.0)

FeaturedSpace: max memory 259522560, total allocated 139407360, free 58645728, used 80761632, increment 0

Sequential: max memory 259522560, total allocated 139407360, free 58645728, used 80761632, increment 0

Initial loss: 9.212637731024866

Initial test loss: 9.212637731024866

Time since start: 125.123

500: time = 18.105000 loss = 6.727293 testLoss 6.332967

Jackknife: leave bio_15 out

Making features

makeFeatures: max memory 259522560, total allocated 139407360, free 43799168, used 95608192, increment 14846560

Amomum_tsaoko_0 bio_15:

Regularization values: linear/quadratic/product: 0.385, categorical: 0.250, threshold: 1.770, hinge: 0.500

23 samples

Density: max memory 259522560, total allocated 139407360, free 53965992, used 85441368, increment -10166824

linearPredictor: max memory 259522560, total allocated 139407360, free 53965992, used 85441368, increment 0

Deactivating (awc_class=2.0)

Deactivating (awc_class=3.0)

Deactivating (awc_class=4.0)

Deactivating (awc_class=6.0)

FeaturedSpace: max memory 259522560, total allocated 139407360, free 53965992, used 85441368, increment 0

Sequential: max memory 259522560, total allocated 139407360, free 53965992, used 85441368, increment 0

Initial loss: 9.212637731024866

Initial test loss: 9.212637731024866

Time since start: 141.029

500: time = 15.618000 loss = 6.674517 testLoss 6.181893

Jackknife: leave bio_17 out

Making features

makeFeatures: max memory 259522560, total allocated 139407360, free 49830208, used 89577152, increment 4135784

Amomum_tsaoko_0 bio_17:

Regularization values: linear/quadratic/product: 0.385, categorical: 0.250, threshold: 1.770, hinge: 0.500

23 samples

Density: max memory 259522560, total allocated 139407360, free 29100128, used 110307232, increment 20730080

linearPredictor: max memory 259522560, total allocated 139407360, free 29100128, used 110307232, increment 0

Deactivating (awc_class=2.0)

Deactivating (awc_class=3.0)

Deactivating (awc_class=4.0)

Deactivating (awc_class=6.0)

FeaturedSpace: max memory 259522560, total allocated 139407360, free 29100128, used 110307232, increment 0

Sequential: max memory 259522560, total allocated 139407360, free 29100128, used 110307232, increment 0

Initial loss: 9.212637731024866

Initial test loss: 9.212637731024866

Time since start: 160.1

500: time = 18.783000 loss = 6.718997 testLoss 6.291156

Jackknife: leave bio_4 out

Making features

makeFeatures: max memory 259522560, total allocated 139407360, free 40895400, used 98511960, increment -11795272

Amomum_tsaoko_0 bio_4:

Regularization values: linear/quadratic/product: 0.385, categorical: 0.250, threshold: 1.770, hinge: 0.500

23 samples

Density: max memory 259522560, total allocated 139407360, free 22482152, used 116925208, increment 18413248

linearPredictor: max memory 259522560, total allocated 139407360, free 22482152, used 116925208, increment 0

Deactivating (awc_class=2.0)

Deactivating (awc_class=3.0)

Deactivating (awc_class=4.0)

Deactivating (awc_class=6.0)

FeaturedSpace: max memory 259522560, total allocated 139407360, free 22482152, used 116925208, increment 0

Sequential: max memory 259522560, total allocated 139407360, free 22482152, used 116925208, increment 0

Initial loss: 9.212637731024866

Initial test loss: 9.212637731024866

Time since start: 175.288

480: time = 14.884000 loss = 6.691834 testLoss 6.375534

Jackknife: leave bio_6 out

Making features

makeFeatures: max memory 259522560, total allocated 139407360, free 71657136, used 67750224, increment -49174984

Amomum_tsaoko_0 bio_6:

Regularization values: linear/quadratic/product: 0.385, categorical: 0.250, threshold: 1.770, hinge: 0.500

23 samples

Density: max memory 259522560, total allocated 139407360, free 77152032, used 62255328, increment -5494896

linearPredictor: max memory 259522560, total allocated 139407360, free 77152032, used 62255328, increment 0

Deactivating (awc_class=2.0)

Deactivating (awc_class=3.0)

Deactivating (awc_class=4.0)

Deactivating (awc_class=6.0)

FeaturedSpace: max memory 259522560, total allocated 139407360, free 77152032, used 62255328, increment 0

Sequential: max memory 259522560, total allocated 139407360, free 77152032, used 62255328, increment 0

Initial loss: 9.212637731024866

Initial test loss: 9.212637731024866

Time since start: 192.57

500: time = 16.985000 loss = 6.716649 testLoss 6.254251

Jackknife: leave elev out

Making features

makeFeatures: max memory 259522560, total allocated 139407360, free 75668104, used 63739256, increment 1483928

Amomum_tsaoko_0 elev:

Regularization values: linear/quadratic/product: 0.385, categorical: 0.250, threshold: 1.770, hinge: 0.500

23 samples

Density: max memory 259522560, total allocated 139407360, free 55552816, used 83854544, increment 20115288

linearPredictor: max memory 259522560, total allocated 139407360, free 55552816, used 83854544, increment 0

Deactivating (awc_class=2.0)

Deactivating (awc_class=3.0)

Deactivating (awc_class=4.0)

Deactivating (awc_class=6.0)

FeaturedSpace: max memory 259522560, total allocated 139407360, free 55552816, used 83854544, increment 0

Sequential: max memory 259522560, total allocated 139407360, free 55552816, used 83854544, increment 0

Initial loss: 9.212637731024866

Initial test loss: 9.212637731024866

Time since start: 211.216

500: time = 18.349000 loss = 6.912737 testLoss 6.383290

Jackknife: leave s_caco3 out

Making features

makeFeatures: max memory 259522560, total allocated 139407360, free 53532112, used 85875248, increment 2020704

Amomum_tsaoko_0 s_caco3:

Regularization values: linear/quadratic/product: 0.385, categorical: 0.250, threshold: 1.770, hinge: 0.500

23 samples

Density: max memory 259522560, total allocated 139407360, free 32495016, used 106912344, increment 21037096

linearPredictor: max memory 259522560, total allocated 139407360, free 32495016, used 106912344, increment 0

Deactivating (awc_class=2.0)

Deactivating (awc_class=3.0)

Deactivating (awc_class=4.0)

Deactivating (awc_class=6.0)

FeaturedSpace: max memory 259522560, total allocated 139407360, free 32495016, used 106912344, increment 0

Sequential: max memory 259522560, total allocated 139407360, free 32495016, used 106912344, increment 0

Initial loss: 9.212637731024866

Initial test loss: 9.212637731024866

Time since start: 230.258

500: time = 18.745000 loss = 6.710734 testLoss 6.229057

Jackknife: leave s_ph_h2o out

Making features

makeFeatures: max memory 259522560, total allocated 139407360, free 35825440, used 103581920, increment -3330424

Amomum_tsaoko_0 s_ph_h2o:

Regularization values: linear/quadratic/product: 0.385, categorical: 0.250, threshold: 1.770, hinge: 0.500

23 samples

Density: max memory 259522560, total allocated 139407360, free 14912416, used 124494944, increment 20913024

linearPredictor: max memory 259522560, total allocated 139407360, free 14912416, used 124494944, increment 0

Deactivating (awc_class=2.0)

Deactivating (awc_class=3.0)

Deactivating (awc_class=4.0)

Deactivating (awc_class=6.0)

FeaturedSpace: max memory 259522560, total allocated 139407360, free 14912416, used 124494944, increment 0

Sequential: max memory 259522560, total allocated 139407360, free 14912416, used 124494944, increment 0

Initial loss: 9.212637731024866

Initial test loss: 9.212637731024866

Time since start: 249.396

500: time = 18.839000 loss = 6.674677 testLoss 6.181444

Jackknife: leave slope out

Making features

makeFeatures: max memory 259522560, total allocated 139407360, free 73040128, used 66367232, increment -58127712

Amomum_tsaoko_0 slope:

Regularization values: linear/quadratic/product: 0.385, categorical: 0.250, threshold: 1.770, hinge: 0.500

23 samples

Density: max memory 259522560, total allocated 139407360, free 77804888, used 61602472, increment -4764760

linearPredictor: max memory 259522560, total allocated 139407360, free 77804888, used 61602472, increment 0

Deactivating (awc_class=2.0)

Deactivating (awc_class=3.0)

Deactivating (awc_class=4.0)

Deactivating (awc_class=6.0)

FeaturedSpace: max memory 259522560, total allocated 139407360, free 77804888, used 61602472, increment 0

Sequential: max memory 259522560, total allocated 139407360, free 77804888, used 61602472, increment 0

Initial loss: 9.212637731024866

Initial test loss: 9.212637731024866

Time since start: 264.455

440: time = 14.793000 loss = 6.933651 testLoss 6.519266

Jackknife: leave t_clay out

Making features

makeFeatures: max memory 259522560, total allocated 139407360, free 74725096, used 64682264, increment 3079792

Amomum_tsaoko_0 t_clay:

Regularization values: linear/quadratic/product: 0.385, categorical: 0.250, threshold: 1.770, hinge: 0.500

23 samples

Density: max memory 259522560, total allocated 139407360, free 53997104, used 85410256, increment 20727992

linearPredictor: max memory 259522560, total allocated 139407360, free 53997104, used 85410256, increment 0

Deactivating (awc_class=2.0)

Deactivating (awc_class=3.0)

Deactivating (awc_class=4.0)

Deactivating (awc_class=6.0)

FeaturedSpace: max memory 259522560, total allocated 139407360, free 53997104, used 85410256, increment 0

Sequential: max memory 259522560, total allocated 139407360, free 53997104, used 85410256, increment 0

Initial loss: 9.212637731024866

Initial test loss: 9.212637731024866

Time since start: 283.294

500: time = 18.554000 loss = 6.674677 testLoss 6.181444

Jackknife: leave t_oc out

Making features

makeFeatures: max memory 259522560, total allocated 139407360, free 58130568, used 81276792, increment -4133464

Amomum_tsaoko_0 t_oc:

Regularization values: linear/quadratic/product: 0.385, categorical: 0.250, threshold: 1.770, hinge: 0.500

23 samples

Density: max memory 259522560, total allocated 139407360, free 37325008, used 102082352, increment 20805560

linearPredictor: max memory 259522560, total allocated 139407360, free 37325008, used 102082352, increment 0

Deactivating (awc_class=2.0)

Deactivating (awc_class=3.0)

Deactivating (awc_class=4.0)

Deactivating (awc_class=6.0)

FeaturedSpace: max memory 259522560, total allocated 139407360, free 37325008, used 102082352, increment 0

Sequential: max memory 259522560, total allocated 139407360, free 37325008, used 102082352, increment 0

Initial loss: 9.212637731024866

Initial test loss: 9.212637731024866

Time since start: 301.842

500: time = 18.254000 loss = 6.681083 testLoss 6.107206

Jackknife: leave t_sand out

Making features

makeFeatures: max memory 259522560, total allocated 139407360, free 40976024, used 98431336, increment -3651016

Amomum_tsaoko_0 t_sand:

Regularization values: linear/quadratic/product: 0.385, categorical: 0.250, threshold: 1.770, hinge: 0.500

23 samples

Density: max memory 259522560, total allocated 139407360, free 20163528, used 119243832, increment 20812496

linearPredictor: max memory 259522560, total allocated 139407360, free 20163528, used 119243832, increment 0

Deactivating (awc_class=2.0)

Deactivating (awc_class=3.0)

Deactivating (awc_class=4.0)

Deactivating (awc_class=6.0)

FeaturedSpace: max memory 259522560, total allocated 139407360, free 20163528, used 119243832, increment 0

Sequential: max memory 259522560, total allocated 139407360, free 20163528, used 119243832, increment 0

Initial loss: 9.212637731024866

Initial test loss: 9.212637731024866

Time since start: 320.615

500: time = 18.509000 loss = 6.674677 testLoss 6.181444

Jackknife: only aspect

Making features

makeFeatures: max memory 259522560, total allocated 139407360, free 91003360, used 48404000, increment -70839832

Amomum_tsaoko_0 aspect:

Regularization values: linear/quadratic/product: 0.385, categorical: 0.250, threshold: 1.770, hinge: 0.500

23 samples

Density: max memory 259522560, total allocated 139407360, free 87787496, used 51619864, increment 3215864

linearPredictor: max memory 259522560, total allocated 139407360, free 87787496, used 51619864, increment 0

FeaturedSpace: max memory 259522560, total allocated 139407360, free 87787496, used 51619864, increment 0

Sequential: max memory 259522560, total allocated 139407360, free 87787496, used 51619864, increment 0

Initial loss: 9.212637731024866

Initial test loss: 9.212637731024866

60: time = 0.863000 loss = 9.104881 testLoss 9.014655

Res.gain: 0.1077569500609723

Jackknife: only awc_class

Making features

makeFeatures: max memory 259522560, total allocated 139407360, free 59084168, used 80323192, increment 28703328

Amomum_tsaoko_0 awc_class:

Regularization values: linear/quadratic/product: 0.385, categorical: 0.250, threshold: 1.770, hinge: 0.500

23 samples

Density: max memory 259522560, total allocated 139407360, free 59072408, used 80334952, increment 11760

linearPredictor: max memory 259522560, total allocated 139407360, free 59072408, used 80334952, increment 0

Deactivating (awc_class=2.0)

Deactivating (awc_class=3.0)

Deactivating (awc_class=4.0)

Deactivating (awc_class=6.0)

FeaturedSpace: max memory 259522560, total allocated 139407360, free 59072408, used 80334952, increment 0

Sequential: max memory 259522560, total allocated 139407360, free 59072408, used 80334952, increment 0

Initial loss: 9.212637731024866

Initial test loss: 9.212637731024866

Time since start: 321.717

100: time = 0.177000 loss = 9.168644 testLoss 9.266267

Res.gain: 0.04399404918563121

Jackknife: only bio_12

Making features

makeFeatures: max memory 259522560, total allocated 139407360, free 57682224, used 81725136, increment 1390184

Amomum_tsaoko_0 bio_12:

Regularization values: linear/quadratic/product: 0.385, categorical: 0.250, threshold: 1.770, hinge: 0.500

23 samples

Density: max memory 259522560, total allocated 139407360, free 95360920, used 44046440, increment -37678696

linearPredictor: max memory 259522560, total allocated 139407360, free 95360920, used 44046440, increment 0

FeaturedSpace: max memory 259522560, total allocated 139407360, free 95360920, used 44046440, increment 0

Sequential: max memory 259522560, total allocated 139407360, free 95360920, used 44046440, increment 0

Initial loss: 9.212637731024866

Initial test loss: 9.212637731024866

120: time = 0.694000 loss = 7.951014 testLoss 7.687381

Res.gain: 1.2616234322166946

Jackknife: only bio_15

Making features

makeFeatures: max memory 259522560, total allocated 139407360, free 85759104, used 53648256, increment 9601816

Amomum_tsaoko_0 bio_15:

Regularization values: linear/quadratic/product: 0.385, categorical: 0.250, threshold: 1.770, hinge: 0.500

23 samples

Density: max memory 259522560, total allocated 139407360, free 82531912, used 56875448, increment 3227192

linearPredictor: max memory 259522560, total allocated 139407360, free 82531912, used 56875448, increment 0

FeaturedSpace: max memory 259522560, total allocated 139407360, free 82531912, used 56875448, increment 0

Sequential: max memory 259522560, total allocated 139407360, free 82531912, used 56875448, increment 0

Initial loss: 9.212637731024866

Initial test loss: 9.212637731024866

Time since start: 323.372

80: time = 0.914000 loss = 8.853958 testLoss 8.615237

Res.gain: 0.35867982507931373

Jackknife: only bio_17

Making features

makeFeatures: max memory 259522560, total allocated 139407360, free 79110800, used 60296560, increment 3421112

Amomum_tsaoko_0 bio_17:

Regularization values: linear/quadratic/product: 0.385, categorical: 0.250, threshold: 1.770, hinge: 0.500

23 samples

Density: max memory 259522560, total allocated 139407360, free 78326968, used 61080392, increment 783832

linearPredictor: max memory 259522560, total allocated 139407360, free 78326968, used 61080392, increment 0

FeaturedSpace: max memory 259522560, total allocated 139407360, free 78326968, used 61080392, increment 0

Sequential: max memory 259522560, total allocated 139407360, free 78326968, used 61080392, increment 0

Initial loss: 9.212637731024866

Initial test loss: 9.212637731024866

Time since start: 324.436

300: time = 1.040000 loss = 7.800135 testLoss 7.335564

Res.gain: 1.4125031884950774

Jackknife: only bio_4

Making features

makeFeatures: max memory 259522560, total allocated 139407360, free 61350240, used 78057120, increment 16976728

Amomum_tsaoko_0 bio_4:

Regularization values: linear/quadratic/product: 0.385, categorical: 0.250, threshold: 1.770, hinge: 0.500

23 samples

Density: max memory 259522560, total allocated 139407360, free 58260048, used 81147312, increment 3090192

linearPredictor: max memory 259522560, total allocated 139407360, free 58260048, used 81147312, increment 0

FeaturedSpace: max memory 259522560, total allocated 139407360, free 58260048, used 81147312, increment 0

Sequential: max memory 259522560, total allocated 139407360, free 58260048, used 81147312, increment 0

Initial loss: 9.212637731024866

Initial test loss: 9.212637731024866

Time since start: 325.953

120: time = 1.489000 loss = 8.024836 testLoss 7.669194

Res.gain: 1.187801383648246

Jackknife: only bio_6

Making features

makeFeatures: max memory 259522560, total allocated 139407360, free 83685456, used 55721904, increment -25425408

Amomum_tsaoko_0 bio_6:

Regularization values: linear/quadratic/product: 0.385, categorical: 0.250, threshold: 1.770, hinge: 0.500

23 samples

Density: max memory 259522560, total allocated 139407360, free 81041672, used 58365688, increment 2643784

linearPredictor: max memory 259522560, total allocated 139407360, free 81041672, used 58365688, increment 0

FeaturedSpace: max memory 259522560, total allocated 139407360, free 81041672, used 58365688, increment 0

Sequential: max memory 259522560, total allocated 139407360, free 81041672, used 58365688, increment 0

Initial loss: 9.212637731024866

Initial test loss: 9.212637731024866

Time since start: 327.204

180: time = 1.218000 loss = 7.816595 testLoss 7.783946

Res.gain: 1.3960428392438722

Jackknife: only elev

Making features

makeFeatures: max memory 259522560, total allocated 139407360, free 89146200, used 50261160, increment -8104528

Amomum_tsaoko_0 elev:

Regularization values: linear/quadratic/product: 0.385, categorical: 0.250, threshold: 1.770, hinge: 0.500

23 samples

Density: max memory 259522560, total allocated 139407360, free 87410296, used 51997064, increment 1735904

linearPredictor: max memory 259522560, total allocated 139407360, free 87410296, used 51997064, increment 0

FeaturedSpace: max memory 259522560, total allocated 139407360, free 87410296, used 51997064, increment 0

Sequential: max memory 259522560, total allocated 139407360, free 87410296, used 51997064, increment 0

Initial loss: 9.212637731024866

Initial test loss: 9.212637731024866

Time since start: 328.212

160: time = 0.978000 loss = 8.855730 testLoss 8.793942

Res.gain: 0.35690723992554396

Jackknife: only s_caco3

Making features

makeFeatures: max memory 259522560, total allocated 139407360, free 64757648, used 74649712, increment 22652648

Amomum_tsaoko_0 s_caco3:

Regularization values: linear/quadratic/product: 0.385, categorical: 0.250, threshold: 1.770, hinge: 0.500

23 samples

Density: max memory 259522560, total allocated 139407360, free 63974448, used 75432912, increment 783200

linearPredictor: max memory 259522560, total allocated 139407360, free 63894248, used 75513112, increment 80200

FeaturedSpace: max memory 259522560, total allocated 139407360, free 63894248, used 75513112, increment 0

Sequential: max memory 259522560, total allocated 139407360, free 63894248, used 75513112, increment 0

Initial loss: 9.212637731024866

Initial test loss: 9.212637731024866

140: time = 0.684000 loss = 8.672303 testLoss 8.649674

Res.gain: 0.5403351267727263

Jackknife: only s_ph_h2o

Making features

makeFeatures: max memory 259522560, total allocated 139407360, free 60646984, used 78760376, increment 3247264

Amomum_tsaoko_0 s_ph_h2o:

Regularization values: linear/quadratic/product: 0.385, categorical: 0.250, threshold: 1.770, hinge: 0.500

23 samples

Density: max memory 259522560, total allocated 139407360, free 59870952, used 79536408, increment 776032

linearPredictor: max memory 259522560, total allocated 139407360, free 59790752, used 79616608, increment 80200

FeaturedSpace: max memory 259522560, total allocated 139407360, free 59790752, used 79616608, increment 0

Sequential: max memory 259522560, total allocated 139407360, free 59790752, used 79616608, increment 0

Initial loss: 9.212637731024866

Initial test loss: 9.212637731024866

Time since start: 329.969

340: time = 1.025000 loss = 8.345314 testLoss 8.300987

Res.gain: 0.8673242286911176

Jackknife: only slope

Making features

makeFeatures: max memory 259522560, total allocated 139407360, free 91415480, used 47991880, increment -31624728

Amomum_tsaoko_0 slope:

Regularization values: linear/quadratic/product: 0.385, categorical: 0.250, threshold: 1.770, hinge: 0.500

23 samples

Density: max memory 259522560, total allocated 139407360, free 89149496, used 50257864, increment 2265984

linearPredictor: max memory 259522560, total allocated 139407360, free 89149496, used 50257864, increment 0

FeaturedSpace: max memory 259522560, total allocated 139407360, free 89149496, used 50257864, increment 0

Sequential: max memory 259522560, total allocated 139407360, free 89149496, used 50257864, increment 0

Initial loss: 9.212637731024866

Initial test loss: 9.212637731024866

Time since start: 332.12

240: time = 2.123000 loss = 9.055976 testLoss 8.874093

Res.gain: 0.15666198872488657

Jackknife: only t_clay

Making features

makeFeatures: max memory 259522560, total allocated 139407360, free 66914160, used 72493200, increment 22235336

Amomum_tsaoko_0 t_clay:

Regularization values: linear/quadratic/product: 0.385, categorical: 0.250, threshold: 1.770, hinge: 0.500

23 samples

Density: max memory 259522560, total allocated 139407360, free 65288704, used 74118656, increment 1625456

linearPredictor: max memory 259522560, total allocated 139407360, free 65288704, used 74118656, increment 0

FeaturedSpace: max memory 259522560, total allocated 139407360, free 65288704, used 74118656, increment 0

Sequential: max memory 259522560, total allocated 139407360, free 65288704, used 74118656, increment 0

Initial loss: 9.212637731024866

Initial test loss: 9.212637731024866

120: time = 0.511000 loss = 8.761769 testLoss 8.738689

Res.gain: 0.45086865370910445

Jackknife: only t_oc

Making features

makeFeatures: max memory 259522560, total allocated 139407360, free 62967120, used 76440240, increment 2321584

Amomum_tsaoko_0 t_oc:

Regularization values: linear/quadratic/product: 0.385, categorical: 0.250, threshold: 1.770, hinge: 0.500

23 samples

Density: max memory 259522560, total allocated 139407360, free 62177624, used 77229736, increment 789496

linearPredictor: max memory 259522560, total allocated 139407360, free 62177624, used 77229736, increment 0

FeaturedSpace: max memory 259522560, total allocated 139407360, free 62177624, used 77229736, increment 0

Sequential: max memory 259522560, total allocated 139407360, free 62177624, used 77229736, increment 0

Initial loss: 9.212637731024866

Initial test loss: 9.212637731024866

Time since start: 333.185

120: time = 0.490000 loss = 8.640603 testLoss 8.960198

Res.gain: 0.5720350885666239

Jackknife: only t_sand

Making features

makeFeatures: max memory 259522560, total allocated 139407360, free 58295560, used 81111800, increment 3882064

Amomum_tsaoko_0 t_sand:

Regularization values: linear/quadratic/product: 0.385, categorical: 0.250, threshold: 1.770, hinge: 0.500

23 samples

Density: max memory 259522560, total allocated 139407360, free 57514088, used 81893272, increment 781472

linearPredictor: max memory 259522560, total allocated 139407360, free 57047224, used 82360136, increment 466864

FeaturedSpace: max memory 259522560, total allocated 139407360, free 57047224, used 82360136, increment 0

Sequential: max memory 259522560, total allocated 139407360, free 57047224, used 82360136, increment 0

Initial loss: 9.212637731024866

Initial test loss: 9.212637731024866

160: time = 0.658000 loss = 9.094963 testLoss 9.046836

Res.gain: 0.11767437834753558

getSamples: max memory 259522560, total allocated 139407360, free 79740736, used 59666624, increment -22693512

Making features

makeFeatures: max memory 259522560, total allocated 139407360, free 70820560, used 68586800, increment 8920176

Amomum_tsaoko_1:

Regularization values: linear/quadratic/product: 0.385, categorical: 0.250, threshold: 1.770, hinge: 0.500

Time since start: 334.347

23 samples

Density: max memory 259522560, total allocated 139407360, free 72498680, used 66908680, increment -1678120

linearPredictor: max memory 259522560, total allocated 139407360, free 72498680, used 66908680, increment 0

Deactivating (awc_class=2.0)

Deactivating (awc_class=3.0)

Deactivating (awc_class=4.0)

Deactivating (awc_class=6.0)

FeaturedSpace: max memory 259522560, total allocated 139407360, free 72498680, used 66908680, increment 0

Sequential: max memory 259522560, total allocated 139407360, free 72498680, used 66908680, increment 0

Initial loss: 9.212438170058329

Initial test loss: 9.212438170058329

Time since start: 353.617

500: time = 19.238000 loss = 7.196532 testLoss 7.581896

Resulting gain: 2.0159057387817017

Projecting...

Writing file E:\Amomum_tsaoko\results2\Amomum_tsaoko_1.asc

Time since start: 357.656

Writing E:\Amomum_tsaoko\results2\plots\Amomum_tsaoko_1.png

Time since start: 359.011

Projecting...

Writing file E:\Amomum_tsaoko\results2\Amomum_tsaoko_1_LGM.asc

Writing file E:\Amomum_tsaoko\results2\Amomum_tsaoko_1_LGM_clamping.asc

Time since start: 363.93

Writing E:\Amomum_tsaoko\results2\plots\Amomum_tsaoko_1_LGM.png

Time since start: 365.287

Writing E:\Amomum_tsaoko\results2\plots\Amomum_tsaoko_1_LGM_clamping.png

Time since start: 366.704

Writing file E:\Amomum_tsaoko\results2\Amomum_tsaoko_1_LGM_novel.asc

Writing file E:\Amomum_tsaoko\results2\Amomum_tsaoko_1_LGM_novel_limiting.asc

Time since start: 370.948

Projecting...

Writing file E:\Amomum_tsaoko\results2\Amomum_tsaoko_1_MH.asc

Writing file E:\Amomum_tsaoko\results2\Amomum_tsaoko_1_MH_clamping.asc

Time since start: 375.786

Writing E:\Amomum_tsaoko\results2\plots\Amomum_tsaoko_1_MH.png

Time since start: 377.174

Writing E:\Amomum_tsaoko\results2\plots\Amomum_tsaoko_1_MH_clamping.png

Time since start: 378.625

Writing file E:\Amomum_tsaoko\results2\Amomum_tsaoko_1_MH_novel.asc

Writing file E:\Amomum_tsaoko\results2\Amomum_tsaoko_1_MH_novel_limiting.asc

Time since start: 382.972

Projecting...

Writing file E:\Amomum_tsaoko\results2\Amomum_tsaoko_1_SSP126-2050S.asc

Writing file E:\Amomum_tsaoko\results2\Amomum_tsaoko_1_SSP126-2050S_clamping.asc

Time since start: 386.96

Writing E:\Amomum_tsaoko\results2\plots\Amomum_tsaoko_1_SSP126-2050S.png

Time since start: 388.331

Writing E:\Amomum_tsaoko\results2\plots\Amomum_tsaoko_1_SSP126-2050S_clamping.png

Time since start: 389.726

Writing file E:\Amomum_tsaoko\results2\Amomum_tsaoko_1_SSP126-2050S_novel.asc

Writing file E:\Amomum_tsaoko\results2\Amomum_tsaoko_1_SSP126-2050S_novel_limiting.asc

Time since start: 394.283

Projecting...

Writing file E:\Amomum_tsaoko\results2\Amomum_tsaoko_1_SSP126-2090S.asc

Writing file E:\Amomum_tsaoko\results2\Amomum_tsaoko_1_SSP126-2090S_clamping.asc

Time since start: 398.357

Writing E:\Amomum_tsaoko\results2\plots\Amomum_tsaoko_1_SSP126-2090S.png

Time since start: 399.689

Writing E:\Amomum_tsaoko\results2\plots\Amomum_tsaoko_1_SSP126-2090S_clamping.png

Time since start: 400.854

Writing file E:\Amomum_tsaoko\results2\Amomum_tsaoko_1_SSP126-2090S_novel.asc

Writing file E:\Amomum_tsaoko\results2\Amomum_tsaoko_1_SSP126-2090S_novel_limiting.asc

Time since start: 405.389

Projecting...

Writing file E:\Amomum_tsaoko\results2\Amomum_tsaoko_1_SSP585-2050S.asc

Writing file E:\Amomum_tsaoko\results2\Amomum_tsaoko_1_SSP585-2050S_clamping.asc

Time since start: 409.415

Writing E:\Amomum_tsaoko\results2\plots\Amomum_tsaoko_1_SSP585-2050S.png

Time since start: 410.718

Writing E:\Amomum_tsaoko\results2\plots\Amomum_tsaoko_1_SSP585-2050S_clamping.png

Time since start: 412.08

Writing file E:\Amomum_tsaoko\results2\Amomum_tsaoko_1_SSP585-2050S_novel.asc

Writing file E:\Amomum_tsaoko\results2\Amomum_tsaoko_1_SSP585-2050S_novel_limiting.asc

Time since start: 416.772

Projecting...

Writing file E:\Amomum_tsaoko\results2\Amomum_tsaoko_1_SSP585-2090S.asc

Writing file E:\Amomum_tsaoko\results2\Amomum_tsaoko_1_SSP585-2090S_clamping.asc

Time since start: 421.268

Writing E:\Amomum_tsaoko\results2\plots\Amomum_tsaoko_1_SSP585-2090S.png

Time since start: 422.563

Writing E:\Amomum_tsaoko\results2\plots\Amomum_tsaoko_1_SSP585-2090S_clamping.png

Time since start: 423.99

Writing file E:\Amomum_tsaoko\results2\Amomum_tsaoko_1_SSP585-2090S_novel.asc

Writing file E:\Amomum_tsaoko\results2\Amomum_tsaoko_1_SSP585-2090S_novel_limiting.asc

Time since start: 428.598

Amomum_tsaoko_1 response curves

Response curve: only aspect

Making features

makeFeatures: max memory 259522560, total allocated 139407360, free 70488408, used 68918952, increment 2010272

Amomum_tsaoko_1 aspect:

Regularization values: linear/quadratic/product: 0.385, categorical: 0.250, threshold: 1.770, hinge: 0.500

23 samples

Density: max memory 259522560, total allocated 139407360, free 67950944, used 71456416, increment 2537464

linearPredictor: max memory 259522560, total allocated 139407360, free 67950944, used 71456416, increment 0

FeaturedSpace: max memory 259522560, total allocated 139407360, free 67950944, used 71456416, increment 0

Sequential: max memory 259522560, total allocated 139407360, free 67950944, used 71456416, increment 0

Initial loss: 9.212438170058329

Time since start: 431.087

120: time = 1.543000 loss = 9.055557

Resulting gain: 0.15688152711631353

Amomum_tsaoko_1 response curves

Response curve: only awc_class

Making features

makeFeatures: max memory 259522560, total allocated 139407360, free 49133064, used 90274296, increment 18817880

Amomum_tsaoko_1 awc_class:

Regularization values: linear/quadratic/product: 0.385, categorical: 0.250, threshold: 1.770, hinge: 0.500

23 samples

Density: max memory 259522560, total allocated 139407360, free 49036352, used 90371008, increment 96712

linearPredictor: max memory 259522560, total allocated 139407360, free 48956168, used 90451192, increment 80184

Deactivating (awc_class=2.0)

Deactivating (awc_class=3.0)

Deactivating (awc_class=4.0)

Deactivating (awc_class=6.0)

FeaturedSpace: max memory 259522560, total allocated 139407360, free 48956168, used 90451192, increment 0

Sequential: max memory 259522560, total allocated 139407360, free 48956168, used 90451192, increment 0

Initial loss: 9.212438170058329

100: time = 0.201000 loss = 9.120973

Resulting gain: 0.09146524519810306

Amomum_tsaoko_1 response curves

Response curve: only bio_12

Making features

makeFeatures: max memory 259522560, total allocated 139407360, free 44129376, used 95277984, increment 4826792

Amomum_tsaoko_1 bio_12:

Regularization values: linear/quadratic/product: 0.385, categorical: 0.250, threshold: 1.770, hinge: 0.500

23 samples

Density: max memory 259522560, total allocated 139407360, free 42453576, used 96953784, increment 1675800

linearPredictor: max memory 259522560, total allocated 139407360, free 42453576, used 96953784, increment 0

FeaturedSpace: max memory 259522560, total allocated 139407360, free 42453576, used 96953784, increment 0

Sequential: max memory 259522560, total allocated 139407360, free 42453576, used 96953784, increment 0

Initial loss: 9.212438170058329

80: time = 0.538000 loss = 8.257979

Resulting gain: 0.9544591257997848

Amomum_tsaoko_1 response curves

Response curve: only bio_15

Making features

makeFeatures: max memory 259522560, total allocated 139407360, free 45674504, used 93732856, increment -3220928

Amomum_tsaoko_1 bio_15:

Regularization values: linear/quadratic/product: 0.385, categorical: 0.250, threshold: 1.770, hinge: 0.500

23 samples

Density: max memory 259522560, total allocated 139407360, free 42441480, used 96965880, increment 3233024

linearPredictor: max memory 259522560, total allocated 139407360, free 42441480, used 96965880, increment 0

FeaturedSpace: max memory 259522560, total allocated 139407360, free 42441480, used 96965880, increment 0

Sequential: max memory 259522560, total allocated 139407360, free 42441480, used 96965880, increment 0

Initial loss: 9.212438170058329

Time since start: 433.291

120: time = 1.230000 loss = 8.820872

Resulting gain: 0.39156581804456536

Amomum_tsaoko_1 response curves

Response curve: only bio_17

Making features

makeFeatures: max memory 259522560, total allocated 139407360, free 36713472, used 102693888, increment 5728008

Amomum_tsaoko_1 bio_17:

Regularization values: linear/quadratic/product: 0.385, categorical: 0.250, threshold: 1.770, hinge: 0.500

23 samples

Density: max memory 259522560, total allocated 139407360, free 35853096, used 103554264, increment 860376

linearPredictor: max memory 259522560, total allocated 139407360, free 35853096, used 103554264, increment 0

FeaturedSpace: max memory 259522560, total allocated 139407360, free 35853096, used 103554264, increment 0

Sequential: max memory 259522560, total allocated 139407360, free 35853096, used 103554264, increment 0

Initial loss: 9.212438170058329

160: time = 0.720000 loss = 8.500821

Resulting gain: 0.711616991768425

Amomum_tsaoko_1 response curves

Response curve: only bio_4

Making features

makeFeatures: max memory 259522560, total allocated 139407360, free 61765784, used 77641576, increment -25912688

Amomum_tsaoko_1 bio_4:

Regularization values: linear/quadratic/product: 0.385, categorical: 0.250, threshold: 1.770, hinge: 0.500

23 samples

Density: max memory 259522560, total allocated 139407360, free 58879840, used 80527520, increment 2885944

linearPredictor: max memory 259522560, total allocated 139407360, free 58879840, used 80527520, increment 0

FeaturedSpace: max memory 259522560, total allocated 139407360, free 58879840, used 80527520, increment 0

Sequential: max memory 259522560, total allocated 139407360, free 58879840, used 80527520, increment 0

Initial loss: 9.212438170058329

Time since start: 435.682

140: time = 1.471000 loss = 7.863452

Resulting gain: 1.3489857849355937

Amomum_tsaoko_1 response curves

Response curve: only bio_6

Making features

makeFeatures: max memory 259522560, total allocated 139407360, free 38218592, used 101188768, increment 20661248

Amomum_tsaoko_1 bio_6:

Regularization values: linear/quadratic/product: 0.385, categorical: 0.250, threshold: 1.770, hinge: 0.500

23 samples

Density: max memory 259522560, total allocated 139407360, free 35613432, used 103793928, increment 2605160

linearPredictor: max memory 259522560, total allocated 139407360, free 35613432, used 103793928, increment 0

FeaturedSpace: max memory 259522560, total allocated 139407360, free 35613432, used 103793928, increment 0

Sequential: max memory 259522560, total allocated 139407360, free 35613432, used 103793928, increment 0

Initial loss: 9.212438170058329

Time since start: 437.963

220: time = 2.163000 loss = 8.112130

Resulting gain: 1.1003084860224988

Amomum_tsaoko_1 response curves

Response curve: only elev

Making features

makeFeatures: max memory 259522560, total allocated 139407360, free 66157840, used 73249520, increment -30544408

Amomum_tsaoko_1 elev:

Regularization values: linear/quadratic/product: 0.385, categorical: 0.250, threshold: 1.770, hinge: 0.500

23 samples

Density: max memory 259522560, total allocated 139407360, free 64578392, used 74828968, increment 1579448

linearPredictor: max memory 259522560, total allocated 139407360, free 64578392, used 74828968, increment 0

FeaturedSpace: max memory 259522560, total allocated 139407360, free 64578392, used 74828968, increment 0

Sequential: max memory 259522560, total allocated 139407360, free 64578392, used 74828968, increment 0

Initial loss: 9.212438170058329

100: time = 0.713000 loss = 9.158576

Resulting gain: 0.05386194261941846

Amomum_tsaoko_1 response curves

Response curve: only s_caco3

Making features

makeFeatures: max memory 259522560, total allocated 139407360, free 37046064, used 102361296, increment 27532328

Amomum_tsaoko_1 s_caco3:

Regularization values: linear/quadratic/product: 0.385, categorical: 0.250, threshold: 1.770, hinge: 0.500

23 samples

Density: max memory 259522560, total allocated 139407360, free 36265000, used 103142360, increment 781064

linearPredictor: max memory 259522560, total allocated 139407360, free 36265000, used 103142360, increment 0

FeaturedSpace: max memory 259522560, total allocated 139407360, free 36265000, used 103142360, increment 0

Sequential: max memory 259522560, total allocated 139407360, free 36265000, used 103142360, increment 0

Initial loss: 9.212438170058329

Time since start: 439.546

120: time = 0.634000 loss = 8.897373

Resulting gain: 0.3150649265486791

Amomum_tsaoko_1 response curves

Response curve: only s_ph_h2o

Making features

makeFeatures: max memory 259522560, total allocated 139407360, free 68427312, used 70980048, increment -32162312

Amomum_tsaoko_1 s_ph_h2o:

Regularization values: linear/quadratic/product: 0.385, categorical: 0.250, threshold: 1.770, hinge: 0.500

23 samples

Density: max memory 259522560, total allocated 139407360, free 67643104, used 71764256, increment 784208

linearPredictor: max memory 259522560, total allocated 139407360, free 67643104, used 71764256, increment 0

FeaturedSpace: max memory 259522560, total allocated 139407360, free 67643104, used 71764256, increment 0

Sequential: max memory 259522560, total allocated 139407360, free 67643104, used 71764256, increment 0

Initial loss: 9.212438170058329

240: time = 0.821000 loss = 8.928114

Resulting gain: 0.28432381869502343

Amomum_tsaoko_1 response curves

Response curve: only slope

Making features

makeFeatures: max memory 259522560, total allocated 139407360, free 59457768, used 79949592, increment 8185336

Amomum_tsaoko_1 slope:

Regularization values: linear/quadratic/product: 0.385, categorical: 0.250, threshold: 1.770, hinge: 0.500

23 samples

Time since start: 440.549

Density: max memory 259522560, total allocated 139407360, free 57058824, used 82348536, increment 2398944

linearPredictor: max memory 259522560, total allocated 139407360, free 57058824, used 82348536, increment 0

FeaturedSpace: max memory 259522560, total allocated 139407360, free 57058824, used 82348536, increment 0

Sequential: max memory 259522560, total allocated 139407360, free 57058824, used 82348536, increment 0

Initial loss: 9.212438170058329

Time since start: 443.468

300: time = 2.919000 loss = 9.006138

Resulting gain: 0.20630037501632437

Amomum_tsaoko_1 response curves

Response curve: only t_clay

Making features

makeFeatures: max memory 259522560, total allocated 139407360, free 71594944, used 67812416, increment -14536120

Amomum_tsaoko_1 t_clay:

Regularization values: linear/quadratic/product: 0.385, categorical: 0.250, threshold: 1.770, hinge: 0.500

23 samples

Density: max memory 259522560, total allocated 139407360, free 70655000, used 68752360, increment 939944

linearPredictor: max memory 259522560, total allocated 139407360, free 70655000, used 68752360, increment 0

FeaturedSpace: max memory 259522560, total allocated 139407360, free 70655000, used 68752360, increment 0

Sequential: max memory 259522560, total allocated 139407360, free 70655000, used 68752360, increment 0

Initial loss: 9.212438170058329

220: time = 0.768000 loss = 8.536387

Resulting gain: 0.6760511402019613

Amomum_tsaoko_1 response curves

Response curve: only t_oc

Making features

makeFeatures: max memory 259522560, total allocated 139407360, free 62600528, used 76806832, increment 8054472

Amomum_tsaoko_1 t_oc:

Regularization values: linear/quadratic/product: 0.385, categorical: 0.250, threshold: 1.770, hinge: 0.500

23 samples

Density: max memory 259522560, total allocated 139407360, free 61796528, used 77610832, increment 804000

linearPredictor: max memory 259522560, total allocated 139407360, free 61796528, used 77610832, increment 0

FeaturedSpace: max memory 259522560, total allocated 139407360, free 61796528, used 77610832, increment 0

Sequential: max memory 259522560, total allocated 139407360, free 61796528, used 77610832, increment 0

Initial loss: 9.212438170058329

Time since start: 444.868

120: time = 0.469000 loss = 8.869531

Resulting gain: 0.3429076067690193

Amomum_tsaoko_1 response curves

Response curve: only t_sand

Making features

makeFeatures: max memory 259522560, total allocated 139407360, free 54438296, used 84969064, increment 7358232

Amomum_tsaoko_1 t_sand:

Regularization values: linear/quadratic/product: 0.385, categorical: 0.250, threshold: 1.770, hinge: 0.500

23 samples

Density: max memory 259522560, total allocated 139407360, free 53453424, used 85953936, increment 984872

linearPredictor: max memory 259522560, total allocated 139407360, free 53373240, used 86034120, increment 80184

FeaturedSpace: max memory 259522560, total allocated 139407360, free 53373240, used 86034120, increment 0

Sequential: max memory 259522560, total allocated 139407360, free 53373240, used 86034120, increment 0

Initial loss: 9.212438170058329

100: time = 0.521000 loss = 8.901530

Resulting gain: 0.3109083772355188

Amomum_tsaoko_1 response curves

Time since start: 446.838

Jackknife: leave aspect out

Making features

makeFeatures: max memory 259522560, total allocated 139407360, free 38812072, used 100595288, increment 14561168

Amomum_tsaoko_1 aspect:

Regularization values: linear/quadratic/product: 0.385, categorical: 0.250, threshold: 1.770, hinge: 0.500

23 samples

Density: max memory 259522560, total allocated 139407360, free 50878720, used 88528640, increment -12066648

linearPredictor: max memory 259522560, total allocated 139407360, free 50878720, used 88528640, increment 0

Deactivating (awc_class=2.0)

Deactivating (awc_class=3.0)

Deactivating (awc_class=4.0)

Deactivating (awc_class=6.0)

FeaturedSpace: max memory 259522560, total allocated 139407360, free 50878720, used 88528640, increment 0

Sequential: max memory 259522560, total allocated 139407360, free 50878720, used 88528640, increment 0

Initial loss: 9.212438170058329

Initial test loss: 9.212438170058329

Time since start: 460.124

400: time = 13.017000 loss = 7.303172 testLoss 7.565443

Jackknife: leave awc_class out

Making features

makeFeatures: max memory 259522560, total allocated 139407360, free 52585432, used 86821928, increment -1706712

Amomum_tsaoko_1 awc_class:

Regularization values: linear/quadratic/product: 0.385, categorical: 0.250, threshold: 1.770, hinge: 0.500

23 samples

Density: max memory 259522560, total allocated 139407360, free 31476448, used 107930912, increment 21108984

linearPredictor: max memory 259522560, total allocated 139407360, free 31396264, used 108011096, increment 80184

FeaturedSpace: max memory 259522560, total allocated 139407360, free 31396264, used 108011096, increment 0

Sequential: max memory 259522560, total allocated 139407360, free 31396264, used 108011096, increment 0

Initial loss: 9.212438170058329

Initial test loss: 9.212438170058329

Time since start: 479.714

500: time = 19.258000 loss = 7.197032 testLoss 7.550631

Jackknife: leave bio_12 out

Making features

makeFeatures: max memory 259522560, total allocated 139407360, free 30403864, used 109003496, increment 992400

Amomum_tsaoko_1 bio_12:

Regularization values: linear/quadratic/product: 0.385, categorical: 0.250, threshold: 1.770, hinge: 0.500

23 samples

Density: max memory 259522560, total allocated 139407360, free 10596264, used 128811096, increment 19807600

linearPredictor: max memory 259522560, total allocated 139407360, free 10516080, used 128891280, increment 80184

Deactivating (awc_class=2.0)

Deactivating (awc_class=3.0)

Deactivating (awc_class=4.0)

Deactivating (awc_class=6.0)

FeaturedSpace: max memory 259522560, total allocated 139407360, free 10516080, used 128891280, increment 0

Sequential: max memory 259522560, total allocated 139407360, free 10516080, used 128891280, increment 0

Initial loss: 9.212438170058329

Initial test loss: 9.212438170058329

Time since start: 498.641

500: time = 18.614000 loss = 7.232312 testLoss 7.541863

Jackknife: leave bio_15 out

Making features

makeFeatures: max memory 259522560, total allocated 139407360, free 61881784, used 77525576, increment -51365704

Amomum_tsaoko_1 bio_15:

Regularization values: linear/quadratic/product: 0.385, categorical: 0.250, threshold: 1.770, hinge: 0.500

23 samples

Density: max memory 259522560, total allocated 139407360, free 72581656, used 66825704, increment -10699872

linearPredictor: max memory 259522560, total allocated 139407360, free 72581656, used 66825704, increment 0

Deactivating (awc_class=2.0)

Deactivating (awc_class=3.0)

Deactivating (awc_class=4.0)

Deactivating (awc_class=6.0)

FeaturedSpace: max memory 259522560, total allocated 139407360, free 72581656, used 66825704, increment 0

Sequential: max memory 259522560, total allocated 139407360, free 72581656, used 66825704, increment 0

Initial loss: 9.212438170058329

Initial test loss: 9.212438170058329

Time since start: 514.797

500: time = 15.854000 loss = 7.251247 testLoss 7.731240

Jackknife: leave bio_17 out

Making features

makeFeatures: max memory 259522560, total allocated 139407360, free 67392064, used 72015296, increment 5189592

Amomum_tsaoko_1 bio_17:

Regularization values: linear/quadratic/product: 0.385, categorical: 0.250, threshold: 1.770, hinge: 0.500

23 samples

Density: max memory 259522560, total allocated 139407360, free 46991080, used 92416280, increment 20400984

linearPredictor: max memory 259522560, total allocated 139407360, free 46991080, used 92416280, increment 0

Deactivating (awc_class=2.0)

Deactivating (awc_class=3.0)

Deactivating (awc_class=4.0)

Deactivating (awc_class=6.0)

FeaturedSpace: max memory 259522560, total allocated 139407360, free 46991080, used 92416280, increment 0

Sequential: max memory 259522560, total allocated 139407360, free 46991080, used 92416280, increment 0

Initial loss: 9.212438170058329

Initial test loss: 9.212438170058329

Time since start: 534.042

500: time = 19.037000 loss = 7.207163 testLoss 7.507442

Jackknife: leave bio_4 out

Making features

makeFeatures: max memory 259522560, total allocated 139407360, free 59922456, used 79484904, increment -12931376

Amomum_tsaoko_1 bio_4:

Regularization values: linear/quadratic/product: 0.385, categorical: 0.250, threshold: 1.770, hinge: 0.500

23 samples

Density: max memory 259522560, total allocated 139407360, free 41336320, used 98071040, increment 18586136

linearPredictor: max memory 259522560, total allocated 139407360, free 41336320, used 98071040, increment 0

Deactivating (awc_class=2.0)

Deactivating (awc_class=3.0)

Deactivating (awc_class=4.0)

Deactivating (awc_class=6.0)

FeaturedSpace: max memory 259522560, total allocated 139407360, free 41336320, used 98071040, increment 0

Sequential: max memory 259522560, total allocated 139407360, free 41336320, used 98071040, increment 0

Initial loss: 9.212438170058329

Initial test loss: 9.212438170058329

Time since start: 549.34

500: time = 14.984000 loss = 7.232469 testLoss 7.533808

Jackknife: leave bio_6 out

Making features

makeFeatures: max memory 259522560, total allocated 139407360, free 38934840, used 100472520, increment 2401480

Amomum_tsaoko_1 bio_6:

Regularization values: linear/quadratic/product: 0.385, categorical: 0.250, threshold: 1.770, hinge: 0.500

23 samples

Density: max memory 259522560, total allocated 139407360, free 19612256, used 119795104, increment 19322584

linearPredictor: max memory 259522560, total allocated 139407360, free 19612256, used 119795104, increment 0

Deactivating (awc_class=2.0)

Deactivating (awc_class=3.0)

Deactivating (awc_class=4.0)

Deactivating (awc_class=6.0)

FeaturedSpace: max memory 259522560, total allocated 139407360, free 19612256, used 119795104, increment 0

Sequential: max memory 259522560, total allocated 139407360, free 19612256, used 119795104, increment 0

Initial loss: 9.212438170058329

Initial test loss: 9.212438170058329

Time since start: 567.174

500: time = 17.540000 loss = 7.222171 testLoss 7.484202

Jackknife: leave elev out

Making features

makeFeatures: max memory 259522560, total allocated 139407360, free 69006480, used 70400880, increment -49394224

Amomum_tsaoko_1 elev:

Regularization values: linear/quadratic/product: 0.385, categorical: 0.250, threshold: 1.770, hinge: 0.500

23 samples

Density: max memory 259522560, total allocated 139407360, free 74402736, used 65004624, increment -5396256

linearPredictor: max memory 259522560, total allocated 139407360, free 74322552, used 65084808, increment 80184

Deactivating (awc_class=2.0)

Deactivating (awc_class=3.0)

Deactivating (awc_class=4.0)

Deactivating (awc_class=6.0)

FeaturedSpace: max memory 259522560, total allocated 139407360, free 74322552, used 65084808, increment 0

Sequential: max memory 259522560, total allocated 139407360, free 74322552, used 65084808, increment 0

Initial loss: 9.212438170058329

Initial test loss: 9.212438170058329

Time since start: 585.763

500: time = 18.264000 loss = 7.205363 testLoss 7.574510

Jackknife: leave s_caco3 out

Making features

makeFeatures: max memory 259522560, total allocated 139407360, free 74532440, used 64874920, increment -209888

Amomum_tsaoko_1 s_caco3:

Regularization values: linear/quadratic/product: 0.385, categorical: 0.250, threshold: 1.770, hinge: 0.500

23 samples

Density: max memory 259522560, total allocated 139407360, free 53905024, used 85502336, increment 20627416

linearPredictor: max memory 259522560, total allocated 139407360, free 53905024, used 85502336, increment 0

Deactivating (awc_class=2.0)

Deactivating (awc_class=3.0)

Deactivating (awc_class=4.0)

Deactivating (awc_class=6.0)

FeaturedSpace: max memory 259522560, total allocated 139407360, free 53905024, used 85502336, increment 0

Sequential: max memory 259522560, total allocated 139407360, free 53905024, used 85502336, increment 0

Initial loss: 9.212438170058329

Initial test loss: 9.212438170058329

Time since start: 605.668

500: time = 19.576000 loss = 7.199948 testLoss 7.576862

Jackknife: leave s_ph_h2o out

Making features

makeFeatures: max memory 259522560, total allocated 139407360, free 58080440, used 81326920, increment -4175416

Amomum_tsaoko_1 s_ph_h2o:

Regularization values: linear/quadratic/product: 0.385, categorical: 0.250, threshold: 1.770, hinge: 0.500

23 samples

Density: max memory 259522560, total allocated 139407360, free 37818624, used 101588736, increment 20261816

linearPredictor: max memory 259522560, total allocated 139407360, free 37738440, used 101668920, increment 80184

Deactivating (awc_class=2.0)

Deactivating (awc_class=3.0)

Deactivating (awc_class=4.0)

Deactivating (awc_class=6.0)

FeaturedSpace: max memory 259522560, total allocated 139407360, free 37738440, used 101668920, increment 0

Sequential: max memory 259522560, total allocated 139407360, free 37738440, used 101668920, increment 0

Initial loss: 9.212438170058329

Initial test loss: 9.212438170058329

Time since start: 625.399

500: time = 19.412000 loss = 7.199703 testLoss 7.584769

Jackknife: leave slope out

Making features

makeFeatures: max memory 259522560, total allocated 139407360, free 36531072, used 102876288, increment 1207368

Amomum_tsaoko_1 slope:

Regularization values: linear/quadratic/product: 0.385, categorical: 0.250, threshold: 1.770, hinge: 0.500

23 samples

Density: max memory 259522560, total allocated 139407360, free 17713992, used 121693368, increment 18817080

linearPredictor: max memory 259522560, total allocated 139407360, free 17713992, used 121693368, increment 0

Deactivating (awc_class=2.0)

Deactivating (awc_class=3.0)

Deactivating (awc_class=4.0)

Deactivating (awc_class=6.0)

FeaturedSpace: max memory 259522560, total allocated 139407360, free 17713992, used 121693368, increment 0

Sequential: max memory 259522560, total allocated 139407360, free 17713992, used 121693368, increment 0

Initial loss: 9.212438170058329

Initial test loss: 9.212438170058329

Time since start: 642.83

500: time = 17.097000 loss = 7.351383 testLoss 7.846719

Jackknife: leave t_clay out

Making features

makeFeatures: max memory 259522560, total allocated 139407360, free 89291856, used 50115504, increment -71577864

Amomum_tsaoko_1 t_clay:

Regularization values: linear/quadratic/product: 0.385, categorical: 0.250, threshold: 1.770, hinge: 0.500

23 samples

Density: max memory 259522560, total allocated 139407360, free 69093880, used 70313480, increment 20197976

linearPredictor: max memory 259522560, total allocated 139407360, free 68329976, used 71077384, increment 763904

Deactivating (awc_class=2.0)

Deactivating (awc_class=3.0)

Deactivating (awc_class=4.0)

Deactivating (awc_class=6.0)

FeaturedSpace: max memory 259522560, total allocated 139407360, free 68329976, used 71077384, increment 0

Sequential: max memory 259522560, total allocated 139407360, free 68329976, used 71077384, increment 0

Initial loss: 9.212438170058329

Initial test loss: 9.212438170058329

Time since start: 662.573

500: time = 19.445000 loss = 7.204472 testLoss 7.645146

Jackknife: leave t_oc out

Making features

makeFeatures: max memory 259522560, total allocated 139407360, free 73939864, used 65467496, increment -5609888

Amomum_tsaoko_1 t_oc:

Regularization values: linear/quadratic/product: 0.385, categorical: 0.250, threshold: 1.770, hinge: 0.500

23 samples

Density: max memory 259522560, total allocated 139407360, free 53057464, used 86349896, increment 20882400

linearPredictor: max memory 259522560, total allocated 139407360, free 53057464, used 86349896, increment 0

Deactivating (awc_class=2.0)

Deactivating (awc_class=3.0)

Deactivating (awc_class=4.0)

Deactivating (awc_class=6.0)

FeaturedSpace: max memory 259522560, total allocated 139407360, free 53057464, used 86349896, increment 0

Sequential: max memory 259522560, total allocated 139407360, free 53057464, used 86349896, increment 0

Initial loss: 9.212438170058329

Initial test loss: 9.212438170058329

Time since start: 683.218

500: time = 20.320000 loss = 7.196478 testLoss 7.586600

Jackknife: leave t_sand out

Making features

makeFeatures: max memory 259522560, total allocated 139407360, free 56359024, used 83048336, increment -3301560

Amomum_tsaoko_1 t_sand:

Regularization values: linear/quadratic/product: 0.385, categorical: 0.250, threshold: 1.770, hinge: 0.500

23 samples

Density: max memory 259522560, total allocated 139407360, free 35381104, used 104026256, increment 20977920

linearPredictor: max memory 259522560, total allocated 139407360, free 35381104, used 104026256, increment 0

Deactivating (awc_class=2.0)

Deactivating (awc_class=3.0)

Deactivating (awc_class=4.0)

Deactivating (awc_class=6.0)

FeaturedSpace: max memory 259522560, total allocated 139407360, free 35381104, used 104026256, increment 0

Sequential: max memory 259522560, total allocated 139407360, free 35381104, used 104026256, increment 0

Initial loss: 9.212438170058329

Initial test loss: 9.212438170058329

Time since start: 702.546

500: time = 19.007000 loss = 7.207883 testLoss 7.468054

Jackknife: only aspect

Making features

makeFeatures: max memory 259522560, total allocated 139407360, free 46206808, used 93200552, increment -10825704

Amomum_tsaoko_1 aspect:

Regularization values: linear/quadratic/product: 0.385, categorical: 0.250, threshold: 1.770, hinge: 0.500

23 samples

Density: max memory 259522560, total allocated 139407360, free 42990904, used 96416456, increment 3215904

linearPredictor: max memory 259522560, total allocated 139407360, free 42990904, used 96416456, increment 0

FeaturedSpace: max memory 259522560, total allocated 139407360, free 42990904, used 96416456, increment 0

Sequential: max memory 259522560, total allocated 139407360, free 42990904, used 96416456, increment 0

Initial loss: 9.212438170058329

Initial test loss: 9.212438170058329

Time since start: 704.112

120: time = 1.504000 loss = 9.055557 testLoss 8.965589

Res.gain: 0.15688152711631353

Jackknife: only awc_class

Making features

makeFeatures: max memory 259522560, total allocated 139407360, free 27425216, used 111982144, increment 15565688

Amomum_tsaoko_1 awc_class:

Regularization values: linear/quadratic/product: 0.385, categorical: 0.250, threshold: 1.770, hinge: 0.500

23 samples

Density: max memory 259522560, total allocated 139407360, free 27414008, used 111993352, increment 11208

linearPredictor: max memory 259522560, total allocated 139407360, free 27414008, used 111993352, increment 0

Deactivating (awc_class=2.0)

Deactivating (awc_class=3.0)

Deactivating (awc_class=4.0)

Deactivating (awc_class=6.0)

FeaturedSpace: max memory 259522560, total allocated 139407360, free 27414008, used 111993352, increment 0

Sequential: max memory 259522560, total allocated 139407360, free 27414008, used 111993352, increment 0

Initial loss: 9.212438170058329

Initial test loss: 9.212438170058329

100: time = 0.202000 loss = 9.120973 testLoss 9.125632

Res.gain: 0.09146524519810306

Jackknife: only bio_12

Making features

makeFeatures: max memory 259522560, total allocated 139407360, free 26023248, used 113384112, increment 1390760

Amomum_tsaoko_1 bio_12:

Regularization values: linear/quadratic/product: 0.385, categorical: 0.250, threshold: 1.770, hinge: 0.500

23 samples

Density: max memory 259522560, total allocated 139407360, free 24389968, used 115017392, increment 1633280

linearPredictor: max memory 259522560, total allocated 139407360, free 24389968, used 115017392, increment 0

FeaturedSpace: max memory 259522560, total allocated 139407360, free 24389968, used 115017392, increment 0

Sequential: max memory 259522560, total allocated 139407360, free 24389968, used 115017392, increment 0

Initial loss: 9.212438170058329

Initial test loss: 9.212438170058329

80: time = 0.501000 loss = 8.257979 testLoss 8.233023

Res.gain: 0.9544591257997848

Jackknife: only bio_15

Making features

makeFeatures: max memory 259522560, total allocated 139407360, free 30983064, used 108424296, increment -6593096

Amomum_tsaoko_1 bio_15:

Regularization values: linear/quadratic/product: 0.385, categorical: 0.250, threshold: 1.770, hinge: 0.500

23 samples

Density: max memory 259522560, total allocated 139407360, free 28176272, used 111231088, increment 2806792

linearPredictor: max memory 259522560, total allocated 139407360, free 28176272, used 111231088, increment 0

FeaturedSpace: max memory 259522560, total allocated 139407360, free 28176272, used 111231088, increment 0

Sequential: max memory 259522560, total allocated 139407360, free 28176272, used 111231088, increment 0

Initial loss: 9.212438170058329

Initial test loss: 9.212438170058329

Time since start: 706.133

120: time = 1.238000 loss = 8.820872 testLoss 8.439703

Res.gain: 0.39156581804456536

Jackknife: only bio_17

Making features

makeFeatures: max memory 259522560, total allocated 139407360, free 27188624, used 112218736, increment 987648

Amomum_tsaoko_1 bio_17:

Regularization values: linear/quadratic/product: 0.385, categorical: 0.250, threshold: 1.770, hinge: 0.500

23 samples

Density: max memory 259522560, total allocated 139407360, free 25597904, used 113809456, increment 1590720

linearPredictor: max memory 259522560, total allocated 139407360, free 25597904, used 113809456, increment 0

FeaturedSpace: max memory 259522560, total allocated 139407360, free 25597904, used 113809456, increment 0

Sequential: max memory 259522560, total allocated 139407360, free 25597904, used 113809456, increment 0

Initial loss: 9.212438170058329

Initial test loss: 9.212438170058329

160: time = 0.673000 loss = 8.500821 testLoss 8.142445

Res.gain: 0.711616991768425

Jackknife: only bio_4

Making features

makeFeatures: max memory 259522560, total allocated 139407360, free 16674456, used 122732904, increment 8923448

Amomum_tsaoko_1 bio_4:

Regularization values: linear/quadratic/product: 0.385, categorical: 0.250, threshold: 1.770, hinge: 0.500

23 samples

Density: max memory 259522560, total allocated 139407360, free 13543408, used 125863952, increment 3131048

linearPredictor: max memory 259522560, total allocated 139407360, free 13543408, used 125863952, increment 0

FeaturedSpace: max memory 259522560, total allocated 139407360, free 13543408, used 125863952, increment 0

Sequential: max memory 259522560, total allocated 139407360, free 13543408, used 125863952, increment 0

Initial loss: 9.212438170058329

Initial test loss: 9.212438170058329

Time since start: 708.264

140: time = 1.394000 loss = 7.863452 testLoss 7.800850

Res.gain: 1.3489857849355937

Jackknife: only bio_6

Making features

makeFeatures: max memory 259522560, total allocated 139407360, free 35513704, used 103893656, increment -21970296

Amomum_tsaoko_1 bio_6:

Regularization values: linear/quadratic/product: 0.385, categorical: 0.250, threshold: 1.770, hinge: 0.500

23 samples

Density: max memory 259522560, total allocated 139407360, free 33150112, used 106257248, increment 2363592

linearPredictor: max memory 259522560, total allocated 139407360, free 33069928, used 106337432, increment 80184

FeaturedSpace: max memory 259522560, total allocated 139407360, free 33069928, used 106337432, increment 0

Sequential: max memory 259522560, total allocated 139407360, free 33069928, used 106337432, increment 0

Initial loss: 9.212438170058329

Initial test loss: 9.212438170058329

Time since start: 710.273

220: time = 1.978000 loss = 8.112130 testLoss 8.086314

Res.gain: 1.1003084860224988

Jackknife: only elev

Making features

makeFeatures: max memory 259522560, total allocated 139407360, free 28844440, used 110562920, increment 4225488

Amomum_tsaoko_1 elev:

Regularization values: linear/quadratic/product: 0.385, categorical: 0.250, threshold: 1.770, hinge: 0.500

23 samples

Density: max memory 259522560, total allocated 139407360, free 27171240, used 112236120, increment 1673200

linearPredictor: max memory 259522560, total allocated 139407360, free 27171240, used 112236120, increment 0

FeaturedSpace: max memory 259522560, total allocated 139407360, free 27171240, used 112236120, increment 0

Sequential: max memory 259522560, total allocated 139407360, free 27171240, used 112236120, increment 0

Initial loss: 9.212438170058329

Initial test loss: 9.212438170058329

100: time = 0.642000 loss = 9.158576 testLoss 9.282380

Res.gain: 0.05386194261941846

Jackknife: only s_caco3

Making features

makeFeatures: max memory 259522560, total allocated 139407360, free 41714920, used 97692440, increment -14543680

Amomum_tsaoko_1 s_caco3:

Regularization values: linear/quadratic/product: 0.385, categorical: 0.250, threshold: 1.770, hinge: 0.500

23 samples

Density: max memory 259522560, total allocated 139407360, free 40933384, used 98473976, increment 781536

linearPredictor: max memory 259522560, total allocated 139407360, free 40933384, used 98473976, increment 0

FeaturedSpace: max memory 259522560, total allocated 139407360, free 40933384, used 98473976, increment 0

Sequential: max memory 259522560, total allocated 139407360, free 40933384, used 98473976, increment 0

Initial loss: 9.212438170058329

Initial test loss: 9.212438170058329

Time since start: 711.572

120: time = 0.579000 loss = 8.897373 testLoss 8.525222

Res.gain: 0.3150649265486791

Jackknife: only s_ph_h2o

Making features

makeFeatures: max memory 259522560, total allocated 139407360, free 38235904, used 101171456, increment 2697480

Amomum_tsaoko_1 s_ph_h2o:

Regularization values: linear/quadratic/product: 0.385, categorical: 0.250, threshold: 1.770, hinge: 0.500

23 samples

Density: max memory 259522560, total allocated 139407360, free 37333784, used 102073576, increment 902120

linearPredictor: max memory 259522560, total allocated 139407360, free 36482928, used 102924432, increment 850856

FeaturedSpace: max memory 259522560, total allocated 139407360, free 36482928, used 102924432, increment 0

Sequential: max memory 259522560, total allocated 139407360, free 36482928, used 102924432, increment 0

Initial loss: 9.212438170058329

Initial test loss: 9.212438170058329

240: time = 0.689000 loss = 8.928114 testLoss 8.552307

Res.gain: 0.28432381869502343

Jackknife: only slope

Making features

makeFeatures: max memory 259522560, total allocated 139407360, free 32478552, used 106928808, increment 4004376

Amomum_tsaoko_1 slope:

Regularization values: linear/quadratic/product: 0.385, categorical: 0.250, threshold: 1.770, hinge: 0.500

23 samples

Density: max memory 259522560, total allocated 139407360, free 30075440, used 109331920, increment 2403112

linearPredictor: max memory 259522560, total allocated 139407360, free 30075440, used 109331920, increment 0

FeaturedSpace: max memory 259522560, total allocated 139407360, free 30075440, used 109331920, increment 0

Sequential: max memory 259522560, total allocated 139407360, free 30075440, used 109331920, increment 0

Initial loss: 9.212438170058329

Initial test loss: 9.212438170058329

Time since start: 715.078

300: time = 2.754000 loss = 9.006138 testLoss 9.000562

Res.gain: 0.20630037501632437

Jackknife: only t_clay

Making features

makeFeatures: max memory 259522560, total allocated 139407360, free 49913728, used 89493632, increment -19838288

Amomum_tsaoko_1 t_clay:

Regularization values: linear/quadratic/product: 0.385, categorical: 0.250, threshold: 1.770, hinge: 0.500

23 samples

Density: max memory 259522560, total allocated 139407360, free 48199504, used 91207856, increment 1714224

linearPredictor: max memory 259522560, total allocated 139407360, free 48199504, used 91207856, increment 0

FeaturedSpace: max memory 259522560, total allocated 139407360, free 48199504, used 91207856, increment 0

Sequential: max memory 259522560, total allocated 139407360, free 48199504, used 91207856, increment 0

Initial loss: 9.212438170058329

Initial test loss: 9.212438170058329

220: time = 0.689000 loss = 8.536387 testLoss 8.713371

Res.gain: 0.6760511402019613

Jackknife: only t_oc

Making features

makeFeatures: max memory 259522560, total allocated 139407360, free 44305648, used 95101712, increment 3893856

Amomum_tsaoko_1 t_oc:

Regularization values: linear/quadratic/product: 0.385, categorical: 0.250, threshold: 1.770, hinge: 0.500

23 samples

Density: max memory 259522560, total allocated 139407360, free 42752168, used 96655192, increment 1553480

linearPredictor: max memory 259522560, total allocated 139407360, free 42752168, used 96655192, increment 0

FeaturedSpace: max memory 259522560, total allocated 139407360, free 42752168, used 96655192, increment 0

Sequential: max memory 259522560, total allocated 139407360, free 42752168, used 96655192, increment 0

Initial loss: 9.212438170058329

Initial test loss: 9.212438170058329

Time since start: 716.285

120: time = 0.457000 loss = 8.869531 testLoss 8.887851

Res.gain: 0.3429076067690193

Jackknife: only t_sand

Making features

makeFeatures: max memory 259522560, total allocated 139407360, free 39532704, used 99874656, increment 3219464

Amomum_tsaoko_1 t_sand:

Regularization values: linear/quadratic/product: 0.385, categorical: 0.250, threshold: 1.770, hinge: 0.500

23 samples

Density: max memory 259522560, total allocated 139407360, free 38553096, used 100854264, increment 979608

linearPredictor: max memory 259522560, total allocated 139407360, free 38472912, used 100934448, increment 80184

FeaturedSpace: max memory 259522560, total allocated 139407360, free 38472912, used 100934448, increment 0

Sequential: max memory 259522560, total allocated 139407360, free 38472912, used 100934448, increment 0

Initial loss: 9.212438170058329

Initial test loss: 9.212438170058329

100: time = 0.472000 loss = 8.901530 testLoss 9.256261

Res.gain: 0.3109083772355188

getSamples: max memory 259522560, total allocated 139407360, free 23273840, used 116133520, increment 15199072

Making features

makeFeatures: max memory 259522560, total allocated 139407360, free 14673616, used 124733744, increment 8600224

Amomum_tsaoko_2:

Regularization values: linear/quadratic/product: 0.385, categorical: 0.250, threshold: 1.770, hinge: 0.500

Time since start: 717.309

23 samples

Density: max memory 259522560, total allocated 139407360, free 23143536, used 116263824, increment -8469920

linearPredictor: max memory 259522560, total allocated 139407360, free 23143536, used 116263824, increment 0

Deactivating (awc_class=2.0)

Deactivating (awc_class=3.0)

Deactivating (awc_class=4.0)

Deactivating (awc_class=6.0)

FeaturedSpace: max memory 259522560, total allocated 139407360, free 23143536, used 116263824, increment 0

Sequential: max memory 259522560, total allocated 139407360, free 23143536, used 116263824, increment 0

Initial loss: 9.212438170058329

Initial test loss: 9.212438170058329

Time since start: 736.384

500: time = 19.059000 loss = 7.153939 testLoss 7.030327

Resulting gain: 2.0584992476538293

Projecting...

Writing file E:\Amomum_tsaoko\results2\Amomum_tsaoko_2.asc

Time since start: 740.431

Writing E:\Amomum_tsaoko\results2\plots\Amomum_tsaoko_2.png

Time since start: 741.843

Projecting...

Writing file E:\Amomum_tsaoko\results2\Amomum_tsaoko_2_LGM.asc

Writing file E:\Amomum_tsaoko\results2\Amomum_tsaoko_2_LGM_clamping.asc

Time since start: 746.571

Writing E:\Amomum_tsaoko\results2\plots\Amomum_tsaoko_2_LGM.png

Time since start: 748.056

Writing E:\Amomum_tsaoko\results2\plots\Amomum_tsaoko_2_LGM_clamping.png

Time since start: 749.556

Writing file E:\Amomum_tsaoko\results2\Amomum_tsaoko_2_LGM_novel.asc

Writing file E:\Amomum_tsaoko\results2\Amomum_tsaoko_2_LGM_novel_limiting.asc

Time since start: 753.839

Projecting...

Writing file E:\Amomum_tsaoko\results2\Amomum_tsaoko_2_MH.asc

Writing file E:\Amomum_tsaoko\results2\Amomum_tsaoko_2_MH_clamping.asc

Time since start: 758.574

Writing E:\Amomum_tsaoko\results2\plots\Amomum_tsaoko_2_MH.png

Time since start: 759.962

Writing E:\Amomum_tsaoko\results2\plots\Amomum_tsaoko_2_MH_clamping.png

Time since start: 761.43

Writing file E:\Amomum_tsaoko\results2\Amomum_tsaoko_2_MH_novel.asc

Writing file E:\Amomum_tsaoko\results2\Amomum_tsaoko_2_MH_novel_limiting.asc

Time since start: 765.798

Projecting...

Writing file E:\Amomum_tsaoko\results2\Amomum_tsaoko_2_SSP126-2050S.asc

Writing file E:\Amomum_tsaoko\results2\Amomum_tsaoko_2_SSP126-2050S_clamping.asc

Time since start: 769.637

Writing E:\Amomum_tsaoko\results2\plots\Amomum_tsaoko_2_SSP126-2050S.png

Time since start: 770.985

Writing E:\Amomum_tsaoko\results2\plots\Amomum_tsaoko_2_SSP126-2050S_clamping.png

Time since start: 772.301

Writing file E:\Amomum_tsaoko\results2\Amomum_tsaoko_2_SSP126-2050S_novel.asc

Writing file E:\Amomum_tsaoko\results2\Amomum_tsaoko_2_SSP126-2050S_novel_limiting.asc

Time since start: 776.92

Projecting...

Writing file E:\Amomum_tsaoko\results2\Amomum_tsaoko_2_SSP126-2090S.asc

Writing file E:\Amomum_tsaoko\results2\Amomum_tsaoko_2_SSP126-2090S_clamping.asc

Time since start: 781.088

Writing E:\Amomum_tsaoko\results2\plots\Amomum_tsaoko_2_SSP126-2090S.png

Time since start: 782.544

Writing E:\Amomum_tsaoko\results2\plots\Amomum_tsaoko_2_SSP126-2090S_clamping.png

Time since start: 783.873

Writing file E:\Amomum_tsaoko\results2\Amomum_tsaoko_2_SSP126-2090S_novel.asc

Writing file E:\Amomum_tsaoko\results2\Amomum_tsaoko_2_SSP126-2090S_novel_limiting.asc

Time since start: 788.385

Projecting...

Writing file E:\Amomum_tsaoko\results2\Amomum_tsaoko_2_SSP585-2050S.asc

Writing file E:\Amomum_tsaoko\results2\Amomum_tsaoko_2_SSP585-2050S_clamping.asc

Time since start: 792.659

Writing E:\Amomum_tsaoko\results2\plots\Amomum_tsaoko_2_SSP585-2050S.png

Time since start: 794.082

Writing E:\Amomum_tsaoko\results2\plots\Amomum_tsaoko_2_SSP585-2050S_clamping.png

Time since start: 795.443

Writing file E:\Amomum_tsaoko\results2\Amomum_tsaoko_2_SSP585-2050S_novel.asc

Writing file E:\Amomum_tsaoko\results2\Amomum_tsaoko_2_SSP585-2050S_novel_limiting.asc

Time since start: 800.403

Projecting...

Writing file E:\Amomum_tsaoko\results2\Amomum_tsaoko_2_SSP585-2090S.asc

Writing file E:\Amomum_tsaoko\results2\Amomum_tsaoko_2_SSP585-2090S_clamping.asc

Time since start: 804.529

Writing E:\Amomum_tsaoko\results2\plots\Amomum_tsaoko_2_SSP585-2090S.png

Time since start: 805.91

Writing E:\Amomum_tsaoko\results2\plots\Amomum_tsaoko_2_SSP585-2090S_clamping.png

Time since start: 807.276

Writing file E:\Amomum_tsaoko\results2\Amomum_tsaoko_2_SSP585-2090S_novel.asc

Writing file E:\Amomum_tsaoko\results2\Amomum_tsaoko_2_SSP585-2090S_novel_limiting.asc

Time since start: 812.036

Amomum_tsaoko_2 response curves

Time since start: 813.053

Response curve: only aspect

Making features

makeFeatures: max memory 259522560, total allocated 139407360, free 42684288, used 96723072, increment -19540752

Amomum_tsaoko_2 aspect:

Regularization values: linear/quadratic/product: 0.385, categorical: 0.250, threshold: 1.770, hinge: 0.500

23 samples

Density: max memory 259522560, total allocated 139407360, free 40378024, used 99029336, increment 2306264

linearPredictor: max memory 259522560, total allocated 139407360, free 40297840, used 99109520, increment 80184

FeaturedSpace: max memory 259522560, total allocated 139407360, free 40297840, used 99109520, increment 0

Sequential: max memory 259522560, total allocated 139407360, free 40297840, used 99109520, increment 0

Initial loss: 9.212438170058329

Time since start: 814.321

100: time = 1.237000 loss = 9.105401

Resulting gain: 0.10703684994142826

Amomum_tsaoko_2 response curves

Response curve: only awc_class

Making features

makeFeatures: max memory 259522560, total allocated 139407360, free 54273280, used 85134080, increment -13975440

Amomum_tsaoko_2 awc_class:

Regularization values: linear/quadratic/product: 0.385, categorical: 0.250, threshold: 1.770, hinge: 0.500

23 samples

Density: max memory 259522560, total allocated 139407360, free 54260896, used 85146464, increment 12384

linearPredictor: max memory 259522560, total allocated 139407360, free 54260896, used 85146464, increment 0

Deactivating (awc_class=2.0)

Deactivating (awc_class=3.0)

Deactivating (awc_class=4.0)

Deactivating (awc_class=6.0)

FeaturedSpace: max memory 259522560, total allocated 139407360, free 54260896, used 85146464, increment 0

Sequential: max memory 259522560, total allocated 139407360, free 54260896, used 85146464, increment 0

Initial loss: 9.212438170058329

80: time = 0.141000 loss = 9.058521

Resulting gain: 0.15391686332801235

Amomum_tsaoko_2 response curves

Response curve: only bio_12

Making features

makeFeatures: max memory 259522560, total allocated 139407360, free 49642008, used 89765352, increment 4618888

Amomum_tsaoko_2 bio_12:

Regularization values: linear/quadratic/product: 0.385, categorical: 0.250, threshold: 1.770, hinge: 0.500

23 samples

Density: max memory 259522560, total allocated 139407360, free 48092488, used 91314872, increment 1549520

linearPredictor: max memory 259522560, total allocated 139407360, free 48092488, used 91314872, increment 0

FeaturedSpace: max memory 259522560, total allocated 139407360, free 48092488, used 91314872, increment 0

Sequential: max memory 259522560, total allocated 139407360, free 48092488, used 91314872, increment 0

Initial loss: 9.212438170058329

140: time = 0.673000 loss = 8.262200

Resulting gain: 0.9502380427164248

Amomum_tsaoko_2 response curves

Time since start: 815.369

Response curve: only bio_15

Making features

makeFeatures: max memory 259522560, total allocated 139407360, free 66337536, used 73069824, increment -18245048

Amomum_tsaoko_2 bio_15:

Regularization values: linear/quadratic/product: 0.385, categorical: 0.250, threshold: 1.770, hinge: 0.500

23 samples

Density: max memory 259522560, total allocated 139407360, free 63436152, used 75971208, increment 2901384

linearPredictor: max memory 259522560, total allocated 139407360, free 63436152, used 75971208, increment 0

FeaturedSpace: max memory 259522560, total allocated 139407360, free 63436152, used 75971208, increment 0

Sequential: max memory 259522560, total allocated 139407360, free 63436152, used 75971208, increment 0

Initial loss: 9.212438170058329

Time since start: 818.373

320: time = 2.988000 loss = 8.866553

Resulting gain: 0.34588516718227424

Amomum_tsaoko_2 response curves

Response curve: only bio_17

Making features

makeFeatures: max memory 259522560, total allocated 139407360, free 60205384, used 79201976, increment 3230768

Amomum_tsaoko_2 bio_17:

Regularization values: linear/quadratic/product: 0.385, categorical: 0.250, threshold: 1.770, hinge: 0.500

23 samples

Density: max memory 259522560, total allocated 139407360, free 59400760, used 80006600, increment 804624

linearPredictor: max memory 259522560, total allocated 139407360, free 59400760, used 80006600, increment 0

FeaturedSpace: max memory 259522560, total allocated 139407360, free 59400760, used 80006600, increment 0

Sequential: max memory 259522560, total allocated 139407360, free 59400760, used 80006600, increment 0

Initial loss: 9.212438170058329

Time since start: 819.553

300: time = 1.051000 loss = 8.217404

Resulting gain: 0.9950345984822846

Amomum_tsaoko_2 response curves

Response curve: only bio_4

Making features

makeFeatures: max memory 259522560, total allocated 139407360, free 38460736, used 100946624, increment 20940024

Amomum_tsaoko_2 bio_4:

Regularization values: linear/quadratic/product: 0.385, categorical: 0.250, threshold: 1.770, hinge: 0.500

23 samples

Density: max memory 259522560, total allocated 139407360, free 35695208, used 103712152, increment 2765528

linearPredictor: max memory 259522560, total allocated 139407360, free 35695208, used 103712152, increment 0

FeaturedSpace: max memory 259522560, total allocated 139407360, free 35695208, used 103712152, increment 0

Sequential: max memory 259522560, total allocated 139407360, free 35695208, used 103712152, increment 0

Initial loss: 9.212438170058329

Time since start: 821.413

180: time = 1.752000 loss = 7.927271

Resulting gain: 1.2851669583644414

Amomum_tsaoko_2 response curves

Response curve: only bio_6

Making features

makeFeatures: max memory 259522560, total allocated 139407360, free 50302048, used 89105312, increment -14606840

Amomum_tsaoko_2 bio_6:

Regularization values: linear/quadratic/product: 0.385, categorical: 0.250, threshold: 1.770, hinge: 0.500

23 samples

Density: max memory 259522560, total allocated 139407360, free 47859472, used 91547888, increment 2442576

linearPredictor: max memory 259522560, total allocated 139407360, free 47859472, used 91547888, increment 0

FeaturedSpace: max memory 259522560, total allocated 139407360, free 47859472, used 91547888, increment 0

Sequential: max memory 259522560, total allocated 139407360, free 47859472, used 91547888, increment 0

Initial loss: 9.212438170058329

Time since start: 823.291

200: time = 1.769000 loss = 8.128740

Resulting gain: 1.0836986098710124

Amomum_tsaoko_2 response curves

Response curve: only elev

Making features

makeFeatures: max memory 259522560, total allocated 139407360, free 63713960, used 75693400, increment -15854488

Amomum_tsaoko_2 elev:

Regularization values: linear/quadratic/product: 0.385, categorical: 0.250, threshold: 1.770, hinge: 0.500

23 samples

Density: max memory 259522560, total allocated 139407360, free 62076936, used 77330424, increment 1637024

linearPredictor: max memory 259522560, total allocated 139407360, free 62076936, used 77330424, increment 0

FeaturedSpace: max memory 259522560, total allocated 139407360, free 62076936, used 77330424, increment 0

Sequential: max memory 259522560, total allocated 139407360, free 62076936, used 77330424, increment 0

Initial loss: 9.212438170058329

60: time = 0.422000 loss = 9.179090

Resulting gain: 0.03334824488017318

Amomum_tsaoko_2 response curves

Response curve: only s_caco3

Making features

makeFeatures: max memory 259522560, total allocated 139407360, free 59418592, used 79988768, increment 2658344

Amomum_tsaoko_2 s_caco3:

Regularization values: linear/quadratic/product: 0.385, categorical: 0.250, threshold: 1.770, hinge: 0.500

23 samples

Density: max memory 259522560, total allocated 139407360, free 58553552, used 80853808, increment 865040

linearPredictor: max memory 259522560, total allocated 139407360, free 58473368, used 80933992, increment 80184

FeaturedSpace: max memory 259522560, total allocated 139407360, free 58473368, used 80933992, increment 0

Sequential: max memory 259522560, total allocated 139407360, free 58473368, used 80933992, increment 0

Initial loss: 9.212438170058329

Time since start: 824.777

180: time = 0.861000 loss = 8.747680

Resulting gain: 0.4647582341978769

Amomum_tsaoko_2 response curves

Response curve: only s_ph_h2o

Making features

makeFeatures: max memory 259522560, total allocated 139407360, free 50720208, used 88687152, increment 7753160

Amomum_tsaoko_2 s_ph_h2o:

Regularization values: linear/quadratic/product: 0.385, categorical: 0.250, threshold: 1.770, hinge: 0.500

23 samples

Density: max memory 259522560, total allocated 139407360, free 49940192, used 89467168, increment 780016

linearPredictor: max memory 259522560, total allocated 139407360, free 49940192, used 89467168, increment 0

FeaturedSpace: max memory 259522560, total allocated 139407360, free 49940192, used 89467168, increment 0

Sequential: max memory 259522560, total allocated 139407360, free 49940192, used 89467168, increment 0

Initial loss: 9.212438170058329

180: time = 0.720000 loss = 8.647498

Resulting gain: 0.5649406427673185

Amomum_tsaoko_2 response curves

Response curve: only slope

Making features

makeFeatures: max memory 259522560, total allocated 139407360, free 42263472, used 97143888, increment 7676720

Amomum_tsaoko_2 slope:

Regularization values: linear/quadratic/product: 0.385, categorical: 0.250, threshold: 1.770, hinge: 0.500

23 samples

Density: max memory 259522560, total allocated 139407360, free 40313784, used 99093576, increment 1949688

linearPredictor: max memory 259522560, total allocated 139407360, free 40313784, used 99093576, increment 0

FeaturedSpace: max memory 259522560, total allocated 139407360, free 40313784, used 99093576, increment 0

Sequential: max memory 259522560, total allocated 139407360, free 40313784, used 99093576, increment 0

Initial loss: 9.212438170058329

Time since start: 827.328

180: time = 1.628000 loss = 9.106161

Resulting gain: 0.1062776429899106

Amomum_tsaoko_2 response curves

Response curve: only t_clay

Making features

makeFeatures: max memory 259522560, total allocated 139407360, free 47499240, used 91908120, increment -7185456

Amomum_tsaoko_2 t_clay:

Regularization values: linear/quadratic/product: 0.385, categorical: 0.250, threshold: 1.770, hinge: 0.500

23 samples

Density: max memory 259522560, total allocated 139407360, free 46696376, used 92710984, increment 802864

linearPredictor: max memory 259522560, total allocated 139407360, free 46696376, used 92710984, increment 0

FeaturedSpace: max memory 259522560, total allocated 139407360, free 46696376, used 92710984, increment 0

Sequential: max memory 259522560, total allocated 139407360, free 46696376, used 92710984, increment 0

Initial loss: 9.212438170058329

100: time = 0.438000 loss = 8.666677

Resulting gain: 0.5457614823845205

Amomum_tsaoko_2 response curves

Response curve: only t_oc

Making features

makeFeatures: max memory 259522560, total allocated 139407360, free 40434320, used 98973040, increment 6262056

Amomum_tsaoko_2 t_oc:

Regularization values: linear/quadratic/product: 0.385, categorical: 0.250, threshold: 1.770, hinge: 0.500

23 samples

Density: max memory 259522560, total allocated 139407360, free 39531120, used 99876240, increment 903200

linearPredictor: max memory 259522560, total allocated 139407360, free 39531120, used 99876240, increment 0

FeaturedSpace: max memory 259522560, total allocated 139407360, free 39531120, used 99876240, increment 0

Sequential: max memory 259522560, total allocated 139407360, free 39531120, used 99876240, increment 0

Initial loss: 9.212438170058329

Time since start: 828.491

160: time = 0.554000 loss = 8.772402

Resulting gain: 0.440036484113584

Amomum_tsaoko_2 response curves

Response curve: only t_sand

Making features

makeFeatures: max memory 259522560, total allocated 139407360, free 68843176, used 70564184, increment -29312056

Amomum_tsaoko_2 t_sand:

Regularization values: linear/quadratic/product: 0.385, categorical: 0.250, threshold: 1.770, hinge: 0.500

23 samples

Density: max memory 259522560, total allocated 139407360, free 68074696, used 71332664, increment 768480

linearPredictor: max memory 259522560, total allocated 139407360, free 67994512, used 71412848, increment 80184

FeaturedSpace: max memory 259522560, total allocated 139407360, free 67994512, used 71412848, increment 0

Sequential: max memory 259522560, total allocated 139407360, free 67994512, used 71412848, increment 0

Initial loss: 9.212438170058329

120: time = 0.536000 loss = 9.077234

Resulting gain: 0.1352045375248636

Amomum_tsaoko_2 response curves

Time since start: 830.381

Jackknife: leave aspect out

Making features

makeFeatures: max memory 259522560, total allocated 139407360, free 51520096, used 87887264, increment 16474416

Amomum_tsaoko_2 aspect:

Regularization values: linear/quadratic/product: 0.385, categorical: 0.250, threshold: 1.770, hinge: 0.500

23 samples

Density: max memory 259522560, total allocated 139407360, free 58788112, used 80619248, increment -7268016

linearPredictor: max memory 259522560, total allocated 139407360, free 58788112, used 80619248, increment 0

Deactivating (awc_class=2.0)

Deactivating (awc_class=3.0)

Deactivating (awc_class=4.0)

Deactivating (awc_class=6.0)

FeaturedSpace: max memory 259522560, total allocated 139407360, free 58788112, used 80619248, increment 0

Sequential: max memory 259522560, total allocated 139407360, free 58788112, used 80619248, increment 0

Initial loss: 9.212438170058329

Initial test loss: 9.212438170058329

Time since start: 846.248

500: time = 15.600000 loss = 7.276622 testLoss 6.939947

Jackknife: leave awc_class out

Making features

makeFeatures: max memory 259522560, total allocated 139407360, free 50846544, used 88560816, increment 7941568

Amomum_tsaoko_2 awc_class:

Regularization values: linear/quadratic/product: 0.385, categorical: 0.250, threshold: 1.770, hinge: 0.500

23 samples

Density: max memory 259522560, total allocated 139407360, free 29384312, used 110023048, increment 21462232

linearPredictor: max memory 259522560, total allocated 139407360, free 29384312, used 110023048, increment 0

FeaturedSpace: max memory 259522560, total allocated 139407360, free 29384312, used 110023048, increment 0

Sequential: max memory 259522560, total allocated 139407360, free 29384312, used 110023048, increment 0

Initial loss: 9.212438170058329

Initial test loss: 9.212438170058329

Time since start: 865.635

500: time = 19.074000 loss = 7.156168 testLoss 7.006156

Jackknife: leave bio_12 out

Making features

makeFeatures: max memory 259522560, total allocated 139407360, free 79607680, used 59799680, increment -50223368

Amomum_tsaoko_2 bio_12:

Regularization values: linear/quadratic/product: 0.385, categorical: 0.250, threshold: 1.770, hinge: 0.500

23 samples

Density: max memory 259522560, total allocated 139407360, free 59502328, used 79905032, increment 20105352

linearPredictor: max memory 259522560, total allocated 139407360, free 59502328, used 79905032, increment 0

Deactivating (awc_class=2.0)

Deactivating (awc_class=3.0)

Deactivating (awc_class=4.0)

Deactivating (awc_class=6.0)

FeaturedSpace: max memory 259522560, total allocated 139407360, free 59502328, used 79905032, increment 0

Sequential: max memory 259522560, total allocated 139407360, free 59502328, used 79905032, increment 0

Initial loss: 9.212438170058329

Initial test loss: 9.212438170058329

Time since start: 884.207

500: time = 18.260000 loss = 7.166393 testLoss 7.126884

Jackknife: leave bio_15 out

Making features

makeFeatures: max memory 259522560, total allocated 139407360, free 43062544, used 96344816, increment 16439784

Amomum_tsaoko_2 bio_15:

Regularization values: linear/quadratic/product: 0.385, categorical: 0.250, threshold: 1.770, hinge: 0.500

23 samples

Density: max memory 259522560, total allocated 139407360, free 54259440, used 85147920, increment -11196896

linearPredictor: max memory 259522560, total allocated 139407360, free 54259440, used 85147920, increment 0

Deactivating (awc_class=2.0)

Deactivating (awc_class=3.0)

Deactivating (awc_class=4.0)

Deactivating (awc_class=6.0)

FeaturedSpace: max memory 259522560, total allocated 139407360, free 54259440, used 85147920, increment 0

Sequential: max memory 259522560, total allocated 139407360, free 54259440, used 85147920, increment 0

Initial loss: 9.212438170058329

Initial test loss: 9.212438170058329

Time since start: 900.397

500: time = 15.892000 loss = 7.194293 testLoss 6.999847

Jackknife: leave bio_17 out

Making features

makeFeatures: max memory 259522560, total allocated 139407360, free 49020296, used 90387064, increment 5239144

Amomum_tsaoko_2 bio_17:

Regularization values: linear/quadratic/product: 0.385, categorical: 0.250, threshold: 1.770, hinge: 0.500

23 samples

Density: max memory 259522560, total allocated 139407360, free 28290864, used 111116496, increment 20729432

linearPredictor: max memory 259522560, total allocated 139407360, free 28290864, used 111116496, increment 0

Deactivating (awc_class=2.0)

Deactivating (awc_class=3.0)

Deactivating (awc_class=4.0)

Deactivating (awc_class=6.0)

FeaturedSpace: max memory 259522560, total allocated 139407360, free 28290864, used 111116496, increment 0

Sequential: max memory 259522560, total allocated 139407360, free 28290864, used 111116496, increment 0

Initial loss: 9.212438170058329

Initial test loss: 9.212438170058329

Time since start: 919.803

500: time = 19.094000 loss = 7.197523 testLoss 7.194585

Jackknife: leave bio_4 out

Making features

makeFeatures: max memory 259522560, total allocated 139407360, free 42480200, used 96927160, increment -14189336

Amomum_tsaoko_2 bio_4:

Regularization values: linear/quadratic/product: 0.385, categorical: 0.250, threshold: 1.770, hinge: 0.500

23 samples

Density: max memory 259522560, total allocated 139407360, free 23889632, used 115517728, increment 18590568

linearPredictor: max memory 259522560, total allocated 139407360, free 23889632, used 115517728, increment 0

Deactivating (awc_class=2.0)

Deactivating (awc_class=3.0)

Deactivating (awc_class=4.0)

Deactivating (awc_class=6.0)

FeaturedSpace: max memory 259522560, total allocated 139407360, free 23889632, used 115517728, increment 0

Sequential: max memory 259522560, total allocated 139407360, free 23889632, used 115517728, increment 0

Initial loss: 9.212438170058329

Initial test loss: 9.212438170058329

Time since start: 935.488

480: time = 15.387000 loss = 7.209412 testLoss 7.066235

Jackknife: leave bio_6 out

Making features

makeFeatures: max memory 259522560, total allocated 139407360, free 68546776, used 70860584, increment -44657144

Amomum_tsaoko_2 bio_6:

Regularization values: linear/quadratic/product: 0.385, categorical: 0.250, threshold: 1.770, hinge: 0.500

23 samples

Density: max memory 259522560, total allocated 139407360, free 74119768, used 65287592, increment -5572992

linearPredictor: max memory 259522560, total allocated 139407360, free 74119768, used 65287592, increment 0

Deactivating (awc_class=2.0)

Deactivating (awc_class=3.0)

Deactivating (awc_class=4.0)

Deactivating (awc_class=6.0)

FeaturedSpace: max memory 259522560, total allocated 139407360, free 74119768, used 65287592, increment 0

Sequential: max memory 259522560, total allocated 139407360, free 74119768, used 65287592, increment 0

Initial loss: 9.212438170058329

Initial test loss: 9.212438170058329

Time since start: 952.811

500: time = 17.024000 loss = 7.184964 testLoss 7.259150

Jackknife: leave elev out

Making features

makeFeatures: max memory 259522560, total allocated 139407360, free 74338344, used 65069016, increment -218576

Amomum_tsaoko_2 elev:

Regularization values: linear/quadratic/product: 0.385, categorical: 0.250, threshold: 1.770, hinge: 0.500

23 samples

Density: max memory 259522560, total allocated 139407360, free 54775056, used 84632304, increment 19563288

linearPredictor: max memory 259522560, total allocated 139407360, free 54694872, used 84712488, increment 80184

Deactivating (awc_class=2.0)

Deactivating (awc_class=3.0)

Deactivating (awc_class=4.0)

Deactivating (awc_class=6.0)

FeaturedSpace: max memory 259522560, total allocated 139407360, free 54694872, used 84712488, increment 0

Sequential: max memory 259522560, total allocated 139407360, free 54694872, used 84712488, increment 0

Initial loss: 9.212438170058329

Initial test loss: 9.212438170058329

Time since start: 971.001

500: time = 17.919000 loss = 7.162540 testLoss 7.058995

Jackknife: leave s_caco3 out

Making features

makeFeatures: max memory 259522560, total allocated 139407360, free 50027128, used 89380232, increment 4667744

Amomum_tsaoko_2 s_caco3:

Regularization values: linear/quadratic/product: 0.385, categorical: 0.250, threshold: 1.770, hinge: 0.500

23 samples

Density: max memory 259522560, total allocated 139407360, free 29321296, used 110086064, increment 20705832

linearPredictor: max memory 259522560, total allocated 139407360, free 29321296, used 110086064, increment 0

Deactivating (awc_class=2.0)

Deactivating (awc_class=3.0)

Deactivating (awc_class=4.0)

Deactivating (awc_class=6.0)

FeaturedSpace: max memory 259522560, total allocated 139407360, free 29321296, used 110086064, increment 0

Sequential: max memory 259522560, total allocated 139407360, free 29321296, used 110086064, increment 0

Initial loss: 9.212438170058329

Initial test loss: 9.212438170058329

Time since start: 990.941

500: time = 19.670000 loss = 7.246201 testLoss 7.086654

Jackknife: leave s_ph_h2o out

Making features

makeFeatures: max memory 259522560, total allocated 139407360, free 32816216, used 106591144, increment -3494920

Amomum_tsaoko_2 s_ph_h2o:

Regularization values: linear/quadratic/product: 0.385, categorical: 0.250, threshold: 1.770, hinge: 0.500

23 samples

Density: max memory 259522560, total allocated 139407360, free 95190096, used 44217264, increment -62373880

linearPredictor: max memory 259522560, total allocated 139407360, free 95190096, used 44217264, increment 0

Deactivating (awc_class=2.0)

Deactivating (awc_class=3.0)

Deactivating (awc_class=4.0)

Deactivating (awc_class=6.0)

FeaturedSpace: max memory 259522560, total allocated 139407360, free 95190096, used 44217264, increment 0

Sequential: max memory 259522560, total allocated 139407360, free 95190096, used 44217264, increment 0

Initial loss: 9.212438170058329

Initial test loss: 9.212438170058329

Time since start: 1010.526

500: time = 19.252000 loss = 7.154186 testLoss 7.019291

Jackknife: leave slope out

Making features

makeFeatures: max memory 259522560, total allocated 139407360, free 70605040, used 68802320, increment 24585056

Amomum_tsaoko_2 slope:

Regularization values: linear/quadratic/product: 0.385, categorical: 0.250, threshold: 1.770, hinge: 0.500

23 samples

Density: max memory 259522560, total allocated 139407360, free 76122920, used 63284440, increment -5517880

linearPredictor: max memory 259522560, total allocated 139407360, free 76122920, used 63284440, increment 0

Deactivating (awc_class=2.0)

Deactivating (awc_class=3.0)

Deactivating (awc_class=4.0)

Deactivating (awc_class=6.0)

FeaturedSpace: max memory 259522560, total allocated 139407360, free 76122920, used 63284440, increment 0

Sequential: max memory 259522560, total allocated 139407360, free 76122920, used 63284440, increment 0

Initial loss: 9.212438170058329

Initial test loss: 9.212438170058329

Time since start: 1027.245

500: time = 16.400000 loss = 7.229342 testLoss 7.323227

Jackknife: leave t_clay out

Making features

makeFeatures: max memory 259522560, total allocated 139407360, free 69939288, used 69468072, increment 6183632

Amomum_tsaoko_2 t_clay:

Regularization values: linear/quadratic/product: 0.385, categorical: 0.250, threshold: 1.770, hinge: 0.500

23 samples

Density: max memory 259522560, total allocated 139407360, free 49126912, used 90280448, increment 20812376

linearPredictor: max memory 259522560, total allocated 139407360, free 49126912, used 90280448, increment 0

Deactivating (awc_class=2.0)

Deactivating (awc_class=3.0)

Deactivating (awc_class=4.0)

Deactivating (awc_class=6.0)

FeaturedSpace: max memory 259522560, total allocated 139407360, free 49126912, used 90280448, increment 0

Sequential: max memory 259522560, total allocated 139407360, free 49126912, used 90280448, increment 0

Initial loss: 9.212438170058329

Initial test loss: 9.212438170058329

Time since start: 1047.873

500: time = 20.308000 loss = 7.162078 testLoss 6.989706

Jackknife: leave t_oc out

Making features

makeFeatures: max memory 259522560, total allocated 139407360, free 50460904, used 88946456, increment -1333992

Amomum_tsaoko_2 t_oc:

Regularization values: linear/quadratic/product: 0.385, categorical: 0.250, threshold: 1.770, hinge: 0.500

23 samples

Density: max memory 259522560, total allocated 139407360, free 29644592, used 109762768, increment 20816312

linearPredictor: max memory 259522560, total allocated 139407360, free 29644592, used 109762768, increment 0

Deactivating (awc_class=2.0)

Deactivating (awc_class=3.0)

Deactivating (awc_class=4.0)

Deactivating (awc_class=6.0)

FeaturedSpace: max memory 259522560, total allocated 139407360, free 29644592, used 109762768, increment 0

Sequential: max memory 259522560, total allocated 139407360, free 29644592, used 109762768, increment 0

Initial loss: 9.212438170058329

Initial test loss: 9.212438170058329

Time since start: 1068.29

500: time = 20.084000 loss = 7.169015 testLoss 6.981054

Jackknife: leave t_sand out

Making features

makeFeatures: max memory 259522560, total allocated 139407360, free 34055992, used 105351368, increment -4411400

Amomum_tsaoko_2 t_sand:

Regularization values: linear/quadratic/product: 0.385, categorical: 0.250, threshold: 1.770, hinge: 0.500

23 samples

Density: max memory 259522560, total allocated 139407360, free 13688288, used 125719072, increment 20367704

linearPredictor: max memory 259522560, total allocated 139407360, free 13688288, used 125719072, increment 0

Deactivating (awc_class=2.0)

Deactivating (awc_class=3.0)

Deactivating (awc_class=4.0)

Deactivating (awc_class=6.0)

FeaturedSpace: max memory 259522560, total allocated 139407360, free 13688288, used 125719072, increment 0

Sequential: max memory 259522560, total allocated 139407360, free 13688288, used 125719072, increment 0

Initial loss: 9.212438170058329

Initial test loss: 9.212438170058329

Time since start: 1088.205

500: time = 19.582000 loss = 7.169341 testLoss 7.005124

Jackknife: only aspect

Making features

makeFeatures: max memory 259522560, total allocated 139407360, free 84015896, used 55391464, increment -70327608

Amomum_tsaoko_2 aspect:

Regularization values: linear/quadratic/product: 0.385, categorical: 0.250, threshold: 1.770, hinge: 0.500

23 samples

Density: max memory 259522560, total allocated 139407360, free 80840208, used 58567152, increment 3175688

linearPredictor: max memory 259522560, total allocated 139407360, free 80840208, used 58567152, increment 0

FeaturedSpace: max memory 259522560, total allocated 139407360, free 80840208, used 58567152, increment 0

Sequential: max memory 259522560, total allocated 139407360, free 80840208, used 58567152, increment 0

Initial loss: 9.212438170058329

Initial test loss: 9.212438170058329

Time since start: 1089.497

100: time = 1.240000 loss = 9.105401 testLoss 9.140142

Res.gain: 0.10703684994142826

Jackknife: only awc_class

Making features

makeFeatures: max memory 259522560, total allocated 139407360, free 60496024, used 78911336, increment 20344184

Amomum_tsaoko_2 awc_class:

Regularization values: linear/quadratic/product: 0.385, categorical: 0.250, threshold: 1.770, hinge: 0.500

23 samples

Density: max memory 259522560, total allocated 139407360, free 60481816, used 78925544, increment 14208

linearPredictor: max memory 259522560, total allocated 139407360, free 60401632, used 79005728, increment 80184

Deactivating (awc_class=2.0)

Deactivating (awc_class=3.0)

Deactivating (awc_class=4.0)

Deactivating (awc_class=6.0)

FeaturedSpace: max memory 259522560, total allocated 139407360, free 60401632, used 79005728, increment 0

Sequential: max memory 259522560, total allocated 139407360, free 60401632, used 79005728, increment 0

Initial loss: 9.212438170058329

Initial test loss: 9.212438170058329

80: time = 0.144000 loss = 9.058521 testLoss 9.141183

Res.gain: 0.15391686332801235

Jackknife: only bio_12

Making features

makeFeatures: max memory 259522560, total allocated 139407360, free 59046968, used 80360392, increment 1354664

Amomum_tsaoko_2 bio_12:

Regularization values: linear/quadratic/product: 0.385, categorical: 0.250, threshold: 1.770, hinge: 0.500

23 samples

Density: max memory 259522560, total allocated 139407360, free 57373296, used 82034064, increment 1673672

linearPredictor: max memory 259522560, total allocated 139407360, free 57373296, used 82034064, increment 0

FeaturedSpace: max memory 259522560, total allocated 139407360, free 57373296, used 82034064, increment 0

Sequential: max memory 259522560, total allocated 139407360, free 57373296, used 82034064, increment 0

Initial loss: 9.212438170058329

Initial test loss: 9.212438170058329

140: time = 0.696000 loss = 8.262200 testLoss 8.186662

Res.gain: 0.9502380427164248

Jackknife: only bio_15

Making features

makeFeatures: max memory 259522560, total allocated 139407360, free 79220232, used 60187128, increment -21846936

Amomum_tsaoko_2 bio_15:

Regularization values: linear/quadratic/product: 0.385, categorical: 0.250, threshold: 1.770, hinge: 0.500

23 samples

Density: max memory 259522560, total allocated 139407360, free 76018216, used 63389144, increment 3202016

linearPredictor: max memory 259522560, total allocated 139407360, free 76018216, used 63389144, increment 0

FeaturedSpace: max memory 259522560, total allocated 139407360, free 76018216, used 63389144, increment 0

Sequential: max memory 259522560, total allocated 139407360, free 76018216, used 63389144, increment 0

Initial loss: 9.212438170058329

Initial test loss: 9.212438170058329

Time since start: 1093.394

320: time = 2.981000 loss = 8.866553 testLoss 8.686143

Res.gain: 0.34588516718227424

Jackknife: only bio_17

Making features

makeFeatures: max memory 259522560, total allocated 139407360, free 76590160, used 62817200, increment -571944

Amomum_tsaoko_2 bio_17:

Regularization values: linear/quadratic/product: 0.385, categorical: 0.250, threshold: 1.770, hinge: 0.500

23 samples

Density: max memory 259522560, total allocated 139407360, free 75808880, used 63598480, increment 781280

linearPredictor: max memory 259522560, total allocated 139407360, free 75038184, used 64369176, increment 770696

FeaturedSpace: max memory 259522560, total allocated 139407360, free 75038184, used 64369176, increment 0

Sequential: max memory 259522560, total allocated 139407360, free 75038184, used 64369176, increment 0

Initial loss: 9.212438170058329

Initial test loss: 9.212438170058329

Time since start: 1094.483

300: time = 1.061000 loss = 8.217404 testLoss 8.266762

Res.gain: 0.9950345984822846

Jackknife: only bio_4

Making features

makeFeatures: max memory 259522560, total allocated 139407360, free 58842848, used 80564512, increment 16195336

Amomum_tsaoko_2 bio_4:

Regularization values: linear/quadratic/product: 0.385, categorical: 0.250, threshold: 1.770, hinge: 0.500

23 samples

Density: max memory 259522560, total allocated 139407360, free 92314184, used 47093176, increment -33471336

linearPredictor: max memory 259522560, total allocated 139407360, free 92314184, used 47093176, increment 0

FeaturedSpace: max memory 259522560, total allocated 139407360, free 92314184, used 47093176, increment 0

Sequential: max memory 259522560, total allocated 139407360, free 92314184, used 47093176, increment 0

Initial loss: 9.212438170058329

Initial test loss: 9.212438170058329

Time since start: 1096.266

180: time = 1.759000 loss = 7.927271 testLoss 7.835200

Res.gain: 1.2851669583644414

Jackknife: only bio_6

Making features

makeFeatures: max memory 259522560, total allocated 139407360, free 74383536, used 65023824, increment 17930648

Amomum_tsaoko_2 bio_6:

Regularization values: linear/quadratic/product: 0.385, categorical: 0.250, threshold: 1.770, hinge: 0.500

23 samples

Density: max memory 259522560, total allocated 139407360, free 72021272, used 67386088, increment 2362264

linearPredictor: max memory 259522560, total allocated 139407360, free 71941088, used 67466272, increment 80184

FeaturedSpace: max memory 259522560, total allocated 139407360, free 71941088, used 67466272, increment 0

Sequential: max memory 259522560, total allocated 139407360, free 71941088, used 67466272, increment 0

Initial loss: 9.212438170058329

Initial test loss: 9.212438170058329

Time since start: 1098.059

200: time = 1.761000 loss = 8.128740 testLoss 7.896867

Res.gain: 1.0836986098710124

Jackknife: only elev

Making features

makeFeatures: max memory 259522560, total allocated 139407360, free 91476032, used 47931328, increment -19534944

Amomum_tsaoko_2 elev:

Regularization values: linear/quadratic/product: 0.385, categorical: 0.250, threshold: 1.770, hinge: 0.500

23 samples

Density: max memory 259522560, total allocated 139407360, free 89842200, used 49565160, increment 1633832

linearPredictor: max memory 259522560, total allocated 139407360, free 89842200, used 49565160, increment 0

FeaturedSpace: max memory 259522560, total allocated 139407360, free 89842200, used 49565160, increment 0

Sequential: max memory 259522560, total allocated 139407360, free 89842200, used 49565160, increment 0

Initial loss: 9.212438170058329

Initial test loss: 9.212438170058329

60: time = 0.452000 loss = 9.179090 testLoss 9.128254

Res.gain: 0.03334824488017318

Jackknife: only s_caco3

Making features

makeFeatures: max memory 259522560, total allocated 139407360, free 90829496, used 48577864, increment -987296

Amomum_tsaoko_2 s_caco3:

Regularization values: linear/quadratic/product: 0.385, categorical: 0.250, threshold: 1.770, hinge: 0.500

23 samples

Density: max memory 259522560, total allocated 139407360, free 89929096, used 49478264, increment 900400

linearPredictor: max memory 259522560, total allocated 139407360, free 89848912, used 49558448, increment 80184

FeaturedSpace: max memory 259522560, total allocated 139407360, free 89848912, used 49558448, increment 0

Sequential: max memory 259522560, total allocated 139407360, free 89848912, used 49558448, increment 0

Initial loss: 9.212438170058329

Initial test loss: 9.212438170058329

Time since start: 1099.477

180: time = 0.898000 loss = 8.747680 testLoss 8.543654

Res.gain: 0.4647582341978769

Jackknife: only s_ph_h2o

Making features

makeFeatures: max memory 259522560, total allocated 139407360, free 85968728, used 53438632, increment 3880184

Amomum_tsaoko_2 s_ph_h2o:

Regularization values: linear/quadratic/product: 0.385, categorical: 0.250, threshold: 1.770, hinge: 0.500

23 samples

Density: max memory 259522560, total allocated 139407360, free 84337640, used 55069720, increment 1631088

linearPredictor: max memory 259522560, total allocated 139407360, free 84337640, used 55069720, increment 0

FeaturedSpace: max memory 259522560, total allocated 139407360, free 84337640, used 55069720, increment 0

Sequential: max memory 259522560, total allocated 139407360, free 84337640, used 55069720, increment 0

Initial loss: 9.212438170058329

Initial test loss: 9.212438170058329

180: time = 0.734000 loss = 8.647498 testLoss 8.504098

Res.gain: 0.5649406427673185

Jackknife: only slope

Making features

makeFeatures: max memory 259522560, total allocated 139407360, free 80707416, used 58699944, increment 3630224

Amomum_tsaoko_2 slope:

Regularization values: linear/quadratic/product: 0.385, categorical: 0.250, threshold: 1.770, hinge: 0.500

23 samples

Density: max memory 259522560, total allocated 139407360, free 78333464, used 61073896, increment 2373952

linearPredictor: max memory 259522560, total allocated 139407360, free 78333464, used 61073896, increment 0

FeaturedSpace: max memory 259522560, total allocated 139407360, free 78333464, used 61073896, increment 0

Sequential: max memory 259522560, total allocated 139407360, free 78333464, used 61073896, increment 0

Initial loss: 9.212438170058329

Initial test loss: 9.212438170058329

Time since start: 1101.916

180: time = 1.650000 loss = 9.106161 testLoss 9.235317

Res.gain: 0.1062776429899106

Jackknife: only t_clay

Making features

makeFeatures: max memory 259522560, total allocated 139407360, free 90113720, used 49293640, increment -11780256

Amomum_tsaoko_2 t_clay:

Regularization values: linear/quadratic/product: 0.385, categorical: 0.250, threshold: 1.770, hinge: 0.500

23 samples

Density: max memory 259522560, total allocated 139407360, free 89323232, used 50084128, increment 790488

linearPredictor: max memory 259522560, total allocated 139407360, free 89323232, used 50084128, increment 0

FeaturedSpace: max memory 259522560, total allocated 139407360, free 89323232, used 50084128, increment 0

Sequential: max memory 259522560, total allocated 139407360, free 89323232, used 50084128, increment 0

Initial loss: 9.212438170058329

Initial test loss: 9.212438170058329

100: time = 0.449000 loss = 8.666677 testLoss 9.001593

Res.gain: 0.5457614823845205

Jackknife: only t_oc

Making features

makeFeatures: max memory 259522560, total allocated 139407360, free 87021608, used 52385752, increment 2301624

Amomum_tsaoko_2 t_oc:

Regularization values: linear/quadratic/product: 0.385, categorical: 0.250, threshold: 1.770, hinge: 0.500

23 samples

Density: max memory 259522560, total allocated 139407360, free 86038768, used 53368592, increment 982840

linearPredictor: max memory 259522560, total allocated 139407360, free 85958584, used 53448776, increment 80184

FeaturedSpace: max memory 259522560, total allocated 139407360, free 85958584, used 53448776, increment 0

Sequential: max memory 259522560, total allocated 139407360, free 85958584, used 53448776, increment 0

Initial loss: 9.212438170058329

Initial test loss: 9.212438170058329

Time since start: 1102.992

160: time = 0.562000 loss = 8.772402 testLoss 9.094122

Res.gain: 0.440036484113584

Jackknife: only t_sand

Making features

makeFeatures: max memory 259522560, total allocated 139407360, free 80998464, used 58408896, increment 4960120

Amomum_tsaoko_2 t_sand:

Regularization values: linear/quadratic/product: 0.385, categorical: 0.250, threshold: 1.770, hinge: 0.500

23 samples

Density: max memory 259522560, total allocated 139407360, free 80216184, used 59191176, increment 782280

linearPredictor: max memory 259522560, total allocated 139407360, free 79446760, used 59960600, increment 769424

FeaturedSpace: max memory 259522560, total allocated 139407360, free 79446760, used 59960600, increment 0

Sequential: max memory 259522560, total allocated 139407360, free 79446760, used 59960600, increment 0

Initial loss: 9.212438170058329

Initial test loss: 9.212438170058329

120: time = 0.528000 loss = 9.077234 testLoss 9.124578

Res.gain: 0.1352045375248636

getSamples: max memory 259522560, total allocated 139407360, free 64864720, used 74542640, increment 14582040

Making features

makeFeatures: max memory 259522560, total allocated 139407360, free 55830368, used 83576992, increment 9034352

Amomum_tsaoko_3:

Regularization values: linear/quadratic/product: 0.385, categorical: 0.250, threshold: 1.770, hinge: 0.500

Time since start: 1104.078

23 samples

Density: max memory 259522560, total allocated 139407360, free 65538832, used 73868528, increment -9708464

linearPredictor: max memory 259522560, total allocated 139407360, free 65538832, used 73868528, increment 0

Deactivating (awc_class=2.0)

Deactivating (awc_class=3.0)

Deactivating (awc_class=4.0)

Deactivating (awc_class=6.0)

FeaturedSpace: max memory 259522560, total allocated 139407360, free 65538832, used 73868528, increment 0

Sequential: max memory 259522560, total allocated 139407360, free 65538832, used 73868528, increment 0

Initial loss: 9.212438170058329

Initial test loss: 9.212438170058329

Time since start: 1123.337

500: time = 19.230000 loss = 6.343500 testLoss 6.694492

Resulting gain: 2.86893859492187

Projecting...

Writing file E:\Amomum_tsaoko\results2\Amomum_tsaoko_3.asc

Time since start: 1127.053

Writing E:\Amomum_tsaoko\results2\plots\Amomum_tsaoko_3.png

Time since start: 1128.323

Projecting...

Writing file E:\Amomum_tsaoko\results2\Amomum_tsaoko_3_LGM.asc

Writing file E:\Amomum_tsaoko\results2\Amomum_tsaoko_3_LGM_clamping.asc

Time since start: 1132.84

Writing E:\Amomum_tsaoko\results2\plots\Amomum_tsaoko_3_LGM.png

Time since start: 1134.255

Writing E:\Amomum_tsaoko\results2\plots\Amomum_tsaoko_3_LGM_clamping.png

Time since start: 1135.788

Writing file E:\Amomum_tsaoko\results2\Amomum_tsaoko_3_LGM_novel.asc

Writing file E:\Amomum_tsaoko\results2\Amomum_tsaoko_3_LGM_novel_limiting.asc

Time since start: 1140.257

Projecting...

Writing file E:\Amomum_tsaoko\results2\Amomum_tsaoko_3_MH.asc

Writing file E:\Amomum_tsaoko\results2\Amomum_tsaoko_3_MH_clamping.asc

Time since start: 1144.955

Writing E:\Amomum_tsaoko\results2\plots\Amomum_tsaoko_3_MH.png

Time since start: 1146.351

Writing E:\Amomum_tsaoko\results2\plots\Amomum_tsaoko_3_MH_clamping.png

Time since start: 1147.845

Writing file E:\Amomum_tsaoko\results2\Amomum_tsaoko_3_MH_novel.asc

Writing file E:\Amomum_tsaoko\results2\Amomum_tsaoko_3_MH_novel_limiting.asc

Time since start: 1152.504

Projecting...

Writing file E:\Amomum_tsaoko\results2\Amomum_tsaoko_3_SSP126-2050S.asc

Writing file E:\Amomum_tsaoko\results2\Amomum_tsaoko_3_SSP126-2050S_clamping.asc

Time since start: 1156.178

Writing E:\Amomum_tsaoko\results2\plots\Amomum_tsaoko_3_SSP126-2050S.png

Time since start: 1157.505

Writing E:\Amomum_tsaoko\results2\plots\Amomum_tsaoko_3_SSP126-2050S_clamping.png

Time since start: 1158.955

Writing file E:\Amomum_tsaoko\results2\Amomum_tsaoko_3_SSP126-2050S_novel.asc

Writing file E:\Amomum_tsaoko\results2\Amomum_tsaoko_3_SSP126-2050S_novel_limiting.asc

Time since start: 1163.636

Projecting...

Writing file E:\Amomum_tsaoko\results2\Amomum_tsaoko_3_SSP126-2090S.asc

Writing file E:\Amomum_tsaoko\results2\Amomum_tsaoko_3_SSP126-2090S_clamping.asc

Time since start: 1167.229

Writing E:\Amomum_tsaoko\results2\plots\Amomum_tsaoko_3_SSP126-2090S.png

Time since start: 1168.51

Writing E:\Amomum_tsaoko\results2\plots\Amomum_tsaoko_3_SSP126-2090S_clamping.png

Time since start: 1169.937

Writing file E:\Amomum_tsaoko\results2\Amomum_tsaoko_3_SSP126-2090S_novel.asc

Writing file E:\Amomum_tsaoko\results2\Amomum_tsaoko_3_SSP126-2090S_novel_limiting.asc

Time since start: 1174.419

Projecting...

Writing file E:\Amomum_tsaoko\results2\Amomum_tsaoko_3_SSP585-2050S.asc

Writing file E:\Amomum_tsaoko\results2\Amomum_tsaoko_3_SSP585-2050S_clamping.asc

Time since start: 1178.065

Writing E:\Amomum_tsaoko\results2\plots\Amomum_tsaoko_3_SSP585-2050S.png

Time since start: 1179.389

Writing E:\Amomum_tsaoko\results2\plots\Amomum_tsaoko_3_SSP585-2050S_clamping.png

Time since start: 1180.761

Writing file E:\Amomum_tsaoko\results2\Amomum_tsaoko_3_SSP585-2050S_novel.asc

Writing file E:\Amomum_tsaoko\results2\Amomum_tsaoko_3_SSP585-2050S_novel_limiting.asc

Time since start: 1185.304

Projecting...

Writing file E:\Amomum_tsaoko\results2\Amomum_tsaoko_3_SSP585-2090S.asc

Writing file E:\Amomum_tsaoko\results2\Amomum_tsaoko_3_SSP585-2090S_clamping.asc

Time since start: 1188.95

Writing E:\Amomum_tsaoko\results2\plots\Amomum_tsaoko_3_SSP585-2090S.png

Time since start: 1190.265

Writing E:\Amomum_tsaoko\results2\plots\Amomum_tsaoko_3_SSP585-2090S_clamping.png

Time since start: 1191.603

Writing file E:\Amomum_tsaoko\results2\Amomum_tsaoko_3_SSP585-2090S_novel.asc

Writing file E:\Amomum_tsaoko\results2\Amomum_tsaoko_3_SSP585-2090S_novel_limiting.asc

Time since start: 1196.056

Amomum_tsaoko_3 response curves

Response curve: only aspect

Making features

makeFeatures: max memory 259522560, total allocated 139407360, free 38980704, used 100426656, increment 26558128

Amomum_tsaoko_3 aspect:

Regularization values: linear/quadratic/product: 0.385, categorical: 0.250, threshold: 1.770, hinge: 0.500

23 samples

Density: max memory 259522560, total allocated 139407360, free 35791144, used 103616216, increment 3189560

linearPredictor: max memory 259522560, total allocated 139407360, free 35791144, used 103616216, increment 0

FeaturedSpace: max memory 259522560, total allocated 139407360, free 35791144, used 103616216, increment 0

Sequential: max memory 259522560, total allocated 139407360, free 35791144, used 103616216, increment 0

Initial loss: 9.212438170058329

Time since start: 1198.209

120: time = 1.268000 loss = 9.046069

Resulting gain: 0.16636920129076316

Amomum_tsaoko_3 response curves

Response curve: only awc_class

Making features

makeFeatures: max memory 259522560, total allocated 139407360, free 55326080, used 84081280, increment -19534936

Amomum_tsaoko_3 awc_class:

Regularization values: linear/quadratic/product: 0.385, categorical: 0.250, threshold: 1.770, hinge: 0.500

23 samples

Density: max memory 259522560, total allocated 139407360, free 54544176, used 84863184, increment 781904

linearPredictor: max memory 259522560, total allocated 139407360, free 54544176, used 84863184, increment 0

Deactivating (awc_class=2.0)

Deactivating (awc_class=3.0)

Deactivating (awc_class=4.0)

Deactivating (awc_class=6.0)

FeaturedSpace: max memory 259522560, total allocated 139407360, free 54544176, used 84863184, increment 0

Sequential: max memory 259522560, total allocated 139407360, free 54544176, used 84863184, increment 0

Initial loss: 9.212438170058329

80: time = 0.152000 loss = 9.058521

Resulting gain: 0.15391686332801235

Amomum_tsaoko_3 response curves

Response curve: only bio_12

Making features

makeFeatures: max memory 259522560, total allocated 139407360, free 49914112, used 89493248, increment 4630064

Amomum_tsaoko_3 bio_12:

Regularization values: linear/quadratic/product: 0.385, categorical: 0.250, threshold: 1.770, hinge: 0.500

23 samples

Density: max memory 259522560, total allocated 139407360, free 48981728, used 90425632, increment 932384

linearPredictor: max memory 259522560, total allocated 139407360, free 48901544, used 90505816, increment 80184

FeaturedSpace: max memory 259522560, total allocated 139407360, free 48901544, used 90505816, increment 0

Sequential: max memory 259522560, total allocated 139407360, free 48901544, used 90505816, increment 0

Initial loss: 9.212438170058329

Time since start: 1199.611

240: time = 1.084000 loss = 8.187292

Resulting gain: 1.02514657150593

Amomum_tsaoko_3 response curves

Response curve: only bio_15

Making features

makeFeatures: max memory 259522560, total allocated 139407360, free 65764632, used 73642728, increment -16863088

Amomum_tsaoko_3 bio_15:

Regularization values: linear/quadratic/product: 0.385, categorical: 0.250, threshold: 1.770, hinge: 0.500

23 samples

Density: max memory 259522560, total allocated 139407360, free 62722520, used 76684840, increment 3042112

linearPredictor: max memory 259522560, total allocated 139407360, free 62722520, used 76684840, increment 0

FeaturedSpace: max memory 259522560, total allocated 139407360, free 62722520, used 76684840, increment 0

Sequential: max memory 259522560, total allocated 139407360, free 62722520, used 76684840, increment 0

Initial loss: 9.212438170058329

Time since start: 1201.291

160: time = 1.588000 loss = 8.751952

Resulting gain: 0.4604860094844625

Amomum_tsaoko_3 response curves

Response curve: only bio_17

Making features

makeFeatures: max memory 259522560, total allocated 139407360, free 57554664, used 81852696, increment 5167856

Amomum_tsaoko_3 bio_17:

Regularization values: linear/quadratic/product: 0.385, categorical: 0.250, threshold: 1.770, hinge: 0.500

23 samples

Density: max memory 259522560, total allocated 139407360, free 56769880, used 82637480, increment 784784

linearPredictor: max memory 259522560, total allocated 139407360, free 56769880, used 82637480, increment 0

FeaturedSpace: max memory 259522560, total allocated 139407360, free 56769880, used 82637480, increment 0

Sequential: max memory 259522560, total allocated 139407360, free 56769880, used 82637480, increment 0

Initial loss: 9.212438170058329

Time since start: 1202.606

380: time = 1.222000 loss = 7.793283

Resulting gain: 1.4191551419177912

Amomum_tsaoko_3 response curves

Response curve: only bio_4

Making features

makeFeatures: max memory 259522560, total allocated 139407360, free 32086512, used 107320848, increment 24683368

Amomum_tsaoko_3 bio_4:

Regularization values: linear/quadratic/product: 0.385, categorical: 0.250, threshold: 1.770, hinge: 0.500

23 samples

Density: max memory 259522560, total allocated 139407360, free 67269264, used 72138096, increment -35182752

linearPredictor: max memory 259522560, total allocated 139407360, free 67269264, used 72138096, increment 0

FeaturedSpace: max memory 259522560, total allocated 139407360, free 67269264, used 72138096, increment 0

Sequential: max memory 259522560, total allocated 139407360, free 67269264, used 72138096, increment 0

Initial loss: 9.212438170058329

Time since start: 1204.33

160: time = 1.618000 loss = 7.351609

Resulting gain: 1.8608293801084166

Amomum_tsaoko_3 response curves

Response curve: only bio_6

Making features

makeFeatures: max memory 259522560, total allocated 139407360, free 45958240, used 93449120, increment 21311024

Amomum_tsaoko_3 bio_6:

Regularization values: linear/quadratic/product: 0.385, categorical: 0.250, threshold: 1.770, hinge: 0.500

23 samples

Density: max memory 259522560, total allocated 139407360, free 44189800, used 95217560, increment 1768440

linearPredictor: max memory 259522560, total allocated 139407360, free 44109616, used 95297744, increment 80184

FeaturedSpace: max memory 259522560, total allocated 139407360, free 44109616, used 95297744, increment 0

Sequential: max memory 259522560, total allocated 139407360, free 44109616, used 95297744, increment 0

Initial loss: 9.212438170058329

Time since start: 1205.682

140: time = 1.244000 loss = 8.201088

Resulting gain: 1.0113499947696276

Amomum_tsaoko_3 response curves

Response curve: only elev

Making features

makeFeatures: max memory 259522560, total allocated 139407360, free 57038448, used 82368912, increment -12928832

Amomum_tsaoko_3 elev:

Regularization values: linear/quadratic/product: 0.385, categorical: 0.250, threshold: 1.770, hinge: 0.500

23 samples

Density: max memory 259522560, total allocated 139407360, free 55403400, used 84003960, increment 1635048

linearPredictor: max memory 259522560, total allocated 139407360, free 55403400, used 84003960, increment 0

FeaturedSpace: max memory 259522560, total allocated 139407360, free 55403400, used 84003960, increment 0

Sequential: max memory 259522560, total allocated 139407360, free 55403400, used 84003960, increment 0

Initial loss: 9.212438170058329

160: time = 0.861000 loss = 8.829996

Resulting gain: 0.3824419911057806

Amomum_tsaoko_3 response curves

Time since start: 1206.711

Response curve: only s_caco3

Making features

makeFeatures: max memory 259522560, total allocated 139407360, free 67236712, used 72170648, increment -11833312

Amomum_tsaoko_3 s_caco3:

Regularization values: linear/quadratic/product: 0.385, categorical: 0.250, threshold: 1.770, hinge: 0.500

23 samples

Density: max memory 259522560, total allocated 139407360, free 66459360, used 72948000, increment 777352

linearPredictor: max memory 259522560, total allocated 139407360, free 66459360, used 72948000, increment 0

FeaturedSpace: max memory 259522560, total allocated 139407360, free 66459360, used 72948000, increment 0

Sequential: max memory 259522560, total allocated 139407360, free 66459360, used 72948000, increment 0

Initial loss: 9.212438170058329

120: time = 0.556000 loss = 8.807822

Resulting gain: 0.40461631382136964

Amomum_tsaoko_3 response curves

Response curve: only s_ph_h2o

Making features

makeFeatures: max memory 259522560, total allocated 139407360, free 60108480, used 79298880, increment 6350880

Amomum_tsaoko_3 s_ph_h2o:

Regularization values: linear/quadratic/product: 0.385, categorical: 0.250, threshold: 1.770, hinge: 0.500

23 samples

Density: max memory 259522560, total allocated 139407360, free 59163616, used 80243744, increment 944864

linearPredictor: max memory 259522560, total allocated 139407360, free 59163616, used 80243744, increment 0

FeaturedSpace: max memory 259522560, total allocated 139407360, free 59163616, used 80243744, increment 0

Sequential: max memory 259522560, total allocated 139407360, free 59163616, used 80243744, increment 0

Initial loss: 9.212438170058329

Time since start: 1208.214

240: time = 0.835000 loss = 8.772464

Resulting gain: 0.43997415173697796

Amomum_tsaoko_3 response curves

Response curve: only slope

Making features

makeFeatures: max memory 259522560, total allocated 139407360, free 51002112, used 88405248, increment 8161504

Amomum_tsaoko_3 slope:

Regularization values: linear/quadratic/product: 0.385, categorical: 0.250, threshold: 1.770, hinge: 0.500

23 samples

Density: max memory 259522560, total allocated 139407360, free 48636392, used 90770968, increment 2365720

linearPredictor: max memory 259522560, total allocated 139407360, free 48636392, used 90770968, increment 0

FeaturedSpace: max memory 259522560, total allocated 139407360, free 48636392, used 90770968, increment 0

Sequential: max memory 259522560, total allocated 139407360, free 48636392, used 90770968, increment 0

Initial loss: 9.212438170058329

Time since start: 1210.663

260: time = 2.368000 loss = 9.008819

Resulting gain: 0.203619127966304

Amomum_tsaoko_3 response curves

Response curve: only t_clay

Making features

makeFeatures: max memory 259522560, total allocated 139407360, free 36071264, used 103336096, increment 12565128

Amomum_tsaoko_3 t_clay:

Regularization values: linear/quadratic/product: 0.385, categorical: 0.250, threshold: 1.770, hinge: 0.500

23 samples

Density: max memory 259522560, total allocated 139407360, free 35290064, used 104117296, increment 781200

linearPredictor: max memory 259522560, total allocated 139407360, free 35290064, used 104117296, increment 0

FeaturedSpace: max memory 259522560, total allocated 139407360, free 35290064, used 104117296, increment 0

Sequential: max memory 259522560, total allocated 139407360, free 35290064, used 104117296, increment 0

Initial loss: 9.212438170058329

180: time = 0.716000 loss = 8.753377

Resulting gain: 0.4590608003088974

Amomum_tsaoko_3 response curves

Response curve: only t_oc

Making features

makeFeatures: max memory 259522560, total allocated 139407360, free 65934872, used 73472488, increment -30644808

Amomum_tsaoko_3 t_oc:

Regularization values: linear/quadratic/product: 0.385, categorical: 0.250, threshold: 1.770, hinge: 0.500

23 samples

Density: max memory 259522560, total allocated 139407360, free 65166864, used 74240496, increment 768008

linearPredictor: max memory 259522560, total allocated 139407360, free 65086680, used 74320680, increment 80184

FeaturedSpace: max memory 259522560, total allocated 139407360, free 65086680, used 74320680, increment 0

Sequential: max memory 259522560, total allocated 139407360, free 65086680, used 74320680, increment 0

Initial loss: 9.212438170058329

Time since start: 1211.953

100: time = 0.388000 loss = 8.903732

Resulting gain: 0.3087057891316274

Amomum_tsaoko_3 response curves

Response curve: only t_sand

Making features

makeFeatures: max memory 259522560, total allocated 139407360, free 58091800, used 81315560, increment 6994880

Amomum_tsaoko_3 t_sand:

Regularization values: linear/quadratic/product: 0.385, categorical: 0.250, threshold: 1.770, hinge: 0.500

23 samples

Density: max memory 259522560, total allocated 139407360, free 57323792, used 82083568, increment 768008

linearPredictor: max memory 259522560, total allocated 139407360, free 57243608, used 82163752, increment 80184

FeaturedSpace: max memory 259522560, total allocated 139407360, free 57243608, used 82163752, increment 0

Sequential: max memory 259522560, total allocated 139407360, free 57243608, used 82163752, increment 0

Initial loss: 9.212438170058329

100: time = 0.430000 loss = 9.116192

Resulting gain: 0.09624596052091583

Amomum_tsaoko_3 response curves

Time since start: 1213.409

Jackknife: leave aspect out

Making features

makeFeatures: max memory 259522560, total allocated 139407360, free 41036024, used 98371336, increment 16207584

Amomum_tsaoko_3 aspect:

Regularization values: linear/quadratic/product: 0.385, categorical: 0.250, threshold: 1.770, hinge: 0.500

23 samples

Density: max memory 259522560, total allocated 139407360, free 49232808, used 90174552, increment -8196784

linearPredictor: max memory 259522560, total allocated 139407360, free 49232808, used 90174552, increment 0

Deactivating (awc_class=2.0)

Deactivating (awc_class=3.0)

Deactivating (awc_class=4.0)

Deactivating (awc_class=6.0)

FeaturedSpace: max memory 259522560, total allocated 139407360, free 49232808, used 90174552, increment 0

Sequential: max memory 259522560, total allocated 139407360, free 49232808, used 90174552, increment 0

Initial loss: 9.212438170058329

Initial test loss: 9.212438170058329

Time since start: 1229.125

500: time = 15.443000 loss = 6.422406 testLoss 6.836105

Jackknife: leave awc_class out

Making features

makeFeatures: max memory 259522560, total allocated 139407360, free 47268424, used 92138936, increment 1964384

Amomum_tsaoko_3 awc_class:

Regularization values: linear/quadratic/product: 0.385, categorical: 0.250, threshold: 1.770, hinge: 0.500

23 samples

Density: max memory 259522560, total allocated 139407360, free 26174192, used 113233168, increment 21094232

linearPredictor: max memory 259522560, total allocated 139407360, free 26094008, used 113313352, increment 80184

FeaturedSpace: max memory 259522560, total allocated 139407360, free 26094008, used 113313352, increment 0

Sequential: max memory 259522560, total allocated 139407360, free 26094008, used 113313352, increment 0

Initial loss: 9.212438170058329

Initial test loss: 9.212438170058329

Time since start: 1248.278

500: time = 18.824000 loss = 6.344205 testLoss 6.698850

Jackknife: leave bio_12 out

Making features

makeFeatures: max memory 259522560, total allocated 139407360, free 73884736, used 65522624, increment -47790728

Amomum_tsaoko_3 bio_12:

Regularization values: linear/quadratic/product: 0.385, categorical: 0.250, threshold: 1.770, hinge: 0.500

23 samples

Density: max memory 259522560, total allocated 139407360, free 77391384, used 62015976, increment -3506648

linearPredictor: max memory 259522560, total allocated 139407360, free 77391384, used 62015976, increment 0

Deactivating (awc_class=2.0)

Deactivating (awc_class=3.0)

Deactivating (awc_class=4.0)

Deactivating (awc_class=6.0)

FeaturedSpace: max memory 259522560, total allocated 139407360, free 77391384, used 62015976, increment 0

Sequential: max memory 259522560, total allocated 139407360, free 77391384, used 62015976, increment 0

Initial loss: 9.212438170058329

Initial test loss: 9.212438170058329

Time since start: 1260.429

320: time = 11.849000 loss = 6.453216 testLoss 6.777128

Jackknife: leave bio_15 out

Making features

makeFeatures: max memory 259522560, total allocated 139407360, free 62752232, used 76655128, increment 14639152

Amomum_tsaoko_3 bio_15:

Regularization values: linear/quadratic/product: 0.385, categorical: 0.250, threshold: 1.770, hinge: 0.500

23 samples

Density: max memory 259522560, total allocated 139407360, free 44573960, used 94833400, increment 18178272

linearPredictor: max memory 259522560, total allocated 139407360, free 44573960, used 94833400, increment 0

Deactivating (awc_class=2.0)

Deactivating (awc_class=3.0)

Deactivating (awc_class=4.0)

Deactivating (awc_class=6.0)

FeaturedSpace: max memory 259522560, total allocated 139407360, free 44573960, used 94833400, increment 0

Sequential: max memory 259522560, total allocated 139407360, free 44573960, used 94833400, increment 0

Initial loss: 9.212438170058329

Initial test loss: 9.212438170058329

Time since start: 1274.326

440: time = 13.604000 loss = 6.344094 testLoss 6.697224

Jackknife: leave bio_17 out

Making features

makeFeatures: max memory 259522560, total allocated 139407360, free 32925480, used 106481880, increment 11648480

Amomum_tsaoko_3 bio_17:

Regularization values: linear/quadratic/product: 0.385, categorical: 0.250, threshold: 1.770, hinge: 0.500

23 samples

Density: max memory 259522560, total allocated 139407360, free 40355288, used 99052072, increment -7429808

linearPredictor: max memory 259522560, total allocated 139407360, free 40355288, used 99052072, increment 0

Deactivating (awc_class=2.0)

Deactivating (awc_class=3.0)

Deactivating (awc_class=4.0)

Deactivating (awc_class=6.0)

FeaturedSpace: max memory 259522560, total allocated 139407360, free 40355288, used 99052072, increment 0

Sequential: max memory 259522560, total allocated 139407360, free 40355288, used 99052072, increment 0

Initial loss: 9.212438170058329

Initial test loss: 9.212438170058329

Time since start: 1289.915

400: time = 15.277000 loss = 6.442700 testLoss 6.882739

Jackknife: leave bio_4 out

Making features

makeFeatures: max memory 259522560, total allocated 139407360, free 42313256, used 97094104, increment -1957968

Amomum_tsaoko_3 bio_4:

Regularization values: linear/quadratic/product: 0.385, categorical: 0.250, threshold: 1.770, hinge: 0.500

23 samples

Density: max memory 259522560, total allocated 139407360, free 24386336, used 115021024, increment 17926920

linearPredictor: max memory 259522560, total allocated 139407360, free 24386336, used 115021024, increment 0

Deactivating (awc_class=2.0)

Deactivating (awc_class=3.0)

Deactivating (awc_class=4.0)

Deactivating (awc_class=6.0)

FeaturedSpace: max memory 259522560, total allocated 139407360, free 24386336, used 115021024, increment 0

Sequential: max memory 259522560, total allocated 139407360, free 24386336, used 115021024, increment 0

Initial loss: 9.212438170058329

Initial test loss: 9.212438170058329

Time since start: 1305.821

500: time = 15.622000 loss = 6.478057 testLoss 6.832046

Jackknife: leave bio_6 out

Making features

makeFeatures: max memory 259522560, total allocated 139407360, free 81080920, used 58326440, increment -56694584

Amomum_tsaoko_3 bio_6:

Regularization values: linear/quadratic/product: 0.385, categorical: 0.250, threshold: 1.770, hinge: 0.500

23 samples

Density: max memory 259522560, total allocated 139407360, free 61762448, used 77644912, increment 19318472

linearPredictor: max memory 259522560, total allocated 139407360, free 61762448, used 77644912, increment 0

Deactivating (awc_class=2.0)

Deactivating (awc_class=3.0)

Deactivating (awc_class=4.0)

Deactivating (awc_class=6.0)

FeaturedSpace: max memory 259522560, total allocated 139407360, free 61762448, used 77644912, increment 0

Sequential: max memory 259522560, total allocated 139407360, free 61762448, used 77644912, increment 0

Initial loss: 9.212438170058329

Initial test loss: 9.212438170058329

Time since start: 1322.503

500: time = 16.385000 loss = 6.343939 testLoss 6.719541

Jackknife: leave elev out

Making features

makeFeatures: max memory 259522560, total allocated 139407360, free 53014688, used 86392672, increment 8747760

Amomum_tsaoko_3 elev:

Regularization values: linear/quadratic/product: 0.385, categorical: 0.250, threshold: 1.770, hinge: 0.500

23 samples

Density: max memory 259522560, total allocated 139407360, free 59375496, used 80031864, increment -6360808

linearPredictor: max memory 259522560, total allocated 139407360, free 59295312, used 80112048, increment 80184

Deactivating (awc_class=2.0)

Deactivating (awc_class=3.0)

Deactivating (awc_class=4.0)

Deactivating (awc_class=6.0)

FeaturedSpace: max memory 259522560, total allocated 139407360, free 59295312, used 80112048, increment 0

Sequential: max memory 259522560, total allocated 139407360, free 59295312, used 80112048, increment 0

Initial loss: 9.212438170058329

Initial test loss: 9.212438170058329

Time since start: 1340.506

500: time = 17.711000 loss = 6.399680 testLoss 6.761432

Jackknife: leave s_caco3 out

Making features

makeFeatures: max memory 259522560, total allocated 139407360, free 60248752, used 79158608, increment -953440

Amomum_tsaoko_3 s_caco3:

Regularization values: linear/quadratic/product: 0.385, categorical: 0.250, threshold: 1.770, hinge: 0.500

23 samples

Density: max memory 259522560, total allocated 139407360, free 39930064, used 99477296, increment 20318688

linearPredictor: max memory 259522560, total allocated 139407360, free 39930064, used 99477296, increment 0

Deactivating (awc_class=2.0)

Deactivating (awc_class=3.0)

Deactivating (awc_class=4.0)

Deactivating (awc_class=6.0)

FeaturedSpace: max memory 259522560, total allocated 139407360, free 39930064, used 99477296, increment 0

Sequential: max memory 259522560, total allocated 139407360, free 39930064, used 99477296, increment 0

Initial loss: 9.212438170058329

Initial test loss: 9.212438170058329

Time since start: 1356.64

420: time = 15.851000 loss = 6.405177 testLoss 6.843394

Jackknife: leave s_ph_h2o out

Making features

makeFeatures: max memory 259522560, total allocated 139407360, free 39360968, used 100046392, increment 569096

Amomum_tsaoko_3 s_ph_h2o:

Regularization values: linear/quadratic/product: 0.385, categorical: 0.250, threshold: 1.770, hinge: 0.500

23 samples

Density: max memory 259522560, total allocated 139407360, free 19092856, used 120314504, increment 20268112

linearPredictor: max memory 259522560, total allocated 139407360, free 19012672, used 120394688, increment 80184

Deactivating (awc_class=2.0)

Deactivating (awc_class=3.0)

Deactivating (awc_class=4.0)

Deactivating (awc_class=6.0)

FeaturedSpace: max memory 259522560, total allocated 139407360, free 19012672, used 120394688, increment 0

Sequential: max memory 259522560, total allocated 139407360, free 19012672, used 120394688, increment 0

Initial loss: 9.212438170058329

Initial test loss: 9.212438170058329

Time since start: 1375.029

480: time = 18.092000 loss = 6.354377 testLoss 6.753921

Jackknife: leave slope out

Making features

makeFeatures: max memory 259522560, total allocated 139407360, free 66159216, used 73248144, increment -47146544

Amomum_tsaoko_3 slope:

Regularization values: linear/quadratic/product: 0.385, categorical: 0.250, threshold: 1.770, hinge: 0.500

23 samples

Density: max memory 259522560, total allocated 139407360, free 72989536, used 66417824, increment -6830320

linearPredictor: max memory 259522560, total allocated 139407360, free 72989536, used 66417824, increment 0

Deactivating (awc_class=2.0)

Deactivating (awc_class=3.0)

Deactivating (awc_class=4.0)

Deactivating (awc_class=6.0)

FeaturedSpace: max memory 259522560, total allocated 139407360, free 72989536, used 66417824, increment 0

Sequential: max memory 259522560, total allocated 139407360, free 72989536, used 66417824, increment 0

Initial loss: 9.212438170058329

Initial test loss: 9.212438170058329

Time since start: 1393.108

500: time = 17.790000 loss = 6.563217 testLoss 6.800543

Jackknife: leave t_clay out

Making features

makeFeatures: max memory 259522560, total allocated 139407360, free 64029016, used 75378344, increment 8960520

Amomum_tsaoko_3 t_clay:

Regularization values: linear/quadratic/product: 0.385, categorical: 0.250, threshold: 1.770, hinge: 0.500

23 samples

Density: max memory 259522560, total allocated 139407360, free 43549048, used 95858312, increment 20479968

linearPredictor: max memory 259522560, total allocated 139407360, free 43549048, used 95858312, increment 0

Deactivating (awc_class=2.0)

Deactivating (awc_class=3.0)

Deactivating (awc_class=4.0)

Deactivating (awc_class=6.0)

FeaturedSpace: max memory 259522560, total allocated 139407360, free 43549048, used 95858312, increment 0

Sequential: max memory 259522560, total allocated 139407360, free 43549048, used 95858312, increment 0

Initial loss: 9.212438170058329

Initial test loss: 9.212438170058329

Time since start: 1411.869

480: time = 18.434000 loss = 6.343510 testLoss 6.695943

Jackknife: leave t_oc out

Making features

makeFeatures: max memory 259522560, total allocated 139407360, free 32777088, used 106630272, increment 10771960

Amomum_tsaoko_3 t_oc:

Regularization values: linear/quadratic/product: 0.385, categorical: 0.250, threshold: 1.770, hinge: 0.500

23 samples

Density: max memory 259522560, total allocated 139407360, free 40686432, used 98720928, increment -7909344

linearPredictor: max memory 259522560, total allocated 139407360, free 40686432, used 98720928, increment 0

Deactivating (awc_class=2.0)

Deactivating (awc_class=3.0)

Deactivating (awc_class=4.0)

Deactivating (awc_class=6.0)

FeaturedSpace: max memory 259522560, total allocated 139407360, free 40686432, used 98720928, increment 0

Sequential: max memory 259522560, total allocated 139407360, free 40686432, used 98720928, increment 0

Initial loss: 9.212438170058329

Initial test loss: 9.212438170058329

Time since start: 1430.364

480: time = 18.191000 loss = 6.343712 testLoss 6.701786

Jackknife: leave t_sand out

Making features

makeFeatures: max memory 259522560, total allocated 139407360, free 39818576, used 99588784, increment 867856

Amomum_tsaoko_3 t_sand:

Regularization values: linear/quadratic/product: 0.385, categorical: 0.250, threshold: 1.770, hinge: 0.500

23 samples

Density: max memory 259522560, total allocated 139407360, free 19550464, used 119856896, increment 20268112

linearPredictor: max memory 259522560, total allocated 139407360, free 19470280, used 119937080, increment 80184

Deactivating (awc_class=2.0)

Deactivating (awc_class=3.0)

Deactivating (awc_class=4.0)

Deactivating (awc_class=6.0)

FeaturedSpace: max memory 259522560, total allocated 139407360, free 19470280, used 119937080, increment 0

Sequential: max memory 259522560, total allocated 139407360, free 19470280, used 119937080, increment 0

Initial loss: 9.212438170058329

Initial test loss: 9.212438170058329

Time since start: 1449.307

500: time = 18.656000 loss = 6.343452 testLoss 6.697650

Jackknife: only aspect

Making features

makeFeatures: max memory 259522560, total allocated 139407360, free 88809496, used 50597864, increment -69339216

Amomum_tsaoko_3 aspect:

Regularization values: linear/quadratic/product: 0.385, categorical: 0.250, threshold: 1.770, hinge: 0.500

23 samples

Density: max memory 259522560, total allocated 139407360, free 85593616, used 53813744, increment 3215880

linearPredictor: max memory 259522560, total allocated 139407360, free 85593616, used 53813744, increment 0

FeaturedSpace: max memory 259522560, total allocated 139407360, free 85593616, used 53813744, increment 0

Sequential: max memory 259522560, total allocated 139407360, free 85593616, used 53813744, increment 0

Initial loss: 9.212438170058329

Initial test loss: 9.212438170058329

Time since start: 1450.578

120: time = 1.227000 loss = 9.046069 testLoss 9.042271

Res.gain: 0.16636920129076316

Jackknife: only awc_class

Making features

makeFeatures: max memory 259522560, total allocated 139407360, free 70514128, used 68893232, increment 15079488

Amomum_tsaoko_3 awc_class:

Regularization values: linear/quadratic/product: 0.385, categorical: 0.250, threshold: 1.770, hinge: 0.500

23 samples

Density: max memory 259522560, total allocated 139407360, free 70503432, used 68903928, increment 10696

linearPredictor: max memory 259522560, total allocated 139407360, free 70423248, used 68984112, increment 80184

Deactivating (awc_class=2.0)

Deactivating (awc_class=3.0)

Deactivating (awc_class=4.0)

Deactivating (awc_class=6.0)

FeaturedSpace: max memory 259522560, total allocated 139407360, free 70423248, used 68984112, increment 0

Sequential: max memory 259522560, total allocated 139407360, free 70423248, used 68984112, increment 0

Initial loss: 9.212438170058329

Initial test loss: 9.212438170058329

80: time = 0.130000 loss = 9.058521 testLoss 8.903468

Res.gain: 0.15391686332801235

Jackknife: only bio_12

Making features

makeFeatures: max memory 259522560, total allocated 139407360, free 69067664, used 70339696, increment 1355584

Amomum_tsaoko_3 bio_12:

Regularization values: linear/quadratic/product: 0.385, categorical: 0.250, threshold: 1.770, hinge: 0.500

23 samples

Density: max memory 259522560, total allocated 139407360, free 67435400, used 71971960, increment 1632264

linearPredictor: max memory 259522560, total allocated 139407360, free 67435400, used 71971960, increment 0

FeaturedSpace: max memory 259522560, total allocated 139407360, free 67435400, used 71971960, increment 0

Sequential: max memory 259522560, total allocated 139407360, free 67435400, used 71971960, increment 0

Initial loss: 9.212438170058329

Initial test loss: 9.212438170058329

Time since start: 1451.823

240: time = 1.083000 loss = 8.187292 testLoss 8.128654

Res.gain: 1.02514657150593

Jackknife: only bio_15

Making features

makeFeatures: max memory 259522560, total allocated 139407360, free 89551376, used 49855984, increment -22115976

Amomum_tsaoko_3 bio_15:

Regularization values: linear/quadratic/product: 0.385, categorical: 0.250, threshold: 1.770, hinge: 0.500

23 samples

Density: max memory 259522560, total allocated 139407360, free 86343320, used 53064040, increment 3208056

linearPredictor: max memory 259522560, total allocated 139407360, free 86343320, used 53064040, increment 0

FeaturedSpace: max memory 259522560, total allocated 139407360, free 86343320, used 53064040, increment 0

Sequential: max memory 259522560, total allocated 139407360, free 86343320, used 53064040, increment 0

Initial loss: 9.212438170058329

Initial test loss: 9.212438170058329

Time since start: 1453.405

160: time = 1.554000 loss = 8.751952 testLoss 8.795318

Res.gain: 0.4604860094844625

Jackknife: only bio_17

Making features

makeFeatures: max memory 259522560, total allocated 139407360, free 84318288, used 55089072, increment 2025032

Amomum_tsaoko_3 bio_17:

Regularization values: linear/quadratic/product: 0.385, categorical: 0.250, threshold: 1.770, hinge: 0.500

23 samples

Density: max memory 259522560, total allocated 139407360, free 83497408, used 55909952, increment 820880

linearPredictor: max memory 259522560, total allocated 139407360, free 83497408, used 55909952, increment 0

FeaturedSpace: max memory 259522560, total allocated 139407360, free 83497408, used 55909952, increment 0

Sequential: max memory 259522560, total allocated 139407360, free 83497408, used 55909952, increment 0

Initial loss: 9.212438170058329

Initial test loss: 9.212438170058329

Time since start: 1454.638

380: time = 1.209000 loss = 7.793283 testLoss 8.291030

Res.gain: 1.4191551419177912

Jackknife: only bio_4

Making features

makeFeatures: max memory 259522560, total allocated 139407360, free 62208904, used 77198456, increment 21288504

Amomum_tsaoko_3 bio_4:

Regularization values: linear/quadratic/product: 0.385, categorical: 0.250, threshold: 1.770, hinge: 0.500

23 samples

Density: max memory 259522560, total allocated 139407360, free 59327896, used 80079464, increment 2881008

linearPredictor: max memory 259522560, total allocated 139407360, free 59327896, used 80079464, increment 0

FeaturedSpace: max memory 259522560, total allocated 139407360, free 59327896, used 80079464, increment 0

Sequential: max memory 259522560, total allocated 139407360, free 59327896, used 80079464, increment 0

Initial loss: 9.212438170058329

Initial test loss: 9.212438170058329

Time since start: 1456.273

160: time = 1.603000 loss = 7.351609 testLoss 7.958465

Res.gain: 1.8608293801084166

Jackknife: only bio_6

Making features

makeFeatures: max memory 259522560, total allocated 139407360, free 80150936, used 59256424, increment -20823040

Amomum_tsaoko_3 bio_6:

Regularization values: linear/quadratic/product: 0.385, categorical: 0.250, threshold: 1.770, hinge: 0.500

23 samples

Density: max memory 259522560, total allocated 139407360, free 77710328, used 61697032, increment 2440608

linearPredictor: max memory 259522560, total allocated 139407360, free 77710328, used 61697032, increment 0

FeaturedSpace: max memory 259522560, total allocated 139407360, free 77710328, used 61697032, increment 0

Sequential: max memory 259522560, total allocated 139407360, free 77710328, used 61697032, increment 0

Initial loss: 9.212438170058329

Initial test loss: 9.212438170058329

Time since start: 1457.587

140: time = 1.282000 loss = 8.201088 testLoss 8.038132

Res.gain: 1.0113499947696276

Jackknife: only elev

Making features

makeFeatures: max memory 259522560, total allocated 139407360, free 94308128, used 45099232, increment -16597800

Amomum_tsaoko_3 elev:

Regularization values: linear/quadratic/product: 0.385, categorical: 0.250, threshold: 1.770, hinge: 0.500

23 samples

Density: max memory 259522560, total allocated 139407360, free 92677184, used 46730176, increment 1630944

linearPredictor: max memory 259522560, total allocated 139407360, free 92677184, used 46730176, increment 0

FeaturedSpace: max memory 259522560, total allocated 139407360, free 92677184, used 46730176, increment 0

Sequential: max memory 259522560, total allocated 139407360, free 92677184, used 46730176, increment 0

Initial loss: 9.212438170058329

Initial test loss: 9.212438170058329

160: time = 0.904000 loss = 8.829996 testLoss 9.337397

Res.gain: 0.3824419911057806

Jackknife: only s_caco3

Making features

makeFeatures: max memory 259522560, total allocated 139407360, free 71496752, used 67910608, increment 21180432

Amomum_tsaoko_3 s_caco3:

Regularization values: linear/quadratic/product: 0.385, categorical: 0.250, threshold: 1.770, hinge: 0.500

23 samples

Density: max memory 259522560, total allocated 139407360, free 70631008, used 68776352, increment 865744

linearPredictor: max memory 259522560, total allocated 139407360, free 70550824, used 68856536, increment 80184

FeaturedSpace: max memory 259522560, total allocated 139407360, free 70550824, used 68856536, increment 0

Sequential: max memory 259522560, total allocated 139407360, free 70550824, used 68856536, increment 0

Initial loss: 9.212438170058329

Initial test loss: 9.212438170058329

Time since start: 1459.155

120: time = 0.604000 loss = 8.807822 testLoss 8.814386

Res.gain: 0.40461631382136964

Jackknife: only s_ph_h2o

Making features

makeFeatures: max memory 259522560, total allocated 139407360, free 67443408, used 71963952, increment 3107416

Amomum_tsaoko_3 s_ph_h2o:

Regularization values: linear/quadratic/product: 0.385, categorical: 0.250, threshold: 1.770, hinge: 0.500

23 samples

Density: max memory 259522560, total allocated 139407360, free 66665440, used 72741920, increment 777968

linearPredictor: max memory 259522560, total allocated 139407360, free 66665440, used 72741920, increment 0

FeaturedSpace: max memory 259522560, total allocated 139407360, free 66665440, used 72741920, increment 0

Sequential: max memory 259522560, total allocated 139407360, free 66665440, used 72741920, increment 0

Initial loss: 9.212438170058329

Initial test loss: 9.212438170058329

240: time = 0.829000 loss = 8.772464 testLoss 8.705861

Res.gain: 0.43997415173697796

Jackknife: only slope

Making features

makeFeatures: max memory 259522560, total allocated 139407360, free 61779312, used 77628048, increment 4886128

Amomum_tsaoko_3 slope:

Regularization values: linear/quadratic/product: 0.385, categorical: 0.250, threshold: 1.770, hinge: 0.500

23 samples

Density: max memory 259522560, total allocated 139407360, free 59157800, used 80249560, increment 2621512

linearPredictor: max memory 259522560, total allocated 139407360, free 59157800, used 80249560, increment 0

FeaturedSpace: max memory 259522560, total allocated 139407360, free 59157800, used 80249560, increment 0

Sequential: max memory 259522560, total allocated 139407360, free 59157800, used 80249560, increment 0

Initial loss: 9.212438170058329

Initial test loss: 9.212438170058329

Time since start: 1462.444

260: time = 2.416000 loss = 9.008819 testLoss 8.986178

Res.gain: 0.203619127966304

Jackknife: only t_clay

Making features

makeFeatures: max memory 259522560, total allocated 139407360, free 89119680, used 50287680, increment -29961880

Amomum_tsaoko_3 t_clay:

Regularization values: linear/quadratic/product: 0.385, categorical: 0.250, threshold: 1.770, hinge: 0.500

23 samples

Density: max memory 259522560, total allocated 139407360, free 87484120, used 51923240, increment 1635560

linearPredictor: max memory 259522560, total allocated 139407360, free 87484120, used 51923240, increment 0

FeaturedSpace: max memory 259522560, total allocated 139407360, free 87484120, used 51923240, increment 0

Sequential: max memory 259522560, total allocated 139407360, free 87484120, used 51923240, increment 0

Initial loss: 9.212438170058329

Initial test loss: 9.212438170058329

180: time = 0.764000 loss = 8.753377 testLoss 8.592608

Res.gain: 0.4590608003088974

Jackknife: only t_oc

Making features

makeFeatures: max memory 259522560, total allocated 139407360, free 83875856, used 55531504, increment 3608264

Amomum_tsaoko_3 t_oc:

Regularization values: linear/quadratic/product: 0.385, categorical: 0.250, threshold: 1.770, hinge: 0.500

23 samples

Density: max memory 259522560, total allocated 139407360, free 83095792, used 56311568, increment 780064

linearPredictor: max memory 259522560, total allocated 139407360, free 83095792, used 56311568, increment 0

FeaturedSpace: max memory 259522560, total allocated 139407360, free 83095792, used 56311568, increment 0

Sequential: max memory 259522560, total allocated 139407360, free 83095792, used 56311568, increment 0

Initial loss: 9.212438170058329

Initial test loss: 9.212438170058329

Time since start: 1463.732

100: time = 0.448000 loss = 8.903732 testLoss 8.781084

Res.gain: 0.3087057891316274

Jackknife: only t_sand

Making features

makeFeatures: max memory 259522560, total allocated 139407360, free 79989256, used 59418104, increment 3106536

Amomum_tsaoko_3 t_sand:

Regularization values: linear/quadratic/product: 0.385, categorical: 0.250, threshold: 1.770, hinge: 0.500

23 samples

Density: max memory 259522560, total allocated 139407360, free 79043872, used 60363488, increment 945384

linearPredictor: max memory 259522560, total allocated 139407360, free 78963688, used 60443672, increment 80184

FeaturedSpace: max memory 259522560, total allocated 139407360, free 78963688, used 60443672, increment 0

Sequential: max memory 259522560, total allocated 139407360, free 78963688, used 60443672, increment 0

Initial loss: 9.212438170058329

Initial test loss: 9.212438170058329

100: time = 0.470000 loss = 9.116192 testLoss 9.037428

Res.gain: 0.09624596052091583

getSamples: max memory 259522560, total allocated 139407360, free 63678816, used 75728544, increment 15284872

Making features

makeFeatures: max memory 259522560, total allocated 139407360, free 87311472, used 52095888, increment -23632656

Amomum_tsaoko_4:

Regularization values: linear/quadratic/product: 0.385, categorical: 0.250, threshold: 1.770, hinge: 0.500

Time since start: 1464.754

23 samples

Density: max memory 259522560, total allocated 139407360, free 66271304, used 73136056, increment 21040168

linearPredictor: max memory 259522560, total allocated 139407360, free 66271304, used 73136056, increment 0

Deactivating (awc_class=2.0)

Deactivating (awc_class=3.0)

Deactivating (awc_class=4.0)

Deactivating (awc_class=5.0)

Deactivating (awc_class=6.0)

FeaturedSpace: max memory 259522560, total allocated 139407360, free 66271304, used 73136056, increment 0

Sequential: max memory 259522560, total allocated 139407360, free 66271304, used 73136056, increment 0

Initial loss: 9.21253795551967

Initial test loss: 9.21253795551967

Time since start: 1484.13

500: time = 19.352000 loss = 7.154986 testLoss 6.612491

Resulting gain: 2.05755168616155

Projecting...

Writing file E:\Amomum_tsaoko\results2\Amomum_tsaoko_4.asc

Time since start: 1487.932

Writing E:\Amomum_tsaoko\results2\plots\Amomum_tsaoko_4.png

Time since start: 1489.414

Projecting...

Writing file E:\Amomum_tsaoko\results2\Amomum_tsaoko_4_LGM.asc

Writing file E:\Amomum_tsaoko\results2\Amomum_tsaoko_4_LGM_clamping.asc

Time since start: 1494.039

Writing E:\Amomum_tsaoko\results2\plots\Amomum_tsaoko_4_LGM.png

Time since start: 1495.396

Writing E:\Amomum_tsaoko\results2\plots\Amomum_tsaoko_4_LGM_clamping.png

Time since start: 1496.757

Writing file E:\Amomum_tsaoko\results2\Amomum_tsaoko_4_LGM_novel.asc

Writing file E:\Amomum_tsaoko\results2\Amomum_tsaoko_4_LGM_novel_limiting.asc

Time since start: 1501.206

Projecting...

Writing file E:\Amomum_tsaoko\results2\Amomum_tsaoko_4_MH.asc

Writing file E:\Amomum_tsaoko\results2\Amomum_tsaoko_4_MH_clamping.asc

Time since start: 1505.814

Writing E:\Amomum_tsaoko\results2\plots\Amomum_tsaoko_4_MH.png

Time since start: 1507.122

Writing E:\Amomum_tsaoko\results2\plots\Amomum_tsaoko_4_MH_clamping.png

Time since start: 1508.498

Writing file E:\Amomum_tsaoko\results2\Amomum_tsaoko_4_MH_novel.asc

Writing file E:\Amomum_tsaoko\results2\Amomum_tsaoko_4_MH_novel_limiting.asc

Time since start: 1513.033

Projecting...

Writing file E:\Amomum_tsaoko\results2\Amomum_tsaoko_4_SSP126-2050S.asc

Writing file E:\Amomum_tsaoko\results2\Amomum_tsaoko_4_SSP126-2050S_clamping.asc

Time since start: 1516.861

Writing E:\Amomum_tsaoko\results2\plots\Amomum_tsaoko_4_SSP126-2050S.png

Time since start: 1518.143

Writing E:\Amomum_tsaoko\results2\plots\Amomum_tsaoko_4_SSP126-2050S_clamping.png

Time since start: 1519.564

Writing file E:\Amomum_tsaoko\results2\Amomum_tsaoko_4_SSP126-2050S_novel.asc

Writing file E:\Amomum_tsaoko\results2\Amomum_tsaoko_4_SSP126-2050S_novel_limiting.asc

Time since start: 1524.195

Projecting...

Writing file E:\Amomum_tsaoko\results2\Amomum_tsaoko_4_SSP126-2090S.asc

Writing file E:\Amomum_tsaoko\results2\Amomum_tsaoko_4_SSP126-2090S_clamping.asc

Time since start: 1528.082

Writing E:\Amomum_tsaoko\results2\plots\Amomum_tsaoko_4_SSP126-2090S.png

Time since start: 1529.445

Writing E:\Amomum_tsaoko\results2\plots\Amomum_tsaoko_4_SSP126-2090S_clamping.png

Time since start: 1530.819

Writing file E:\Amomum_tsaoko\results2\Amomum_tsaoko_4_SSP126-2090S_novel.asc

Writing file E:\Amomum_tsaoko\results2\Amomum_tsaoko_4_SSP126-2090S_novel_limiting.asc

Time since start: 1535.498

Projecting...

Writing file E:\Amomum_tsaoko\results2\Amomum_tsaoko_4_SSP585-2050S.asc

Writing file E:\Amomum_tsaoko\results2\Amomum_tsaoko_4_SSP585-2050S_clamping.asc

Time since start: 1539.367

Writing E:\Amomum_tsaoko\results2\plots\Amomum_tsaoko_4_SSP585-2050S.png

Time since start: 1540.795

Writing E:\Amomum_tsaoko\results2\plots\Amomum_tsaoko_4_SSP585-2050S_clamping.png

Time since start: 1542.154

Writing file E:\Amomum_tsaoko\results2\Amomum_tsaoko_4_SSP585-2050S_novel.asc

Writing file E:\Amomum_tsaoko\results2\Amomum_tsaoko_4_SSP585-2050S_novel_limiting.asc

Time since start: 1546.596

Projecting...

Writing file E:\Amomum_tsaoko\results2\Amomum_tsaoko_4_SSP585-2090S.asc

Writing file E:\Amomum_tsaoko\results2\Amomum_tsaoko_4_SSP585-2090S_clamping.asc

Time since start: 1550.436

Writing E:\Amomum_tsaoko\results2\plots\Amomum_tsaoko_4_SSP585-2090S.png

Time since start: 1551.763

Writing E:\Amomum_tsaoko\results2\plots\Amomum_tsaoko_4_SSP585-2090S_clamping.png

Time since start: 1553.032

Writing file E:\Amomum_tsaoko\results2\Amomum_tsaoko_4_SSP585-2090S_novel.asc

Writing file E:\Amomum_tsaoko\results2\Amomum_tsaoko_4_SSP585-2090S_novel_limiting.asc

Time since start: 1557.497

Amomum_tsaoko_4 response curves

Response curve: only aspect

Making features

makeFeatures: max memory 259522560, total allocated 139407360, free 58782504, used 80624856, increment 7488800

Amomum_tsaoko_4 aspect:

Regularization values: linear/quadratic/product: 0.385, categorical: 0.250, threshold: 1.770, hinge: 0.500

23 samples

Density: max memory 259522560, total allocated 139407360, free 56175120, used 83232240, increment 2607384

linearPredictor: max memory 259522560, total allocated 139407360, free 56175120, used 83232240, increment 0

FeaturedSpace: max memory 259522560, total allocated 139407360, free 56175120, used 83232240, increment 0

Sequential: max memory 259522560, total allocated 139407360, free 56175120, used 83232240, increment 0

Initial loss: 9.21253795551967

Time since start: 1559.803

120: time = 1.385000 loss = 8.990602

Resulting gain: 0.22193629579181895

Amomum_tsaoko_4 response curves

Response curve: only awc_class

Making features

makeFeatures: max memory 259522560, total allocated 139407360, free 36531784, used 102875576, increment 19643336

Amomum_tsaoko_4 awc_class:

Regularization values: linear/quadratic/product: 0.385, categorical: 0.250, threshold: 1.770, hinge: 0.500

23 samples

Density: max memory 259522560, total allocated 139407360, free 36519720, used 102887640, increment 12064

linearPredictor: max memory 259522560, total allocated 139407360, free 36519720, used 102887640, increment 0

Deactivating (awc_class=2.0)

Deactivating (awc_class=3.0)

Deactivating (awc_class=4.0)

Deactivating (awc_class=5.0)

Deactivating (awc_class=6.0)

FeaturedSpace: max memory 259522560, total allocated 139407360, free 36519720, used 102887640, increment 0

Sequential: max memory 259522560, total allocated 139407360, free 36519720, used 102887640, increment 0

Initial loss: 9.21253795551967

60: time = 0.100000 loss = 9.163118

Resulting gain: 0.04941999573573064

Amomum_tsaoko_4 response curves

Response curve: only bio_12

Making features

makeFeatures: max memory 259522560, total allocated 139407360, free 70740160, used 68667200, increment -34220440

Amomum_tsaoko_4 bio_12:

Regularization values: linear/quadratic/product: 0.385, categorical: 0.250, threshold: 1.770, hinge: 0.500

23 samples

Density: max memory 259522560, total allocated 139407360, free 69186688, used 70220672, increment 1553472

linearPredictor: max memory 259522560, total allocated 139407360, free 69186688, used 70220672, increment 0

FeaturedSpace: max memory 259522560, total allocated 139407360, free 69186688, used 70220672, increment 0

Sequential: max memory 259522560, total allocated 139407360, free 69186688, used 70220672, increment 0

Initial loss: 9.21253795551967

100: time = 0.562000 loss = 8.314502

Resulting gain: 0.8980360254175448

Amomum_tsaoko_4 response curves

Response curve: only bio_15

Making features

makeFeatures: max memory 259522560, total allocated 139407360, free 64037024, used 75370336, increment 5149664

Amomum_tsaoko_4 bio_15:

Regularization values: linear/quadratic/product: 0.385, categorical: 0.250, threshold: 1.770, hinge: 0.500

23 samples

Density: max memory 259522560, total allocated 139407360, free 60888272, used 78519088, increment 3148752

linearPredictor: max memory 259522560, total allocated 139407360, free 60888272, used 78519088, increment 0

FeaturedSpace: max memory 259522560, total allocated 139407360, free 60888272, used 78519088, increment 0

Sequential: max memory 259522560, total allocated 139407360, free 60888272, used 78519088, increment 0

Initial loss: 9.21253795551967

Time since start: 1562.47

180: time = 1.744000 loss = 8.936686

Resulting gain: 0.2758519617599582

Amomum_tsaoko_4 response curves

Response curve: only bio_17

Making features

makeFeatures: max memory 259522560, total allocated 139407360, free 56356296, used 83051064, increment 4531976

Amomum_tsaoko_4 bio_17:

Regularization values: linear/quadratic/product: 0.385, categorical: 0.250, threshold: 1.770, hinge: 0.500

23 samples

Density: max memory 259522560, total allocated 139407360, free 55573288, used 83834072, increment 783008

linearPredictor: max memory 259522560, total allocated 139407360, free 55573288, used 83834072, increment 0

FeaturedSpace: max memory 259522560, total allocated 139407360, free 55573288, used 83834072, increment 0

Sequential: max memory 259522560, total allocated 139407360, free 55573288, used 83834072, increment 0

Initial loss: 9.21253795551967

Time since start: 1564.14

460: time = 1.567000 loss = 8.193234

Resulting gain: 1.0193040257324775

Amomum_tsaoko_4 response curves

Response curve: only bio_4

Making features

makeFeatures: max memory 259522560, total allocated 139407360, free 64745624, used 74661736, increment -9172336

Amomum_tsaoko_4 bio_4:

Regularization values: linear/quadratic/product: 0.385, categorical: 0.250, threshold: 1.770, hinge: 0.500

23 samples

Density: max memory 259522560, total allocated 139407360, free 61991784, used 77415576, increment 2753840

linearPredictor: max memory 259522560, total allocated 139407360, free 61911592, used 77495768, increment 80192

FeaturedSpace: max memory 259522560, total allocated 139407360, free 61911592, used 77495768, increment 0

Sequential: max memory 259522560, total allocated 139407360, free 61911592, used 77495768, increment 0

Initial loss: 9.21253795551967

Time since start: 1565.473

120: time = 1.240000 loss = 7.960986

Resulting gain: 1.251551892438771

Amomum_tsaoko_4 response curves

Response curve: only bio_6

Making features

makeFeatures: max memory 259522560, total allocated 139407360, free 44139896, used 95267464, increment 17771696

Amomum_tsaoko_4 bio_6:

Regularization values: linear/quadratic/product: 0.385, categorical: 0.250, threshold: 1.770, hinge: 0.500

23 samples

Density: max memory 259522560, total allocated 139407360, free 41722680, used 97684680, increment 2417216

linearPredictor: max memory 259522560, total allocated 139407360, free 41722680, used 97684680, increment 0

FeaturedSpace: max memory 259522560, total allocated 139407360, free 41722680, used 97684680, increment 0

Sequential: max memory 259522560, total allocated 139407360, free 41722680, used 97684680, increment 0

Initial loss: 9.21253795551967

Time since start: 1566.805

140: time = 1.227000 loss = 8.272658

Resulting gain: 0.9398803784520275

Amomum_tsaoko_4 response curves

Response curve: only elev

Making features

makeFeatures: max memory 259522560, total allocated 139407360, free 55190088, used 84217272, increment -13467408

Amomum_tsaoko_4 elev:

Regularization values: linear/quadratic/product: 0.385, categorical: 0.250, threshold: 1.770, hinge: 0.500

23 samples

Density: max memory 259522560, total allocated 139407360, free 53636776, used 85770584, increment 1553312

linearPredictor: max memory 259522560, total allocated 139407360, free 53636776, used 85770584, increment 0

FeaturedSpace: max memory 259522560, total allocated 139407360, free 53636776, used 85770584, increment 0

Sequential: max memory 259522560, total allocated 139407360, free 53636776, used 85770584, increment 0

Initial loss: 9.21253795551967

Time since start: 1567.907

180: time = 1.010000 loss = 9.107066

Resulting gain: 0.10547216570914308

Amomum_tsaoko_4 response curves

Response curve: only s_caco3

Making features

makeFeatures: max memory 259522560, total allocated 139407360, free 54535248, used 84872112, increment -898472

Amomum_tsaoko_4 s_caco3:

Regularization values: linear/quadratic/product: 0.385, categorical: 0.250, threshold: 1.770, hinge: 0.500

23 samples

Density: max memory 259522560, total allocated 139407360, free 53733016, used 85674344, increment 802232

linearPredictor: max memory 259522560, total allocated 139407360, free 53733016, used 85674344, increment 0

FeaturedSpace: max memory 259522560, total allocated 139407360, free 53733016, used 85674344, increment 0

Sequential: max memory 259522560, total allocated 139407360, free 53733016, used 85674344, increment 0

Initial loss: 9.21253795551967

80: time = 0.474000 loss = 8.730136

Resulting gain: 0.4824022922888265

Amomum_tsaoko_4 response curves

Response curve: only s_ph_h2o

Making features

makeFeatures: max memory 259522560, total allocated 139407360, free 48271448, used 91135912, increment 5461568

Amomum_tsaoko_4 s_ph_h2o:

Regularization values: linear/quadratic/product: 0.385, categorical: 0.250, threshold: 1.770, hinge: 0.500

23 samples

Density: max memory 259522560, total allocated 139407360, free 46634648, used 92772712, increment 1636800

linearPredictor: max memory 259522560, total allocated 139407360, free 46634648, used 92772712, increment 0

FeaturedSpace: max memory 259522560, total allocated 139407360, free 46634648, used 92772712, increment 0

Sequential: max memory 259522560, total allocated 139407360, free 46634648, used 92772712, increment 0

Initial loss: 9.21253795551967

Time since start: 1569.222

220: time = 0.646000 loss = 8.532506

Resulting gain: 0.6800323152594565

Amomum_tsaoko_4 response curves

Response curve: only slope

Making features

makeFeatures: max memory 259522560, total allocated 139407360, free 38635032, used 100772328, increment 7999616

Amomum_tsaoko_4 slope:

Regularization values: linear/quadratic/product: 0.385, categorical: 0.250, threshold: 1.770, hinge: 0.500

23 samples

Density: max memory 259522560, total allocated 139407360, free 36307992, used 103099368, increment 2327040

linearPredictor: max memory 259522560, total allocated 139407360, free 36307992, used 103099368, increment 0

FeaturedSpace: max memory 259522560, total allocated 139407360, free 36307992, used 103099368, increment 0

Sequential: max memory 259522560, total allocated 139407360, free 36307992, used 103099368, increment 0

Initial loss: 9.21253795551967

Time since start: 1572.128

320: time = 2.822000 loss = 9.020725

Resulting gain: 0.1918130970965457

Amomum_tsaoko_4 response curves

Response curve: only t_clay

Making features

makeFeatures: max memory 259522560, total allocated 139407360, free 66674536, used 72732824, increment -30366544

Amomum_tsaoko_4 t_clay:

Regularization values: linear/quadratic/product: 0.385, categorical: 0.250, threshold: 1.770, hinge: 0.500

23 samples

Density: max memory 259522560, total allocated 139407360, free 65686384, used 73720976, increment 988152

linearPredictor: max memory 259522560, total allocated 139407360, free 65606192, used 73801168, increment 80192

FeaturedSpace: max memory 259522560, total allocated 139407360, free 65606192, used 73801168, increment 0

Sequential: max memory 259522560, total allocated 139407360, free 65606192, used 73801168, increment 0

Initial loss: 9.21253795551967

120: time = 0.574000 loss = 8.795239

Resulting gain: 0.41729866435116847

Amomum_tsaoko_4 response curves

Response curve: only t_oc

Making features

makeFeatures: max memory 259522560, total allocated 139407360, free 59078592, used 80328768, increment 6527600

Amomum_tsaoko_4 t_oc:

Regularization values: linear/quadratic/product: 0.385, categorical: 0.250, threshold: 1.770, hinge: 0.500

23 samples

Density: max memory 259522560, total allocated 139407360, free 58291224, used 81116136, increment 787368

linearPredictor: max memory 259522560, total allocated 139407360, free 58291224, used 81116136, increment 0

FeaturedSpace: max memory 259522560, total allocated 139407360, free 58291224, used 81116136, increment 0

Sequential: max memory 259522560, total allocated 139407360, free 58291224, used 81116136, increment 0

Initial loss: 9.21253795551967

Time since start: 1573.405

160: time = 0.518000 loss = 8.860053

Resulting gain: 0.352484784555644

Amomum_tsaoko_4 response curves

Response curve: only t_sand

Making features

makeFeatures: max memory 259522560, total allocated 139407360, free 49293072, used 90114288, increment 8998152

Amomum_tsaoko_4 t_sand:

Regularization values: linear/quadratic/product: 0.385, categorical: 0.250, threshold: 1.770, hinge: 0.500

23 samples

Density: max memory 259522560, total allocated 139407360, free 48509920, used 90897440, increment 783152

linearPredictor: max memory 259522560, total allocated 139407360, free 48509920, used 90897440, increment 0

FeaturedSpace: max memory 259522560, total allocated 139407360, free 48509920, used 90897440, increment 0

Sequential: max memory 259522560, total allocated 139407360, free 48509920, used 90897440, increment 0

Initial loss: 9.21253795551967

180: time = 0.709000 loss = 8.992108

Resulting gain: 0.2204297162220623

Amomum_tsaoko_4 response curves

Time since start: 1575.355

Jackknife: leave aspect out

Making features

makeFeatures: max memory 259522560, total allocated 139407360, free 67290296, used 72117064, increment -18780376

Amomum_tsaoko_4 aspect:

Regularization values: linear/quadratic/product: 0.385, categorical: 0.250, threshold: 1.770, hinge: 0.500

23 samples

Density: max memory 259522560, total allocated 139407360, free 48836104, used 90571256, increment 18454192

linearPredictor: max memory 259522560, total allocated 139407360, free 48836104, used 90571256, increment 0

Deactivating (awc_class=2.0)

Deactivating (awc_class=3.0)

Deactivating (awc_class=4.0)

Deactivating (awc_class=5.0)

Deactivating (awc_class=6.0)

FeaturedSpace: max memory 259522560, total allocated 139407360, free 48836104, used 90571256, increment 0

Sequential: max memory 259522560, total allocated 139407360, free 48836104, used 90571256, increment 0

Initial loss: 9.21253795551967

Initial test loss: 9.21253795551967

Time since start: 1589.905

440: time = 14.280000 loss = 7.376742 testLoss 6.767599

Jackknife: leave awc_class out

Making features

makeFeatures: max memory 259522560, total allocated 139407360, free 52263728, used 87143632, increment -3427624

Amomum_tsaoko_4 awc_class:

Regularization values: linear/quadratic/product: 0.385, categorical: 0.250, threshold: 1.770, hinge: 0.500

23 samples

Density: max memory 259522560, total allocated 139407360, free 31152568, used 108254792, increment 21111160

linearPredictor: max memory 259522560, total allocated 139407360, free 31072376, used 108334984, increment 80192

FeaturedSpace: max memory 259522560, total allocated 139407360, free 31072376, used 108334984, increment 0

Sequential: max memory 259522560, total allocated 139407360, free 31072376, used 108334984, increment 0

Initial loss: 9.21253795551967

Initial test loss: 9.21253795551967

Time since start: 1609.923

500: time = 19.683000 loss = 7.191904 testLoss 6.481846

Jackknife: leave bio_12 out

Making features

makeFeatures: max memory 259522560, total allocated 139407360, free 32506280, used 106901080, increment -1433904

Amomum_tsaoko_4 bio_12:

Regularization values: linear/quadratic/product: 0.385, categorical: 0.250, threshold: 1.770, hinge: 0.500

23 samples

Density: max memory 259522560, total allocated 139407360, free 12702376, used 126704984, increment 19803904

linearPredictor: max memory 259522560, total allocated 139407360, free 12622184, used 126785176, increment 80192

Deactivating (awc_class=2.0)

Deactivating (awc_class=3.0)

Deactivating (awc_class=4.0)

Deactivating (awc_class=5.0)

Deactivating (awc_class=6.0)

FeaturedSpace: max memory 259522560, total allocated 139407360, free 12622184, used 126785176, increment 0

Sequential: max memory 259522560, total allocated 139407360, free 12622184, used 126785176, increment 0

Initial loss: 9.21253795551967

Initial test loss: 9.21253795551967

Time since start: 1629.118

500: time = 18.922000 loss = 7.249782 testLoss 6.855726

Jackknife: leave bio_15 out

Making features

makeFeatures: max memory 259522560, total allocated 139407360, free 62875176, used 76532184, increment -50252992

Amomum_tsaoko_4 bio_15:

Regularization values: linear/quadratic/product: 0.385, categorical: 0.250, threshold: 1.770, hinge: 0.500

23 samples

Density: max memory 259522560, total allocated 139407360, free 73080832, used 66326528, increment -10205656

linearPredictor: max memory 259522560, total allocated 139407360, free 73080832, used 66326528, increment 0

Deactivating (awc_class=2.0)

Deactivating (awc_class=3.0)

Deactivating (awc_class=4.0)

Deactivating (awc_class=5.0)

Deactivating (awc_class=6.0)

FeaturedSpace: max memory 259522560, total allocated 139407360, free 73080832, used 66326528, increment 0

Sequential: max memory 259522560, total allocated 139407360, free 73080832, used 66326528, increment 0

Initial loss: 9.21253795551967

Initial test loss: 9.21253795551967

Time since start: 1646.118

500: time = 16.660000 loss = 7.157378 testLoss 6.612958

Jackknife: leave bio_17 out

Making features

makeFeatures: max memory 259522560, total allocated 139407360, free 70040192, used 69367168, increment 3040640

Amomum_tsaoko_4 bio_17:

Regularization values: linear/quadratic/product: 0.385, categorical: 0.250, threshold: 1.770, hinge: 0.500

23 samples

Density: max memory 259522560, total allocated 139407360, free 49306128, used 90101232, increment 20734064

linearPredictor: max memory 259522560, total allocated 139407360, free 49306128, used 90101232, increment 0

Deactivating (awc_class=2.0)

Deactivating (awc_class=3.0)

Deactivating (awc_class=4.0)

Deactivating (awc_class=5.0)

Deactivating (awc_class=6.0)

FeaturedSpace: max memory 259522560, total allocated 139407360, free 49306128, used 90101232, increment 0

Sequential: max memory 259522560, total allocated 139407360, free 49306128, used 90101232, increment 0

Initial loss: 9.21253795551967

Initial test loss: 9.21253795551967

Time since start: 1665.838

500: time = 19.432000 loss = 7.201328 testLoss 6.662628

Jackknife: leave bio_4 out

Making features

makeFeatures: max memory 259522560, total allocated 139407360, free 59814560, used 79592800, increment -10508432

Amomum_tsaoko_4 bio_4:

Regularization values: linear/quadratic/product: 0.385, categorical: 0.250, threshold: 1.770, hinge: 0.500

23 samples

Density: max memory 259522560, total allocated 139407360, free 41815984, used 97591376, increment 17998576

linearPredictor: max memory 259522560, total allocated 139407360, free 41735792, used 97671568, increment 80192

Deactivating (awc_class=2.0)

Deactivating (awc_class=3.0)

Deactivating (awc_class=4.0)

Deactivating (awc_class=5.0)

Deactivating (awc_class=6.0)

FeaturedSpace: max memory 259522560, total allocated 139407360, free 41735792, used 97671568, increment 0

Sequential: max memory 259522560, total allocated 139407360, free 41735792, used 97671568, increment 0

Initial loss: 9.21253795551967

Initial test loss: 9.21253795551967

Time since start: 1682.227

500: time = 16.087000 loss = 7.160464 testLoss 6.605208

Jackknife: leave bio_6 out

Making features

makeFeatures: max memory 259522560, total allocated 139407360, free 45231736, used 94175624, increment -3495944

Amomum_tsaoko_4 bio_6:

Regularization values: linear/quadratic/product: 0.385, categorical: 0.250, threshold: 1.770, hinge: 0.500

23 samples

Density: max memory 259522560, total allocated 139407360, free 25913840, used 113493520, increment 19317896

linearPredictor: max memory 259522560, total allocated 139407360, free 25913840, used 113493520, increment 0

Deactivating (awc_class=2.0)

Deactivating (awc_class=3.0)

Deactivating (awc_class=4.0)

Deactivating (awc_class=5.0)

Deactivating (awc_class=6.0)

FeaturedSpace: max memory 259522560, total allocated 139407360, free 25913840, used 113493520, increment 0

Sequential: max memory 259522560, total allocated 139407360, free 25913840, used 113493520, increment 0

Initial loss: 9.21253795551967

Initial test loss: 9.21253795551967

Time since start: 1699.714

500: time = 17.198000 loss = 7.163432 testLoss 6.609474

Jackknife: leave elev out

Making features

makeFeatures: max memory 259522560, total allocated 139407360, free 74873072, used 64534288, increment -48959232

Amomum_tsaoko_4 elev:

Regularization values: linear/quadratic/product: 0.385, categorical: 0.250, threshold: 1.770, hinge: 0.500

23 samples

Density: max memory 259522560, total allocated 139407360, free 79055184, used 60352176, increment -4182112

linearPredictor: max memory 259522560, total allocated 139407360, free 79055184, used 60352176, increment 0

Deactivating (awc_class=2.0)

Deactivating (awc_class=3.0)

Deactivating (awc_class=4.0)

Deactivating (awc_class=5.0)

Deactivating (awc_class=6.0)

FeaturedSpace: max memory 259522560, total allocated 139407360, free 79055184, used 60352176, increment 0

Sequential: max memory 259522560, total allocated 139407360, free 79055184, used 60352176, increment 0

Initial loss: 9.21253795551967

Initial test loss: 9.21253795551967

Time since start: 1718.433

500: time = 18.424000 loss = 7.267387 testLoss 6.697404

Jackknife: leave s_caco3 out

Making features

makeFeatures: max memory 259522560, total allocated 139407360, free 46238416, used 93168944, increment 32816768

Amomum_tsaoko_4 s_caco3:

Regularization values: linear/quadratic/product: 0.385, categorical: 0.250, threshold: 1.770, hinge: 0.500

23 samples

Density: max memory 259522560, total allocated 139407360, free 55045488, used 84361872, increment -8807072

linearPredictor: max memory 259522560, total allocated 139407360, free 55045488, used 84361872, increment 0

Deactivating (awc_class=2.0)

Deactivating (awc_class=3.0)

Deactivating (awc_class=4.0)

Deactivating (awc_class=5.0)

Deactivating (awc_class=6.0)

FeaturedSpace: max memory 259522560, total allocated 139407360, free 55045488, used 84361872, increment 0

Sequential: max memory 259522560, total allocated 139407360, free 55045488, used 84361872, increment 0

Initial loss: 9.21253795551967

Initial test loss: 9.21253795551967

Time since start: 1738.263

500: time = 19.518000 loss = 7.162506 testLoss 6.602501

Jackknife: leave s_ph_h2o out

Making features

makeFeatures: max memory 259522560, total allocated 139407360, free 59632800, used 79774560, increment -4587312

Amomum_tsaoko_4 s_ph_h2o:

Regularization values: linear/quadratic/product: 0.385, categorical: 0.250, threshold: 1.770, hinge: 0.500

23 samples

Density: max memory 259522560, total allocated 139407360, free 39400064, used 100007296, increment 20232736

linearPredictor: max memory 259522560, total allocated 139407360, free 39319872, used 100087488, increment 80192

Deactivating (awc_class=2.0)

Deactivating (awc_class=3.0)

Deactivating (awc_class=4.0)

Deactivating (awc_class=5.0)

Deactivating (awc_class=6.0)

FeaturedSpace: max memory 259522560, total allocated 139407360, free 39319872, used 100087488, increment 0

Sequential: max memory 259522560, total allocated 139407360, free 39319872, used 100087488, increment 0

Initial loss: 9.21253795551967

Initial test loss: 9.21253795551967

Time since start: 1758.062

500: time = 19.491000 loss = 7.155962 testLoss 6.614404

Jackknife: leave slope out

Making features

makeFeatures: max memory 259522560, total allocated 139407360, free 44644960, used 94762400, increment -5325088

Amomum_tsaoko_4 slope:

Regularization values: linear/quadratic/product: 0.385, categorical: 0.250, threshold: 1.770, hinge: 0.500

23 samples

Density: max memory 259522560, total allocated 139407360, free 25332152, used 114075208, increment 19312808

linearPredictor: max memory 259522560, total allocated 139407360, free 25332152, used 114075208, increment 0

Deactivating (awc_class=2.0)

Deactivating (awc_class=3.0)

Deactivating (awc_class=4.0)

Deactivating (awc_class=5.0)

Deactivating (awc_class=6.0)

FeaturedSpace: max memory 259522560, total allocated 139407360, free 25332152, used 114075208, increment 0

Sequential: max memory 259522560, total allocated 139407360, free 25332152, used 114075208, increment 0

Initial loss: 9.21253795551967

Initial test loss: 9.21253795551967

Time since start: 1773.267

420: time = 14.900000 loss = 7.323113 testLoss 6.695789

Jackknife: leave t_clay out

Making features

makeFeatures: max memory 259522560, total allocated 139407360, free 82639792, used 56767568, increment -57307640

Amomum_tsaoko_4 t_clay:

Regularization values: linear/quadratic/product: 0.385, categorical: 0.250, threshold: 1.770, hinge: 0.500

23 samples

Density: max memory 259522560, total allocated 139407360, free 61859464, used 77547896, increment 20780328

linearPredictor: max memory 259522560, total allocated 139407360, free 61859464, used 77547896, increment 0

Deactivating (awc_class=2.0)

Deactivating (awc_class=3.0)

Deactivating (awc_class=4.0)

Deactivating (awc_class=5.0)

Deactivating (awc_class=6.0)

FeaturedSpace: max memory 259522560, total allocated 139407360, free 61859464, used 77547896, increment 0

Sequential: max memory 259522560, total allocated 139407360, free 61859464, used 77547896, increment 0

Initial loss: 9.21253795551967

Initial test loss: 9.21253795551967

Time since start: 1792.576

500: time = 19.025000 loss = 7.156124 testLoss 6.613425

Jackknife: leave t_oc out

Making features

makeFeatures: max memory 259522560, total allocated 139407360, free 64252208, used 75155152, increment -2392744

Amomum_tsaoko_4 t_oc:

Regularization values: linear/quadratic/product: 0.385, categorical: 0.250, threshold: 1.770, hinge: 0.500

23 samples

Density: max memory 259522560, total allocated 139407360, free 44319664, used 95087696, increment 19932544

linearPredictor: max memory 259522560, total allocated 139407360, free 44239472, used 95167888, increment 80192

Deactivating (awc_class=2.0)

Deactivating (awc_class=3.0)

Deactivating (awc_class=4.0)

Deactivating (awc_class=5.0)

Deactivating (awc_class=6.0)

FeaturedSpace: max memory 259522560, total allocated 139407360, free 44239472, used 95167888, increment 0

Sequential: max memory 259522560, total allocated 139407360, free 44239472, used 95167888, increment 0

Initial loss: 9.21253795551967

Initial test loss: 9.21253795551967

Time since start: 1812.062

500: time = 19.219000 loss = 7.156195 testLoss 6.613543

Jackknife: leave t_sand out

Making features

makeFeatures: max memory 259522560, total allocated 139407360, free 46409600, used 92997760, increment -2170128

Amomum_tsaoko_4 t_sand:

Regularization values: linear/quadratic/product: 0.385, categorical: 0.250, threshold: 1.770, hinge: 0.500

23 samples

Density: max memory 259522560, total allocated 139407360, free 49344392, used 90062968, increment -2934792

linearPredictor: max memory 259522560, total allocated 139407360, free 49344392, used 90062968, increment 0

Deactivating (awc_class=2.0)

Deactivating (awc_class=3.0)

Deactivating (awc_class=4.0)

Deactivating (awc_class=5.0)

Deactivating (awc_class=6.0)

FeaturedSpace: max memory 259522560, total allocated 139407360, free 49344392, used 90062968, increment 0

Sequential: max memory 259522560, total allocated 139407360, free 49344392, used 90062968, increment 0

Initial loss: 9.21253795551967

Initial test loss: 9.21253795551967

Time since start: 1831.43

500: time = 19.077000 loss = 7.182486 testLoss 6.595611

Jackknife: only aspect

Making features

makeFeatures: max memory 259522560, total allocated 139407360, free 36773720, used 102633640, increment 12570672

Amomum_tsaoko_4 aspect:

Regularization values: linear/quadratic/product: 0.385, categorical: 0.250, threshold: 1.770, hinge: 0.500

23 samples

Density: max memory 259522560, total allocated 139407360, free 33984984, used 105422376, increment 2788736

linearPredictor: max memory 259522560, total allocated 139407360, free 33984984, used 105422376, increment 0

FeaturedSpace: max memory 259522560, total allocated 139407360, free 33984984, used 105422376, increment 0

Sequential: max memory 259522560, total allocated 139407360, free 33984984, used 105422376, increment 0

Initial loss: 9.21253795551967

Initial test loss: 9.21253795551967

Time since start: 1832.918

120: time = 1.432000 loss = 8.990602 testLoss 9.033474

Res.gain: 0.22193629579181895

Jackknife: only awc_class

Making features

makeFeatures: max memory 259522560, total allocated 139407360, free 18536528, used 120870832, increment 15448456

Amomum_tsaoko_4 awc_class:

Regularization values: linear/quadratic/product: 0.385, categorical: 0.250, threshold: 1.770, hinge: 0.500

23 samples

Density: max memory 259522560, total allocated 139407360, free 17751600, used 121655760, increment 784928

linearPredictor: max memory 259522560, total allocated 139407360, free 17751600, used 121655760, increment 0

Deactivating (awc_class=2.0)

Deactivating (awc_class=3.0)

Deactivating (awc_class=4.0)

Deactivating (awc_class=5.0)

Deactivating (awc_class=6.0)

FeaturedSpace: max memory 259522560, total allocated 139407360, free 17751600, used 121655760, increment 0

Sequential: max memory 259522560, total allocated 139407360, free 17751600, used 121655760, increment 0

Initial loss: 9.21253795551967

Initial test loss: 9.21253795551967

60: time = 0.091000 loss = 9.163118 testLoss 9.359626

Res.gain: 0.04941999573573064

Jackknife: only bio_12

Making features

makeFeatures: max memory 259522560, total allocated 139407360, free 17370504, used 122036856, increment 381096

Amomum_tsaoko_4 bio_12:

Regularization values: linear/quadratic/product: 0.385, categorical: 0.250, threshold: 1.770, hinge: 0.500

23 samples

Density: max memory 259522560, total allocated 139407360, free 15817112, used 123590248, increment 1553392

linearPredictor: max memory 259522560, total allocated 139407360, free 15817112, used 123590248, increment 0

FeaturedSpace: max memory 259522560, total allocated 139407360, free 15817112, used 123590248, increment 0

Sequential: max memory 259522560, total allocated 139407360, free 15817112, used 123590248, increment 0

Initial loss: 9.21253795551967

Initial test loss: 9.21253795551967

100: time = 0.527000 loss = 8.314502 testLoss 8.139069

Res.gain: 0.8980360254175448

Jackknife: only bio_15

Making features

makeFeatures: max memory 259522560, total allocated 139407360, free 15107504, used 124299856, increment 709608

Amomum_tsaoko_4 bio_15:

Regularization values: linear/quadratic/product: 0.385, categorical: 0.250, threshold: 1.770, hinge: 0.500

23 samples

Density: max memory 259522560, total allocated 139407360, free 50162352, used 89245008, increment -35054848

linearPredictor: max memory 259522560, total allocated 139407360, free 50082160, used 89325200, increment 80192

FeaturedSpace: max memory 259522560, total allocated 139407360, free 50082160, used 89325200, increment 0

Sequential: max memory 259522560, total allocated 139407360, free 50082160, used 89325200, increment 0

Initial loss: 9.21253795551967

Initial test loss: 9.21253795551967

Time since start: 1835.251

180: time = 1.652000 loss = 8.936686 testLoss 8.989970

Res.gain: 0.2758519617599582

Jackknife: only bio_17

Making features

makeFeatures: max memory 259522560, total allocated 139407360, free 50055424, used 89351936, increment 26736

Amomum_tsaoko_4 bio_17:

Regularization values: linear/quadratic/product: 0.385, categorical: 0.250, threshold: 1.770, hinge: 0.500

23 samples

Density: max memory 259522560, total allocated 139407360, free 49193496, used 90213864, increment 861928

linearPredictor: max memory 259522560, total allocated 139407360, free 49113304, used 90294056, increment 80192

FeaturedSpace: max memory 259522560, total allocated 139407360, free 49113304, used 90294056, increment 0

Sequential: max memory 259522560, total allocated 139407360, free 49113304, used 90294056, increment 0

Initial loss: 9.21253795551967

Initial test loss: 9.21253795551967

Time since start: 1836.764

460: time = 1.488000 loss = 8.193234 testLoss 7.897273

Res.gain: 1.0193040257324775

Jackknife: only bio_4

Making features

makeFeatures: max memory 259522560, total allocated 139407360, free 23548816, used 115858544, increment 25564488

Amomum_tsaoko_4 bio_4:

Regularization values: linear/quadratic/product: 0.385, categorical: 0.250, threshold: 1.770, hinge: 0.500

23 samples

Density: max memory 259522560, total allocated 139407360, free 20626584, used 118780776, increment 2922232

linearPredictor: max memory 259522560, total allocated 139407360, free 20626584, used 118780776, increment 0

FeaturedSpace: max memory 259522560, total allocated 139407360, free 20626584, used 118780776, increment 0

Sequential: max memory 259522560, total allocated 139407360, free 20626584, used 118780776, increment 0

Initial loss: 9.21253795551967

Initial test loss: 9.21253795551967

Time since start: 1838.013

120: time = 1.220000 loss = 7.960986 testLoss 7.756093

Res.gain: 1.251551892438771

Jackknife: only bio_6

Making features

makeFeatures: max memory 259522560, total allocated 139407360, free 46164408, used 93242952, increment -25537824

Amomum_tsaoko_4 bio_6:

Regularization values: linear/quadratic/product: 0.385, categorical: 0.250, threshold: 1.770, hinge: 0.500

23 samples

Density: max memory 259522560, total allocated 139407360, free 43845672, used 95561688, increment 2318736

linearPredictor: max memory 259522560, total allocated 139407360, free 43845672, used 95561688, increment 0

FeaturedSpace: max memory 259522560, total allocated 139407360, free 43845672, used 95561688, increment 0

Sequential: max memory 259522560, total allocated 139407360, free 43845672, used 95561688, increment 0

Initial loss: 9.21253795551967

Initial test loss: 9.21253795551967

Time since start: 1839.195

140: time = 1.154000 loss = 8.272658 testLoss 8.135305

Res.gain: 0.9398803784520275

Jackknife: only elev

Making features

makeFeatures: max memory 259522560, total allocated 139407360, free 22543896, used 116863464, increment 21301776

Amomum_tsaoko_4 elev:

Regularization values: linear/quadratic/product: 0.385, categorical: 0.250, threshold: 1.770, hinge: 0.500

23 samples

Density: max memory 259522560, total allocated 139407360, free 20969448, used 118437912, increment 1574448

linearPredictor: max memory 259522560, total allocated 139407360, free 20969448, used 118437912, increment 0

FeaturedSpace: max memory 259522560, total allocated 139407360, free 20969448, used 118437912, increment 0

Sequential: max memory 259522560, total allocated 139407360, free 20969448, used 118437912, increment 0

Initial loss: 9.21253795551967

Initial test loss: 9.21253795551967

Time since start: 1840.27

180: time = 1.044000 loss = 9.107066 testLoss 8.838180

Res.gain: 0.10547216570914308

Jackknife: only s_caco3

Making features

makeFeatures: max memory 259522560, total allocated 139407360, free 24902144, used 114505216, increment -3932696

Amomum_tsaoko_4 s_caco3:

Regularization values: linear/quadratic/product: 0.385, categorical: 0.250, threshold: 1.770, hinge: 0.500

23 samples

Density: max memory 259522560, total allocated 139407360, free 24116864, used 115290496, increment 785280

linearPredictor: max memory 259522560, total allocated 139407360, free 24116864, used 115290496, increment 0

FeaturedSpace: max memory 259522560, total allocated 139407360, free 24116864, used 115290496, increment 0

Sequential: max memory 259522560, total allocated 139407360, free 24116864, used 115290496, increment 0

Initial loss: 9.21253795551967

Initial test loss: 9.21253795551967

80: time = 0.525000 loss = 8.730136 testLoss 8.709381

Res.gain: 0.4824022922888265

Jackknife: only s_ph_h2o

Making features

makeFeatures: max memory 259522560, total allocated 139407360, free 22089312, used 117318048, increment 2027552

Amomum_tsaoko_4 s_ph_h2o:

Regularization values: linear/quadratic/product: 0.385, categorical: 0.250, threshold: 1.770, hinge: 0.500

23 samples

Density: max memory 259522560, total allocated 139407360, free 21191008, used 118216352, increment 898304

linearPredictor: max memory 259522560, total allocated 139407360, free 21191008, used 118216352, increment 0

FeaturedSpace: max memory 259522560, total allocated 139407360, free 21191008, used 118216352, increment 0

Sequential: max memory 259522560, total allocated 139407360, free 21191008, used 118216352, increment 0

Initial loss: 9.21253795551967

Initial test loss: 9.21253795551967

Time since start: 1841.559

220: time = 0.711000 loss = 8.532506 testLoss 8.382600

Res.gain: 0.6800323152594565

Jackknife: only slope

Making features

makeFeatures: max memory 259522560, total allocated 139407360, free 16964752, used 122442608, increment 4226256

Amomum_tsaoko_4 slope:

Regularization values: linear/quadratic/product: 0.385, categorical: 0.250, threshold: 1.770, hinge: 0.500

23 samples

Density: max memory 259522560, total allocated 139407360, free 14565440, used 124841920, increment 2399312

linearPredictor: max memory 259522560, total allocated 139407360, free 14565440, used 124841920, increment 0

FeaturedSpace: max memory 259522560, total allocated 139407360, free 14565440, used 124841920, increment 0

Sequential: max memory 259522560, total allocated 139407360, free 14565440, used 124841920, increment 0

Initial loss: 9.21253795551967

Initial test loss: 9.21253795551967

Time since start: 1844.46

320: time = 2.872000 loss = 9.020725 testLoss 8.848625

Res.gain: 0.1918130970965457

Jackknife: only t_clay

Making features

makeFeatures: max memory 259522560, total allocated 139407360, free 48004096, used 91403264, increment -33438656

Amomum_tsaoko_4 t_clay:

Regularization values: linear/quadratic/product: 0.385, categorical: 0.250, threshold: 1.770, hinge: 0.500

23 samples

Density: max memory 259522560, total allocated 139407360, free 47024728, used 92382632, increment 979368

linearPredictor: max memory 259522560, total allocated 139407360, free 46944536, used 92462824, increment 80192

FeaturedSpace: max memory 259522560, total allocated 139407360, free 46944536, used 92462824, increment 0

Sequential: max memory 259522560, total allocated 139407360, free 46944536, used 92462824, increment 0

Initial loss: 9.21253795551967

Initial test loss: 9.21253795551967

120: time = 0.541000 loss = 8.795239 testLoss 8.343246

Res.gain: 0.41729866435116847

Jackknife: only t_oc

Making features

makeFeatures: max memory 259522560, total allocated 139407360, free 43803784, used 95603576, increment 3140752

Amomum_tsaoko_4 t_oc:

Regularization values: linear/quadratic/product: 0.385, categorical: 0.250, threshold: 1.770, hinge: 0.500

23 samples

Density: max memory 259522560, total allocated 139407360, free 42980432, used 96426928, increment 823352

linearPredictor: max memory 259522560, total allocated 139407360, free 42980432, used 96426928, increment 0

FeaturedSpace: max memory 259522560, total allocated 139407360, free 42980432, used 96426928, increment 0

Sequential: max memory 259522560, total allocated 139407360, free 42980432, used 96426928, increment 0

Initial loss: 9.21253795551967

Initial test loss: 9.21253795551967

Time since start: 1845.679

160: time = 0.616000 loss = 8.860053 testLoss 8.634365

Res.gain: 0.352484784555644

Jackknife: only t_sand

Making features

makeFeatures: max memory 259522560, total allocated 139407360, free 38023960, used 101383400, increment 4956472

Amomum_tsaoko_4 t_sand:

Regularization values: linear/quadratic/product: 0.385, categorical: 0.250, threshold: 1.770, hinge: 0.500

23 samples

Density: max memory 259522560, total allocated 139407360, free 37080224, used 102327136, increment 943736

linearPredictor: max memory 259522560, total allocated 139407360, free 37080224, used 102327136, increment 0

FeaturedSpace: max memory 259522560, total allocated 139407360, free 37080224, used 102327136, increment 0

Sequential: max memory 259522560, total allocated 139407360, free 37080224, used 102327136, increment 0

Initial loss: 9.21253795551967

Initial test loss: 9.21253795551967

180: time = 0.715000 loss = 8.992108 testLoss 8.707632

Res.gain: 0.2204297162220623

getSamples: max memory 259522560, total allocated 139407360, free 20375816, used 119031544, increment 16704408

Making features

Time since start: 1846.707

makeFeatures: max memory 259522560, total allocated 139407360, free 43476096, used 95931264, increment -23100280

Amomum_tsaoko_5:

Regularization values: linear/quadratic/product: 0.385, categorical: 0.250, threshold: 1.770, hinge: 0.500

23 samples

Density: max memory 259522560, total allocated 139407360, free 22515072, used 116892288, increment 20961024

linearPredictor: max memory 259522560, total allocated 139407360, free 22515072, used 116892288, increment 0

Deactivating (awc_class=2.0)

Deactivating (awc_class=3.0)

Deactivating (awc_class=4.0)

Deactivating (awc_class=6.0)

FeaturedSpace: max memory 259522560, total allocated 139407360, free 22515072, used 116892288, increment 0

Sequential: max memory 259522560, total allocated 139407360, free 22515072, used 116892288, increment 0

Initial loss: 9.212637731024866

Initial test loss: 9.212637731024866

Time since start: 1866.584

500: time = 19.677000 loss = 6.758085 testLoss 6.299167

Resulting gain: 2.454553224927837

Projecting...

Writing file E:\Amomum_tsaoko\results2\Amomum_tsaoko_5.asc

Time since start: 1870.765

Writing E:\Amomum_tsaoko\results2\plots\Amomum_tsaoko_5.png

Time since start: 1872.125

Projecting...

Writing file E:\Amomum_tsaoko\results2\Amomum_tsaoko_5_LGM.asc

Writing file E:\Amomum_tsaoko\results2\Amomum_tsaoko_5_LGM_clamping.asc

Time since start: 1877.13

Writing E:\Amomum_tsaoko\results2\plots\Amomum_tsaoko_5_LGM.png

Time since start: 1879.031

Writing E:\Amomum_tsaoko\results2\plots\Amomum_tsaoko_5_LGM_clamping.png

Time since start: 1881.011

Writing file E:\Amomum_tsaoko\results2\Amomum_tsaoko_5_LGM_novel.asc

Writing file E:\Amomum_tsaoko\results2\Amomum_tsaoko_5_LGM_novel_limiting.asc

Time since start: 1885.553

Projecting...

Writing file E:\Amomum_tsaoko\results2\Amomum_tsaoko_5_MH.asc

Writing file E:\Amomum_tsaoko\results2\Amomum_tsaoko_5_MH_clamping.asc

Time since start: 1890.38

Writing E:\Amomum_tsaoko\results2\plots\Amomum_tsaoko_5_MH.png

Time since start: 1892.179

Writing E:\Amomum_tsaoko\results2\plots\Amomum_tsaoko_5_MH_clamping.png

Time since start: 1894.074

Writing file E:\Amomum_tsaoko\results2\Amomum_tsaoko_5_MH_novel.asc

Writing file E:\Amomum_tsaoko\results2\Amomum_tsaoko_5_MH_novel_limiting.asc

Time since start: 1898.456

Projecting...

Writing file E:\Amomum_tsaoko\results2\Amomum_tsaoko_5_SSP126-2050S.asc

Writing file E:\Amomum_tsaoko\results2\Amomum_tsaoko_5_SSP126-2050S_clamping.asc

Time since start: 1902.494

Writing E:\Amomum_tsaoko\results2\plots\Amomum_tsaoko_5_SSP126-2050S.png

Time since start: 1903.824

Writing E:\Amomum_tsaoko\results2\plots\Amomum_tsaoko_5_SSP126-2050S_clamping.png

Time since start: 1905.186

Writing file E:\Amomum_tsaoko\results2\Amomum_tsaoko_5_SSP126-2050S_novel.asc

Writing file E:\Amomum_tsaoko\results2\Amomum_tsaoko_5_SSP126-2050S_novel_limiting.asc

Time since start: 1909.57

Projecting...

Writing file E:\Amomum_tsaoko\results2\Amomum_tsaoko_5_SSP126-2090S.asc

Writing file E:\Amomum_tsaoko\results2\Amomum_tsaoko_5_SSP126-2090S_clamping.asc

Time since start: 1913.591

Writing E:\Amomum_tsaoko\results2\plots\Amomum_tsaoko_5_SSP126-2090S.png

Time since start: 1914.889

Writing E:\Amomum_tsaoko\results2\plots\Amomum_tsaoko_5_SSP126-2090S_clamping.png

Time since start: 1916.246

Writing file E:\Amomum_tsaoko\results2\Amomum_tsaoko_5_SSP126-2090S_novel.asc

Writing file E:\Amomum_tsaoko\results2\Amomum_tsaoko_5_SSP126-2090S_novel_limiting.asc

Time since start: 1920.698

Projecting...

Writing file E:\Amomum_tsaoko\results2\Amomum_tsaoko_5_SSP585-2050S.asc

Writing file E:\Amomum_tsaoko\results2\Amomum_tsaoko_5_SSP585-2050S_clamping.asc

Time since start: 1924.687

Writing E:\Amomum_tsaoko\results2\plots\Amomum_tsaoko_5_SSP585-2050S.png

Time since start: 1925.985

Writing E:\Amomum_tsaoko\results2\plots\Amomum_tsaoko_5_SSP585-2050S_clamping.png

Time since start: 1927.282

Writing file E:\Amomum_tsaoko\results2\Amomum_tsaoko_5_SSP585-2050S_novel.asc

Writing file E:\Amomum_tsaoko\results2\Amomum_tsaoko_5_SSP585-2050S_novel_limiting.asc

Time since start: 1931.793

Projecting...

Writing file E:\Amomum_tsaoko\results2\Amomum_tsaoko_5_SSP585-2090S.asc

Writing file E:\Amomum_tsaoko\results2\Amomum_tsaoko_5_SSP585-2090S_clamping.asc

Time since start: 1935.72

Writing E:\Amomum_tsaoko\results2\plots\Amomum_tsaoko_5_SSP585-2090S.png

Time since start: 1937.083

Writing E:\Amomum_tsaoko\results2\plots\Amomum_tsaoko_5_SSP585-2090S_clamping.png

Time since start: 1938.413

Writing file E:\Amomum_tsaoko\results2\Amomum_tsaoko_5_SSP585-2090S_novel.asc

Writing file E:\Amomum_tsaoko\results2\Amomum_tsaoko_5_SSP585-2090S_novel_limiting.asc

Time since start: 1942.937

Amomum_tsaoko_5 response curves

Response curve: only aspect

Making features

makeFeatures: max memory 259522560, total allocated 139407360, free 39044592, used 100362768, increment -16529520

Amomum_tsaoko_5 aspect:

Regularization values: linear/quadratic/product: 0.385, categorical: 0.250, threshold: 1.770, hinge: 0.500

23 samples

Density: max memory 259522560, total allocated 139407360, free 36289768, used 103117592, increment 2754824

linearPredictor: max memory 259522560, total allocated 139407360, free 73547384, used 65859976, increment -37257616

FeaturedSpace: max memory 259522560, total allocated 139407360, free 72776664, used 66630696, increment 770720

Sequential: max memory 259522560, total allocated 139407360, free 72776664, used 66630696, increment 0

Initial loss: 9.212637731024866

Time since start: 1945.879

180: time = 1.955000 loss = 8.956515

Resulting gain: 0.256122331579677

Amomum_tsaoko_5 response curves

Response curve: only awc_class

Making features

makeFeatures: max memory 259522560, total allocated 139407360, free 67922080, used 71485280, increment 4854584

Amomum_tsaoko_5 awc_class:

Regularization values: linear/quadratic/product: 0.385, categorical: 0.250, threshold: 1.770, hinge: 0.500

23 samples

Density: max memory 259522560, total allocated 139407360, free 67910240, used 71497120, increment 11840

linearPredictor: max memory 259522560, total allocated 139407360, free 67910240, used 71497120, increment 0

Deactivating (awc_class=2.0)

Deactivating (awc_class=3.0)

Deactivating (awc_class=4.0)

Deactivating (awc_class=6.0)

FeaturedSpace: max memory 259522560, total allocated 139407360, free 67910240, used 71497120, increment 0

Sequential: max memory 259522560, total allocated 139407360, free 67910240, used 71497120, increment 0

Initial loss: 9.212637731024866

100: time = 0.191000 loss = 9.168644

Resulting gain: 0.04399404918563121

Amomum_tsaoko_5 response curves

Response curve: only bio_12

Making features

makeFeatures: max memory 259522560, total allocated 139407360, free 63239928, used 76167432, increment 4670312

Amomum_tsaoko_5 bio_12:

Regularization values: linear/quadratic/product: 0.385, categorical: 0.250, threshold: 1.770, hinge: 0.500

23 samples

Density: max memory 259522560, total allocated 139407360, free 61526096, used 77881264, increment 1713832

linearPredictor: max memory 259522560, total allocated 139407360, free 61526096, used 77881264, increment 0

FeaturedSpace: max memory 259522560, total allocated 139407360, free 61526096, used 77881264, increment 0

Sequential: max memory 259522560, total allocated 139407360, free 61526096, used 77881264, increment 0

Initial loss: 9.212637731024866

80: time = 0.485000 loss = 7.982779

Resulting gain: 1.229859140368509

Amomum_tsaoko_5 response curves

Response curve: only bio_15

Making features

makeFeatures: max memory 259522560, total allocated 139407360, free 64667840, used 74739520, increment -3141744

Amomum_tsaoko_5 bio_15:

Regularization values: linear/quadratic/product: 0.385, categorical: 0.250, threshold: 1.770, hinge: 0.500

23 samples

Density: max memory 259522560, total allocated 139407360, free 61523336, used 77884024, increment 3144504

linearPredictor: max memory 259522560, total allocated 139407360, free 61523336, used 77884024, increment 0

FeaturedSpace: max memory 259522560, total allocated 139407360, free 61523336, used 77884024, increment 0

Sequential: max memory 259522560, total allocated 139407360, free 61523336, used 77884024, increment 0

Initial loss: 9.212637731024866

Time since start: 1949.025

240: time = 2.222000 loss = 8.675885

Resulting gain: 0.5367525187465318

Amomum_tsaoko_5 response curves

Response curve: only bio_17

Making features

makeFeatures: max memory 259522560, total allocated 139407360, free 57829216, used 81578144, increment 3694120

Amomum_tsaoko_5 bio_17:

Regularization values: linear/quadratic/product: 0.385, categorical: 0.250, threshold: 1.770, hinge: 0.500

23 samples

Density: max memory 259522560, total allocated 139407360, free 57045712, used 82361648, increment 783504

linearPredictor: max memory 259522560, total allocated 139407360, free 57045712, used 82361648, increment 0

FeaturedSpace: max memory 259522560, total allocated 139407360, free 57045712, used 82361648, increment 0

Sequential: max memory 259522560, total allocated 139407360, free 57045712, used 82361648, increment 0

Initial loss: 9.212637731024866

Time since start: 1950.436

360: time = 1.301000 loss = 7.748616

Resulting gain: 1.4640215226167426

Amomum_tsaoko_5 response curves

Response curve: only bio_4

Making features

makeFeatures: max memory 259522560, total allocated 139407360, free 71536152, used 67871208, increment -14490440

Amomum_tsaoko_5 bio_4:

Regularization values: linear/quadratic/product: 0.385, categorical: 0.250, threshold: 1.770, hinge: 0.500

23 samples

Density: max memory 259522560, total allocated 139407360, free 68390088, used 71017272, increment 3146064

linearPredictor: max memory 259522560, total allocated 139407360, free 68309888, used 71097472, increment 80200

FeaturedSpace: max memory 259522560, total allocated 139407360, free 68309888, used 71097472, increment 0

Sequential: max memory 259522560, total allocated 139407360, free 68309888, used 71097472, increment 0

Initial loss: 9.212637731024866

Time since start: 1951.796

120: time = 1.267000 loss = 7.842362

Resulting gain: 1.370275236572744

Amomum_tsaoko_5 response curves

Response curve: only bio_6

Making features

makeFeatures: max memory 259522560, total allocated 139407360, free 51492320, used 87915040, increment 16817568

Amomum_tsaoko_5 bio_6:

Regularization values: linear/quadratic/product: 0.385, categorical: 0.250, threshold: 1.770, hinge: 0.500

23 samples

Density: max memory 259522560, total allocated 139407360, free 49090408, used 90316952, increment 2401912

linearPredictor: max memory 259522560, total allocated 139407360, free 49090408, used 90316952, increment 0

FeaturedSpace: max memory 259522560, total allocated 139407360, free 49090408, used 90316952, increment 0

Sequential: max memory 259522560, total allocated 139407360, free 49090408, used 90316952, increment 0

Initial loss: 9.212637731024866

Time since start: 1953.658

260: time = 1.753000 loss = 7.709475

Resulting gain: 1.5031623503871625

Amomum_tsaoko_5 response curves

Response curve: only elev

Making features

makeFeatures: max memory 259522560, total allocated 139407360, free 67343784, used 72063576, increment -18253376

Amomum_tsaoko_5 elev:

Regularization values: linear/quadratic/product: 0.385, categorical: 0.250, threshold: 1.770, hinge: 0.500

23 samples

Density: max memory 259522560, total allocated 139407360, free 65792264, used 73615096, increment 1551520

linearPredictor: max memory 259522560, total allocated 139407360, free 65792264, used 73615096, increment 0

FeaturedSpace: max memory 259522560, total allocated 139407360, free 65792264, used 73615096, increment 0

Sequential: max memory 259522560, total allocated 139407360, free 65792264, used 73615096, increment 0

Initial loss: 9.212637731024866

120: time = 0.794000 loss = 9.080291

Resulting gain: 0.13234675179986155

Amomum_tsaoko_5 response curves

Response curve: only s_caco3

Making features

makeFeatures: max memory 259522560, total allocated 139407360, free 65128688, used 74278672, increment 663576

Amomum_tsaoko_5 s_caco3:

Regularization values: linear/quadratic/product: 0.385, categorical: 0.250, threshold: 1.770, hinge: 0.500

Time since start: 1954.66

23 samples

Density: max memory 259522560, total allocated 139407360, free 64083712, used 75323648, increment 1044976

linearPredictor: max memory 259522560, total allocated 139407360, free 64083712, used 75323648, increment 0

FeaturedSpace: max memory 259522560, total allocated 139407360, free 64083712, used 75323648, increment 0

Sequential: max memory 259522560, total allocated 139407360, free 64083712, used 75323648, increment 0

Initial loss: 9.212637731024866

80: time = 0.500000 loss = 8.755787

Resulting gain: 0.45685058953585234

Amomum_tsaoko_5 response curves

Response curve: only s_ph_h2o

Making features

makeFeatures: max memory 259522560, total allocated 139407360, free 57992608, used 81414752, increment 6091104

Amomum_tsaoko_5 s_ph_h2o:

Regularization values: linear/quadratic/product: 0.385, categorical: 0.250, threshold: 1.770, hinge: 0.500

23 samples

Density: max memory 259522560, total allocated 139407360, free 57215808, used 82191552, increment 776800

linearPredictor: max memory 259522560, total allocated 139407360, free 57215808, used 82191552, increment 0

FeaturedSpace: max memory 259522560, total allocated 139407360, free 57215808, used 82191552, increment 0

Sequential: max memory 259522560, total allocated 139407360, free 57215808, used 82191552, increment 0

Initial loss: 9.212637731024866

Time since start: 1955.865

180: time = 0.610000 loss = 8.690640

Resulting gain: 0.5219974809505548

Amomum_tsaoko_5 response curves

Response curve: only slope

Making features

makeFeatures: max memory 259522560, total allocated 139407360, free 50248360, used 89159000, increment 6967448

Amomum_tsaoko_5 slope:

Regularization values: linear/quadratic/product: 0.385, categorical: 0.250, threshold: 1.770, hinge: 0.500

23 samples

Density: max memory 259522560, total allocated 139407360, free 47803464, used 91603896, increment 2444896

linearPredictor: max memory 259522560, total allocated 139407360, free 47803464, used 91603896, increment 0

FeaturedSpace: max memory 259522560, total allocated 139407360, free 47803464, used 91603896, increment 0

Sequential: max memory 259522560, total allocated 139407360, free 47803464, used 91603896, increment 0

Initial loss: 9.212637731024866

Time since start: 1958.118

260: time = 2.160000 loss = 9.080349

Resulting gain: 0.1322887053689179

Amomum_tsaoko_5 response curves

Response curve: only t_clay

Making features

makeFeatures: max memory 259522560, total allocated 139407360, free 72793200, used 66614160, increment -24989736

Amomum_tsaoko_5 t_clay:

Regularization values: linear/quadratic/product: 0.385, categorical: 0.250, threshold: 1.770, hinge: 0.500

23 samples

Density: max memory 259522560, total allocated 139407360, free 72011672, used 67395688, increment 781528

linearPredictor: max memory 259522560, total allocated 139407360, free 72011672, used 67395688, increment 0

FeaturedSpace: max memory 259522560, total allocated 139407360, free 71241248, used 68166112, increment 770424

Sequential: max memory 259522560, total allocated 139407360, free 71241248, used 68166112, increment 0

Initial loss: 9.212637731024866

120: time = 0.595000 loss = 8.711494

Resulting gain: 0.5011433199792457

Amomum_tsaoko_5 response curves

Response curve: only t_oc

Making features

makeFeatures: max memory 259522560, total allocated 139407360, free 65424328, used 73983032, increment 5816920

Amomum_tsaoko_5 t_oc:

Regularization values: linear/quadratic/product: 0.385, categorical: 0.250, threshold: 1.770, hinge: 0.500

23 samples

Density: max memory 259522560, total allocated 139407360, free 64646640, used 74760720, increment 777688

linearPredictor: max memory 259522560, total allocated 139407360, free 64646640, used 74760720, increment 0

FeaturedSpace: max memory 259522560, total allocated 139407360, free 64646640, used 74760720, increment 0

Sequential: max memory 259522560, total allocated 139407360, free 64646640, used 74760720, increment 0

Initial loss: 9.212637731024866

Time since start: 1959.577

160: time = 0.708000 loss = 8.738449

Resulting gain: 0.4741889132201518

Amomum_tsaoko_5 response curves

Response curve: only t_sand

Making features

makeFeatures: max memory 259522560, total allocated 139407360, free 55766088, used 83641272, increment 8880552

Amomum_tsaoko_5 t_sand:

Regularization values: linear/quadratic/product: 0.385, categorical: 0.250, threshold: 1.770, hinge: 0.500

23 samples

Density: max memory 259522560, total allocated 139407360, free 54984560, used 84422800, increment 781528

linearPredictor: max memory 259522560, total allocated 139407360, free 54984560, used 84422800, increment 0

FeaturedSpace: max memory 259522560, total allocated 139407360, free 54984560, used 84422800, increment 0

Sequential: max memory 259522560, total allocated 139407360, free 54984560, used 84422800, increment 0

Initial loss: 9.212637731024866

140: time = 0.610000 loss = 9.044081

Resulting gain: 0.16855636895079762

Amomum_tsaoko_5 response curves

Time since start: 1961.688

Jackknife: leave aspect out

Making features

makeFeatures: max memory 259522560, total allocated 139407360, free 38751712, used 100655648, increment 16232848

Amomum_tsaoko_5 aspect:

Regularization values: linear/quadratic/product: 0.385, categorical: 0.250, threshold: 1.770, hinge: 0.500

23 samples

Density: max memory 259522560, total allocated 139407360, free 50706224, used 88701136, increment -11954512

linearPredictor: max memory 259522560, total allocated 139407360, free 50706224, used 88701136, increment 0

Deactivating (awc_class=2.0)

Deactivating (awc_class=3.0)

Deactivating (awc_class=4.0)

Deactivating (awc_class=6.0)

FeaturedSpace: max memory 259522560, total allocated 139407360, free 50706224, used 88701136, increment 0

Sequential: max memory 259522560, total allocated 139407360, free 50706224, used 88701136, increment 0

Initial loss: 9.212637731024866

Initial test loss: 9.212637731024866

Time since start: 1977.656

500: time = 15.686000 loss = 7.010286 testLoss 6.991331

Jackknife: leave awc_class out

Making features

makeFeatures: max memory 259522560, total allocated 139407360, free 51736832, used 87670528, increment -1030608

Amomum_tsaoko_5 awc_class:

Regularization values: linear/quadratic/product: 0.385, categorical: 0.250, threshold: 1.770, hinge: 0.500

23 samples

Density: max memory 259522560, total allocated 139407360, free 30927240, used 108480120, increment 20809592

linearPredictor: max memory 259522560, total allocated 139407360, free 30162936, used 109244424, increment 764304

FeaturedSpace: max memory 259522560, total allocated 139407360, free 30162936, used 109244424, increment 0

Sequential: max memory 259522560, total allocated 139407360, free 30162936, used 109244424, increment 0

Initial loss: 9.212637731024866

Initial test loss: 9.212637731024866

Time since start: 1997.218

500: time = 19.248000 loss = 6.758180 testLoss 6.301071

Jackknife: leave bio_12 out

Making features

makeFeatures: max memory 259522560, total allocated 139407360, free 27387944, used 112019416, increment 2774992

Amomum_tsaoko_5 bio_12:

Regularization values: linear/quadratic/product: 0.385, categorical: 0.250, threshold: 1.770, hinge: 0.500

23 samples

Density: max memory 259522560, total allocated 139407360, free 7555840, used 131851520, increment 19832104

linearPredictor: max memory 259522560, total allocated 139407360, free 6785152, used 132622208, increment 770688

Deactivating (awc_class=2.0)

Deactivating (awc_class=3.0)

Deactivating (awc_class=4.0)

Deactivating (awc_class=6.0)

FeaturedSpace: max memory 259522560, total allocated 139407360, free 6785152, used 132622208, increment 0

Sequential: max memory 259522560, total allocated 139407360, free 6785152, used 132622208, increment 0

Initial loss: 9.212637731024866

Initial test loss: 9.212637731024866

Time since start: 2016.004

500: time = 18.490000 loss = 6.762829 testLoss 6.315237

Jackknife: leave bio_15 out

Making features

makeFeatures: max memory 259522560, total allocated 139407360, free 88741336, used 50666024, increment -81956184

Amomum_tsaoko_5 bio_15:

Regularization values: linear/quadratic/product: 0.385, categorical: 0.250, threshold: 1.770, hinge: 0.500

23 samples

Density: max memory 259522560, total allocated 139407360, free 70052600, used 69354760, increment 18688736

linearPredictor: max memory 259522560, total allocated 139407360, free 70052600, used 69354760, increment 0

Deactivating (awc_class=2.0)

Deactivating (awc_class=3.0)

Deactivating (awc_class=4.0)

Deactivating (awc_class=6.0)

FeaturedSpace: max memory 259522560, total allocated 139407360, free 70052600, used 69354760, increment 0

Sequential: max memory 259522560, total allocated 139407360, free 70052600, used 69354760, increment 0

Initial loss: 9.212637731024866

Initial test loss: 9.212637731024866

Time since start: 2031.833

500: time = 15.522000 loss = 6.855333 testLoss 6.353619

Jackknife: leave bio_17 out

Making features

makeFeatures: max memory 259522560, total allocated 139407360, free 61117144, used 78290216, increment 8935456

Amomum_tsaoko_5 bio_17:

Regularization values: linear/quadratic/product: 0.385, categorical: 0.250, threshold: 1.770, hinge: 0.500

23 samples

Density: max memory 259522560, total allocated 139407360, free 64140352, used 75267008, increment -3023208

linearPredictor: max memory 259522560, total allocated 139407360, free 64140352, used 75267008, increment 0

Deactivating (awc_class=2.0)

Deactivating (awc_class=3.0)

Deactivating (awc_class=4.0)

Deactivating (awc_class=6.0)

FeaturedSpace: max memory 259522560, total allocated 139407360, free 64140352, used 75267008, increment 0

Sequential: max memory 259522560, total allocated 139407360, free 64140352, used 75267008, increment 0

Initial loss: 9.212637731024866

Initial test loss: 9.212637731024866

Time since start: 2048.223

440: time = 16.089000 loss = 6.841839 testLoss 6.518473

Jackknife: leave bio_4 out

Making features

makeFeatures: max memory 259522560, total allocated 139407360, free 51220680, used 88186680, increment 12919672

Amomum_tsaoko_5 bio_4:

Regularization values: linear/quadratic/product: 0.385, categorical: 0.250, threshold: 1.770, hinge: 0.500

23 samples

Density: max memory 259522560, total allocated 139407360, free 32821152, used 106586208, increment 18399528

linearPredictor: max memory 259522560, total allocated 139407360, free 32821152, used 106586208, increment 0

Deactivating (awc_class=2.0)

Deactivating (awc_class=3.0)

Deactivating (awc_class=4.0)

Deactivating (awc_class=6.0)

FeaturedSpace: max memory 259522560, total allocated 139407360, free 32821152, used 106586208, increment 0

Sequential: max memory 259522560, total allocated 139407360, free 32821152, used 106586208, increment 0

Initial loss: 9.212637731024866

Initial test loss: 9.212637731024866

Time since start: 2063.915

500: time = 15.402000 loss = 6.763480 testLoss 6.332053

Jackknife: leave bio_6 out

Making features

makeFeatures: max memory 259522560, total allocated 139407360, free 28954240, used 110453120, increment 3866912

Amomum_tsaoko_5 bio_6:

Regularization values: linear/quadratic/product: 0.385, categorical: 0.250, threshold: 1.770, hinge: 0.500

23 samples

Density: max memory 259522560, total allocated 139407360, free 34019744, used 105387616, increment -5065504

linearPredictor: max memory 259522560, total allocated 139407360, free 34019744, used 105387616, increment 0

Deactivating (awc_class=2.0)

Deactivating (awc_class=3.0)

Deactivating (awc_class=4.0)

Deactivating (awc_class=6.0)

FeaturedSpace: max memory 259522560, total allocated 139407360, free 34019744, used 105387616, increment 0

Sequential: max memory 259522560, total allocated 139407360, free 34019744, used 105387616, increment 0

Initial loss: 9.212637731024866

Initial test loss: 9.212637731024866

Time since start: 2081.144

500: time = 16.947000 loss = 6.799762 testLoss 6.336671

Jackknife: leave elev out

Making features

makeFeatures: max memory 259522560, total allocated 139407360, free 58802584, used 80604776, increment -24782840

Amomum_tsaoko_5 elev:

Regularization values: linear/quadratic/product: 0.385, categorical: 0.250, threshold: 1.770, hinge: 0.500

23 samples

Density: max memory 259522560, total allocated 139407360, free 70159840, used 69247520, increment -11357256

linearPredictor: max memory 259522560, total allocated 139407360, free 70079640, used 69327720, increment 80200

Deactivating (awc_class=2.0)

Deactivating (awc_class=3.0)

Deactivating (awc_class=4.0)

Deactivating (awc_class=6.0)

FeaturedSpace: max memory 259522560, total allocated 139407360, free 70079640, used 69327720, increment 0

Sequential: max memory 259522560, total allocated 139407360, free 70079640, used 69327720, increment 0

Initial loss: 9.212637731024866

Initial test loss: 9.212637731024866

Time since start: 2099.206

500: time = 17.779000 loss = 6.777755 testLoss 6.306944

Jackknife: leave s_caco3 out

Making features

makeFeatures: max memory 259522560, total allocated 139407360, free 68492056, used 70915304, increment 1587584

Amomum_tsaoko_5 s_caco3:

Regularization values: linear/quadratic/product: 0.385, categorical: 0.250, threshold: 1.770, hinge: 0.500

23 samples

Density: max memory 259522560, total allocated 139407360, free 47758576, used 91648784, increment 20733480

linearPredictor: max memory 259522560, total allocated 139407360, free 47758576, used 91648784, increment 0

Deactivating (awc_class=2.0)

Deactivating (awc_class=3.0)

Deactivating (awc_class=4.0)

Deactivating (awc_class=6.0)

FeaturedSpace: max memory 259522560, total allocated 139407360, free 47758576, used 91648784, increment 0

Sequential: max memory 259522560, total allocated 139407360, free 47758576, used 91648784, increment 0

Initial loss: 9.212637731024866

Initial test loss: 9.212637731024866

Time since start: 2118.464

500: time = 18.940000 loss = 6.784368 testLoss 6.345649

Jackknife: leave s_ph_h2o out

Making features

makeFeatures: max memory 259522560, total allocated 139407360, free 47108368, used 92298992, increment 650208

Amomum_tsaoko_5 s_ph_h2o:

Regularization values: linear/quadratic/product: 0.385, categorical: 0.250, threshold: 1.770, hinge: 0.500

23 samples

Density: max memory 259522560, total allocated 139407360, free 50333760, used 89073600, increment -3225392

linearPredictor: max memory 259522560, total allocated 139407360, free 50333760, used 89073600, increment 0

Deactivating (awc_class=2.0)

Deactivating (awc_class=3.0)

Deactivating (awc_class=4.0)

Deactivating (awc_class=6.0)

FeaturedSpace: max memory 259522560, total allocated 139407360, free 50333760, used 89073600, increment 0

Sequential: max memory 259522560, total allocated 139407360, free 50333760, used 89073600, increment 0

Initial loss: 9.212637731024866

Initial test loss: 9.212637731024866

Time since start: 2138.027

500: time = 19.264000 loss = 6.802817 testLoss 6.261086

Jackknife: leave slope out

Making features

makeFeatures: max memory 259522560, total allocated 139407360, free 22748080, used 116659280, increment 27585680

Amomum_tsaoko_5 slope:

Regularization values: linear/quadratic/product: 0.385, categorical: 0.250, threshold: 1.770, hinge: 0.500

23 samples

Density: max memory 259522560, total allocated 139407360, free 29650560, used 109756800, increment -6902480

linearPredictor: max memory 259522560, total allocated 139407360, free 29570360, used 109837000, increment 80200

Deactivating (awc_class=2.0)

Deactivating (awc_class=3.0)

Deactivating (awc_class=4.0)

Deactivating (awc_class=6.0)

FeaturedSpace: max memory 259522560, total allocated 139407360, free 29570360, used 109837000, increment 0

Sequential: max memory 259522560, total allocated 139407360, free 29570360, used 109837000, increment 0

Initial loss: 9.212637731024866

Initial test loss: 9.212637731024866

Time since start: 2155.216

500: time = 16.877000 loss = 6.792925 testLoss 6.446735

Jackknife: leave t_clay out

Making features

makeFeatures: max memory 259522560, total allocated 139407360, free 79663232, used 59744128, increment -50092872

Amomum_tsaoko_5 t_clay:

Regularization values: linear/quadratic/product: 0.385, categorical: 0.250, threshold: 1.770, hinge: 0.500

23 samples

Density: max memory 259522560, total allocated 139407360, free 58848880, used 80558480, increment 20814352

linearPredictor: max memory 259522560, total allocated 139407360, free 58848880, used 80558480, increment 0

Deactivating (awc_class=2.0)

Deactivating (awc_class=3.0)

Deactivating (awc_class=4.0)

Deactivating (awc_class=6.0)

FeaturedSpace: max memory 259522560, total allocated 139407360, free 58848880, used 80558480, increment 0

Sequential: max memory 259522560, total allocated 139407360, free 58848880, used 80558480, increment 0

Initial loss: 9.212637731024866

Initial test loss: 9.212637731024866

Time since start: 2174.706

500: time = 19.208000 loss = 6.758241 testLoss 6.301723

Jackknife: leave t_oc out

Making features

makeFeatures: max memory 259522560, total allocated 139407360, free 56082416, used 83324944, increment 2766464

Amomum_tsaoko_5 t_oc:

Regularization values: linear/quadratic/product: 0.385, categorical: 0.250, threshold: 1.770, hinge: 0.500

23 samples

Density: max memory 259522560, total allocated 139407360, free 59911880, used 79495480, increment -3829464

linearPredictor: max memory 259522560, total allocated 139407360, free 59831680, used 79575680, increment 80200

Deactivating (awc_class=2.0)

Deactivating (awc_class=3.0)

Deactivating (awc_class=4.0)

Deactivating (awc_class=6.0)

FeaturedSpace: max memory 259522560, total allocated 139407360, free 59831680, used 79575680, increment 0

Sequential: max memory 259522560, total allocated 139407360, free 59831680, used 79575680, increment 0

Initial loss: 9.212637731024866

Initial test loss: 9.212637731024866

Time since start: 2194.703

500: time = 19.700000 loss = 6.773472 testLoss 6.299189

Jackknife: leave t_sand out

Making features

makeFeatures: max memory 259522560, total allocated 139407360, free 39624952, used 99782408, increment 20206728

Amomum_tsaoko_5 t_sand:

Regularization values: linear/quadratic/product: 0.385, categorical: 0.250, threshold: 1.770, hinge: 0.500

23 samples

Density: max memory 259522560, total allocated 139407360, free 44251712, used 95155648, increment -4626760

linearPredictor: max memory 259522560, total allocated 139407360, free 44171512, used 95235848, increment 80200

Deactivating (awc_class=2.0)

Deactivating (awc_class=3.0)

Deactivating (awc_class=4.0)

Deactivating (awc_class=6.0)

FeaturedSpace: max memory 259522560, total allocated 139407360, free 44171512, used 95235848, increment 0

Sequential: max memory 259522560, total allocated 139407360, free 44171512, used 95235848, increment 0

Initial loss: 9.212637731024866

Initial test loss: 9.212637731024866

Time since start: 2214.867

500: time = 19.849000 loss = 6.760294 testLoss 6.323526

Jackknife: only aspect

Making features

makeFeatures: max memory 259522560, total allocated 139407360, free 29072584, used 110334776, increment 15098928

Amomum_tsaoko_5 aspect:

Regularization values: linear/quadratic/product: 0.385, categorical: 0.250, threshold: 1.770, hinge: 0.500

23 samples

Density: max memory 259522560, total allocated 139407360, free 26251840, used 113155520, increment 2820744

linearPredictor: max memory 259522560, total allocated 139407360, free 26251840, used 113155520, increment 0

FeaturedSpace: max memory 259522560, total allocated 139407360, free 26251840, used 113155520, increment 0

Sequential: max memory 259522560, total allocated 139407360, free 26251840, used 113155520, increment 0

Initial loss: 9.212637731024866

Initial test loss: 9.212637731024866

Time since start: 2216.909

180: time = 1.991000 loss = 8.956515 testLoss 8.652276

Res.gain: 0.256122331579677

Jackknife: only awc_class

Making features

makeFeatures: max memory 259522560, total allocated 139407360, free 23063288, used 116344072, increment 3188552

Amomum_tsaoko_5 awc_class:

Regularization values: linear/quadratic/product: 0.385, categorical: 0.250, threshold: 1.770, hinge: 0.500

23 samples

Density: max memory 259522560, total allocated 139407360, free 23053480, used 116353880, increment 9808

linearPredictor: max memory 259522560, total allocated 139407360, free 23053480, used 116353880, increment 0

Deactivating (awc_class=2.0)

Deactivating (awc_class=3.0)

Deactivating (awc_class=4.0)

Deactivating (awc_class=6.0)

FeaturedSpace: max memory 259522560, total allocated 139407360, free 23053480, used 116353880, increment 0

Sequential: max memory 259522560, total allocated 139407360, free 23053480, used 116353880, increment 0

Initial loss: 9.212637731024866

Initial test loss: 9.212637731024866

100: time = 0.186000 loss = 9.168644 testLoss 9.122887

Res.gain: 0.04399404918563121

Jackknife: only bio_12

Making features

makeFeatures: max memory 259522560, total allocated 139407360, free 21606224, used 117801136, increment 1447256

Amomum_tsaoko_5 bio_12:

Regularization values: linear/quadratic/product: 0.385, categorical: 0.250, threshold: 1.770, hinge: 0.500

23 samples

Density: max memory 259522560, total allocated 139407360, free 20705536, used 118701824, increment 900688

linearPredictor: max memory 259522560, total allocated 139407360, free 20625336, used 118782024, increment 80200

FeaturedSpace: max memory 259522560, total allocated 139407360, free 20625336, used 118782024, increment 0

Sequential: max memory 259522560, total allocated 139407360, free 20625336, used 118782024, increment 0

Initial loss: 9.212637731024866

Initial test loss: 9.212637731024866

80: time = 0.498000 loss = 7.982779 testLoss 7.830206

Res.gain: 1.229859140368509

Jackknife: only bio_15

Making features

makeFeatures: max memory 259522560, total allocated 139407360, free 27302216, used 112105144, increment -6676880

Amomum_tsaoko_5 bio_15:

Regularization values: linear/quadratic/product: 0.385, categorical: 0.250, threshold: 1.770, hinge: 0.500

23 samples

Density: max memory 259522560, total allocated 139407360, free 24069768, used 115337592, increment 3232448

linearPredictor: max memory 259522560, total allocated 139407360, free 24069768, used 115337592, increment 0

FeaturedSpace: max memory 259522560, total allocated 139407360, free 24069768, used 115337592, increment 0

Sequential: max memory 259522560, total allocated 139407360, free 24069768, used 115337592, increment 0

Initial loss: 9.212637731024866

Initial test loss: 9.212637731024866

Time since start: 2219.952

240: time = 2.287000 loss = 8.675885 testLoss 8.653107

Res.gain: 0.5367525187465318

Jackknife: only bio_17

Making features

makeFeatures: max memory 259522560, total allocated 139407360, free 24341736, used 115065624, increment -271968

Amomum_tsaoko_5 bio_17:

Regularization values: linear/quadratic/product: 0.385, categorical: 0.250, threshold: 1.770, hinge: 0.500

23 samples

Density: max memory 259522560, total allocated 139407360, free 23401976, used 116005384, increment 939760

linearPredictor: max memory 259522560, total allocated 139407360, free 23321776, used 116085584, increment 80200

FeaturedSpace: max memory 259522560, total allocated 139407360, free 23321776, used 116085584, increment 0

Sequential: max memory 259522560, total allocated 139407360, free 23321776, used 116085584, increment 0

Initial loss: 9.212637731024866

Initial test loss: 9.212637731024866

Time since start: 2221.331

360: time = 1.338000 loss = 7.748616 testLoss 7.550922

Res.gain: 1.4640215226167426

Jackknife: only bio_4

Making features

makeFeatures: max memory 259522560, total allocated 139407360, free 42049512, used 97357848, increment -18727736

Amomum_tsaoko_5 bio_4:

Regularization values: linear/quadratic/product: 0.385, categorical: 0.250, threshold: 1.770, hinge: 0.500

23 samples

Density: max memory 259522560, total allocated 139407360, free 38996584, used 100410776, increment 3052928

linearPredictor: max memory 259522560, total allocated 139407360, free 38996584, used 100410776, increment 0

FeaturedSpace: max memory 259522560, total allocated 139407360, free 38996584, used 100410776, increment 0

Sequential: max memory 259522560, total allocated 139407360, free 38996584, used 100410776, increment 0

Initial loss: 9.212637731024866

Initial test loss: 9.212637731024866

Time since start: 2222.666

120: time = 1.304000 loss = 7.842362 testLoss 8.113283

Res.gain: 1.370275236572744

Jackknife: only bio_6

Making features

makeFeatures: max memory 259522560, total allocated 139407360, free 24574600, used 114832760, increment 14421984

Amomum_tsaoko_5 bio_6:

Regularization values: linear/quadratic/product: 0.385, categorical: 0.250, threshold: 1.770, hinge: 0.500

23 samples

Density: max memory 259522560, total allocated 139407360, free 22901056, used 116506304, increment 1673544

linearPredictor: max memory 259522560, total allocated 139407360, free 22820856, used 116586504, increment 80200

FeaturedSpace: max memory 259522560, total allocated 139407360, free 22820856, used 116586504, increment 0

Sequential: max memory 259522560, total allocated 139407360, free 22820856, used 116586504, increment 0

Initial loss: 9.212637731024866

Initial test loss: 9.212637731024866

Time since start: 2224.443

260: time = 1.749000 loss = 7.709475 testLoss 7.794352

Res.gain: 1.5031623503871625

Jackknife: only elev

Making features

makeFeatures: max memory 259522560, total allocated 139407360, free 44131576, used 95275784, increment -21310720

Amomum_tsaoko_5 elev:

Regularization values: linear/quadratic/product: 0.385, categorical: 0.250, threshold: 1.770, hinge: 0.500

23 samples

Density: max memory 259522560, total allocated 139407360, free 42539648, used 96867712, increment 1591928

linearPredictor: max memory 259522560, total allocated 139407360, free 42539648, used 96867712, increment 0

FeaturedSpace: max memory 259522560, total allocated 139407360, free 42539648, used 96867712, increment 0

Sequential: max memory 259522560, total allocated 139407360, free 42539648, used 96867712, increment 0

Initial loss: 9.212637731024866

Initial test loss: 9.212637731024866

120: time = 0.803000 loss = 9.080291 testLoss 9.053968

Res.gain: 0.13234675179986155

Jackknife: only s_caco3

Making features

makeFeatures: max memory 259522560, total allocated 139407360, free 45678320, used 93729040, increment -3138672

Amomum_tsaoko_5 s_caco3:

Regularization values: linear/quadratic/product: 0.385, categorical: 0.250, threshold: 1.770, hinge: 0.500

23 samples

Density: max memory 259522560, total allocated 139407360, free 44774416, used 94632944, increment 903904

linearPredictor: max memory 259522560, total allocated 139407360, free 44694216, used 94713144, increment 80200

FeaturedSpace: max memory 259522560, total allocated 139407360, free 44694216, used 94713144, increment 0

Sequential: max memory 259522560, total allocated 139407360, free 44694216, used 94713144, increment 0

Initial loss: 9.212637731024866

Initial test loss: 9.212637731024866

Time since start: 2225.805

80: time = 0.501000 loss = 8.755787 testLoss 8.710266

Res.gain: 0.45685058953585234

Jackknife: only s_ph_h2o

Making features

makeFeatures: max memory 259522560, total allocated 139407360, free 42647200, used 96760160, increment 2047016

Amomum_tsaoko_5 s_ph_h2o:

Regularization values: linear/quadratic/product: 0.385, categorical: 0.250, threshold: 1.770, hinge: 0.500

23 samples

Density: max memory 259522560, total allocated 139407360, free 41783888, used 97623472, increment 863312

linearPredictor: max memory 259522560, total allocated 139407360, free 41013200, used 98394160, increment 770688

FeaturedSpace: max memory 259522560, total allocated 139407360, free 41013200, used 98394160, increment 0

Sequential: max memory 259522560, total allocated 139407360, free 41013200, used 98394160, increment 0

Initial loss: 9.212637731024866

Initial test loss: 9.212637731024866

180: time = 0.604000 loss = 8.690640 testLoss 8.484955

Res.gain: 0.5219974809505548

Jackknife: only slope

Making features

makeFeatures: max memory 259522560, total allocated 139407360, free 37944208, used 101463152, increment 3068992

Amomum_tsaoko_5 slope:

Regularization values: linear/quadratic/product: 0.385, categorical: 0.250, threshold: 1.770, hinge: 0.500

23 samples

Density: max memory 259522560, total allocated 139407360, free 35539336, used 103868024, increment 2404872

linearPredictor: max memory 259522560, total allocated 139407360, free 35539336, used 103868024, increment 0

FeaturedSpace: max memory 259522560, total allocated 139407360, free 35539336, used 103868024, increment 0

Sequential: max memory 259522560, total allocated 139407360, free 35539336, used 103868024, increment 0

Initial loss: 9.212637731024866

Initial test loss: 9.212637731024866

Time since start: 2228.719

260: time = 2.267000 loss = 9.080349 testLoss 8.981977

Res.gain: 0.1322887053689179

Jackknife: only t_clay

Making features

makeFeatures: max memory 259522560, total allocated 139407360, free 26371296, used 113036064, increment 9168040

Amomum_tsaoko_5 t_clay:

Regularization values: linear/quadratic/product: 0.385, categorical: 0.250, threshold: 1.770, hinge: 0.500

23 samples

Density: max memory 259522560, total allocated 139407360, free 25583992, used 113823368, increment 787304

linearPredictor: max memory 259522560, total allocated 139407360, free 25583992, used 113823368, increment 0

FeaturedSpace: max memory 259522560, total allocated 139407360, free 25583992, used 113823368, increment 0

Sequential: max memory 259522560, total allocated 139407360, free 25583992, used 113823368, increment 0

Initial loss: 9.212637731024866

Initial test loss: 9.212637731024866

120: time = 0.602000 loss = 8.711494 testLoss 8.313579

Res.gain: 0.5011433199792457

Jackknife: only t_oc

Making features

makeFeatures: max memory 259522560, total allocated 139407360, free 22391552, used 117015808, increment 3192440

Amomum_tsaoko_5 t_oc:

Regularization values: linear/quadratic/product: 0.385, categorical: 0.250, threshold: 1.770, hinge: 0.500

23 samples

Density: max memory 259522560, total allocated 139407360, free 21567576, used 117839784, increment 823976

linearPredictor: max memory 259522560, total allocated 139407360, free 21567576, used 117839784, increment 0

FeaturedSpace: max memory 259522560, total allocated 139407360, free 21567576, used 117839784, increment 0

Sequential: max memory 259522560, total allocated 139407360, free 21567576, used 117839784, increment 0

Initial loss: 9.212637731024866

Initial test loss: 9.212637731024866

Time since start: 2230.065

160: time = 0.689000 loss = 8.738449 testLoss 8.783491

Res.gain: 0.4741889132201518

Jackknife: only t_sand

Making features

makeFeatures: max memory 259522560, total allocated 139407360, free 16746152, used 122661208, increment 4821424

Amomum_tsaoko_5 t_sand:

Regularization values: linear/quadratic/product: 0.385, categorical: 0.250, threshold: 1.770, hinge: 0.500

23 samples

Density: max memory 259522560, total allocated 139407360, free 15801984, used 123605376, increment 944168

linearPredictor: max memory 259522560, total allocated 139407360, free 15801984, used 123605376, increment 0

FeaturedSpace: max memory 259522560, total allocated 139407360, free 15801984, used 123605376, increment 0

Sequential: max memory 259522560, total allocated 139407360, free 15801984, used 123605376, increment 0

Initial loss: 9.212637731024866

Initial test loss: 9.212637731024866

140: time = 0.630000 loss = 9.044081 testLoss 8.812423

Res.gain: 0.16855636895079762

getSamples: max memory 259522560, total allocated 139407360, free 39507176, used 99900184, increment -23705192

Making features

Time since start: 2231.105

makeFeatures: max memory 259522560, total allocated 139407360, free 30606024, used 108801336, increment 8901152

Amomum_tsaoko_6:

Regularization values: linear/quadratic/product: 0.385, categorical: 0.250, threshold: 1.770, hinge: 0.500

23 samples

Density: max memory 259522560, total allocated 139407360, free 107145968, used 32261392, increment -76539944

linearPredictor: max memory 259522560, total allocated 139407360, free 107145968, used 32261392, increment 0

Deactivating (awc_class=2.0)

Deactivating (awc_class=3.0)

Deactivating (awc_class=4.0)

Deactivating (awc_class=6.0)

FeaturedSpace: max memory 259522560, total allocated 139407360, free 107145968, used 32261392, increment 0

Sequential: max memory 259522560, total allocated 139407360, free 107145968, used 32261392, increment 0

Initial loss: 9.212637731024866

Initial test loss: 9.212637731024866

Time since start: 2249.445

420: time = 18.107000 loss = 6.926018 testLoss 7.919205

Resulting gain: 2.2866196276377977

Projecting...

Writing file E:\Amomum_tsaoko\results2\Amomum_tsaoko_6.asc

Time since start: 2253.875

Writing E:\Amomum_tsaoko\results2\plots\Amomum_tsaoko_6.png

Time since start: 2255.389

Projecting...

Writing file E:\Amomum_tsaoko\results2\Amomum_tsaoko_6_LGM.asc

Writing file E:\Amomum_tsaoko\results2\Amomum_tsaoko_6_LGM_clamping.asc

Time since start: 2260.352

Writing E:\Amomum_tsaoko\results2\plots\Amomum_tsaoko_6_LGM.png

Time since start: 2261.761

Writing E:\Amomum_tsaoko\results2\plots\Amomum_tsaoko_6_LGM_clamping.png

Time since start: 2263.186

Writing file E:\Amomum_tsaoko\results2\Amomum_tsaoko_6_LGM_novel.asc

Writing file E:\Amomum_tsaoko\results2\Amomum_tsaoko_6_LGM_novel_limiting.asc

Time since start: 2267.738

Projecting...

Writing file E:\Amomum_tsaoko\results2\Amomum_tsaoko_6_MH.asc

Writing file E:\Amomum_tsaoko\results2\Amomum_tsaoko_6_MH_clamping.asc

Time since start: 2272.443

Writing E:\Amomum_tsaoko\results2\plots\Amomum_tsaoko_6_MH.png

Time since start: 2273.821

Writing E:\Amomum_tsaoko\results2\plots\Amomum_tsaoko_6_MH_clamping.png

Time since start: 2275.308

Writing file E:\Amomum_tsaoko\results2\Amomum_tsaoko_6_MH_novel.asc

Writing file E:\Amomum_tsaoko\results2\Amomum_tsaoko_6_MH_novel_limiting.asc

Time since start: 2279.787

Projecting...

Writing file E:\Amomum_tsaoko\results2\Amomum_tsaoko_6_SSP126-2050S.asc

Writing file E:\Amomum_tsaoko\results2\Amomum_tsaoko_6_SSP126-2050S_clamping.asc

Time since start: 2283.715

Writing E:\Amomum_tsaoko\results2\plots\Amomum_tsaoko_6_SSP126-2050S.png

Time since start: 2285.109

Writing E:\Amomum_tsaoko\results2\plots\Amomum_tsaoko_6_SSP126-2050S_clamping.png

Time since start: 2286.457

Writing file E:\Amomum_tsaoko\results2\Amomum_tsaoko_6_SSP126-2050S_novel.asc

Writing file E:\Amomum_tsaoko\results2\Amomum_tsaoko_6_SSP126-2050S_novel_limiting.asc

Time since start: 2290.873

Projecting...

Writing file E:\Amomum_tsaoko\results2\Amomum_tsaoko_6_SSP126-2090S.asc

Writing file E:\Amomum_tsaoko\results2\Amomum_tsaoko_6_SSP126-2090S_clamping.asc

Time since start: 2294.676

Writing E:\Amomum_tsaoko\results2\plots\Amomum_tsaoko_6_SSP126-2090S.png

Time since start: 2296.068

Writing E:\Amomum_tsaoko\results2\plots\Amomum_tsaoko_6_SSP126-2090S_clamping.png

Time since start: 2297.431

Writing file E:\Amomum_tsaoko\results2\Amomum_tsaoko_6_SSP126-2090S_novel.asc

Writing file E:\Amomum_tsaoko\results2\Amomum_tsaoko_6_SSP126-2090S_novel_limiting.asc

Time since start: 2301.912

Projecting...

Writing file E:\Amomum_tsaoko\results2\Amomum_tsaoko_6_SSP585-2050S.asc

Writing file E:\Amomum_tsaoko\results2\Amomum_tsaoko_6_SSP585-2050S_clamping.asc

Time since start: 2305.84

Writing E:\Amomum_tsaoko\results2\plots\Amomum_tsaoko_6_SSP585-2050S.png

Time since start: 2307.217

Writing E:\Amomum_tsaoko\results2\plots\Amomum_tsaoko_6_SSP585-2050S_clamping.png

Time since start: 2308.593

Writing file E:\Amomum_tsaoko\results2\Amomum_tsaoko_6_SSP585-2050S_novel.asc

Writing file E:\Amomum_tsaoko\results2\Amomum_tsaoko_6_SSP585-2050S_novel_limiting.asc

Time since start: 2313.173

Projecting...

Writing file E:\Amomum_tsaoko\results2\Amomum_tsaoko_6_SSP585-2090S.asc

Writing file E:\Amomum_tsaoko\results2\Amomum_tsaoko_6_SSP585-2090S_clamping.asc

Time since start: 2317.09

Writing E:\Amomum_tsaoko\results2\plots\Amomum_tsaoko_6_SSP585-2090S.png

Time since start: 2318.522

Writing E:\Amomum_tsaoko\results2\plots\Amomum_tsaoko_6_SSP585-2090S_clamping.png

Time since start: 2319.981

Writing file E:\Amomum_tsaoko\results2\Amomum_tsaoko_6_SSP585-2090S_novel.asc

Writing file E:\Amomum_tsaoko\results2\Amomum_tsaoko_6_SSP585-2090S_novel_limiting.asc

Time since start: 2324.418

Amomum_tsaoko_6 response curves

Response curve: only aspect

Making features

makeFeatures: max memory 259522560, total allocated 139407360, free 67250528, used 72156832, increment 39895440

Amomum_tsaoko_6 aspect:

Regularization values: linear/quadratic/product: 0.385, categorical: 0.250, threshold: 1.770, hinge: 0.500

23 samples

Density: max memory 259522560, total allocated 139407360, free 64658368, used 74748992, increment 2592160

linearPredictor: max memory 259522560, total allocated 139407360, free 64658368, used 74748992, increment 0

FeaturedSpace: max memory 259522560, total allocated 139407360, free 64658368, used 74748992, increment 0

Sequential: max memory 259522560, total allocated 139407360, free 64658368, used 74748992, increment 0

Initial loss: 9.212637731024866

Time since start: 2327.286

180: time = 1.959000 loss = 9.071740

Resulting gain: 0.14089821353594267

Amomum_tsaoko_6 response curves

Response curve: only awc_class

Making features

makeFeatures: max memory 259522560, total allocated 139407360, free 59251592, used 80155768, increment 5406776

Amomum_tsaoko_6 awc_class:

Regularization values: linear/quadratic/product: 0.385, categorical: 0.250, threshold: 1.770, hinge: 0.500

23 samples

Density: max memory 259522560, total allocated 139407360, free 59240720, used 80166640, increment 10872

linearPredictor: max memory 259522560, total allocated 139407360, free 59240720, used 80166640, increment 0

Deactivating (awc_class=2.0)

Deactivating (awc_class=3.0)

Deactivating (awc_class=4.0)

Deactivating (awc_class=6.0)

FeaturedSpace: max memory 259522560, total allocated 139407360, free 59240720, used 80166640, increment 0

Sequential: max memory 259522560, total allocated 139407360, free 59240720, used 80166640, increment 0

Initial loss: 9.212637731024866

100: time = 0.204000 loss = 9.121152

Resulting gain: 0.09148580031358833

Amomum_tsaoko_6 response curves

Response curve: only bio_12

Making features

makeFeatures: max memory 259522560, total allocated 139407360, free 54494984, used 84912376, increment 4745736

Amomum_tsaoko_6 bio_12:

Regularization values: linear/quadratic/product: 0.385, categorical: 0.250, threshold: 1.770, hinge: 0.500

23 samples

Density: max memory 259522560, total allocated 139407360, free 52942776, used 86464584, increment 1552208

linearPredictor: max memory 259522560, total allocated 139407360, free 52942776, used 86464584, increment 0

FeaturedSpace: max memory 259522560, total allocated 139407360, free 52942776, used 86464584, increment 0

Sequential: max memory 259522560, total allocated 139407360, free 52942776, used 86464584, increment 0

Initial loss: 9.212637731024866

Time since start: 2328.504

160: time = 0.841000 loss = 8.108887

Resulting gain: 1.1037507806625904

Amomum_tsaoko_6 response curves

Response curve: only bio_15

Making features

makeFeatures: max memory 259522560, total allocated 139407360, free 62954336, used 76453024, increment -10011560

Amomum_tsaoko_6 bio_15:

Regularization values: linear/quadratic/product: 0.385, categorical: 0.250, threshold: 1.770, hinge: 0.500

23 samples

Density: max memory 259522560, total allocated 139407360, free 60381632, used 79025728, increment 2572704

linearPredictor: max memory 259522560, total allocated 139407360, free 60381632, used 79025728, increment 0

FeaturedSpace: max memory 259522560, total allocated 139407360, free 60381632, used 79025728, increment 0

Sequential: max memory 259522560, total allocated 139407360, free 60381632, used 79025728, increment 0

Initial loss: 9.212637731024866

Time since start: 2330.736

220: time = 2.134000 loss = 8.814387

Resulting gain: 0.39825104087677055

Amomum_tsaoko_6 response curves

Response curve: only bio_17

Making features

makeFeatures: max memory 259522560, total allocated 139407360, free 56108792, used 83298568, increment 4272840

Amomum_tsaoko_6 bio_17:

Regularization values: linear/quadratic/product: 0.385, categorical: 0.250, threshold: 1.770, hinge: 0.500

23 samples

Density: max memory 259522560, total allocated 139407360, free 55321848, used 84085512, increment 786944

linearPredictor: max memory 259522560, total allocated 139407360, free 55321848, used 84085512, increment 0

FeaturedSpace: max memory 259522560, total allocated 139407360, free 55321848, used 84085512, increment 0

Sequential: max memory 259522560, total allocated 139407360, free 55321848, used 84085512, increment 0

Initial loss: 9.212637731024866

Time since start: 2332.162

400: time = 1.332000 loss = 7.917400

Resulting gain: 1.2952378124594244

Amomum_tsaoko_6 response curves

Response curve: only bio_4

Making features

makeFeatures: max memory 259522560, total allocated 139407360, free 67690072, used 71717288, increment -12368224

Amomum_tsaoko_6 bio_4:

Regularization values: linear/quadratic/product: 0.385, categorical: 0.250, threshold: 1.770, hinge: 0.500

23 samples

Density: max memory 259522560, total allocated 139407360, free 64672264, used 74735096, increment 3017808

linearPredictor: max memory 259522560, total allocated 139407360, free 64672264, used 74735096, increment 0

FeaturedSpace: max memory 259522560, total allocated 139407360, free 64672264, used 74735096, increment 0

Sequential: max memory 259522560, total allocated 139407360, free 64672264, used 74735096, increment 0

Initial loss: 9.212637731024866

Time since start: 2333.71

140: time = 1.439000 loss = 7.900645

Resulting gain: 1.3119926205194146

Amomum_tsaoko_6 response curves

Response curve: only bio_6

Making features

makeFeatures: max memory 259522560, total allocated 139407360, free 43741424, used 95665936, increment 20930840

Amomum_tsaoko_6 bio_6:

Regularization values: linear/quadratic/product: 0.385, categorical: 0.250, threshold: 1.770, hinge: 0.500

23 samples

Density: max memory 259522560, total allocated 139407360, free 41320232, used 98087128, increment 2421192

linearPredictor: max memory 259522560, total allocated 139407360, free 41320232, used 98087128, increment 0

FeaturedSpace: max memory 259522560, total allocated 139407360, free 41320232, used 98087128, increment 0

Sequential: max memory 259522560, total allocated 139407360, free 41320232, used 98087128, increment 0

Initial loss: 9.212637731024866

Time since start: 2335.197

180: time = 1.378000 loss = 7.999799

Resulting gain: 1.2128389479478185

Amomum_tsaoko_6 response curves

Response curve: only elev

Making features

makeFeatures: max memory 259522560, total allocated 139407360, free 44032944, used 95374416, increment -2712712

Amomum_tsaoko_6 elev:

Regularization values: linear/quadratic/product: 0.385, categorical: 0.250, threshold: 1.770, hinge: 0.500

23 samples

Density: max memory 259522560, total allocated 139407360, free 42357080, used 97050280, increment 1675864

linearPredictor: max memory 259522560, total allocated 139407360, free 42357080, used 97050280, increment 0

FeaturedSpace: max memory 259522560, total allocated 139407360, free 42357080, used 97050280, increment 0

Sequential: max memory 259522560, total allocated 139407360, free 42357080, used 97050280, increment 0

Initial loss: 9.212637731024866

120: time = 0.720000 loss = 9.047861

Resulting gain: 0.16477691544985973

Amomum_tsaoko_6 response curves

Response curve: only s_caco3

Making features

makeFeatures: max memory 259522560, total allocated 139407360, free 41795384, used 97611976, increment 561696

Amomum_tsaoko_6 s_caco3:

Regularization values: linear/quadratic/product: 0.385, categorical: 0.250, threshold: 1.770, hinge: 0.500

23 samples

Density: max memory 259522560, total allocated 139407360, free 40931656, used 98475704, increment 863728

linearPredictor: max memory 259522560, total allocated 139407360, free 40931656, used 98475704, increment 0

FeaturedSpace: max memory 259522560, total allocated 139407360, free 40931656, used 98475704, increment 0

Sequential: max memory 259522560, total allocated 139407360, free 40931656, used 98475704, increment 0

Initial loss: 9.212637731024866

Time since start: 2336.574

80: time = 0.470000 loss = 8.474385

Resulting gain: 0.7382529746755342

Amomum_tsaoko_6 response curves

Response curve: only s_ph_h2o

Making features

makeFeatures: max memory 259522560, total allocated 139407360, free 35454432, used 103952928, increment 5477224

Amomum_tsaoko_6 s_ph_h2o:

Regularization values: linear/quadratic/product: 0.385, categorical: 0.250, threshold: 1.770, hinge: 0.500

23 samples

Density: max memory 259522560, total allocated 139407360, free 34504376, used 104902984, increment 950056

linearPredictor: max memory 259522560, total allocated 139407360, free 73264816, used 66142544, increment -38760440

FeaturedSpace: max memory 259522560, total allocated 139407360, free 72504952, used 66902408, increment 759864

Sequential: max memory 259522560, total allocated 139407360, free 72504952, used 66902408, increment 0

Initial loss: 9.212637731024866

Time since start: 2337.576

260: time = 0.907000 loss = 8.517128

Resulting gain: 0.6955098321357287

Amomum_tsaoko_6 response curves

Response curve: only slope

Making features

makeFeatures: max memory 259522560, total allocated 139407360, free 64398008, used 75009352, increment 8106944

Amomum_tsaoko_6 slope:

Regularization values: linear/quadratic/product: 0.385, categorical: 0.250, threshold: 1.770, hinge: 0.500

23 samples

Density: max memory 259522560, total allocated 139407360, free 61790400, used 77616960, increment 2607608

linearPredictor: max memory 259522560, total allocated 139407360, free 61790400, used 77616960, increment 0

FeaturedSpace: max memory 259522560, total allocated 139407360, free 61790400, used 77616960, increment 0

Sequential: max memory 259522560, total allocated 139407360, free 61790400, used 77616960, increment 0

Initial loss: 9.212637731024866

Time since start: 2339.516

200: time = 1.846000 loss = 9.074977

Resulting gain: 0.13766102989559847

Amomum_tsaoko_6 response curves

Response curve: only t_clay

Making features

makeFeatures: max memory 259522560, total allocated 139407360, free 44066304, used 95341056, increment 17724096

Amomum_tsaoko_6 t_clay:

Regularization values: linear/quadratic/product: 0.385, categorical: 0.250, threshold: 1.770, hinge: 0.500

23 samples

Density: max memory 259522560, total allocated 139407360, free 43163240, used 96244120, increment 903064

linearPredictor: max memory 259522560, total allocated 139407360, free 43163240, used 96244120, increment 0

FeaturedSpace: max memory 259522560, total allocated 139407360, free 43163240, used 96244120, increment 0

Sequential: max memory 259522560, total allocated 139407360, free 43163240, used 96244120, increment 0

Initial loss: 9.212637731024866

100: time = 0.475000 loss = 8.432537

Resulting gain: 0.78010050037593

Amomum_tsaoko_6 response curves

Response curve: only t_oc

Making features

makeFeatures: max memory 259522560, total allocated 139407360, free 36868392, used 102538968, increment 6294848

Amomum_tsaoko_6 t_oc:

Regularization values: linear/quadratic/product: 0.385, categorical: 0.250, threshold: 1.770, hinge: 0.500

23 samples

Density: max memory 259522560, total allocated 139407360, free 36085728, used 103321632, increment 782664

linearPredictor: max memory 259522560, total allocated 139407360, free 36085728, used 103321632, increment 0

FeaturedSpace: max memory 259522560, total allocated 139407360, free 36085728, used 103321632, increment 0

Sequential: max memory 259522560, total allocated 139407360, free 36085728, used 103321632, increment 0

Initial loss: 9.212637731024866

100: time = 0.312000 loss = 8.842995

Resulting gain: 0.3696423036904122

Amomum_tsaoko_6 response curves

Time since start: 2340.565

Response curve: only t_sand

Making features

makeFeatures: max memory 259522560, total allocated 139407360, free 67554256, used 71853104, increment -31468528

Amomum_tsaoko_6 t_sand:

Regularization values: linear/quadratic/product: 0.385, categorical: 0.250, threshold: 1.770, hinge: 0.500

23 samples

Density: max memory 259522560, total allocated 139407360, free 66752312, used 72655048, increment 801944

linearPredictor: max memory 259522560, total allocated 139407360, free 66752312, used 72655048, increment 0

FeaturedSpace: max memory 259522560, total allocated 139407360, free 66752312, used 72655048, increment 0

Sequential: max memory 259522560, total allocated 139407360, free 66752312, used 72655048, increment 0

Initial loss: 9.212637731024866

160: time = 0.688000 loss = 8.846065

Resulting gain: 0.36657273854514116

Amomum_tsaoko_6 response curves

Time since start: 2342.568

Jackknife: leave aspect out

Making features

makeFeatures: max memory 259522560, total allocated 139407360, free 49593992, used 89813368, increment 17158320

Amomum_tsaoko_6 aspect:

Regularization values: linear/quadratic/product: 0.385, categorical: 0.250, threshold: 1.770, hinge: 0.500

23 samples

Density: max memory 259522560, total allocated 139407360, free 56768768, used 82638592, increment -7174776

linearPredictor: max memory 259522560, total allocated 139407360, free 56768768, used 82638592, increment 0

Deactivating (awc_class=2.0)

Deactivating (awc_class=3.0)

Deactivating (awc_class=4.0)

Deactivating (awc_class=6.0)

FeaturedSpace: max memory 259522560, total allocated 139407360, free 56768768, used 82638592, increment 0

Sequential: max memory 259522560, total allocated 139407360, free 56768768, used 82638592, increment 0

Initial loss: 9.212637731024866

Initial test loss: 9.212637731024866

Time since start: 2357.263

420: time = 14.396000 loss = 6.972888 testLoss 7.978664

Jackknife: leave awc_class out

Making features

makeFeatures: max memory 259522560, total allocated 139407360, free 35075448, used 104331912, increment 21693320

Amomum_tsaoko_6 awc_class:

Regularization values: linear/quadratic/product: 0.385, categorical: 0.250, threshold: 1.770, hinge: 0.500

23 samples

Density: max memory 259522560, total allocated 139407360, free 35750216, used 103657144, increment -674768

linearPredictor: max memory 259522560, total allocated 139407360, free 35750216, used 103657144, increment 0

FeaturedSpace: max memory 259522560, total allocated 139407360, free 35750216, used 103657144, increment 0

Sequential: max memory 259522560, total allocated 139407360, free 35750216, used 103657144, increment 0

Initial loss: 9.212637731024866

Initial test loss: 9.212637731024866

Time since start: 2374.11

400: time = 16.498000 loss = 6.934862 testLoss 7.896729

Jackknife: leave bio_12 out

Making features

makeFeatures: max memory 259522560, total allocated 139407360, free 74802744, used 64604616, increment -39052528

Amomum_tsaoko_6 bio_12:

Regularization values: linear/quadratic/product: 0.385, categorical: 0.250, threshold: 1.770, hinge: 0.500

23 samples

Density: max memory 259522560, total allocated 139407360, free 78841288, used 60566072, increment -4038544

linearPredictor: max memory 259522560, total allocated 139407360, free 78761088, used 60646272, increment 80200

Deactivating (awc_class=2.0)

Deactivating (awc_class=3.0)

Deactivating (awc_class=4.0)

Deactivating (awc_class=6.0)

FeaturedSpace: max memory 259522560, total allocated 139407360, free 78761088, used 60646272, increment 0

Sequential: max memory 259522560, total allocated 139407360, free 78761088, used 60646272, increment 0

Initial loss: 9.212637731024866

Initial test loss: 9.212637731024866

Time since start: 2392.681

480: time = 18.258000 loss = 6.957694 testLoss 7.843544

Jackknife: leave bio_15 out

Making features

makeFeatures: max memory 259522560, total allocated 139407360, free 57955056, used 81452304, increment 20806032

Amomum_tsaoko_6 bio_15:

Regularization values: linear/quadratic/product: 0.385, categorical: 0.250, threshold: 1.770, hinge: 0.500

23 samples

Density: max memory 259522560, total allocated 139407360, free 63977304, used 75430056, increment -6022248

linearPredictor: max memory 259522560, total allocated 139407360, free 63977304, used 75430056, increment 0

Deactivating (awc_class=2.0)

Deactivating (awc_class=3.0)

Deactivating (awc_class=4.0)

Deactivating (awc_class=6.0)

FeaturedSpace: max memory 259522560, total allocated 139407360, free 63977304, used 75430056, increment 0

Sequential: max memory 259522560, total allocated 139407360, free 63977304, used 75430056, increment 0

Initial loss: 9.212637731024866

Initial test loss: 9.212637731024866

Time since start: 2409.133

500: time = 16.157000 loss = 6.946660 testLoss 7.894139

Jackknife: leave bio_17 out

Making features

makeFeatures: max memory 259522560, total allocated 139407360, free 58690832, used 80716528, increment 5286472

Amomum_tsaoko_6 bio_17:

Regularization values: linear/quadratic/product: 0.385, categorical: 0.250, threshold: 1.770, hinge: 0.500

23 samples

Density: max memory 259522560, total allocated 139407360, free 38494680, used 100912680, increment 20196152

linearPredictor: max memory 259522560, total allocated 139407360, free 38414480, used 100992880, increment 80200

Deactivating (awc_class=2.0)

Deactivating (awc_class=3.0)

Deactivating (awc_class=4.0)

Deactivating (awc_class=6.0)

FeaturedSpace: max memory 259522560, total allocated 139407360, free 38414480, used 100992880, increment 0

Sequential: max memory 259522560, total allocated 139407360, free 38414480, used 100992880, increment 0

Initial loss: 9.212637731024866

Initial test loss: 9.212637731024866

Time since start: 2424.582

380: time = 15.152000 loss = 6.968104 testLoss 8.025896

Jackknife: leave bio_4 out

Making features

makeFeatures: max memory 259522560, total allocated 139407360, free 23014168, used 116393192, increment 15400312

Amomum_tsaoko_6 bio_4:

Regularization values: linear/quadratic/product: 0.385, categorical: 0.250, threshold: 1.770, hinge: 0.500

23 samples

Density: max memory 259522560, total allocated 139407360, free 90487296, used 48920064, increment -67473128

linearPredictor: max memory 259522560, total allocated 139407360, free 90487296, used 48920064, increment 0

Deactivating (awc_class=2.0)

Deactivating (awc_class=3.0)

Deactivating (awc_class=4.0)

Deactivating (awc_class=6.0)

FeaturedSpace: max memory 259522560, total allocated 139407360, free 90487296, used 48920064, increment 0

Sequential: max memory 259522560, total allocated 139407360, free 90487296, used 48920064, increment 0

Initial loss: 9.212637731024866

Initial test loss: 9.212637731024866

Time since start: 2438.724

420: time = 13.818000 loss = 6.979509 testLoss 8.034063

Jackknife: leave bio_6 out

Making features

makeFeatures: max memory 259522560, total allocated 139407360, free 89064776, used 50342584, increment 1422520

Amomum_tsaoko_6 bio_6:

Regularization values: linear/quadratic/product: 0.385, categorical: 0.250, threshold: 1.770, hinge: 0.500

23 samples

Density: max memory 259522560, total allocated 139407360, free 69701408, used 69705952, increment 19363368

linearPredictor: max memory 259522560, total allocated 139407360, free 69701408, used 69705952, increment 0

Deactivating (awc_class=2.0)

Deactivating (awc_class=3.0)

Deactivating (awc_class=4.0)

Deactivating (awc_class=6.0)

FeaturedSpace: max memory 259522560, total allocated 139407360, free 69701408, used 69705952, increment 0

Sequential: max memory 259522560, total allocated 139407360, free 69701408, used 69705952, increment 0

Initial loss: 9.212637731024866

Initial test loss: 9.212637731024866

Time since start: 2454.588

440: time = 15.556000 loss = 6.933313 testLoss 7.888391

Jackknife: leave elev out

Making features

makeFeatures: max memory 259522560, total allocated 139407360, free 64233648, used 75173712, increment 5467760

Amomum_tsaoko_6 elev:

Regularization values: linear/quadratic/product: 0.385, categorical: 0.250, threshold: 1.770, hinge: 0.500

23 samples

Density: max memory 259522560, total allocated 139407360, free 44622408, used 94784952, increment 19611240

linearPredictor: max memory 259522560, total allocated 139407360, free 44542208, used 94865152, increment 80200

Deactivating (awc_class=2.0)

Deactivating (awc_class=3.0)

Deactivating (awc_class=4.0)

Deactivating (awc_class=6.0)

FeaturedSpace: max memory 259522560, total allocated 139407360, free 44542208, used 94865152, increment 0

Sequential: max memory 259522560, total allocated 139407360, free 44542208, used 94865152, increment 0

Initial loss: 9.212637731024866

Initial test loss: 9.212637731024866

Time since start: 2469.636

400: time = 14.760000 loss = 6.971650 testLoss 7.784010

Jackknife: leave s_caco3 out

Making features

makeFeatures: max memory 259522560, total allocated 139407360, free 33560448, used 105846912, increment 10981760

Amomum_tsaoko_6 s_caco3:

Regularization values: linear/quadratic/product: 0.385, categorical: 0.250, threshold: 1.770, hinge: 0.500

23 samples

Density: max memory 259522560, total allocated 139407360, free 41763048, used 97644312, increment -8202600

linearPredictor: max memory 259522560, total allocated 139407360, free 41763048, used 97644312, increment 0

Deactivating (awc_class=2.0)

Deactivating (awc_class=3.0)

Deactivating (awc_class=4.0)

Deactivating (awc_class=6.0)

FeaturedSpace: max memory 259522560, total allocated 139407360, free 41763048, used 97644312, increment 0

Sequential: max memory 259522560, total allocated 139407360, free 41763048, used 97644312, increment 0

Initial loss: 9.212637731024866

Initial test loss: 9.212637731024866

Time since start: 2489.405

500: time = 19.445000 loss = 6.993139 testLoss 7.911774

Jackknife: leave s_ph_h2o out

Making features

makeFeatures: max memory 259522560, total allocated 139407360, free 45870816, used 93536544, increment -4107768

Amomum_tsaoko_6 s_ph_h2o:

Regularization values: linear/quadratic/product: 0.385, categorical: 0.250, threshold: 1.770, hinge: 0.500

23 samples

Density: max memory 259522560, total allocated 139407360, free 25276120, used 114131240, increment 20594696

linearPredictor: max memory 259522560, total allocated 139407360, free 25276120, used 114131240, increment 0

Deactivating (awc_class=2.0)

Deactivating (awc_class=3.0)

Deactivating (awc_class=4.0)

Deactivating (awc_class=6.0)

FeaturedSpace: max memory 259522560, total allocated 139407360, free 25276120, used 114131240, increment 0

Sequential: max memory 259522560, total allocated 139407360, free 25276120, used 114131240, increment 0

Initial loss: 9.212637731024866

Initial test loss: 9.212637731024866

Time since start: 2509.734

480: time = 20.030000 loss = 6.926031 testLoss 7.920062

Jackknife: leave slope out

Making features

makeFeatures: max memory 259522560, total allocated 139407360, free 73971240, used 65436120, increment -48695120

Amomum_tsaoko_6 slope:

Regularization values: linear/quadratic/product: 0.385, categorical: 0.250, threshold: 1.770, hinge: 0.500

23 samples

Density: max memory 259522560, total allocated 139407360, free 79159896, used 60247464, increment -5188656

linearPredictor: max memory 259522560, total allocated 139407360, free 79159896, used 60247464, increment 0

Deactivating (awc_class=2.0)

Deactivating (awc_class=3.0)

Deactivating (awc_class=4.0)

Deactivating (awc_class=6.0)

FeaturedSpace: max memory 259522560, total allocated 139407360, free 79159896, used 60247464, increment 0

Sequential: max memory 259522560, total allocated 139407360, free 79159896, used 60247464, increment 0

Initial loss: 9.212637731024866

Initial test loss: 9.212637731024866

Time since start: 2523.974

380: time = 13.895000 loss = 7.006191 testLoss 7.926331

Jackknife: leave t_clay out

Making features

makeFeatures: max memory 259522560, total allocated 139407360, free 49788376, used 89618984, increment 29371520

Amomum_tsaoko_6 t_clay:

Regularization values: linear/quadratic/product: 0.385, categorical: 0.250, threshold: 1.770, hinge: 0.500

23 samples

Density: max memory 259522560, total allocated 139407360, free 56726728, used 82680632, increment -6938352

linearPredictor: max memory 259522560, total allocated 139407360, free 56726728, used 82680632, increment 0

Deactivating (awc_class=2.0)

Deactivating (awc_class=3.0)

Deactivating (awc_class=4.0)

Deactivating (awc_class=6.0)

FeaturedSpace: max memory 259522560, total allocated 139407360, free 56726728, used 82680632, increment 0

Sequential: max memory 259522560, total allocated 139407360, free 56726728, used 82680632, increment 0

Initial loss: 9.212637731024866

Initial test loss: 9.212637731024866

Time since start: 2540.956

420: time = 16.686000 loss = 6.926075 testLoss 7.917644

Jackknife: leave t_oc out

Making features

makeFeatures: max memory 259522560, total allocated 139407360, free 59637208, used 79770152, increment -2910480

Amomum_tsaoko_6 t_oc:

Regularization values: linear/quadratic/product: 0.385, categorical: 0.250, threshold: 1.770, hinge: 0.500

23 samples

Density: max memory 259522560, total allocated 139407360, free 38910440, used 100496920, increment 20726768

linearPredictor: max memory 259522560, total allocated 139407360, free 38910440, used 100496920, increment 0

Deactivating (awc_class=2.0)

Deactivating (awc_class=3.0)

Deactivating (awc_class=4.0)

Deactivating (awc_class=6.0)

FeaturedSpace: max memory 259522560, total allocated 139407360, free 38910440, used 100496920, increment 0

Sequential: max memory 259522560, total allocated 139407360, free 38910440, used 100496920, increment 0

Initial loss: 9.212637731024866

Initial test loss: 9.212637731024866

Time since start: 2556.753

380: time = 15.479000 loss = 6.929595 testLoss 7.951113

Jackknife: leave t_sand out

Making features

makeFeatures: max memory 259522560, total allocated 139407360, free 44850624, used 94556736, increment -5940184

Amomum_tsaoko_6 t_sand:

Regularization values: linear/quadratic/product: 0.385, categorical: 0.250, threshold: 1.770, hinge: 0.500

23 samples

Density: max memory 259522560, total allocated 139407360, free 24656256, used 114751104, increment 20194368

linearPredictor: max memory 259522560, total allocated 139407360, free 23892480, used 115514880, increment 763776

Deactivating (awc_class=2.0)

Deactivating (awc_class=3.0)

Deactivating (awc_class=4.0)

Deactivating (awc_class=6.0)

FeaturedSpace: max memory 259522560, total allocated 139407360, free 23892480, used 115514880, increment 0

Sequential: max memory 259522560, total allocated 139407360, free 23892480, used 115514880, increment 0

Initial loss: 9.212637731024866

Initial test loss: 9.212637731024866

Time since start: 2578.059

500: time = 21.010000 loss = 6.967706 testLoss 7.839581

Jackknife: only aspect

Making features

makeFeatures: max memory 259522560, total allocated 139407360, free 58750344, used 80657016, increment -34857864

Amomum_tsaoko_6 aspect:

Regularization values: linear/quadratic/product: 0.385, categorical: 0.250, threshold: 1.770, hinge: 0.500

23 samples

Density: max memory 259522560, total allocated 139407360, free 92971600, used 46435760, increment -34221256

linearPredictor: max memory 259522560, total allocated 139407360, free 92971600, used 46435760, increment 0

FeaturedSpace: max memory 259522560, total allocated 139407360, free 92971600, used 46435760, increment 0

Sequential: max memory 259522560, total allocated 139407360, free 92971600, used 46435760, increment 0

Initial loss: 9.212637731024866

Initial test loss: 9.212637731024866

Time since start: 2580.294

180: time = 2.170000 loss = 9.071740 testLoss 9.056873

Res.gain: 0.14089821353594267

Jackknife: only awc_class

Making features

makeFeatures: max memory 259522560, total allocated 139407360, free 91337736, used 48069624, increment 1633864

Amomum_tsaoko_6 awc_class:

Regularization values: linear/quadratic/product: 0.385, categorical: 0.250, threshold: 1.770, hinge: 0.500

23 samples

Density: max memory 259522560, total allocated 139407360, free 91327416, used 48079944, increment 10320

linearPredictor: max memory 259522560, total allocated 139407360, free 91327416, used 48079944, increment 0

Deactivating (awc_class=2.0)

Deactivating (awc_class=3.0)

Deactivating (awc_class=4.0)

Deactivating (awc_class=6.0)

FeaturedSpace: max memory 259522560, total allocated 139407360, free 91327416, used 48079944, increment 0

Sequential: max memory 259522560, total allocated 139407360, free 91327416, used 48079944, increment 0

Initial loss: 9.212637731024866

Initial test loss: 9.212637731024866

100: time = 0.214000 loss = 9.121152 testLoss 9.125823

Res.gain: 0.09148580031358833

Jackknife: only bio_12

Making features

makeFeatures: max memory 259522560, total allocated 139407360, free 89945672, used 49461688, increment 1381744

Amomum_tsaoko_6 bio_12:

Regularization values: linear/quadratic/product: 0.385, categorical: 0.250, threshold: 1.770, hinge: 0.500

23 samples

Density: max memory 259522560, total allocated 139407360, free 88313504, used 51093856, increment 1632168

linearPredictor: max memory 259522560, total allocated 139407360, free 88313504, used 51093856, increment 0

FeaturedSpace: max memory 259522560, total allocated 139407360, free 88313504, used 51093856, increment 0

Sequential: max memory 259522560, total allocated 139407360, free 88313504, used 51093856, increment 0

Initial loss: 9.212637731024866

Initial test loss: 9.212637731024866

Time since start: 2581.503

160: time = 0.959000 loss = 8.108887 testLoss 8.158104

Res.gain: 1.1037507806625904

Jackknife: only bio_15

Making features

makeFeatures: max memory 259522560, total allocated 139407360, free 64549760, used 74857600, increment 23763744

Amomum_tsaoko_6 bio_15:

Regularization values: linear/quadratic/product: 0.385, categorical: 0.250, threshold: 1.770, hinge: 0.500

23 samples

Density: max memory 259522560, total allocated 139407360, free 61319936, used 78087424, increment 3229824

linearPredictor: max memory 259522560, total allocated 139407360, free 61319936, used 78087424, increment 0

FeaturedSpace: max memory 259522560, total allocated 139407360, free 61319936, used 78087424, increment 0

Sequential: max memory 259522560, total allocated 139407360, free 61319936, used 78087424, increment 0

Initial loss: 9.212637731024866

Initial test loss: 9.212637731024866

Time since start: 2584.191

220: time = 2.651000 loss = 8.814387 testLoss 8.778357

Res.gain: 0.39825104087677055

Jackknife: only bio_17

Making features

makeFeatures: max memory 259522560, total allocated 139407360, free 60937664, used 78469696, increment 382272

Amomum_tsaoko_6 bio_17:

Regularization values: linear/quadratic/product: 0.385, categorical: 0.250, threshold: 1.770, hinge: 0.500

23 samples

Density: max memory 259522560, total allocated 139407360, free 59954528, used 79452832, increment 983136

linearPredictor: max memory 259522560, total allocated 139407360, free 59874328, used 79533032, increment 80200

FeaturedSpace: max memory 259522560, total allocated 139407360, free 59874328, used 79533032, increment 0

Sequential: max memory 259522560, total allocated 139407360, free 59874328, used 79533032, increment 0

Initial loss: 9.212637731024866

Initial test loss: 9.212637731024866

Time since start: 2585.841

400: time = 1.622000 loss = 7.917400 testLoss 7.868916

Res.gain: 1.2952378124594244

Jackknife: only bio_4

Making features

makeFeatures: max memory 259522560, total allocated 139407360, free 76211400, used 63195960, increment -16337072

Amomum_tsaoko_6 bio_4:

Regularization values: linear/quadratic/product: 0.385, categorical: 0.250, threshold: 1.770, hinge: 0.500

23 samples

Density: max memory 259522560, total allocated 139407360, free 73005160, used 66402200, increment 3206240

linearPredictor: max memory 259522560, total allocated 139407360, free 73005160, used 66402200, increment 0

FeaturedSpace: max memory 259522560, total allocated 139407360, free 73005160, used 66402200, increment 0

Sequential: max memory 259522560, total allocated 139407360, free 73005160, used 66402200, increment 0

Initial loss: 9.212637731024866

Initial test loss: 9.212637731024866

Time since start: 2587.528

140: time = 1.647000 loss = 7.900645 testLoss 7.794034

Res.gain: 1.3119926205194146

Jackknife: only bio_6

Making features

makeFeatures: max memory 259522560, total allocated 139407360, free 94134304, used 45273056, increment -21129144

Amomum_tsaoko_6 bio_6:

Regularization values: linear/quadratic/product: 0.385, categorical: 0.250, threshold: 1.770, hinge: 0.500

23 samples

Density: max memory 259522560, total allocated 139407360, free 91641488, used 47765872, increment 2492816

linearPredictor: max memory 259522560, total allocated 139407360, free 91641488, used 47765872, increment 0

FeaturedSpace: max memory 259522560, total allocated 139407360, free 91641488, used 47765872, increment 0

Sequential: max memory 259522560, total allocated 139407360, free 91641488, used 47765872, increment 0

Initial loss: 9.212637731024866

Initial test loss: 9.212637731024866

Time since start: 2589.15

180: time = 1.587000 loss = 7.999799 testLoss 8.196438

Res.gain: 1.2128389479478185

Jackknife: only elev

Making features

makeFeatures: max memory 259522560, total allocated 139407360, free 60574800, used 78832560, increment 31066688

Amomum_tsaoko_6 elev:

Regularization values: linear/quadratic/product: 0.385, categorical: 0.250, threshold: 1.770, hinge: 0.500

23 samples

Density: max memory 259522560, total allocated 139407360, free 58903496, used 80503864, increment 1671304

linearPredictor: max memory 259522560, total allocated 139407360, free 58903496, used 80503864, increment 0

FeaturedSpace: max memory 259522560, total allocated 139407360, free 58903496, used 80503864, increment 0

Sequential: max memory 259522560, total allocated 139407360, free 58903496, used 80503864, increment 0

Initial loss: 9.212637731024866

Initial test loss: 9.212637731024866

120: time = 0.872000 loss = 9.047861 testLoss 9.305020

Res.gain: 0.16477691544985973

Jackknife: only s_caco3

Making features

makeFeatures: max memory 259522560, total allocated 139407360, free 61441672, used 77965688, increment -2538176

Amomum_tsaoko_6 s_caco3:

Regularization values: linear/quadratic/product: 0.385, categorical: 0.250, threshold: 1.770, hinge: 0.500

23 samples

Density: max memory 259522560, total allocated 139407360, free 60658968, used 78748392, increment 782704

linearPredictor: max memory 259522560, total allocated 139407360, free 60578768, used 78828592, increment 80200

FeaturedSpace: max memory 259522560, total allocated 139407360, free 60578768, used 78828592, increment 0

Sequential: max memory 259522560, total allocated 139407360, free 60578768, used 78828592, increment 0

Initial loss: 9.212637731024866

Initial test loss: 9.212637731024866

Time since start: 2590.672

80: time = 0.586000 loss = 8.474385 testLoss 9.031302

Res.gain: 0.7382529746755342

Jackknife: only s_ph_h2o

Making features

makeFeatures: max memory 259522560, total allocated 139407360, free 58537344, used 80870016, increment 2041424

Amomum_tsaoko_6 s_ph_h2o:

Regularization values: linear/quadratic/product: 0.385, categorical: 0.250, threshold: 1.770, hinge: 0.500

23 samples

Density: max memory 259522560, total allocated 139407360, free 57106880, used 82300480, increment 1430464

linearPredictor: max memory 259522560, total allocated 139407360, free 57106880, used 82300480, increment 0

FeaturedSpace: max memory 259522560, total allocated 139407360, free 57106880, used 82300480, increment 0

Sequential: max memory 259522560, total allocated 139407360, free 57106880, used 82300480, increment 0

Initial loss: 9.212637731024866

Initial test loss: 9.212637731024866

Time since start: 2591.757

260: time = 1.058000 loss = 8.517128 testLoss 8.805352

Res.gain: 0.6955098321357287

Jackknife: only slope

Making features

makeFeatures: max memory 259522560, total allocated 139407360, free 90880536, used 48526824, increment -33773656

Amomum_tsaoko_6 slope:

Regularization values: linear/quadratic/product: 0.385, categorical: 0.250, threshold: 1.770, hinge: 0.500

23 samples

Density: max memory 259522560, total allocated 139407360, free 88533816, used 50873544, increment 2346720

linearPredictor: max memory 259522560, total allocated 139407360, free 88533816, used 50873544, increment 0

FeaturedSpace: max memory 259522560, total allocated 139407360, free 88533816, used 50873544, increment 0

Sequential: max memory 259522560, total allocated 139407360, free 88533816, used 50873544, increment 0

Initial loss: 9.212637731024866

Initial test loss: 9.212637731024866

Time since start: 2593.807

200: time = 2.022000 loss = 9.074977 testLoss 9.024833

Res.gain: 0.13766102989559847

Jackknife: only t_clay

Making features

makeFeatures: max memory 259522560, total allocated 139407360, free 74509480, used 64897880, increment 14024336

Amomum_tsaoko_6 t_clay:

Regularization values: linear/quadratic/product: 0.385, categorical: 0.250, threshold: 1.770, hinge: 0.500

23 samples

Density: max memory 259522560, total allocated 139407360, free 73566840, used 65840520, increment 942640

linearPredictor: max memory 259522560, total allocated 139407360, free 73486640, used 65920720, increment 80200

FeaturedSpace: max memory 259522560, total allocated 139407360, free 73486640, used 65920720, increment 0

Sequential: max memory 259522560, total allocated 139407360, free 73486640, used 65920720, increment 0

Initial loss: 9.212637731024866

Initial test loss: 9.212637731024866

100: time = 0.504000 loss = 8.432537 testLoss 9.160832

Res.gain: 0.78010050037593

Jackknife: only t_oc

Making features

makeFeatures: max memory 259522560, total allocated 139407360, free 71183280, used 68224080, increment 2303360

Amomum_tsaoko_6 t_oc:

Regularization values: linear/quadratic/product: 0.385, categorical: 0.250, threshold: 1.770, hinge: 0.500

23 samples

Density: max memory 259522560, total allocated 139407360, free 70200440, used 69206920, increment 982840

linearPredictor: max memory 259522560, total allocated 139407360, free 70120240, used 69287120, increment 80200

FeaturedSpace: max memory 259522560, total allocated 139407360, free 70120240, used 69287120, increment 0

Sequential: max memory 259522560, total allocated 139407360, free 70120240, used 69287120, increment 0

Initial loss: 9.212637731024866

Initial test loss: 9.212637731024866

Time since start: 2594.812

100: time = 0.448000 loss = 8.842995 testLoss 8.838855

Res.gain: 0.3696423036904122

Jackknife: only t_sand

Making features

makeFeatures: max memory 259522560, total allocated 139407360, free 66941144, used 72466216, increment 3179096

Amomum_tsaoko_6 t_sand:

Regularization values: linear/quadratic/product: 0.385, categorical: 0.250, threshold: 1.770, hinge: 0.500

23 samples

Density: max memory 259522560, total allocated 139407360, free 66038296, used 73369064, increment 902848

linearPredictor: max memory 259522560, total allocated 139407360, free 65269064, used 74138296, increment 769232

FeaturedSpace: max memory 259522560, total allocated 139407360, free 65269064, used 74138296, increment 0

Sequential: max memory 259522560, total allocated 139407360, free 65269064, used 74138296, increment 0

Initial loss: 9.212637731024866

Initial test loss: 9.212637731024866

160: time = 0.653000 loss = 8.846065 testLoss 9.423121

Res.gain: 0.36657273854514116

Time since start: 2595.818

getSamples: max memory 259522560, total allocated 139407360, free 88033032, used 51374328, increment -22763968

Making features

makeFeatures: max memory 259522560, total allocated 139407360, free 79130744, used 60276616, increment 8902288

Amomum_tsaoko_7:

Regularization values: linear/quadratic/product: 0.385, categorical: 0.250, threshold: 1.770, hinge: 0.500

23 samples

Density: max memory 259522560, total allocated 139407360, free 57988264, used 81419096, increment 21142480

linearPredictor: max memory 259522560, total allocated 139407360, free 57988264, used 81419096, increment 0

Deactivating (awc_class=2.0)

Deactivating (awc_class=3.0)

Deactivating (awc_class=4.0)

Deactivating (awc_class=6.0)

FeaturedSpace: max memory 259522560, total allocated 139407360, free 57988264, used 81419096, increment 0

Sequential: max memory 259522560, total allocated 139407360, free 57988264, used 81419096, increment 0

Initial loss: 9.21253795551967

Initial test loss: 9.21253795551967

Time since start: 2616.964

500: time = 20.833000 loss = 6.959355 testLoss 7.705234

Resulting gain: 2.253183018388122

Projecting...

Writing file E:\Amomum_tsaoko\results2\Amomum_tsaoko_7.asc

Time since start: 2621.562

Writing E:\Amomum_tsaoko\results2\plots\Amomum_tsaoko_7.png

Time since start: 2622.976

Projecting...

Writing file E:\Amomum_tsaoko\results2\Amomum_tsaoko_7_LGM.asc

Writing file E:\Amomum_tsaoko\results2\Amomum_tsaoko_7_LGM_clamping.asc

Time since start: 2628.541

Writing E:\Amomum_tsaoko\results2\plots\Amomum_tsaoko_7_LGM.png

Time since start: 2630.029

Writing E:\Amomum_tsaoko\results2\plots\Amomum_tsaoko_7_LGM_clamping.png

Time since start: 2631.651

Writing file E:\Amomum_tsaoko\results2\Amomum_tsaoko_7_LGM_novel.asc

Writing file E:\Amomum_tsaoko\results2\Amomum_tsaoko_7_LGM_novel_limiting.asc

Time since start: 2636.397

Projecting...

Writing file E:\Amomum_tsaoko\results2\Amomum_tsaoko_7_MH.asc

Writing file E:\Amomum_tsaoko\results2\Amomum_tsaoko_7_MH_clamping.asc

Time since start: 2641.776

Writing E:\Amomum_tsaoko\results2\plots\Amomum_tsaoko_7_MH.png

Time since start: 2643.303

Writing E:\Amomum_tsaoko\results2\plots\Amomum_tsaoko_7_MH_clamping.png

Time since start: 2644.902

Writing file E:\Amomum_tsaoko\results2\Amomum_tsaoko_7_MH_novel.asc

Writing file E:\Amomum_tsaoko\results2\Amomum_tsaoko_7_MH_novel_limiting.asc

Time since start: 2649.567

Projecting...

Writing file E:\Amomum_tsaoko\results2\Amomum_tsaoko_7_SSP126-2050S.asc

Writing file E:\Amomum_tsaoko\results2\Amomum_tsaoko_7_SSP126-2050S_clamping.asc

Time since start: 2654.034

Writing E:\Amomum_tsaoko\results2\plots\Amomum_tsaoko_7_SSP126-2050S.png

Time since start: 2655.706

Writing E:\Amomum_tsaoko\results2\plots\Amomum_tsaoko_7_SSP126-2050S_clamping.png

Time since start: 2657.183

Writing file E:\Amomum_tsaoko\results2\Amomum_tsaoko_7_SSP126-2050S_novel.asc

Writing file E:\Amomum_tsaoko\results2\Amomum_tsaoko_7_SSP126-2050S_novel_limiting.asc

Time since start: 2661.792

Projecting...

Writing file E:\Amomum_tsaoko\results2\Amomum_tsaoko_7_SSP126-2090S.asc

Writing file E:\Amomum_tsaoko\results2\Amomum_tsaoko_7_SSP126-2090S_clamping.asc

Time since start: 2666.327

Writing E:\Amomum_tsaoko\results2\plots\Amomum_tsaoko_7_SSP126-2090S.png

Time since start: 2667.751

Writing E:\Amomum_tsaoko\results2\plots\Amomum_tsaoko_7_SSP126-2090S_clamping.png

Time since start: 2669.11

Writing file E:\Amomum_tsaoko\results2\Amomum_tsaoko_7_SSP126-2090S_novel.asc

Writing file E:\Amomum_tsaoko\results2\Amomum_tsaoko_7_SSP126-2090S_novel_limiting.asc

Time since start: 2674.03

Projecting...

Writing file E:\Amomum_tsaoko\results2\Amomum_tsaoko_7_SSP585-2050S.asc

Writing file E:\Amomum_tsaoko\results2\Amomum_tsaoko_7_SSP585-2050S_clamping.asc

Time since start: 2678.411

Writing E:\Amomum_tsaoko\results2\plots\Amomum_tsaoko_7_SSP585-2050S.png

Time since start: 2679.921

Writing E:\Amomum_tsaoko\results2\plots\Amomum_tsaoko_7_SSP585-2050S_clamping.png

Time since start: 2681.387

Writing file E:\Amomum_tsaoko\results2\Amomum_tsaoko_7_SSP585-2050S_novel.asc

Writing file E:\Amomum_tsaoko\results2\Amomum_tsaoko_7_SSP585-2050S_novel_limiting.asc

Time since start: 2686.184

Projecting...

Writing file E:\Amomum_tsaoko\results2\Amomum_tsaoko_7_SSP585-2090S.asc

Writing file E:\Amomum_tsaoko\results2\Amomum_tsaoko_7_SSP585-2090S_clamping.asc

Time since start: 2690.514

Writing E:\Amomum_tsaoko\results2\plots\Amomum_tsaoko_7_SSP585-2090S.png

Time since start: 2691.877

Writing E:\Amomum_tsaoko\results2\plots\Amomum_tsaoko_7_SSP585-2090S_clamping.png

Time since start: 2693.381

Writing file E:\Amomum_tsaoko\results2\Amomum_tsaoko_7_SSP585-2090S_novel.asc

Writing file E:\Amomum_tsaoko\results2\Amomum_tsaoko_7_SSP585-2090S_novel_limiting.asc

Time since start: 2698.236

Amomum_tsaoko_7 response curves

Response curve: only aspect

Making features

makeFeatures: max memory 259522560, total allocated 139407360, free 50721168, used 88686192, increment 7267096

Amomum_tsaoko_7 aspect:

Regularization values: linear/quadratic/product: 0.385, categorical: 0.250, threshold: 1.770, hinge: 0.500

23 samples

Density: max memory 259522560, total allocated 139407360, free 48089680, used 91317680, increment 2631488

linearPredictor: max memory 259522560, total allocated 139407360, free 48089680, used 91317680, increment 0

FeaturedSpace: max memory 259522560, total allocated 139407360, free 48089680, used 91317680, increment 0

Sequential: max memory 259522560, total allocated 139407360, free 48089680, used 91317680, increment 0

Initial loss: 9.21253795551967

Time since start: 2702.383

280: time = 3.168000 loss = 9.083583

Resulting gain: 0.1289552708642816

Amomum_tsaoko_7 response curves

Response curve: only awc_class

Making features

makeFeatures: max memory 259522560, total allocated 139407360, free 62679872, used 76727488, increment -14590192

Amomum_tsaoko_7 awc_class:

Regularization values: linear/quadratic/product: 0.385, categorical: 0.250, threshold: 1.770, hinge: 0.500

23 samples

Density: max memory 259522560, total allocated 139407360, free 62668944, used 76738416, increment 10928

linearPredictor: max memory 259522560, total allocated 139407360, free 62668944, used 76738416, increment 0

Deactivating (awc_class=2.0)

Deactivating (awc_class=3.0)

Deactivating (awc_class=4.0)

Deactivating (awc_class=6.0)

FeaturedSpace: max memory 259522560, total allocated 139407360, free 62668944, used 76738416, increment 0

Sequential: max memory 259522560, total allocated 139407360, free 62668944, used 76738416, increment 0

Initial loss: 9.21253795551967

100: time = 0.145000 loss = 9.196540

Resulting gain: 0.015997823449556137

Amomum_tsaoko_7 response curves

Response curve: only bio_12

Making features

makeFeatures: max memory 259522560, total allocated 139407360, free 57963512, used 81443848, increment 4705432

Amomum_tsaoko_7 bio_12:

Regularization values: linear/quadratic/product: 0.385, categorical: 0.250, threshold: 1.770, hinge: 0.500

23 samples

Density: max memory 259522560, total allocated 139407360, free 56408400, used 82998960, increment 1555112

linearPredictor: max memory 259522560, total allocated 139407360, free 56408400, used 82998960, increment 0

FeaturedSpace: max memory 259522560, total allocated 139407360, free 56408400, used 82998960, increment 0

Sequential: max memory 259522560, total allocated 139407360, free 56408400, used 82998960, increment 0

Initial loss: 9.21253795551967

Time since start: 2703.821

220: time = 1.094000 loss = 8.012855

Resulting gain: 1.1996825551398818

Amomum_tsaoko_7 response curves

Response curve: only bio_15

Making features

makeFeatures: max memory 259522560, total allocated 139407360, free 43773080, used 95634280, increment 12635320

Amomum_tsaoko_7 bio_15:

Regularization values: linear/quadratic/product: 0.385, categorical: 0.250, threshold: 1.770, hinge: 0.500

23 samples

Density: max memory 259522560, total allocated 139407360, free 40578304, used 98829056, increment 3194776

linearPredictor: max memory 259522560, total allocated 139407360, free 40578304, used 98829056, increment 0

FeaturedSpace: max memory 259522560, total allocated 139407360, free 40578304, used 98829056, increment 0

Sequential: max memory 259522560, total allocated 139407360, free 40578304, used 98829056, increment 0

Initial loss: 9.21253795551967

Time since start: 2708.7

500: time = 4.779000 loss = 8.771239

Resulting gain: 0.4412991149911516

Amomum_tsaoko_7 response curves

Response curve: only bio_17

Making features

makeFeatures: max memory 259522560, total allocated 139407360, free 42513680, used 96893680, increment -1935376

Amomum_tsaoko_7 bio_17:

Regularization values: linear/quadratic/product: 0.385, categorical: 0.250, threshold: 1.770, hinge: 0.500

23 samples

Density: max memory 259522560, total allocated 139407360, free 41613232, used 97794128, increment 900448

linearPredictor: max memory 259522560, total allocated 139407360, free 41533040, used 97874320, increment 80192

FeaturedSpace: max memory 259522560, total allocated 139407360, free 41533040, used 97874320, increment 0

Sequential: max memory 259522560, total allocated 139407360, free 41533040, used 97874320, increment 0

Initial loss: 9.21253795551967

Time since start: 2709.876

300: time = 1.064000 loss = 8.012343

Resulting gain: 1.2001948614080487

Amomum_tsaoko_7 response curves

Response curve: only bio_4

Making features

makeFeatures: max memory 259522560, total allocated 139407360, free 58347296, used 81060064, increment -16814256

Amomum_tsaoko_7 bio_4:

Regularization values: linear/quadratic/product: 0.385, categorical: 0.250, threshold: 1.770, hinge: 0.500

23 samples

Density: max memory 259522560, total allocated 139407360, free 55554936, used 83852424, increment 2792360

linearPredictor: max memory 259522560, total allocated 139407360, free 55474744, used 83932616, increment 80192

FeaturedSpace: max memory 259522560, total allocated 139407360, free 55474744, used 83932616, increment 0

Sequential: max memory 259522560, total allocated 139407360, free 55474744, used 83932616, increment 0

Initial loss: 9.21253795551967

Time since start: 2711.312

120: time = 1.315000 loss = 7.733019

Resulting gain: 1.4795189239488407

Amomum_tsaoko_7 response curves

Response curve: only bio_6

Making features

makeFeatures: max memory 259522560, total allocated 139407360, free 37336784, used 102070576, increment 18137960

Amomum_tsaoko_7 bio_6:

Regularization values: linear/quadratic/product: 0.385, categorical: 0.250, threshold: 1.770, hinge: 0.500

23 samples

Density: max memory 259522560, total allocated 139407360, free 35522480, used 103884880, increment 1814304

linearPredictor: max memory 259522560, total allocated 139407360, free 35442288, used 103965072, increment 80192

FeaturedSpace: max memory 259522560, total allocated 139407360, free 35442288, used 103965072, increment 0

Sequential: max memory 259522560, total allocated 139407360, free 35442288, used 103965072, increment 0

Initial loss: 9.21253795551967

Time since start: 2712.972

180: time = 1.547000 loss = 7.933779

Resulting gain: 1.2787586876665218

Amomum_tsaoko_7 response curves

Response curve: only elev

Making features

makeFeatures: max memory 259522560, total allocated 139407360, free 38315864, used 101091496, increment -2873576

Amomum_tsaoko_7 elev:

Regularization values: linear/quadratic/product: 0.385, categorical: 0.250, threshold: 1.770, hinge: 0.500

23 samples

Density: max memory 259522560, total allocated 139407360, free 36765072, used 102642288, increment 1550792

linearPredictor: max memory 259522560, total allocated 139407360, free 36765072, used 102642288, increment 0

FeaturedSpace: max memory 259522560, total allocated 139407360, free 36765072, used 102642288, increment 0

Sequential: max memory 259522560, total allocated 139407360, free 36765072, used 102642288, increment 0

Initial loss: 9.21253795551967

100: time = 0.830000 loss = 9.068528

Resulting gain: 0.14400948920119028

Amomum_tsaoko_7 response curves

Time since start: 2714.03

Response curve: only s_caco3

Making features

makeFeatures: max memory 259522560, total allocated 139407360, free 47721152, used 91686208, increment -10956080

Amomum_tsaoko_7 s_caco3:

Regularization values: linear/quadratic/product: 0.385, categorical: 0.250, threshold: 1.770, hinge: 0.500

23 samples

Density: max memory 259522560, total allocated 139407360, free 46942280, used 92465080, increment 778872

linearPredictor: max memory 259522560, total allocated 139407360, free 46942280, used 92465080, increment 0

FeaturedSpace: max memory 259522560, total allocated 139407360, free 46942280, used 92465080, increment 0

Sequential: max memory 259522560, total allocated 139407360, free 46942280, used 92465080, increment 0

Initial loss: 9.21253795551967

120: time = 0.848000 loss = 8.713660

Resulting gain: 0.49887812472639403

Amomum_tsaoko_7 response curves

Response curve: only s_ph_h2o

Making features

makeFeatures: max memory 259522560, total allocated 139407360, free 40623880, used 98783480, increment 6318400

Amomum_tsaoko_7 s_ph_h2o:

Regularization values: linear/quadratic/product: 0.385, categorical: 0.250, threshold: 1.770, hinge: 0.500

Time since start: 2715.042

23 samples

Density: max memory 259522560, total allocated 139407360, free 39794320, used 99613040, increment 829560

linearPredictor: max memory 259522560, total allocated 139407360, free 39794320, used 99613040, increment 0

FeaturedSpace: max memory 259522560, total allocated 139407360, free 39794320, used 99613040, increment 0

Sequential: max memory 259522560, total allocated 139407360, free 39794320, used 99613040, increment 0

Initial loss: 9.21253795551967

Time since start: 2716.266

200: time = 1.224000 loss = 8.686695

Resulting gain: 0.5258425306944083

Amomum_tsaoko_7 response curves

Response curve: only slope

Making features

makeFeatures: max memory 259522560, total allocated 139407360, free 69649400, used 69757960, increment -29855080

Amomum_tsaoko_7 slope:

Regularization values: linear/quadratic/product: 0.385, categorical: 0.250, threshold: 1.770, hinge: 0.500

23 samples

Density: max memory 259522560, total allocated 139407360, free 67892600, used 71514760, increment 1756800

linearPredictor: max memory 259522560, total allocated 139407360, free 67892600, used 71514760, increment 0

FeaturedSpace: max memory 259522560, total allocated 139407360, free 67892600, used 71514760, increment 0

Sequential: max memory 259522560, total allocated 139407360, free 67132184, used 72275176, increment 760416

Initial loss: 9.21253795551967

Time since start: 2719.517

200: time = 3.087000 loss = 9.091610

Resulting gain: 0.12092795361604125

Amomum_tsaoko_7 response curves

Response curve: only t_clay

Making features

makeFeatures: max memory 259522560, total allocated 139407360, free 51333056, used 88074304, increment 15799128

Amomum_tsaoko_7 t_clay:

Regularization values: linear/quadratic/product: 0.385, categorical: 0.250, threshold: 1.770, hinge: 0.500

23 samples

Density: max memory 259522560, total allocated 139407360, free 50553288, used 88854072, increment 779768

linearPredictor: max memory 259522560, total allocated 139407360, free 50553288, used 88854072, increment 0

FeaturedSpace: max memory 259522560, total allocated 139407360, free 50553288, used 88854072, increment 0

Sequential: max memory 259522560, total allocated 139407360, free 50553288, used 88854072, increment 0

Initial loss: 9.21253795551967

100: time = 0.639000 loss = 8.700937

Resulting gain: 0.5116014139287373

Amomum_tsaoko_7 response curves

Response curve: only t_oc

Making features

makeFeatures: max memory 259522560, total allocated 139407360, free 44311984, used 95095376, increment 6241304

Amomum_tsaoko_7 t_oc:

Regularization values: linear/quadratic/product: 0.385, categorical: 0.250, threshold: 1.770, hinge: 0.500

23 samples

Density: max memory 259522560, total allocated 139407360, free 43532168, used 95875192, increment 779816

linearPredictor: max memory 259522560, total allocated 139407360, free 43532168, used 95875192, increment 0

FeaturedSpace: max memory 259522560, total allocated 139407360, free 43532168, used 95875192, increment 0

Sequential: max memory 259522560, total allocated 139407360, free 43532168, used 95875192, increment 0

Initial loss: 9.21253795551967

Time since start: 2720.905

80: time = 0.503000 loss = 8.815381

Resulting gain: 0.397157452718627

Amomum_tsaoko_7 response curves

Response curve: only t_sand

Making features

makeFeatures: max memory 259522560, total allocated 139407360, free 37294336, used 102113024, increment 6237832

Amomum_tsaoko_7 t_sand:

Regularization values: linear/quadratic/product: 0.385, categorical: 0.250, threshold: 1.770, hinge: 0.500

23 samples

Density: max memory 259522560, total allocated 139407360, free 36353768, used 103053592, increment 940568

linearPredictor: max memory 259522560, total allocated 139407360, free 36273576, used 103133784, increment 80192

FeaturedSpace: max memory 259522560, total allocated 139407360, free 36273576, used 103133784, increment 0

Sequential: max memory 259522560, total allocated 139407360, free 36273576, used 103133784, increment 0

Initial loss: 9.21253795551967

Time since start: 2722.026

180: time = 1.009000 loss = 8.979608

Resulting gain: 0.23292996628114437

Amomum_tsaoko_7 response curves

Time since start: 2723.881

Jackknife: leave aspect out

Making features

makeFeatures: max memory 259522560, total allocated 139407360, free 57110696, used 82296664, increment -20837120

Amomum_tsaoko_7 aspect:

Regularization values: linear/quadratic/product: 0.385, categorical: 0.250, threshold: 1.770, hinge: 0.500

23 samples

Density: max memory 259522560, total allocated 139407360, free 38339272, used 101068088, increment 18771424

linearPredictor: max memory 259522560, total allocated 139407360, free 38339272, used 101068088, increment 0

Deactivating (awc_class=2.0)

Deactivating (awc_class=3.0)

Deactivating (awc_class=4.0)

Deactivating (awc_class=6.0)

FeaturedSpace: max memory 259522560, total allocated 139407360, free 38339272, used 101068088, increment 0

Sequential: max memory 259522560, total allocated 139407360, free 38339272, used 101068088, increment 0

Initial loss: 9.21253795551967

Initial test loss: 9.21253795551967

Time since start: 2741.805

500: time = 17.484000 loss = 7.034492 testLoss 7.821398

Jackknife: leave awc_class out

Making features

makeFeatures: max memory 259522560, total allocated 139407360, free 27278240, used 112129120, increment 11061032

Amomum_tsaoko_7 awc_class:

Regularization values: linear/quadratic/product: 0.385, categorical: 0.250, threshold: 1.770, hinge: 0.500

23 samples

Density: max memory 259522560, total allocated 139407360, free 32101048, used 107306312, increment -4822808

linearPredictor: max memory 259522560, total allocated 139407360, free 32101048, used 107306312, increment 0

FeaturedSpace: max memory 259522560, total allocated 139407360, free 32101048, used 107306312, increment 0

Sequential: max memory 259522560, total allocated 139407360, free 32101048, used 107306312, increment 0

Initial loss: 9.21253795551967

Initial test loss: 9.21253795551967

Time since start: 2762.447

500: time = 20.322000 loss = 6.959738 testLoss 7.685880

Jackknife: leave bio_12 out

Making features

makeFeatures: max memory 259522560, total allocated 139407360, free 87200264, used 52207096, increment -55099216

Amomum_tsaoko_7 bio_12:

Regularization values: linear/quadratic/product: 0.385, categorical: 0.250, threshold: 1.770, hinge: 0.500

23 samples

Density: max memory 259522560, total allocated 139407360, free 67135696, used 72271664, increment 20064568

linearPredictor: max memory 259522560, total allocated 139407360, free 67135696, used 72271664, increment 0

Deactivating (awc_class=2.0)

Deactivating (awc_class=3.0)

Deactivating (awc_class=4.0)

Deactivating (awc_class=6.0)

FeaturedSpace: max memory 259522560, total allocated 139407360, free 67135696, used 72271664, increment 0

Sequential: max memory 259522560, total allocated 139407360, free 67135696, used 72271664, increment 0

Initial loss: 9.21253795551967

Initial test loss: 9.21253795551967

Time since start: 2782.206

500: time = 19.351000 loss = 7.000745 testLoss 7.820508

Jackknife: leave bio_15 out

Making features

makeFeatures: max memory 259522560, total allocated 139407360, free 52619512, used 86787848, increment 14516184

Amomum_tsaoko_7 bio_15:

Regularization values: linear/quadratic/product: 0.385, categorical: 0.250, threshold: 1.770, hinge: 0.500

23 samples

Density: max memory 259522560, total allocated 139407360, free 59484088, used 79923272, increment -6864576

linearPredictor: max memory 259522560, total allocated 139407360, free 59484088, used 79923272, increment 0

Deactivating (awc_class=2.0)

Deactivating (awc_class=3.0)

Deactivating (awc_class=4.0)

Deactivating (awc_class=6.0)

FeaturedSpace: max memory 259522560, total allocated 139407360, free 59484088, used 79923272, increment 0

Sequential: max memory 259522560, total allocated 139407360, free 59484088, used 79923272, increment 0

Initial loss: 9.21253795551967

Initial test loss: 9.21253795551967

Time since start: 2799.013

500: time = 16.499000 loss = 6.960672 testLoss 7.759042

Jackknife: leave bio_17 out

Making features

makeFeatures: max memory 259522560, total allocated 139407360, free 57478928, used 81928432, increment 2005160

Amomum_tsaoko_7 bio_17:

Regularization values: linear/quadratic/product: 0.385, categorical: 0.250, threshold: 1.770, hinge: 0.500

23 samples

Density: max memory 259522560, total allocated 139407360, free 36757448, used 102649912, increment 20721480

linearPredictor: max memory 259522560, total allocated 139407360, free 36757448, used 102649912, increment 0

Deactivating (awc_class=2.0)

Deactivating (awc_class=3.0)

Deactivating (awc_class=4.0)

Deactivating (awc_class=6.0)

FeaturedSpace: max memory 259522560, total allocated 139407360, free 36757448, used 102649912, increment 0

Sequential: max memory 259522560, total allocated 139407360, free 36757448, used 102649912, increment 0

Initial loss: 9.21253795551967

Initial test loss: 9.21253795551967

Time since start: 2808.757

500: time = 9.539000 loss = 6.982563 testLoss 7.348682

Jackknife: leave bio_4 out

Making features

makeFeatures: max memory 259522560, total allocated 139407360, free 13129728, used 126277632, increment 23627720

Amomum_tsaoko_7 bio_4:

Regularization values: linear/quadratic/product: 0.385, categorical: 0.250, threshold: 1.770, hinge: 0.500

23 samples

Density: max memory 259522560, total allocated 139407360, free 25796216, used 113611144, increment -12666488

linearPredictor: max memory 259522560, total allocated 139407360, free 25716024, used 113691336, increment 80192

Deactivating (awc_class=2.0)

Deactivating (awc_class=3.0)

Deactivating (awc_class=4.0)

Deactivating (awc_class=6.0)

FeaturedSpace: max memory 259522560, total allocated 139407360, free 25716024, used 113691336, increment 0

Sequential: max memory 259522560, total allocated 139407360, free 25716024, used 113691336, increment 0

Initial loss: 9.21253795551967

Initial test loss: 9.21253795551967

Time since start: 2821.121

500: time = 12.251000 loss = 6.995975 testLoss 7.729548

Jackknife: leave bio_6 out

Making features

makeFeatures: max memory 259522560, total allocated 139407360, free 89274224, used 50133136, increment -63558200

Amomum_tsaoko_7 bio_6:

Regularization values: linear/quadratic/product: 0.385, categorical: 0.250, threshold: 1.770, hinge: 0.500

23 samples

Density: max memory 259522560, total allocated 139407360, free 69956304, used 69451056, increment 19317920

linearPredictor: max memory 259522560, total allocated 139407360, free 69956304, used 69451056, increment 0

Deactivating (awc_class=2.0)

Deactivating (awc_class=3.0)

Deactivating (awc_class=4.0)

Deactivating (awc_class=6.0)

FeaturedSpace: max memory 259522560, total allocated 139407360, free 69956304, used 69451056, increment 0

Sequential: max memory 259522560, total allocated 139407360, free 69956304, used 69451056, increment 0

Initial loss: 9.21253795551967

Initial test loss: 9.21253795551967

Time since start: 2841.48

500: time = 20.043000 loss = 6.968227 testLoss 7.685786

Jackknife: leave elev out

Making features

makeFeatures: max memory 259522560, total allocated 139407360, free 61585272, used 77822088, increment 8371032

Amomum_tsaoko_7 elev:

Regularization values: linear/quadratic/product: 0.385, categorical: 0.250, threshold: 1.770, hinge: 0.500

23 samples

Density: max memory 259522560, total allocated 139407360, free 65636896, used 73770464, increment -4051624

linearPredictor: max memory 259522560, total allocated 139407360, free 65636896, used 73770464, increment 0

Deactivating (awc_class=2.0)

Deactivating (awc_class=3.0)

Deactivating (awc_class=4.0)

Deactivating (awc_class=6.0)

FeaturedSpace: max memory 259522560, total allocated 139407360, free 65636896, used 73770464, increment 0

Sequential: max memory 259522560, total allocated 139407360, free 65636896, used 73770464, increment 0

Initial loss: 9.21253795551967

Initial test loss: 9.21253795551967

Time since start: 2860.245

500: time = 18.441000 loss = 7.022437 testLoss 7.452446

Jackknife: leave s_caco3 out

Making features

makeFeatures: max memory 259522560, total allocated 139407360, free 32418712, used 106988648, increment 33218184

Amomum_tsaoko_7 s_caco3:

Regularization values: linear/quadratic/product: 0.385, categorical: 0.250, threshold: 1.770, hinge: 0.500

23 samples

Density: max memory 259522560, total allocated 139407360, free 41537568, used 97869792, increment -9118856

linearPredictor: max memory 259522560, total allocated 139407360, free 41457376, used 97949984, increment 80192

Deactivating (awc_class=2.0)

Deactivating (awc_class=3.0)

Deactivating (awc_class=4.0)

Deactivating (awc_class=6.0)

FeaturedSpace: max memory 259522560, total allocated 139407360, free 41457376, used 97949984, increment 0

Sequential: max memory 259522560, total allocated 139407360, free 41457376, used 97949984, increment 0

Initial loss: 9.21253795551967

Initial test loss: 9.21253795551967

Time since start: 2880.151

500: time = 19.586000 loss = 6.981684 testLoss 7.794148

Jackknife: leave s_ph_h2o out

Making features

makeFeatures: max memory 259522560, total allocated 139407360, free 45437624, used 93969736, increment -3980248

Amomum_tsaoko_7 s_ph_h2o:

Regularization values: linear/quadratic/product: 0.385, categorical: 0.250, threshold: 1.770, hinge: 0.500

23 samples

Density: max memory 259522560, total allocated 139407360, free 24839024, used 114568336, increment 20598600

linearPredictor: max memory 259522560, total allocated 139407360, free 24839024, used 114568336, increment 0

Deactivating (awc_class=2.0)

Deactivating (awc_class=3.0)

Deactivating (awc_class=4.0)

Deactivating (awc_class=6.0)

FeaturedSpace: max memory 259522560, total allocated 139407360, free 24839024, used 114568336, increment 0

Sequential: max memory 259522560, total allocated 139407360, free 24839024, used 114568336, increment 0

Initial loss: 9.21253795551967

Initial test loss: 9.21253795551967

Time since start: 2899.583

500: time = 19.104000 loss = 6.960418 testLoss 7.741178

Jackknife: leave slope out

Making features

makeFeatures: max memory 259522560, total allocated 139407360, free 84882056, used 54525304, increment -60043032

Amomum_tsaoko_7 slope:

Regularization values: linear/quadratic/product: 0.385, categorical: 0.250, threshold: 1.770, hinge: 0.500

23 samples

Density: max memory 259522560, total allocated 139407360, free 65544672, used 73862688, increment 19337384

linearPredictor: max memory 259522560, total allocated 139407360, free 65544672, used 73862688, increment 0

Deactivating (awc_class=2.0)

Deactivating (awc_class=3.0)

Deactivating (awc_class=4.0)

Deactivating (awc_class=6.0)

FeaturedSpace: max memory 259522560, total allocated 139407360, free 65544672, used 73862688, increment 0

Sequential: max memory 259522560, total allocated 139407360, free 65544672, used 73862688, increment 0

Initial loss: 9.21253795551967

Initial test loss: 9.21253795551967

Time since start: 2917.321

500: time = 17.434000 loss = 7.132950 testLoss 8.165385

Jackknife: leave t_clay out

Making features

makeFeatures: max memory 259522560, total allocated 139407360, free 49265192, used 90142168, increment 16279480

Amomum_tsaoko_7 t_clay:

Regularization values: linear/quadratic/product: 0.385, categorical: 0.250, threshold: 1.770, hinge: 0.500

23 samples

Density: max memory 259522560, total allocated 139407360, free 60130536, used 79276824, increment -10865344

linearPredictor: max memory 259522560, total allocated 139407360, free 60050344, used 79357016, increment 80192

Deactivating (awc_class=2.0)

Deactivating (awc_class=3.0)

Deactivating (awc_class=4.0)

Deactivating (awc_class=6.0)

FeaturedSpace: max memory 259522560, total allocated 139407360, free 60050344, used 79357016, increment 0

Sequential: max memory 259522560, total allocated 139407360, free 60050344, used 79357016, increment 0

Initial loss: 9.21253795551967

Initial test loss: 9.21253795551967

Time since start: 2938.587

500: time = 20.942000 loss = 6.959409 testLoss 7.708333

Jackknife: leave t_oc out

Making features

makeFeatures: max memory 259522560, total allocated 139407360, free 66733144, used 72674216, increment -6682800

Amomum_tsaoko_7 t_oc:

Regularization values: linear/quadratic/product: 0.385, categorical: 0.250, threshold: 1.770, hinge: 0.500

23 samples

Density: max memory 259522560, total allocated 139407360, free 45839680, used 93567680, increment 20893464

linearPredictor: max memory 259522560, total allocated 139407360, free 45839680, used 93567680, increment 0

Deactivating (awc_class=2.0)

Deactivating (awc_class=3.0)

Deactivating (awc_class=4.0)

Deactivating (awc_class=6.0)

FeaturedSpace: max memory 259522560, total allocated 139407360, free 45839680, used 93567680, increment 0

Sequential: max memory 259522560, total allocated 139407360, free 45839680, used 93567680, increment 0

Initial loss: 9.21253795551967

Initial test loss: 9.21253795551967

Time since start: 2959.603

500: time = 20.674000 loss = 6.959426 testLoss 7.714490

Jackknife: leave t_sand out

Making features

makeFeatures: max memory 259522560, total allocated 139407360, free 54063080, used 85344280, increment -8223400

Amomum_tsaoko_7 t_sand:

Regularization values: linear/quadratic/product: 0.385, categorical: 0.250, threshold: 1.770, hinge: 0.500

23 samples

Density: max memory 259522560, total allocated 139407360, free 33253552, used 106153808, increment 20809528

linearPredictor: max memory 259522560, total allocated 139407360, free 33253552, used 106153808, increment 0

Deactivating (awc_class=2.0)

Deactivating (awc_class=3.0)

Deactivating (awc_class=4.0)

Deactivating (awc_class=6.0)

FeaturedSpace: max memory 259522560, total allocated 139407360, free 33253552, used 106153808, increment 0

Sequential: max memory 259522560, total allocated 139407360, free 33253552, used 106153808, increment 0

Initial loss: 9.21253795551967

Initial test loss: 9.21253795551967

Time since start: 2979.829

500: time = 19.903000 loss = 6.970187 testLoss 7.635332

Jackknife: only aspect

Making features

makeFeatures: max memory 259522560, total allocated 139407360, free 48864136, used 90543224, increment -15610584

Amomum_tsaoko_7 aspect:

Regularization values: linear/quadratic/product: 0.385, categorical: 0.250, threshold: 1.770, hinge: 0.500

23 samples

Density: max memory 259522560, total allocated 139407360, free 46235840, used 93171520, increment 2628296

linearPredictor: max memory 259522560, total allocated 139407360, free 46235840, used 93171520, increment 0

FeaturedSpace: max memory 259522560, total allocated 139407360, free 46235840, used 93171520, increment 0

Sequential: max memory 259522560, total allocated 139407360, free 46235840, used 93171520, increment 0

Initial loss: 9.21253795551967

Initial test loss: 9.21253795551967

Time since start: 2982.932

280: time = 3.023000 loss = 9.083583 testLoss 9.032168

Res.gain: 0.1289552708642816

Jackknife: only awc_class

Making features

makeFeatures: max memory 259522560, total allocated 139407360, free 25211824, used 114195536, increment 21024016

Amomum_tsaoko_7 awc_class:

Regularization values: linear/quadratic/product: 0.385, categorical: 0.250, threshold: 1.770, hinge: 0.500

23 samples

Density: max memory 259522560, total allocated 139407360, free 25195896, used 114211464, increment 15928

linearPredictor: max memory 259522560, total allocated 139407360, free 25195896, used 114211464, increment 0

Deactivating (awc_class=2.0)

Deactivating (awc_class=3.0)

Deactivating (awc_class=4.0)

Deactivating (awc_class=6.0)

FeaturedSpace: max memory 259522560, total allocated 139407360, free 25195896, used 114211464, increment 0

Sequential: max memory 259522560, total allocated 139407360, free 25195896, used 114211464, increment 0

Initial loss: 9.21253795551967

Initial test loss: 9.21253795551967

100: time = 0.174000 loss = 9.196540 testLoss 9.068659

Res.gain: 0.015997823449556137

Jackknife: only bio_12

Making features

makeFeatures: max memory 259522560, total allocated 139407360, free 23778208, used 115629152, increment 1417688

Amomum_tsaoko_7 bio_12:

Regularization values: linear/quadratic/product: 0.385, categorical: 0.250, threshold: 1.770, hinge: 0.500

23 samples

Density: max memory 259522560, total allocated 139407360, free 22875152, used 116532208, increment 903056

linearPredictor: max memory 259522560, total allocated 139407360, free 22794960, used 116612400, increment 80192

FeaturedSpace: max memory 259522560, total allocated 139407360, free 22794960, used 116612400, increment 0

Sequential: max memory 259522560, total allocated 139407360, free 22794960, used 116612400, increment 0

Initial loss: 9.21253795551967

Initial test loss: 9.21253795551967

Time since start: 2984.204

220: time = 1.046000 loss = 8.012855 testLoss 8.337432

Res.gain: 1.1996825551398818

Jackknife: only bio_15

Making features

makeFeatures: max memory 259522560, total allocated 139407360, free 51983960, used 87423400, increment -29189000

Amomum_tsaoko_7 bio_15:

Regularization values: linear/quadratic/product: 0.385, categorical: 0.250, threshold: 1.770, hinge: 0.500

23 samples

Density: max memory 259522560, total allocated 139407360, free 48893736, used 90513624, increment 3090224

linearPredictor: max memory 259522560, total allocated 139407360, free 48893736, used 90513624, increment 0

FeaturedSpace: max memory 259522560, total allocated 139407360, free 48893736, used 90513624, increment 0

Sequential: max memory 259522560, total allocated 139407360, free 48893736, used 90513624, increment 0

Initial loss: 9.21253795551967

Initial test loss: 9.21253795551967

Time since start: 2989.045

500: time = 4.814000 loss = 8.771239 testLoss 8.917711

Res.gain: 0.4412991149911516

Jackknife: only bio_17

Making features

makeFeatures: max memory 259522560, total allocated 139407360, free 53697480, used 85709880, increment -4803744

Amomum_tsaoko_7 bio_17:

Regularization values: linear/quadratic/product: 0.385, categorical: 0.250, threshold: 1.770, hinge: 0.500

23 samples

Density: max memory 259522560, total allocated 139407360, free 52914728, used 86492632, increment 782752

linearPredictor: max memory 259522560, total allocated 139407360, free 52914728, used 86492632, increment 0

FeaturedSpace: max memory 259522560, total allocated 139407360, free 52914728, used 86492632, increment 0

Sequential: max memory 259522560, total allocated 139407360, free 52914728, used 86492632, increment 0

Initial loss: 9.21253795551967

Initial test loss: 9.21253795551967

Time since start: 2990.176

300: time = 1.099000 loss = 8.012343 testLoss 9.216735

Res.gain: 1.2001948614080487

Jackknife: only bio_4

Making features

makeFeatures: max memory 259522560, total allocated 139407360, free 35965344, used 103442016, increment 16949384

Amomum_tsaoko_7 bio_4:

Regularization values: linear/quadratic/product: 0.385, categorical: 0.250, threshold: 1.770, hinge: 0.500

23 samples

Density: max memory 259522560, total allocated 139407360, free 33042264, used 106365096, increment 2923080

linearPredictor: max memory 259522560, total allocated 139407360, free 33042264, used 106365096, increment 0

FeaturedSpace: max memory 259522560, total allocated 139407360, free 33042264, used 106365096, increment 0

Sequential: max memory 259522560, total allocated 139407360, free 33042264, used 106365096, increment 0

Initial loss: 9.21253795551967

Initial test loss: 9.21253795551967

Time since start: 2991.534

120: time = 1.324000 loss = 7.733019 testLoss 8.041343

Res.gain: 1.4795189239488407

Jackknife: only bio_6

Making features

makeFeatures: max memory 259522560, total allocated 139407360, free 17630904, used 121776456, increment 15411360

Amomum_tsaoko_7 bio_6:

Regularization values: linear/quadratic/product: 0.385, categorical: 0.250, threshold: 1.770, hinge: 0.500

23 samples

Density: max memory 259522560, total allocated 139407360, free 15865696, used 123541664, increment 1765208

linearPredictor: max memory 259522560, total allocated 139407360, free 15785504, used 123621856, increment 80192

FeaturedSpace: max memory 259522560, total allocated 139407360, free 15785504, used 123621856, increment 0

Sequential: max memory 259522560, total allocated 139407360, free 15785504, used 123621856, increment 0

Initial loss: 9.21253795551967

Initial test loss: 9.21253795551967

Time since start: 2992.99

180: time = 1.424000 loss = 7.933779 testLoss 7.943412

Res.gain: 1.2787586876665218

Jackknife: only elev

Making features

makeFeatures: max memory 259522560, total allocated 139407360, free 22203280, used 117204080, increment -6417776

Amomum_tsaoko_7 elev:

Regularization values: linear/quadratic/product: 0.385, categorical: 0.250, threshold: 1.770, hinge: 0.500

23 samples

Density: max memory 259522560, total allocated 139407360, free 20566872, used 118840488, increment 1636408

linearPredictor: max memory 259522560, total allocated 139407360, free 20566872, used 118840488, increment 0

FeaturedSpace: max memory 259522560, total allocated 139407360, free 20566872, used 118840488, increment 0

Sequential: max memory 259522560, total allocated 139407360, free 20566872, used 118840488, increment 0

Initial loss: 9.21253795551967

Initial test loss: 9.21253795551967

100: time = 0.792000 loss = 9.068528 testLoss 9.507784

Res.gain: 0.14400948920119028

Jackknife: only s_caco3

Making features

makeFeatures: max memory 259522560, total allocated 139407360, free 34984416, used 104422944, increment -14417544

Amomum_tsaoko_7 s_caco3:

Regularization values: linear/quadratic/product: 0.385, categorical: 0.250, threshold: 1.770, hinge: 0.500

23 samples

Density: max memory 259522560, total allocated 139407360, free 34207352, used 105200008, increment 777064

linearPredictor: max memory 259522560, total allocated 139407360, free 34207352, used 105200008, increment 0

FeaturedSpace: max memory 259522560, total allocated 139407360, free 34207352, used 105200008, increment 0

Sequential: max memory 259522560, total allocated 139407360, free 34207352, used 105200008, increment 0

Initial loss: 9.21253795551967

Initial test loss: 9.21253795551967

Time since start: 2994.549

120: time = 0.706000 loss = 8.713660 testLoss 8.954828

Res.gain: 0.49887812472639403

Jackknife: only s_ph_h2o

Making features

makeFeatures: max memory 259522560, total allocated 139407360, free 31149888, used 108257472, increment 3057464

Amomum_tsaoko_7 s_ph_h2o:

Regularization values: linear/quadratic/product: 0.385, categorical: 0.250, threshold: 1.770, hinge: 0.500

23 samples

Density: max memory 259522560, total allocated 139407360, free 30326152, used 109081208, increment 823736

linearPredictor: max memory 259522560, total allocated 139407360, free 30326152, used 109081208, increment 0

FeaturedSpace: max memory 259522560, total allocated 139407360, free 30326152, used 109081208, increment 0

Sequential: max memory 259522560, total allocated 139407360, free 30326152, used 109081208, increment 0

Initial loss: 9.21253795551967

Initial test loss: 9.21253795551967

200: time = 0.700000 loss = 8.686695 testLoss 8.852916

Res.gain: 0.5258425306944083

Jackknife: only slope

Making features

makeFeatures: max memory 259522560, total allocated 139407360, free 26577248, used 112830112, increment 3748904

Amomum_tsaoko_7 slope:

Regularization values: linear/quadratic/product: 0.385, categorical: 0.250, threshold: 1.770, hinge: 0.500

23 samples

Density: max memory 259522560, total allocated 139407360, free 24132088, used 115275272, increment 2445160

linearPredictor: max memory 259522560, total allocated 139407360, free 24132088, used 115275272, increment 0

FeaturedSpace: max memory 259522560, total allocated 139407360, free 24132088, used 115275272, increment 0

Sequential: max memory 259522560, total allocated 139407360, free 24132088, used 115275272, increment 0

Initial loss: 9.21253795551967

Initial test loss: 9.21253795551967

Time since start: 2997.0

200: time = 1.696000 loss = 9.091610 testLoss 8.935621

Res.gain: 0.12092795361604125

Jackknife: only t_clay

Making features

makeFeatures: max memory 259522560, total allocated 139407360, free 48705888, used 90701472, increment -24573800

Amomum_tsaoko_7 t_clay:

Regularization values: linear/quadratic/product: 0.385, categorical: 0.250, threshold: 1.770, hinge: 0.500

23 samples

Density: max memory 259522560, total allocated 139407360, free 47921800, used 91485560, increment 784088

linearPredictor: max memory 259522560, total allocated 139407360, free 47152872, used 92254488, increment 768928

FeaturedSpace: max memory 259522560, total allocated 139407360, free 47152872, used 92254488, increment 0

Sequential: max memory 259522560, total allocated 139407360, free 47152872, used 92254488, increment 0

Initial loss: 9.21253795551967

Initial test loss: 9.21253795551967

100: time = 0.463000 loss = 8.700937 testLoss 8.211529

Res.gain: 0.5116014139287373

Jackknife: only t_oc

Making features

makeFeatures: max memory 259522560, total allocated 139407360, free 45472448, used 93934912, increment 1680424

Amomum_tsaoko_7 t_oc:

Regularization values: linear/quadratic/product: 0.385, categorical: 0.250, threshold: 1.770, hinge: 0.500

23 samples

Density: max memory 259522560, total allocated 139407360, free 44533168, used 94874192, increment 939280

linearPredictor: max memory 259522560, total allocated 139407360, free 44452976, used 94954384, increment 80192

FeaturedSpace: max memory 259522560, total allocated 139407360, free 44452976, used 94954384, increment 0

Sequential: max memory 259522560, total allocated 139407360, free 44452976, used 94954384, increment 0

Initial loss: 9.21253795551967

Initial test loss: 9.21253795551967

80: time = 0.406000 loss = 8.815381 testLoss 8.769619

Res.gain: 0.397157452718627

Jackknife: only t_sand

Making features

makeFeatures: max memory 259522560, total allocated 139407360, free 41642152, used 97765208, increment 2810824

Amomum_tsaoko_7 t_sand:

Regularization values: linear/quadratic/product: 0.385, categorical: 0.250, threshold: 1.770, hinge: 0.500

23 samples

Density: max memory 259522560, total allocated 139407360, free 40858272, used 98549088, increment 783880

linearPredictor: max memory 259522560, total allocated 139407360, free 40089344, used 99318016, increment 768928

FeaturedSpace: max memory 259522560, total allocated 139407360, free 40089344, used 99318016, increment 0

Sequential: max memory 259522560, total allocated 139407360, free 40089344, used 99318016, increment 0

Initial loss: 9.21253795551967

Initial test loss: 9.21253795551967

Time since start: 2998.669

180: time = 0.734000 loss = 8.979608 testLoss 8.874623

Res.gain: 0.23292996628114437

getSamples: max memory 259522560, total allocated 139407360, free 23404568, used 116002792, increment 16684776

Making features

makeFeatures: max memory 259522560, total allocated 139407360, free 15133224, used 124274136, increment 8271344

Amomum_tsaoko_8:

Regularization values: linear/quadratic/product: 0.385, categorical: 0.250, threshold: 1.770, hinge: 0.500

23 samples

Density: max memory 259522560, total allocated 139407360, free 24431624, used 114975736, increment -9298400

linearPredictor: max memory 259522560, total allocated 139407360, free 24431624, used 114975736, increment 0

Deactivating (awc_class=2.0)

Deactivating (awc_class=3.0)

Deactivating (awc_class=4.0)

Deactivating (awc_class=6.0)

FeaturedSpace: max memory 259522560, total allocated 139407360, free 24431624, used 114975736, increment 0

Sequential: max memory 259522560, total allocated 139407360, free 24431624, used 114975736, increment 0

Initial loss: 9.21253795551967

Initial test loss: 9.21253795551967

Time since start: 3019.224

500: time = 19.996000 loss = 7.032210 testLoss 6.252195

Resulting gain: 2.1803280071136886

Projecting...

Writing file E:\Amomum_tsaoko\results2\Amomum_tsaoko_8.asc

Time since start: 3023.357

Writing E:\Amomum_tsaoko\results2\plots\Amomum_tsaoko_8.png

Time since start: 3024.832

Projecting...

Writing file E:\Amomum_tsaoko\results2\Amomum_tsaoko_8_LGM.asc

Writing file E:\Amomum_tsaoko\results2\Amomum_tsaoko_8_LGM_clamping.asc

Time since start: 3029.51

Writing E:\Amomum_tsaoko\results2\plots\Amomum_tsaoko_8_LGM.png

Time since start: 3030.918

Writing E:\Amomum_tsaoko\results2\plots\Amomum_tsaoko_8_LGM_clamping.png

Time since start: 3032.365

Writing file E:\Amomum_tsaoko\results2\Amomum_tsaoko_8_LGM_novel.asc

Writing file E:\Amomum_tsaoko\results2\Amomum_tsaoko_8_LGM_novel_limiting.asc

Time since start: 3036.9

Projecting...

Writing file E:\Amomum_tsaoko\results2\Amomum_tsaoko_8_MH.asc

Writing file E:\Amomum_tsaoko\results2\Amomum_tsaoko_8_MH_clamping.asc

Time since start: 3041.445

Writing E:\Amomum_tsaoko\results2\plots\Amomum_tsaoko_8_MH.png

Time since start: 3042.777

Writing E:\Amomum_tsaoko\results2\plots\Amomum_tsaoko_8_MH_clamping.png

Time since start: 3044.216

Writing file E:\Amomum_tsaoko\results2\Amomum_tsaoko_8_MH_novel.asc

Writing file E:\Amomum_tsaoko\results2\Amomum_tsaoko_8_MH_novel_limiting.asc

Time since start: 3048.637

Projecting...

Writing file E:\Amomum_tsaoko\results2\Amomum_tsaoko_8_SSP126-2050S.asc

Writing file E:\Amomum_tsaoko\results2\Amomum_tsaoko_8_SSP126-2050S_clamping.asc

Time since start: 3052.613

Writing E:\Amomum_tsaoko\results2\plots\Amomum_tsaoko_8_SSP126-2050S.png

Time since start: 3054.069

Writing E:\Amomum_tsaoko\results2\plots\Amomum_tsaoko_8_SSP126-2050S_clamping.png

Time since start: 3055.448

Writing file E:\Amomum_tsaoko\results2\Amomum_tsaoko_8_SSP126-2050S_novel.asc

Writing file E:\Amomum_tsaoko\results2\Amomum_tsaoko_8_SSP126-2050S_novel_limiting.asc

Time since start: 3059.983

Projecting...

Writing file E:\Amomum_tsaoko\results2\Amomum_tsaoko_8_SSP126-2090S.asc

Writing file E:\Amomum_tsaoko\results2\Amomum_tsaoko_8_SSP126-2090S_clamping.asc

Time since start: 3063.929

Writing E:\Amomum_tsaoko\results2\plots\Amomum_tsaoko_8_SSP126-2090S.png

Time since start: 3065.415

Writing E:\Amomum_tsaoko\results2\plots\Amomum_tsaoko_8_SSP126-2090S_clamping.png

Time since start: 3066.762

Writing file E:\Amomum_tsaoko\results2\Amomum_tsaoko_8_SSP126-2090S_novel.asc

Writing file E:\Amomum_tsaoko\results2\Amomum_tsaoko_8_SSP126-2090S_novel_limiting.asc

Time since start: 3071.321

Projecting...

Writing file E:\Amomum_tsaoko\results2\Amomum_tsaoko_8_SSP585-2050S.asc

Writing file E:\Amomum_tsaoko\results2\Amomum_tsaoko_8_SSP585-2050S_clamping.asc

Time since start: 3075.283

Writing E:\Amomum_tsaoko\results2\plots\Amomum_tsaoko_8_SSP585-2050S.png

Time since start: 3076.759

Writing E:\Amomum_tsaoko\results2\plots\Amomum_tsaoko_8_SSP585-2050S_clamping.png

Time since start: 3078.235

Writing file E:\Amomum_tsaoko\results2\Amomum_tsaoko_8_SSP585-2050S_novel.asc

Writing file E:\Amomum_tsaoko\results2\Amomum_tsaoko_8_SSP585-2050S_novel_limiting.asc

Time since start: 3082.88

Projecting...

Writing file E:\Amomum_tsaoko\results2\Amomum_tsaoko_8_SSP585-2090S.asc

Writing file E:\Amomum_tsaoko\results2\Amomum_tsaoko_8_SSP585-2090S_clamping.asc

Time since start: 3086.685

Writing E:\Amomum_tsaoko\results2\plots\Amomum_tsaoko_8_SSP585-2090S.png

Time since start: 3088.03

Writing E:\Amomum_tsaoko\results2\plots\Amomum_tsaoko_8_SSP585-2090S_clamping.png

Time since start: 3089.355

Writing file E:\Amomum_tsaoko\results2\Amomum_tsaoko_8_SSP585-2090S_novel.asc

Writing file E:\Amomum_tsaoko\results2\Amomum_tsaoko_8_SSP585-2090S_novel_limiting.asc

Time since start: 3093.859

Amomum_tsaoko_8 response curves

Response curve: only aspect

Making features

makeFeatures: max memory 259522560, total allocated 139407360, free 43123392, used 96283968, increment -18691768

Amomum_tsaoko_8 aspect:

Regularization values: linear/quadratic/product: 0.385, categorical: 0.250, threshold: 1.770, hinge: 0.500

23 samples

Density: max memory 259522560, total allocated 139407360, free 39932024, used 99475336, increment 3191368

linearPredictor: max memory 259522560, total allocated 139407360, free 39932024, used 99475336, increment 0

FeaturedSpace: max memory 259522560, total allocated 139407360, free 39932024, used 99475336, increment 0

Sequential: max memory 259522560, total allocated 139407360, free 39932024, used 99475336, increment 0

Initial loss: 9.21253795551967

Time since start: 3096.575

160: time = 1.736000 loss = 9.108221

Resulting gain: 0.10431696560752002

Amomum_tsaoko_8 response curves

Response curve: only awc_class

Making features

makeFeatures: max memory 259522560, total allocated 139407360, free 66345040, used 73062320, increment -26413016

Amomum_tsaoko_8 awc_class:

Regularization values: linear/quadratic/product: 0.385, categorical: 0.250, threshold: 1.770, hinge: 0.500

23 samples

Density: max memory 259522560, total allocated 139407360, free 66334896, used 73072464, increment 10144

linearPredictor: max memory 259522560, total allocated 139407360, free 66334896, used 73072464, increment 0

Deactivating (awc_class=2.0)

Deactivating (awc_class=3.0)

Deactivating (awc_class=4.0)

Deactivating (awc_class=6.0)

FeaturedSpace: max memory 259522560, total allocated 139407360, free 66334896, used 73072464, increment 0

Sequential: max memory 259522560, total allocated 139407360, free 66334896, used 73072464, increment 0

Initial loss: 9.21253795551967

100: time = 0.185000 loss = 9.168549

Resulting gain: 0.0439888397479713

Amomum_tsaoko_8 response curves

Response curve: only bio_12

Making features

makeFeatures: max memory 259522560, total allocated 139407360, free 61673024, used 77734336, increment 4661872

Amomum_tsaoko_8 bio_12:

Regularization values: linear/quadratic/product: 0.385, categorical: 0.250, threshold: 1.770, hinge: 0.500

23 samples

Density: max memory 259522560, total allocated 139407360, free 60119328, used 79288032, increment 1553696

linearPredictor: max memory 259522560, total allocated 139407360, free 60119328, used 79288032, increment 0

FeaturedSpace: max memory 259522560, total allocated 139407360, free 60119328, used 79288032, increment 0

Sequential: max memory 259522560, total allocated 139407360, free 60119328, used 79288032, increment 0

Initial loss: 9.21253795551967

100: time = 0.521000 loss = 8.107054

Resulting gain: 1.1054837540300735

Amomum_tsaoko_8 response curves

Response curve: only bio_15

Making features

makeFeatures: max memory 259522560, total allocated 139407360, free 55439664, used 83967696, increment 4679664

Amomum_tsaoko_8 bio_15:

Regularization values: linear/quadratic/product: 0.385, categorical: 0.250, threshold: 1.770, hinge: 0.500

23 samples

Density: max memory 259522560, total allocated 139407360, free 52784576, used 86622784, increment 2655088

linearPredictor: max memory 259522560, total allocated 139407360, free 52784576, used 86622784, increment 0

FeaturedSpace: max memory 259522560, total allocated 139407360, free 52784576, used 86622784, increment 0

Sequential: max memory 259522560, total allocated 139407360, free 52784576, used 86622784, increment 0

Initial loss: 9.21253795551967

Time since start: 3101.415

420: time = 3.885000 loss = 8.839483

Resulting gain: 0.3730547493518195

Amomum_tsaoko_8 response curves

Response curve: only bio_17

Making features

makeFeatures: max memory 259522560, total allocated 139407360, free 52322656, used 87084704, increment 461920

Amomum_tsaoko_8 bio_17:

Regularization values: linear/quadratic/product: 0.385, categorical: 0.250, threshold: 1.770, hinge: 0.500

23 samples

Density: max memory 259522560, total allocated 139407360, free 51539328, used 87868032, increment 783328

linearPredictor: max memory 259522560, total allocated 139407360, free 51539328, used 87868032, increment 0

FeaturedSpace: max memory 259522560, total allocated 139407360, free 51539328, used 87868032, increment 0

Sequential: max memory 259522560, total allocated 139407360, free 50768624, used 88638736, increment 770704

Initial loss: 9.21253795551967

Time since start: 3102.597

340: time = 1.073000 loss = 7.868597

Resulting gain: 1.3439407500892013

Amomum_tsaoko_8 response curves

Response curve: only bio_4

Making features

makeFeatures: max memory 259522560, total allocated 139407360, free 66370944, used 73036416, increment -15602320

Amomum_tsaoko_8 bio_4:

Regularization values: linear/quadratic/product: 0.385, categorical: 0.250, threshold: 1.770, hinge: 0.500

23 samples

Density: max memory 259522560, total allocated 139407360, free 63165992, used 76241368, increment 3204952

linearPredictor: max memory 259522560, total allocated 139407360, free 63085800, used 76321560, increment 80192

FeaturedSpace: max memory 259522560, total allocated 139407360, free 63085800, used 76321560, increment 0

Sequential: max memory 259522560, total allocated 139407360, free 63085800, used 76321560, increment 0

Initial loss: 9.21253795551967

Time since start: 3104.047

120: time = 1.362000 loss = 7.834963

Resulting gain: 1.3775748628071272

Amomum_tsaoko_8 response curves

Response curve: only bio_6

Making features

makeFeatures: max memory 259522560, total allocated 139407360, free 45640224, used 93767136, increment 17445576

Amomum_tsaoko_8 bio_6:

Regularization values: linear/quadratic/product: 0.385, categorical: 0.250, threshold: 1.770, hinge: 0.500

23 samples

Density: max memory 259522560, total allocated 139407360, free 43309280, used 96098080, increment 2330944

linearPredictor: max memory 259522560, total allocated 139407360, free 43309280, used 96098080, increment 0

FeaturedSpace: max memory 259522560, total allocated 139407360, free 43309280, used 96098080, increment 0

Sequential: max memory 259522560, total allocated 139407360, free 43309280, used 96098080, increment 0

Initial loss: 9.21253795551967

Time since start: 3105.633

200: time = 1.493000 loss = 7.732287

Resulting gain: 1.4802509230723455

Amomum_tsaoko_8 response curves

Response curve: only elev

Making features

makeFeatures: max memory 259522560, total allocated 139407360, free 58875008, used 80532352, increment -15565728

Amomum_tsaoko_8 elev:

Regularization values: linear/quadratic/product: 0.385, categorical: 0.250, threshold: 1.770, hinge: 0.500

23 samples

Density: max memory 259522560, total allocated 139407360, free 57272016, used 82135344, increment 1602992

linearPredictor: max memory 259522560, total allocated 139407360, free 57272016, used 82135344, increment 0

FeaturedSpace: max memory 259522560, total allocated 139407360, free 57272016, used 82135344, increment 0

Sequential: max memory 259522560, total allocated 139407360, free 57272016, used 82135344, increment 0

Initial loss: 9.21253795551967

100: time = 0.610000 loss = 9.093581

Resulting gain: 0.11895662204089241

Amomum_tsaoko_8 response curves

Response curve: only s_caco3

Making features

makeFeatures: max memory 259522560, total allocated 139407360, free 68361720, used 71045640, increment -11089704

Amomum_tsaoko_8 s_caco3:

Regularization values: linear/quadratic/product: 0.385, categorical: 0.250, threshold: 1.770, hinge: 0.500

23 samples

Density: max memory 259522560, total allocated 139407360, free 67459704, used 71947656, increment 902016

linearPredictor: max memory 259522560, total allocated 139407360, free 67379512, used 72027848, increment 80192

FeaturedSpace: max memory 259522560, total allocated 139407360, free 67379512, used 72027848, increment 0

Sequential: max memory 259522560, total allocated 139407360, free 67379512, used 72027848, increment 0

Initial loss: 9.21253795551967

Time since start: 3107.126

140: time = 0.696000 loss = 8.672079

Resulting gain: 0.5404590636596396

Amomum_tsaoko_8 response curves

Response curve: only s_ph_h2o

Making features

makeFeatures: max memory 259522560, total allocated 139407360, free 60091968, used 79315392, increment 7287544

Amomum_tsaoko_8 s_ph_h2o:

Regularization values: linear/quadratic/product: 0.385, categorical: 0.250, threshold: 1.770, hinge: 0.500

23 samples

Density: max memory 259522560, total allocated 139407360, free 59304536, used 80102824, increment 787432

linearPredictor: max memory 259522560, total allocated 139407360, free 59304536, used 80102824, increment 0

FeaturedSpace: max memory 259522560, total allocated 139407360, free 59304536, used 80102824, increment 0

Sequential: max memory 259522560, total allocated 139407360, free 59304536, used 80102824, increment 0

Initial loss: 9.21253795551967

140: time = 0.538000 loss = 8.355955

Resulting gain: 0.8565833217156111

Amomum_tsaoko_8 response curves

Response curve: only slope

Making features

makeFeatures: max memory 259522560, total allocated 139407360, free 52661968, used 86745392, increment 6642568

Amomum_tsaoko_8 slope:

Regularization values: linear/quadratic/product: 0.385, categorical: 0.250, threshold: 1.770, hinge: 0.500

23 samples

Density: max memory 259522560, total allocated 139407360, free 50124488, used 89282872, increment 2537480

linearPredictor: max memory 259522560, total allocated 139407360, free 50124488, used 89282872, increment 0

FeaturedSpace: max memory 259522560, total allocated 139407360, free 50124488, used 89282872, increment 0

Sequential: max memory 259522560, total allocated 139407360, free 50124488, used 89282872, increment 0

Initial loss: 9.21253795551967

Time since start: 3110.982

340: time = 3.144000 loss = 8.963021

Resulting gain: 0.24951701501638368

Amomum_tsaoko_8 response curves

Response curve: only t_clay

Making features

makeFeatures: max memory 259522560, total allocated 139407360, free 55929256, used 83478104, increment -5804768

Amomum_tsaoko_8 t_clay:

Regularization values: linear/quadratic/product: 0.385, categorical: 0.250, threshold: 1.770, hinge: 0.500

23 samples

Density: max memory 259522560, total allocated 139407360, free 55108128, used 84299232, increment 821128

linearPredictor: max memory 259522560, total allocated 139407360, free 55108128, used 84299232, increment 0

FeaturedSpace: max memory 259522560, total allocated 139407360, free 55108128, used 84299232, increment 0

Sequential: max memory 259522560, total allocated 139407360, free 55108128, used 84299232, increment 0

Initial loss: 9.21253795551967

140: time = 0.633000 loss = 8.814500

Resulting gain: 0.3980377640878139

Amomum_tsaoko_8 response curves

Response curve: only t_oc

Making features

makeFeatures: max memory 259522560, total allocated 139407360, free 48464848, used 90942512, increment 6643280

Amomum_tsaoko_8 t_oc:

Regularization values: linear/quadratic/product: 0.385, categorical: 0.250, threshold: 1.770, hinge: 0.500

23 samples

Density: max memory 259522560, total allocated 139407360, free 47680360, used 91727000, increment 784488

linearPredictor: max memory 259522560, total allocated 139407360, free 47680360, used 91727000, increment 0

FeaturedSpace: max memory 259522560, total allocated 139407360, free 47680360, used 91727000, increment 0

Sequential: max memory 259522560, total allocated 139407360, free 47680360, used 91727000, increment 0

Initial loss: 9.21253795551967

Time since start: 3112.46

160: time = 0.668000 loss = 8.723218

Resulting gain: 0.4893196486528133

Amomum_tsaoko_8 response curves

Response curve: only t_sand

Making features

makeFeatures: max memory 259522560, total allocated 139407360, free 38895376, used 100511984, increment 8784984

Amomum_tsaoko_8 t_sand:

Regularization values: linear/quadratic/product: 0.385, categorical: 0.250, threshold: 1.770, hinge: 0.500

23 samples

Density: max memory 259522560, total allocated 139407360, free 38114232, used 101293128, increment 781144

linearPredictor: max memory 259522560, total allocated 139407360, free 38114232, used 101293128, increment 0

FeaturedSpace: max memory 259522560, total allocated 139407360, free 38114232, used 101293128, increment 0

Sequential: max memory 259522560, total allocated 139407360, free 38114232, used 101293128, increment 0

Initial loss: 9.21253795551967

100: time = 0.453000 loss = 9.106196

Resulting gain: 0.1063414620131411

Amomum_tsaoko_8 response curves

Time since start: 3114.168

Jackknife: leave aspect out

Making features

makeFeatures: max memory 259522560, total allocated 139407360, free 61087280, used 78320080, increment -22973048

Amomum_tsaoko_8 aspect:

Regularization values: linear/quadratic/product: 0.385, categorical: 0.250, threshold: 1.770, hinge: 0.500

23 samples

Density: max memory 259522560, total allocated 139407360, free 42351440, used 97055920, increment 18735840

linearPredictor: max memory 259522560, total allocated 139407360, free 42351440, used 97055920, increment 0

Deactivating (awc_class=2.0)

Deactivating (awc_class=3.0)

Deactivating (awc_class=4.0)

Deactivating (awc_class=6.0)

FeaturedSpace: max memory 259522560, total allocated 139407360, free 42351440, used 97055920, increment 0

Sequential: max memory 259522560, total allocated 139407360, free 42351440, used 97055920, increment 0

Initial loss: 9.21253795551967

Initial test loss: 9.21253795551967

Time since start: 3130.112

500: time = 15.684000 loss = 7.087337 testLoss 6.359047

Jackknife: leave awc_class out

Making features

makeFeatures: max memory 259522560, total allocated 139407360, free 34680584, used 104726776, increment 7670856

Amomum_tsaoko_8 awc_class:

Regularization values: linear/quadratic/product: 0.385, categorical: 0.250, threshold: 1.770, hinge: 0.500

23 samples

Density: max memory 259522560, total allocated 139407360, free 35305552, used 104101808, increment -624968

linearPredictor: max memory 259522560, total allocated 139407360, free 35305552, used 104101808, increment 0

FeaturedSpace: max memory 259522560, total allocated 139407360, free 35305552, used 104101808, increment 0

Sequential: max memory 259522560, total allocated 139407360, free 35305552, used 104101808, increment 0

Initial loss: 9.21253795551967

Initial test loss: 9.21253795551967

Time since start: 3150.132

500: time = 19.653000 loss = 7.032701 testLoss 6.240200

Jackknife: leave bio_12 out

Making features

makeFeatures: max memory 259522560, total allocated 139407360, free 53979776, used 85427584, increment -18674224

Amomum_tsaoko_8 bio_12:

Regularization values: linear/quadratic/product: 0.385, categorical: 0.250, threshold: 1.770, hinge: 0.500

23 samples

Density: max memory 259522560, total allocated 139407360, free 64238808, used 75168552, increment -10259032

linearPredictor: max memory 259522560, total allocated 139407360, free 64158616, used 75248744, increment 80192

Deactivating (awc_class=2.0)

Deactivating (awc_class=3.0)

Deactivating (awc_class=4.0)

Deactivating (awc_class=6.0)

FeaturedSpace: max memory 259522560, total allocated 139407360, free 64158616, used 75248744, increment 0

Sequential: max memory 259522560, total allocated 139407360, free 64158616, used 75248744, increment 0

Initial loss: 9.21253795551967

Initial test loss: 9.21253795551967

Time since start: 3169.694

500: time = 19.220000 loss = 7.049666 testLoss 6.254274

Jackknife: leave bio_15 out

Making features

makeFeatures: max memory 259522560, total allocated 139407360, free 56609680, used 82797680, increment 7548936

Amomum_tsaoko_8 bio_15:

Regularization values: linear/quadratic/product: 0.385, categorical: 0.250, threshold: 1.770, hinge: 0.500

23 samples

Density: max memory 259522560, total allocated 139407360, free 37879600, used 101527760, increment 18730080

linearPredictor: max memory 259522560, total allocated 139407360, free 37879600, used 101527760, increment 0

Deactivating (awc_class=2.0)

Deactivating (awc_class=3.0)

Deactivating (awc_class=4.0)

Deactivating (awc_class=6.0)

FeaturedSpace: max memory 259522560, total allocated 139407360, free 37879600, used 101527760, increment 0

Sequential: max memory 259522560, total allocated 139407360, free 37879600, used 101527760, increment 0

Initial loss: 9.21253795551967

Initial test loss: 9.21253795551967

Time since start: 3186.423

500: time = 16.442000 loss = 7.062363 testLoss 6.282181

Jackknife: leave bio_17 out

Making features

makeFeatures: max memory 259522560, total allocated 139407360, free 24186416, used 115220944, increment 13693184

Amomum_tsaoko_8 bio_17:

Regularization values: linear/quadratic/product: 0.385, categorical: 0.250, threshold: 1.770, hinge: 0.500

23 samples

Density: max memory 259522560, total allocated 139407360, free 34623000, used 104784360, increment -10436584

linearPredictor: max memory 259522560, total allocated 139407360, free 34542808, used 104864552, increment 80192

Deactivating (awc_class=2.0)

Deactivating (awc_class=3.0)

Deactivating (awc_class=4.0)

Deactivating (awc_class=6.0)

FeaturedSpace: max memory 259522560, total allocated 139407360, free 34542808, used 104864552, increment 0

Sequential: max memory 259522560, total allocated 139407360, free 34542808, used 104864552, increment 0

Initial loss: 9.21253795551967

Initial test loss: 9.21253795551967

Time since start: 3205.956

500: time = 19.217000 loss = 7.129285 testLoss 6.472667

Jackknife: leave bio_4 out

Making features

makeFeatures: max memory 259522560, total allocated 139407360, free 18377384, used 121029976, increment 16165424

Amomum_tsaoko_8 bio_4:

Regularization values: linear/quadratic/product: 0.385, categorical: 0.250, threshold: 1.770, hinge: 0.500

23 samples

Density: max memory 259522560, total allocated 139407360, free 90745088, used 48662272, increment -72367704

linearPredictor: max memory 259522560, total allocated 139407360, free 90664896, used 48742464, increment 80192

Deactivating (awc_class=2.0)

Deactivating (awc_class=3.0)

Deactivating (awc_class=4.0)

Deactivating (awc_class=6.0)

FeaturedSpace: max memory 259522560, total allocated 139407360, free 90664896, used 48742464, increment 0

Sequential: max memory 259522560, total allocated 139407360, free 90664896, used 48742464, increment 0

Initial loss: 9.21253795551967

Initial test loss: 9.21253795551967

Time since start: 3221.679

480: time = 15.386000 loss = 7.037933 testLoss 6.305650

Jackknife: leave bio_6 out

Making features

makeFeatures: max memory 259522560, total allocated 139407360, free 86118256, used 53289104, increment 4546640

Amomum_tsaoko_8 bio_6:

Regularization values: linear/quadratic/product: 0.385, categorical: 0.250, threshold: 1.770, hinge: 0.500

23 samples

Density: max memory 259522560, total allocated 139407360, free 66780128, used 72627232, increment 19338128

linearPredictor: max memory 259522560, total allocated 139407360, free 66780128, used 72627232, increment 0

Deactivating (awc_class=2.0)

Deactivating (awc_class=3.0)

Deactivating (awc_class=4.0)

Deactivating (awc_class=6.0)

FeaturedSpace: max memory 259522560, total allocated 139407360, free 66780128, used 72627232, increment 0

Sequential: max memory 259522560, total allocated 139407360, free 66780128, used 72627232, increment 0

Initial loss: 9.21253795551967

Initial test loss: 9.21253795551967

Time since start: 3238.521

500: time = 16.550000 loss = 7.108000 testLoss 6.281871

Jackknife: leave elev out

Making features

makeFeatures: max memory 259522560, total allocated 139407360, free 58398512, used 81008848, increment 8381616

Amomum_tsaoko_8 elev:

Regularization values: linear/quadratic/product: 0.385, categorical: 0.250, threshold: 1.770, hinge: 0.500

23 samples

Density: max memory 259522560, total allocated 139407360, free 62215288, used 77192072, increment -3816776

linearPredictor: max memory 259522560, total allocated 139407360, free 62215288, used 77192072, increment 0

Deactivating (awc_class=2.0)

Deactivating (awc_class=3.0)

Deactivating (awc_class=4.0)

Deactivating (awc_class=6.0)

FeaturedSpace: max memory 259522560, total allocated 139407360, free 62215288, used 77192072, increment 0

Sequential: max memory 259522560, total allocated 139407360, free 62215288, used 77192072, increment 0

Initial loss: 9.21253795551967

Initial test loss: 9.21253795551967

Time since start: 3256.377

500: time = 17.563000 loss = 7.048539 testLoss 6.353060

Jackknife: leave s_caco3 out

Making features

makeFeatures: max memory 259522560, total allocated 139407360, free 60627696, used 78779664, increment 1587592

Amomum_tsaoko_8 s_caco3:

Regularization values: linear/quadratic/product: 0.385, categorical: 0.250, threshold: 1.770, hinge: 0.500

23 samples

Density: max memory 259522560, total allocated 139407360, free 40596832, used 98810528, increment 20030864

linearPredictor: max memory 259522560, total allocated 139407360, free 40516640, used 98890720, increment 80192

Deactivating (awc_class=2.0)

Deactivating (awc_class=3.0)

Deactivating (awc_class=4.0)

Deactivating (awc_class=6.0)

FeaturedSpace: max memory 259522560, total allocated 139407360, free 40516640, used 98890720, increment 0

Sequential: max memory 259522560, total allocated 139407360, free 40516640, used 98890720, increment 0

Initial loss: 9.21253795551967

Initial test loss: 9.21253795551967

Time since start: 3275.397

500: time = 18.734000 loss = 7.096067 testLoss 6.338744

Jackknife: leave s_ph_h2o out

Making features

makeFeatures: max memory 259522560, total allocated 139407360, free 45787344, used 93620016, increment -5270704

Amomum_tsaoko_8 s_ph_h2o:

Regularization values: linear/quadratic/product: 0.385, categorical: 0.250, threshold: 1.770, hinge: 0.500

23 samples

Density: max memory 259522560, total allocated 139407360, free 25008912, used 114398448, increment 20778432

linearPredictor: max memory 259522560, total allocated 139407360, free 25008912, used 114398448, increment 0

Deactivating (awc_class=2.0)

Deactivating (awc_class=3.0)

Deactivating (awc_class=4.0)

Deactivating (awc_class=6.0)

FeaturedSpace: max memory 259522560, total allocated 139407360, free 25008912, used 114398448, increment 0

Sequential: max memory 259522560, total allocated 139407360, free 25008912, used 114398448, increment 0

Initial loss: 9.21253795551967

Initial test loss: 9.21253795551967

Time since start: 3294.923

500: time = 19.233000 loss = 7.032153 testLoss 6.244452

Jackknife: leave slope out

Making features

makeFeatures: max memory 259522560, total allocated 139407360, free 86693632, used 52713728, increment -61684720

Amomum_tsaoko_8 slope:

Regularization values: linear/quadratic/product: 0.385, categorical: 0.250, threshold: 1.770, hinge: 0.500

23 samples

Density: max memory 259522560, total allocated 139407360, free 67392816, used 72014544, increment 19300816

linearPredictor: max memory 259522560, total allocated 139407360, free 67392816, used 72014544, increment 0

Deactivating (awc_class=2.0)

Deactivating (awc_class=3.0)

Deactivating (awc_class=4.0)

Deactivating (awc_class=6.0)

FeaturedSpace: max memory 259522560, total allocated 139407360, free 67392816, used 72014544, increment 0

Sequential: max memory 259522560, total allocated 139407360, free 67392816, used 72014544, increment 0

Initial loss: 9.21253795551967

Initial test loss: 9.21253795551967

Time since start: 3312.703

500: time = 17.471000 loss = 7.033461 testLoss 6.259257

Jackknife: leave t_clay out

Making features

makeFeatures: max memory 259522560, total allocated 139407360, free 46425544, used 92981816, increment 20967272

Amomum_tsaoko_8 t_clay:

Regularization values: linear/quadratic/product: 0.385, categorical: 0.250, threshold: 1.770, hinge: 0.500

23 samples

Density: max memory 259522560, total allocated 139407360, free 55337392, used 84069968, increment -8911848

linearPredictor: max memory 259522560, total allocated 139407360, free 55337392, used 84069968, increment 0

Deactivating (awc_class=2.0)

Deactivating (awc_class=3.0)

Deactivating (awc_class=4.0)

Deactivating (awc_class=6.0)

FeaturedSpace: max memory 259522560, total allocated 139407360, free 55337392, used 84069968, increment 0

Sequential: max memory 259522560, total allocated 139407360, free 55337392, used 84069968, increment 0

Initial loss: 9.21253795551967

Initial test loss: 9.21253795551967

Time since start: 3332.633

500: time = 19.611000 loss = 7.034139 testLoss 6.274744

Jackknife: leave t_oc out

Making features

makeFeatures: max memory 259522560, total allocated 139407360, free 59401512, used 80005848, increment -4064120

Amomum_tsaoko_8 t_oc:

Regularization values: linear/quadratic/product: 0.385, categorical: 0.250, threshold: 1.770, hinge: 0.500

23 samples

Density: max memory 259522560, total allocated 139407360, free 39617328, used 99790032, increment 19784184

linearPredictor: max memory 259522560, total allocated 139407360, free 38854112, used 100553248, increment 763216

Deactivating (awc_class=2.0)

Deactivating (awc_class=3.0)

Deactivating (awc_class=4.0)

Deactivating (awc_class=6.0)

FeaturedSpace: max memory 259522560, total allocated 139407360, free 38854112, used 100553248, increment 0

Sequential: max memory 259522560, total allocated 139407360, free 38854112, used 100553248, increment 0

Initial loss: 9.21253795551967

Initial test loss: 9.21253795551967

Time since start: 3352.111

500: time = 19.172000 loss = 7.053123 testLoss 6.245179

Jackknife: leave t_sand out

Making features

makeFeatures: max memory 259522560, total allocated 139407360, free 44812456, used 94594904, increment -5958344

Amomum_tsaoko_8 t_sand:

Regularization values: linear/quadratic/product: 0.385, categorical: 0.250, threshold: 1.770, hinge: 0.500

23 samples

Density: max memory 259522560, total allocated 139407360, free 24546768, used 114860592, increment 20265688

linearPredictor: max memory 259522560, total allocated 139407360, free 24466576, used 114940784, increment 80192

Deactivating (awc_class=2.0)

Deactivating (awc_class=3.0)

Deactivating (awc_class=4.0)

Deactivating (awc_class=6.0)

FeaturedSpace: max memory 259522560, total allocated 139407360, free 24466576, used 114940784, increment 0

Sequential: max memory 259522560, total allocated 139407360, free 24466576, used 114940784, increment 0

Initial loss: 9.21253795551967

Initial test loss: 9.21253795551967

Time since start: 3371.156

500: time = 18.748000 loss = 7.032333 testLoss 6.253431

Jackknife: only aspect

Making features

makeFeatures: max memory 259522560, total allocated 139407360, free 59515448, used 79891912, increment -35048872

Amomum_tsaoko_8 aspect:

Regularization values: linear/quadratic/product: 0.385, categorical: 0.250, threshold: 1.770, hinge: 0.500

23 samples

Density: max memory 259522560, total allocated 139407360, free 94542616, used 44864744, increment -35027168

linearPredictor: max memory 259522560, total allocated 139407360, free 94462424, used 44944936, increment 80192

FeaturedSpace: max memory 259522560, total allocated 139407360, free 94462424, used 44944936, increment 0

Sequential: max memory 259522560, total allocated 139407360, free 94462424, used 44944936, increment 0

Initial loss: 9.21253795551967

Initial test loss: 9.21253795551967

Time since start: 3372.935

160: time = 1.715000 loss = 9.108221 testLoss 9.070360

Res.gain: 0.10431696560752002

Jackknife: only awc_class

Making features

makeFeatures: max memory 259522560, total allocated 139407360, free 88241680, used 51165680, increment 6220744

Amomum_tsaoko_8 awc_class:

Regularization values: linear/quadratic/product: 0.385, categorical: 0.250, threshold: 1.770, hinge: 0.500

23 samples

Density: max memory 259522560, total allocated 139407360, free 88151152, used 51256208, increment 90528

linearPredictor: max memory 259522560, total allocated 139407360, free 88070960, used 51336400, increment 80192

Deactivating (awc_class=2.0)

Deactivating (awc_class=3.0)

Deactivating (awc_class=4.0)

Deactivating (awc_class=6.0)

FeaturedSpace: max memory 259522560, total allocated 139407360, free 88070960, used 51336400, increment 0

Sequential: max memory 259522560, total allocated 139407360, free 88070960, used 51336400, increment 0

Initial loss: 9.21253795551967

Initial test loss: 9.21253795551967

100: time = 0.160000 loss = 9.168549 testLoss 9.266156

Res.gain: 0.0439888397479713

Jackknife: only bio_12

Making features

makeFeatures: max memory 259522560, total allocated 139407360, free 86678008, used 52729352, increment 1392952

Amomum_tsaoko_8 bio_12:

Regularization values: linear/quadratic/product: 0.385, categorical: 0.250, threshold: 1.770, hinge: 0.500

23 samples

Density: max memory 259522560, total allocated 139407360, free 85005920, used 54401440, increment 1672088

linearPredictor: max memory 259522560, total allocated 139407360, free 85005920, used 54401440, increment 0

FeaturedSpace: max memory 259522560, total allocated 139407360, free 85005920, used 54401440, increment 0

Sequential: max memory 259522560, total allocated 139407360, free 85005920, used 54401440, increment 0

Initial loss: 9.21253795551967

Initial test loss: 9.21253795551967

100: time = 0.556000 loss = 8.107054 testLoss 7.819972

Res.gain: 1.1054837540300735

Jackknife: only bio_15

Making features

makeFeatures: max memory 259522560, total allocated 139407360, free 84082216, used 55325144, increment 923704

Amomum_tsaoko_8 bio_15:

Regularization values: linear/quadratic/product: 0.385, categorical: 0.250, threshold: 1.770, hinge: 0.500

23 samples

Density: max memory 259522560, total allocated 139407360, free 80868416, used 58538944, increment 3213800

linearPredictor: max memory 259522560, total allocated 139407360, free 80868416, used 58538944, increment 0

FeaturedSpace: max memory 259522560, total allocated 139407360, free 80868416, used 58538944, increment 0

Sequential: max memory 259522560, total allocated 139407360, free 80868416, used 58538944, increment 0

Initial loss: 9.21253795551967

Initial test loss: 9.21253795551967

Time since start: 3377.464

420: time = 3.760000 loss = 8.839483 testLoss 8.506122

Res.gain: 0.3730547493518195

Jackknife: only bio_17

Making features

makeFeatures: max memory 259522560, total allocated 139407360, free 84331128, used 55076232, increment -3462712

Amomum_tsaoko_8 bio_17:

Regularization values: linear/quadratic/product: 0.385, categorical: 0.250, threshold: 1.770, hinge: 0.500

23 samples

Density: max memory 259522560, total allocated 139407360, free 83346968, used 56060392, increment 984160

linearPredictor: max memory 259522560, total allocated 139407360, free 83346968, used 56060392, increment 0

FeaturedSpace: max memory 259522560, total allocated 139407360, free 83346968, used 56060392, increment 0

Sequential: max memory 259522560, total allocated 139407360, free 83346968, used 56060392, increment 0

Initial loss: 9.21253795551967

Initial test loss: 9.21253795551967

Time since start: 3378.648

340: time = 1.152000 loss = 7.868597 testLoss 7.089038

Res.gain: 1.3439407500892013

Jackknife: only bio_4

Making features

makeFeatures: max memory 259522560, total allocated 139407360, free 64542696, used 74864664, increment 18804272

Amomum_tsaoko_8 bio_4:

Regularization values: linear/quadratic/product: 0.385, categorical: 0.250, threshold: 1.770, hinge: 0.500

23 samples

Density: max memory 259522560, total allocated 139407360, free 61327000, used 78080360, increment 3215696

linearPredictor: max memory 259522560, total allocated 139407360, free 61327000, used 78080360, increment 0

FeaturedSpace: max memory 259522560, total allocated 139407360, free 61327000, used 78080360, increment 0

Sequential: max memory 259522560, total allocated 139407360, free 61327000, used 78080360, increment 0

Initial loss: 9.21253795551967

Initial test loss: 9.21253795551967

Time since start: 3380.299

120: time = 1.623000 loss = 7.834963 testLoss 7.663438

Res.gain: 1.3775748628071272

Jackknife: only bio_6

Making features

makeFeatures: max memory 259522560, total allocated 139407360, free 84451904, used 54955456, increment -23124904

Amomum_tsaoko_8 bio_6:

Regularization values: linear/quadratic/product: 0.385, categorical: 0.250, threshold: 1.770, hinge: 0.500

23 samples

Density: max memory 259522560, total allocated 139407360, free 82779432, used 56627928, increment 1672472

linearPredictor: max memory 259522560, total allocated 139407360, free 82699240, used 56708120, increment 80192

FeaturedSpace: max memory 259522560, total allocated 139407360, free 82699240, used 56708120, increment 0

Sequential: max memory 259522560, total allocated 139407360, free 82699240, used 56708120, increment 0

Initial loss: 9.21253795551967

Initial test loss: 9.21253795551967

Time since start: 3382.429

200: time = 2.093000 loss = 7.732287 testLoss 7.440190

Res.gain: 1.4802509230723455

Jackknife: only elev

Making features

makeFeatures: max memory 259522560, total allocated 139407360, free 63795968, used 75611392, increment 18903272

Amomum_tsaoko_8 elev:

Regularization values: linear/quadratic/product: 0.385, categorical: 0.250, threshold: 1.770, hinge: 0.500

23 samples

Density: max memory 259522560, total allocated 139407360, free 62245080, used 77162280, increment 1550888

linearPredictor: max memory 259522560, total allocated 139407360, free 62245080, used 77162280, increment 0

FeaturedSpace: max memory 259522560, total allocated 139407360, free 62245080, used 77162280, increment 0

Sequential: max memory 259522560, total allocated 139407360, free 62245080, used 77162280, increment 0

Initial loss: 9.21253795551967

Initial test loss: 9.21253795551967

100: time = 0.673000 loss = 9.093581 testLoss 8.920439

Res.gain: 0.11895662204089241

Jackknife: only s_caco3

Making features

makeFeatures: max memory 259522560, total allocated 139407360, free 77255696, used 62151664, increment -15010616

Amomum_tsaoko_8 s_caco3:

Regularization values: linear/quadratic/product: 0.385, categorical: 0.250, threshold: 1.770, hinge: 0.500

23 samples

Density: max memory 259522560, total allocated 139407360, free 76432256, used 62975104, increment 823440

linearPredictor: max memory 259522560, total allocated 139407360, free 76352064, used 63055296, increment 80192

FeaturedSpace: max memory 259522560, total allocated 139407360, free 76352064, used 63055296, increment 0

Sequential: max memory 259522560, total allocated 139407360, free 76352064, used 63055296, increment 0

Initial loss: 9.21253795551967

Initial test loss: 9.21253795551967

Time since start: 3383.92

140: time = 0.749000 loss = 8.672079 testLoss 8.649444

Res.gain: 0.5404590636596396

Jackknife: only s_ph_h2o

Making features

makeFeatures: max memory 259522560, total allocated 139407360, free 73108736, used 66298624, increment 3243328

Amomum_tsaoko_8 s_ph_h2o:

Regularization values: linear/quadratic/product: 0.385, categorical: 0.250, threshold: 1.770, hinge: 0.500

23 samples

Density: max memory 259522560, total allocated 139407360, free 72325512, used 67081848, increment 783224

linearPredictor: max memory 259522560, total allocated 139407360, free 72245320, used 67162040, increment 80192

FeaturedSpace: max memory 259522560, total allocated 139407360, free 72245320, used 67162040, increment 0

Sequential: max memory 259522560, total allocated 139407360, free 72245320, used 67162040, increment 0

Initial loss: 9.21253795551967

Initial test loss: 9.21253795551967

140: time = 0.570000 loss = 8.355955 testLoss 8.019856

Res.gain: 0.8565833217156111

Jackknife: only slope

Making features

makeFeatures: max memory 259522560, total allocated 139407360, free 68888848, used 70518512, increment 3356472

Amomum_tsaoko_8 slope:

Regularization values: linear/quadratic/product: 0.385, categorical: 0.250, threshold: 1.770, hinge: 0.500

23 samples

Density: max memory 259522560, total allocated 139407360, free 66459840, used 72947520, increment 2429008

linearPredictor: max memory 259522560, total allocated 139407360, free 66459840, used 72947520, increment 0

FeaturedSpace: max memory 259522560, total allocated 139407360, free 66459840, used 72947520, increment 0

Sequential: max memory 259522560, total allocated 139407360, free 66459840, used 72947520, increment 0

Initial loss: 9.21253795551967

Initial test loss: 9.21253795551967

Time since start: 3387.558

340: time = 3.022000 loss = 8.963021 testLoss 9.353072

Res.gain: 0.24951701501638368

Jackknife: only t_clay

Making features

makeFeatures: max memory 259522560, total allocated 139407360, free 76844352, used 62563008, increment -10384512

Amomum_tsaoko_8 t_clay:

Regularization values: linear/quadratic/product: 0.385, categorical: 0.250, threshold: 1.770, hinge: 0.500

23 samples
[truncated: 53,251 more chars]
