# Supplementary material for: Assessment of Chinese suitable habitats of Amomum tsao-ko in different climatic conditions
Source: Front Plant Sci. 2025 May 8;16:1561026. doi: 10.3389/fpls.2025.1561026 (PMC12095335; doi:10.3389/fpls.2025.1561026)
Supplement: Supplementary file 1 [file SupplementaryFile1.zip › Table S1 and Table S2.DOCX]

Table S1 Description of environmental factors

| Variable | Description | Variable | Description |
| --- | --- | --- | --- |
| Bio01 | Annual mean temperature | Bio18 | Precipitation of warmest quarter |
| Bio02 | Mean diurnal range (mean of monthly (max temp-min temp)) | Bio19 | Precipitation of coldest quarter |
| Bio03 | Isothermality ((Bio02/Bio07) * 100) | awc_class | Soil available water content |
| Bio04 | Temperature seasonality (standard deviation *100) | s_caco3 | Topsoil calcium Carbonate |
| Bio05 | Max temperature of warmest month | s_clay | Substrate-soil clay content |
| Bio06 | Min temperature of coldest month | s_oc | Substrate-soil organic carbon |
| Bio07 | Temperature annual range (Bi05-Bi06) | s_ph_h2o | Substrate-soil pH |
| Bio08 | Mean temperature of driest quarte | s_sand | Sediment content in the subsoil |
| Bio09 | Mean temperature of warmest quarter | t_caco3 | Topsoil carbonate or lime content |
| Bio10 | Mean temperature of coldest quarter | t_clay | Clay content in the upper soil |
| Bio11 | Annual precipitation | t_oc | Topsoil organic carbon |
| Bio12 | Precipitation of wettest month | t_ph_h2o | Topsoil pH |
| Bio13 | Precipitation of driest month | t_sand | Sand content |
| Bio14 | Precipitation seasonality (coefficient of variation) | aspect | Aspect |
| Bio15 | Precipitation of wettest quarter | elev | Elevation |
| Bio16 | Precipitation of driest quarter | slope | Slope |
| Bio17 | Driest quarterly precipitation |  |  |

Table S2 *A. tsao-ko* collection information table

| Sample No. | Location | Longitude | Latitude | Gatherer |
| --- | --- | --- | --- | --- |
| S1 | Honghe | 103.55959E | 23.14126N | Q. Yang |
| S2 | Lincang | 98.924457E | 28.47009N | J.D. Chen |
| S3 | Nujiang | 98.673738E | 27.74868N | Y. Yang |
| S4 | Tengchong | 98.500419E | 24.99543N | Y.Q. Liu |
| S5 | Malipo | 104.70756E | 23.13024N | Q. Yang |
| S6 | Maguan | 104.40041E | 23.01899N | Q. Yang |
| S7 | Wenshan | 105.63749E | 23.63138N | Q. Yang |
| S8 | Xishuangbanna | 100.45434E | 21.96216N | J.D. Chen |
