# Supplementary figures and images for: Assessment of Chinese suitable habitats of Amomum tsao-ko in different climatic conditions
Source: Front Plant Sci. 2025 May 8;16:1561026. doi: 10.3389/fpls.2025.1561026 (PMC12095335; doi:10.3389/fpls.2025.1561026)

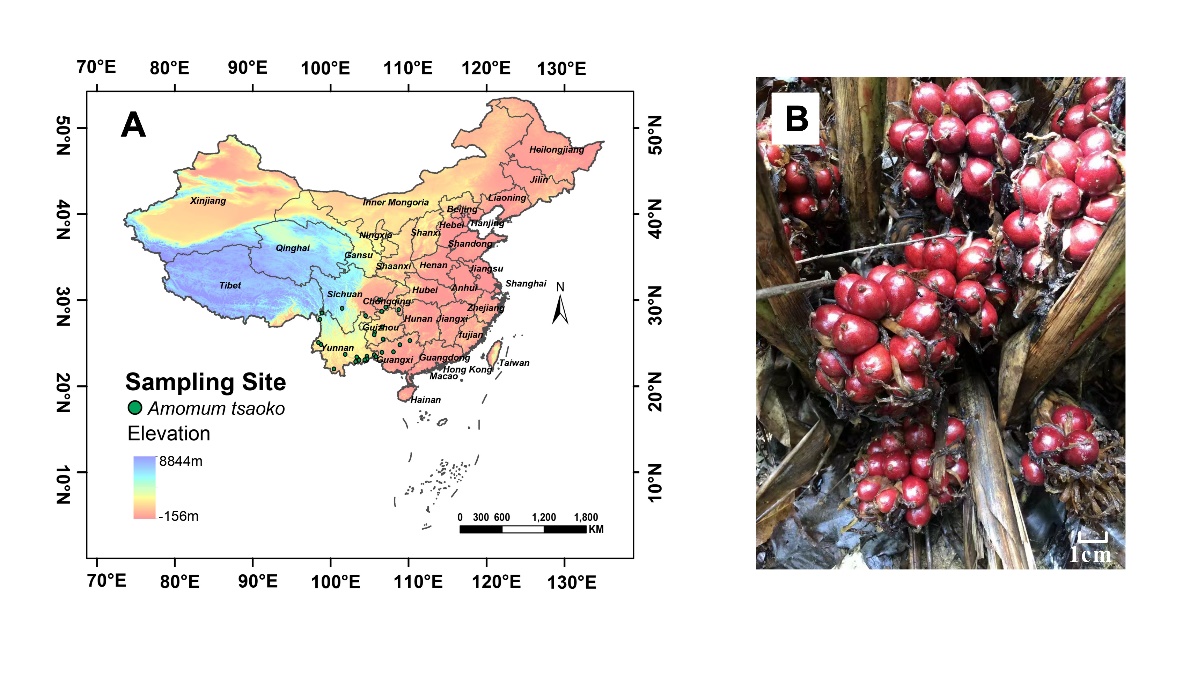


**Fig. S1** (A) Distribution points of *A. tsao-ko* in China. (B) *A. tsao-ko* in the field.

Supplement: Supplementary file 1 [file SupplementaryFile1.zip › Figure S1.DOCX]
